# Severe Acute Respiratory Syndrome and Particulate Matter Exposure: A Systematic Review

**DOI:** 10.3390/life13020538

**Published:** 2023-02-15

**Authors:** Sanjiti Podury, Sophia Kwon, Urooj Javed, Muhammad S. Farooqi, Yiwei Li, Mengling Liu, Gabriele Grunig, Anna Nolan

**Affiliations:** 1 Department of Medicine, Division of Pulmonary, Critical Care and Sleep Medicine, New York University Grossman School of Medicine (NYUGSoM), New York, NY 10016, USA; sanjiti.podury@nyulangone.org (S.P.); sophia.kwon@nyulangone.org (S.K.); urooj.javed@nyulangone.org (U.J.); muhammad.farooqi@nyulangone.org (M.S.F.); 2 Department of Population Health, Division of Biostatistics, New York University Grossman School of Medicine (NYUGSoM), New York, NY 10016, USA; yiwei.li@nyulangone.org (Y.L.); mengling.liu@nyulangone.org (M.L.); 3 Department of Medicine, Division of Environmental Medicine, New York University Grossman School of Medicine (NYUGSoM), New York, NY 10016, USA; grunig01@nyu.edu

**Keywords:** particulate matter, COVID-19, SARS, systematic review, mortality, incidence, prevalence, morbidity

## Abstract

Background: Particulate matter (PM) exposure is responsible for seven million deaths annually and has been implicated in the pathogenesis of respiratory infections such as severe acute respiratory syndrome (SARS). Understanding modifiable risk factors of high mortality, resource burdensome C19 and exposure risks such as PM is key to mitigating their devastating effects. This systematic review focuses on the literature available, identifying the spatial and temporal variation in the role of quantified PM exposure in SARS disease outcome and planning our future experimental studies. Methods: The systematic review utilized keywords adhered to the PRISMA guidelines. We included original human research studies in English. Results: Initial search yielded *N* = 906, application of eligibility criteria yielded *N* = 46. Upon analysis of risk of bias *N* = 41 demonstrated high risk. Studies found a positive association between elevated PM_2.5_, PM_10_ and SARS-related outcomes. A geographic and temporal variation in both PM and C19’s role was observed. Conclusion: C19 is a high mortality and resource intensive disease which devastated the globe. PM exposure is also a global health crisis. Our systematic review focuses on the intersection of this impactful disease-exposure dyad and understanding the role of PM is important in the development of interventions to prevent future spread of viral infections.

## 1. Introduction

Coronaviruses (CoV) are a common cause of respiratory disease. However, at least two novel CoVs have plagued humanity [1,2]. In 2003, the severe acute respiratory syndrome-CoV-1 (SARS-CoV-1) virus caused SARS, which affected over 8000 people worldwide and caused the death of over 700. In 2019, the latest novel CoV was identified in Wuhan, China, and was named SARS-CoV-2 [1]. By early 2020 the spread of SARS-CoV-2 was declared a pandemic [3]. Coronavirus disease 2019 (COVID-19; C19) was the official name given by the World Health Organization (WHO) to the disease caused by SARS-CoV-2 [3]. In addition, to the clinical signs and symptoms of cough and fever, radiographic findings in severe cases include lung infiltrates that require hospitalization. The COVID-19 pandemic is the third leading cause of death since 2020, and continues to threaten the health and well-being of humanity [4]. Therefore, it is imperative that we further evaluate exacerbating factors such as particulate matter (PM) that may allow us to mitigate morbidity and mortality.

Elevated PM exposure is associated with cancer, obstructive airway disease, ischemic heart disease, stroke, and respiratory infections resulting in 7-million deaths annually [5,6,7]. PM-induced pulmonary inflammation causes acute exacerbation of cardiovascular disease due to hypercoagulability [8]. PM_2.5_ is known to activate tumor-associated signaling pathways by microRNA dysregulation, DNA methylation and by increasing the levels of inflammatory cells, cytokines. Altered macrophage-mediated inflammatory response to viral infections due to PM exposure has been hypothesized to play a role in these adverse outcomes [9]. Exposure to PM_10_ increases RNA viral replication and worsens infections in human lung epithelial cells [10]. PM is a known carrier for several viruses and increased the transportation of avian influenza virus H5N1 across long distances during dust storms in Beijing, China [11]. A 10 µg/m^3^ increase in PM_2.5_ concentration per day was associated with a significant rise in the incidence of measles [12]. A high incidence of influenza, hospitalization with culture negative pneumonia and respiratory syncytial virus spread in children was observed with increased PM exposure [13,14,15]. These PM-associated end-organ effects are biologically plausible mediators of C19-related morbidity. Transmissibility, severity, and mortality of COVID infection was variable throughout the pandemic, likely from innate, genetic, socioeconomic, and environmental contributors such as PM. With growing exposures due to wildfires, dust storms, and domestic cooking, it is important to further understand the role of PM in susceptibility, severity, and mortality due to viral respiratory illnesses like SARS [16,17,18].

Prior reviews investigating SARS and PM have focused on acute vs. chronic duration of exposure to air pollution, including PM and other ambient exposures such as NO_2_, SO_2_, O_3_ [19] and PM as a transmitting vector [20]. Several studies have implicated PM as a severity risk of C19 [21,22,23,24]. Specifically, mortality doubled in regions with higher pollution compared to less polluted areas despite similar ICU admission rates [25]. Each 1 ng/m^3^ increase in PM was associated with 8% higher C19 confounder adjusted deaths [22,23]. These reviews were limited in terms of quantifying exposure levels of PM, lack of analysis of spatial/temporal variation and inadequate assessment of bias. Our systematic review focuses on the literature available, identifying the spatial, temporal variation and thereby laying the foundation for planning our future experimental studies that will quantify the adverse effects of PM exposure in SARS disease susceptibility, severity and mortality.

## 2. Materials and Methods

**Figure 1:**
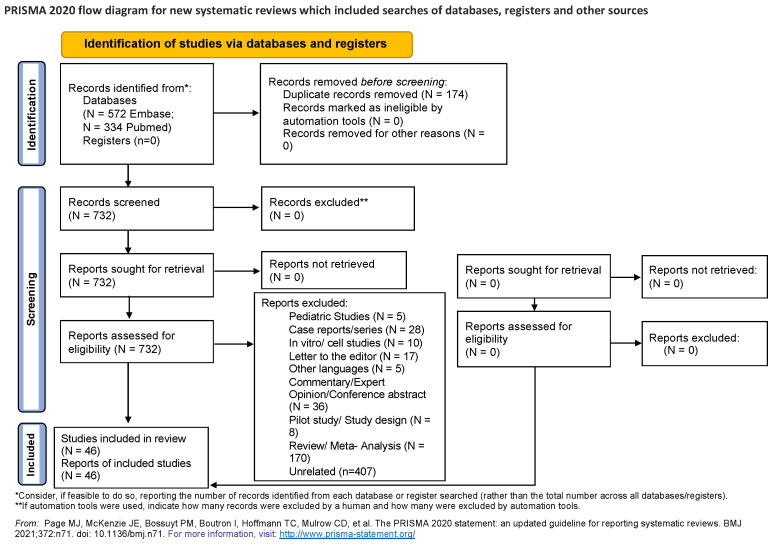
Study Design. Per Preferred reporting Items for systematic reviews and Meta-Analyses (PRISMA) Guidelines. PRISMA is an evidence-based minimum set of items for reporting in systematic reviews and meta-analyses [26]. http://www.prisma-statement.org/, accessed on 7 January 2023.

Details of our systematic review were registered with PROSPERO (CRD42022316121; https://www.crd.york.ac.uk/prospero/#myprospero, accessed on 15 April 2022). A systematic review of the literature was performed adhering to the Preferred Reporting Items for Systematic Reviews and Meta-analysis (PRISMA) guidelines (Figure 1) [26]. Our Population, Exposure, Outcome (PEO) question was “In the adult population (P) with diagnosed SARS infection we performed a systematic review to identify the role of quantifiable particulate matter exposure (E) in disease susceptibility, severity and mortality (O)”.

### 2.1. Search Terms

A PUBMED Medical Subject Headings (MeSH) search was performed and the following entry terms were identified: (Severe Acute Respiratory Syndrome Virus OR SARS-Related Coronavirus OR SARS Related Coronavirus OR SARS-CoV OR Urbani SARS-Associated Coronavirus OR Urbani SARS Associated Coronavirus OR SARS Coronavirus OR Severe acute respiratory syndrome-related coronavirus OR Severe acute respiratory syndrome related coronavirus OR SARS-Associated Coronavirus OR SARS Associated Coronavirus) and (Ultrafine Fibers OR Fiber, Ultrafine OR Airborne Particulate Matter OR Air Pollutants, Particulate OR Ambient Particulate Matter OR Ultrafine Particulate Matter OR Ultrafine Particles OR Ultrafine Particle).

We then searched for articles that addressed how quantifiable particulate matter exposure is associated with the risk, severity and mortality due to SARS infection.

For the purposes of this review we define PM as a mixture of solid particles and liquid droplets found in the air [27]. Severe acute respiratory syndrome is a viral respiratory illness caused by coronaviruses first detected in 2003. This review focuses on both SARS-CoV-1 and SARS-CoV-2.

Articles were selected based on the following inclusion criteria: (1) adult population; (2) articles written in English; (3) articles should include the concentration of the PM exposure in association with incidence, prevalence, severity and mortality due to SARS (SARS-CoV-1 and SARS-CoV-2); (4) studies after November 2002.

Articles were excluded if they: (1) were not in English language; (2) did not quantify the concentration of PM exposure; (3) involved any non-human subjects/in vitro work/cell studies/immunohistochemistry; (4) were conducted on pediatric population; (5) focused on gaseous pollutants; or (6) were not original research. Two independent researchers conducted the literature search and determined studies that met the inclusion/exclusion criteria. A third investigator resolved disagreements.

### 2.2. Quality Assessment and Risk of Bias (RoB)

**Figure 2:**
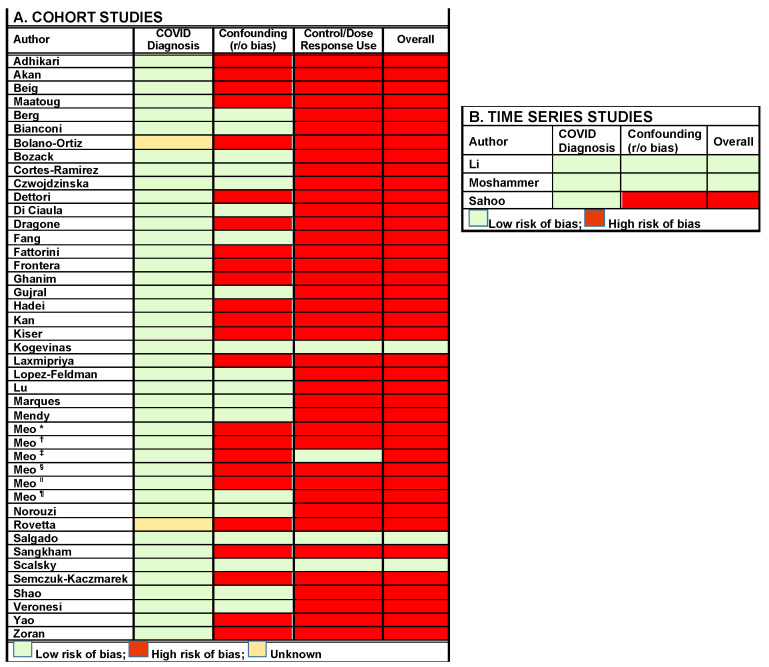
(**A**) Risk of Bias Assessment. Cohort Studies were evaluated in three main domains, i.e., outcome assessment, risk of confounding and presence of control dose-response comparator. (**B**) Risk of bias assessment. Time series studies, which were evaluated in two domains, i.e., outcome assessment, risk of confounding. Studies were color coded red or green for high vs. low risk of bias. Studies were categorized as low overall risk of bias if they were at low risk (green) for all key domains and high if any of the domains were high (red). RoB of * Meo [59]; ^†^ Meo [60]; ^‡^ Meo [61]; ^§^ Meo [68]; ^‖^ Meo [45]; ^¶^ Meo [62].

The overall RoB of the Cohort studies included in this review was determined with the approach described by Lee et al., 2020 (Figure 2A,B) [28]. We assessed three key domains of interest in the studies:

#### 2.2.1. Assessment of Outcomes

Studies that performed Nucleic Acid Amplification Test (NAAT) using reverse-transcription polymerase chain reaction (RT-PCR) to detect SARS-CoV-2 RNA from the upper respiratory tract, physician diagnosis or other clinical tests, were categorized as low risk for detection bias. For studies with unknown methods of diagnosis, we categorized them as unclear risk of detection bias.

#### 2.2.2. Adjustment for Confounding

Studies that adjusted for age, gender, individual levels of exposure or any other relevant covariates were categorized as low risk for this domain. Studies that did not adjust results for at least one covariate were categorized as high risk.

#### 2.2.3. Control/Dose–Response Comparator Was Used for Comparative Analysis

Studies that included a control group were categorized as low risk for this domain, whereas those that did not were categorized as high risk. The three key domains were assessed for overall risk of bias judgment. Studies were categorized as low overall risk of bias if it was at low risk for all key domains, and high if any of the domains were high. For the time series studies only two domains, i.e., assessment of outcomes and adjustment for confounding were considered to analyze the risk of bias.

### 2.3. Data Management/Extraction

Based on the inclusion and exclusion criteria, we screened and selected manuscripts (EndNote™ 20.1). Each article was screened for study design, patient characteristics, sample size, tools used, incidence, severity and mortality of SARS in association with quantifiable PM exposure. Results from each database search were filtered for human subjects, English language, publication date (after November 2002) and imported into EndNote. The references were then screened for duplicates. Only original research papers were then reviewed for title, abstract and full text to ascertain eligibility. The references cited in the relevant articles were also examined. All results were screened by SP and MSF and further independently evaluated by AN. Disagreements were resolved by consensus (see Supplementary Tables S1–S3).

### 2.4. Data Synthesis (GraphPad Prism 9; Ver 9.2.0)

**Figure 3:**
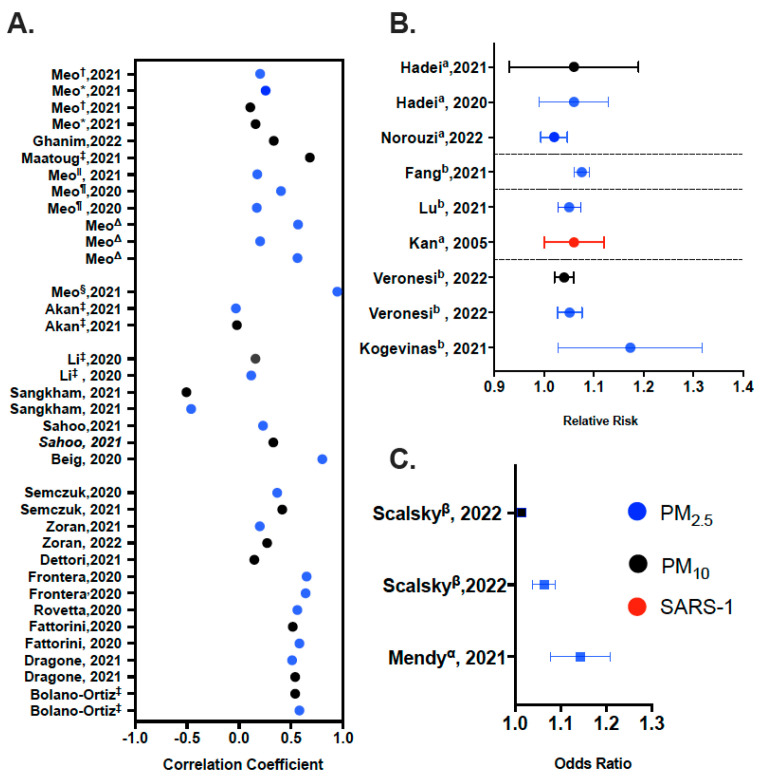
Overview of Data Synthesis. (**A**) Correlation coefficients were estimate for: PM_2.5 and 10_ and C19 Incidence and mortality for * Low green space countries and ^†^ High green space countries: Meo [62]; PM_2.5 and 10_ and C19 Incidence in Akan, Li, Sahoo and Fattorini, Sangkham; PM_2.5 and 10_ and C19 Prevalence in Zoran, Dragone; PM_2.5_ and C19 Incidence in Meo ^§^ [68], Meo ^‖^ [59], Meo ^Δ^ [60]; Bolano Ortiz; PM_2.5_ and C19 Prevalence in Semczuk; PM_2.5_ and C19 Mortality in Beig; PM_2.5_ and C19 Incidence, Prevalence, Mortality in Meo ^¶^ [61]; PM_2.5_ and C19 spread in Rovetta; PM_2.5_ and C19 Morbidity, Prevalence in Frontera; PM_10_ and C19 Incidence in Maatoug; PM10 and Mortality in Ghanim; PM_10_ and C19 Standardized (age) mortality ratio in Dettori. ^‡^ For studies where more than one city was analyzed, the highest correlation coefficient was plotted. Data grouped by region. (**B**) Relative risk of ^a^ mortality from C19 due to PM exposure and ^b^ Incidence of C19 due to PM exposure. Studies are grouped based on regions. (**C**) Odds ratios of ^α^ Hospitalization from C19 due to PM exposure and ^β^ Incidence of C19 due to PM exposure. Additional Information provided for relevant articles within each panel description.

Data was generated from sources using our review PEO question and summarized into tables and plots (Figure 3). Qualitative data synthesis was performed for studies, using thematic analysis that included three stages: (i). identifying information about the selected studies’ methodology and findings; (ii). organizing them into subheadings and descriptive categories; and (iii). developing these categories into analytic themes [29].

## 3. Results/Synthesis

### 3.1. Literature Search

A total of 906 studies (334 PubMed and 572 Embase) were identified after filtering for relevant studies (Figure 1, Supplementary Tables S1 and S2). After removing duplicates, *N* = 732 were assessed for inclusion (abstract and title review). Finally, 46 original research articles were considered eligible [25,30,31,32,33,34,35,36,37,38,39,40,41,42,43,44,45,46,47,48,49,50,51,52,53,54,55,56,57,58,59,60,61,62,63,64,65,66,67,68,69,70,71,72,73,74] Table 1. Data from screening and extraction is available (Supplementary Tables S1–S3).

Risk of bias assessment was performed for outcome, confounders and control group assessment. Of the three domains assessed for cohort studies, *N* = 2 studies were high risk for outcome assessment, *N* = 24 were high risk due to lack of adjustment for confounders and *N* = 39 were high risk due to lack of a control group in their studies. Overall, *N* = 3 studies had low risk of bias, whereas *N* = 40 had high risk of bias 

For the time series studies of the two domains assessed (outcome and confounders), *N* = 3 were low risk for outcome assessment and *N*= 1 was considered high risk for due to lack of adjustment for confounders. Overall, *N* = 2 were low risk for bias and *N* = 1 had high risk of bias.

### 3.2. Study Characteristics

As the C19 pandemic swept the globe from 31 December 2019, understanding the phenotype of both the disease and associated risk factors of disease severity has been challenging. Cohort studies were the predominant type (*N* = 43), while *N* = 3 were time-series studies [31,33,43]. The association of PM in the context of C19 surges, geographic location, and type of SARS infection are also of great interest and were further examined. In the context of these categories we will also discuss how PM_2.5_ and PM_10_ have played a role in SARS severity and spread.

### 3.3. Coronaviruses Have Been the Cause of Several Outbreaks

SARS-1 originated in Guangdong, China in 2003, and in six months had spread to more than two dozen countries resulting in at least 774 deaths [75]. Due to limited transmission, there are few studies that focus on this pathogen. Only one study that analyzed and noted positive association between PM and SARS-1 infection was noted by Kan et al., who found that for every 10 μg/m^3^ increase in PM_10_ the Relative risk (RR) of daily SARS mortality was 1.06 (1.00–1.12) [30]. There were few variants or recurrence of SARS-1 [76]. In contrast, the SARS-CoV-2 virus has several variants and lineages, and been responsible for at least 6 million deaths worldwide [77,78].

### 3.4. Temporal Relationship of PM and SARS

A decline in the incidence, mortality, and hospitalization was observed during the later pandemic period, from approximately late April–June 2020. A temporal analysis in Beijing from 25 April–31 May 2020 showed a declining trend in daily mortality count [30]. While this could be attributed to the implementation of more stringent mitigation measures, there are several other factors that may be relevant [79]. To understand the role of PM in the temporal variegation of outcomes, investigators have examined the impact of PM during the early and later phase of the pandemic. Dragone et al. noted that PM levels exceeded the daily limit during two early pandemic periods (16–25 February and 17–20 March 2020) in Italy. During this period, areas with the highest levels of ambient PM also had the highest number of infected populations [40]. Similarly, Li et al. noted a positive association between C19 cases and PM_2.5_ through a risks study using days with the highest and lowest incidence numbers in February [31]. Analysis investigating C19 cases in January–April 2020 in India showed that a 10 µg/m^3^ increase in PM_2.5_ and PM_10_ resulted in 2.21% (95% CI:1.13–3.29), 2.67% (95% CI: 0.33–5.01), increase in daily counts of C19 infected cases, respectively [33]. The early pandemic was the focus of 14/46 studies. PM was positively associated with C19 for a number of studies in the following aspects: incidence (*N* = 6); prevalence (*N* = 2); morbidity (*N* = 1) and mortality (*N* = 6). Negative association was observed with mortality (*N* = 1) and incidence (*N* = 1), and equivocal results were reported by *N* = 1 [34,52,63]. Of the 32 studies from the later pandemic period, PM was positively associated with C19 based on: incidence (*N* = 17); prevalence (*N* = 4); morbidity (*N* = 6) and mortality (*N* = 18). Negative association with incidence was observed in *N* = 2. Equivocal results reported by *N* = 4 [72].

### 3.5. Understanding Geographic Epidemiology Based on Region-Based Outcomes

Few, if any, areas of the globe have been left unaffected by the C19 pandemic. Meo et al. studied 17 countries across the globe and noted a significant positive association between PM and C19 incidence [45]. Certain areas like Malawi and Indonesia have been disproportionately impacted, and reported the highest case fatality rates on 8/26/22 [80].

Europe was the setting of (*N* = 16) studies [25,39,40,41,42,43,44,45,46,47,48,49,50,51,52,53]. A statistically significant correlation between PM_2.5_ and C19 cases was observed in Lombardy, Italy by Rovetta et al. [42]. With 1 unit increase in PM_2.5_ levels, the number of SARS-CoV-2 infections significantly increased by 0.1% in London [45]. Similarly, Scalsky et al. observed that PM_2.5_ levels recorded in 2010 were significantly associated with an increased SARS-CoV-2 positive testing likelihood (OR = 1.063, 95% CI 1.04–1.09) [46]. Overall, PM was positively associated with C19: mortality (*N* = 9); incidence (*N* = 6); prevalence (*N* = 4); morbidity (*N* = 2). Equivocal results were reported by (*N* = 2) [52,53].

Similarly, of the nine studies dedicated to Asia [30,31,32,33,34,35,36,37,38], a significant positive association was seen between PM_2.5_ and C19 cases in Wuhan (R^2^ = 0.13, *p* < 0.05) and Xiagan (R^2^ = 0.223, *p* < 0.01) [31]. In Hubei, a 10-µg/m^3^ rise in levels of PM_2.5_ (lag 0–14 was positively associated with RR of 1.050 (95% CI: 1.028–1.073) daily newly confirmed cases [32]. Overall, a positive association was observed between PM and C19 incidence (*N* = 4); mortality (*N* = 3); prevalence (*N* = 1).

Six studies with a focus on the Middle East were identified [66,67,68,69,70,71]. In a study conducted in Riyadh, Jeddah and Makkah, PM_10_ positively correlated to daily cases of C19 (Pearson correlation coefficients were 0.68, 0.54, 0.38, respectively) [66]. Similar observations were made in three Iranian cities where exposure to PM_2.5_ for several days showed significant association to confirmed cases [67]. Increase in PM_2.5_ due to a sandstorm in Saudi Arabia was associated with a significant increase in the number of SARS-CoV-2 cases (Spearman’s correlation coefficient ρ = 0.944 (<0.0001)) [68].

In the U.S, data from seven New York City (NYC) hospitals concluded that higher and long-term exposure to PM_2.5_ was associated with an increased risk of mortality (RR 1.11, 95%CI: 1.02–1.21) and ICU admission (RR 1.13, 95%CI: 1.00–1.28) per 1-µg/m^3^ increase in PM_2.5_ [55]. Similarly, a study from five regions noted that the number of cases significantly augmented with a rise in the levels of PM_2.5_ (ρ = 0.176, *p <* 0.001). PM was positively associated with C19: incidence rate (*N* = 7); mortality (*N* = 4) and prevalence (*N* = 1). A negative association with mortality was observed by *N* = 2 [63,64].

Similarly, as in 2/3 Latin American countries, PM and C19 incidence and mortality had a positive association. In contrast, one study reported equivocal results [72]. Specifically, an increase of 1 µg/m^3^ in PM_2.5_ increased the mortality risk by approximately 7.4% in Mexico City metropolitan area in October 2020 [73].

### 3.6. Aerodynamic Diameter of PM and SARS (PM_2.5_ vs. PM_10_)

PM is a heterogeneous mixture of solid particles and liquid droplets found in the air. It is commonly grouped by diameter into fine PM_2.5_ (<2.5 mm) and coarse PM_10_ (<10 mm). PM_2.5_ is more likely to travel and deposit deeper in the lungs like the alveolus, whereas PM_10_ can deposit on the surfaces of larger airways inducing inflammation. Ambient air pollutants are risk factors for cardiopulmonary diseases and responsible for over 6 million annual deaths.

The role of PM_2.5_ was assessed by 19/46 studies. PM_2.5_ was positively associated with C19: incidence (*N* = 11); mortality (*N* = 6); morbidity (*N* = 5); and prevalence (*N* = 1). Negative association with prevalence was seen in only one study [63].

PM_10_ was evaluated in eight studies, and it was positively associated with C19 incidence (*N* = 5); prevalence (*N* = 1); and mortality (*N* = 1). Finally, PM_10_ and PM_2.5_ were evaluated by *N* = 19 studies. A positive association with C19 incidence (*N* = 9); mortality (*N* = 5) was seen. Negative association was seen in *N* = 2 and equivocal results were identified in *N* = 1.

## 4. Discussion

Our systematic review identified the role of PM to be important in the incidence, mortality and morbidity due to SARS infection. These studies had significant differences in the populations, methods, and outcomes that were studied (Table 1). We identified three themes, temporality, PM size/dose, and spatial, which define the relationship of C19 with PM exposure. Evaluating the heterogeneous characteristics of the disease across different territories and phases of the pandemic is important to implement measures to contain spread.

Longer durations and higher levels of PM increased the risk of ICU admissions and deaths due to C19. Several mechanisms have been hypothesized. Oxidative stress and disruptive immune and/or neuroendocrine function can result in increased severity of viral pulmonary infections [81,82].

PM has been associated with enhanced infection with RNA viruses such as SARS [83]. PM concentration and virus dissemination were positively correlated in the spread of measles in several studies [12]. A 10 µg/m^3^ increase in PM_2.5_ was associated with increased measles incidence. A similar observation was also noted with respiratory syncytial virus, which causes bronchiolitis and pneumonia [15]. A study in Kuala Lumpur collected PM_2.5_ in four study sites and found the highest levels of SARS-CoV-2 RNA on PM_2.5_ [84]. PM exposure in murine models was associated with upregulated Angiotensin converting enzyme 2 (ACE2) and Transmembrane protease serine 2 (TMPRSS2), receptors required for SARS entry into host cells [85,86]. Exposure to PM induced Renin Angiotensin—aldosterone (RAAS) and Kallikrein—kinin systems (KKS), involved in cardiovascular and lung diseases. PM-induced damage to lung cells increases the inflammatory state which can increase the mortality and severity of C19 [87,88]. Therefore, it may be important to implement measures to reduce PM emissions in the atmosphere. Studies in this review further highlighted the importance of measures such as lockdown and movement restrictions, public awareness regarding pollution via media tools and professional programs and strengthening rural infrastructure that may limit the infectivity of SARS [69,72].

### 4.1. Geographic Epidemiology

Variation in C19 outcomes in different regions could be attributed to social determinants of health such as poverty, access to health care facilities and health literacy. Populations with limited resources also have a high prevalence of chronic health conditions [89,90]. Urban areas with industries had elevated levels of PM_2.5_. Spatial variation in the concentration of PM_2.5_ in some areas such as California’s central valley and Italy’s Po Valley can be contributed to geographical features with climate inversion events that trap these pollutants. The air trapping in these regions can also lead to chronic exposure to these particulates increasing the risk for respiratory and cardiovascular diseases which further enhances the risk [91].

### 4.2. Temporal Association

Worsening asthma and COPD leading to hospitalization has been noted with short-term (up to 24 h) exposures to PM_10_ [92]. Long-term PM_2.5_ is known to increase the risk of COPD, a known risk factor for severe C19 infection [93].

An NYC hospital-based study noted that mortality rates dropped from 25.6% in March to 7.6% in August 2020 [94]. This reduction in the number of severe C19 cases that was observed during the later pandemic phase could be attributed to multiple reasons, including the development of immunity due to availability of vaccinations, and treatment modalities including corticosteroids, targeted antiviral therapy, and anti-cytokine treatments. The quarantine restrictions and mask policies enforced by many countries also could have reduced exposure to ambient PM [95,96,97].

Strengths of Systematic Review. This review focuses on the environmental inducers of infectious diseases, a global health issue. It incorporates the variation in PM and the risk of Coronaviruses geographically. Manuscripts from across the globe were reviewed, which made this study more generalizable. Each article was screened for study design and was further subcategorized to understand temporal, spatial variability.

Our systematic review has several limitations. Since assessing the risk of bias inherently has some level of subjectivity, we categorized high- vs. low-risk studies using a determined set of criteria. While many of the studies that we assessed had a high risk of bias, future studies would benefit from assessing their data in the context of potential confounders including age, gender, and comorbidities. Several variants have been identified since the 2019; however, our manuscript does not discuss the disparity in disease outcomes for different variants [77]. The manuscripts that have been included in our review have not determined the variants that were present in their communities during their data acquisition [30,72,73]. There is also a variation in the PM concentration across countries and regions that could add to the bias. However, only *N* = 6 studies analyzed the spatial variation [32,38,40,55,62,65]. Also, additional studies not identified in the two large databases could have caused selection bias. There is a limitation in data available on SARS-1, which could be due to selection of manuscripts in the English language. Finally, while meta-analysis in the context of a systematic review may provide a more accurate effects estimate, for this to occur we would require source data availability and methodologic similarity. We therefore reviewed all 46 studies for available supplemental data and for similar methodologies and outcomes. Studies were grouped according to the statistical outcomes measured, i.e., relative risk vs. odd’s ratio. Seven out of 46 evaluated relative risks. Four out of these seven had supplementary data available. Out of the three studies that evaluated the odds ratio, one had supplementary data available. Unfortunately, only two studies performed the Generalized additive models (GAM) to analyze the association between PM and C19 outcomes; however, the C19 outcomes were different (Incidence vs. morbidity), which limits our ability to perform meta-analysis.

## 5. Conclusions

In conclusion, these studies have expanded our knowledge of PM exposure and its association with SARS infection. The review highlights the clinical impact of PM and the need for implementing measures to combat climate change and dangerous levels of environmental toxin. There was a spatial and temporal variation in the characteristics of the disease. Overall, it was seen that exposure to quantified PM was associated with increased incidence, mortality and morbidity to C19. Measures can be taken on both global and a personal level, such as improving air ventilation design and systems in enclosed spaces and buildings, restricting wildlife trading and deforestation, and training our healthcare professionals to educate masses on taking personal steps to ensure less production and exposure to pollution, such as using facemasks, and walking/cycling instead of motorized transport.

## 6. Future Plans

Future experimental studies will include developing our understanding of the role of PM in accentuating the response to pathogens such as C19, understanding the effects estimate for chronic vs. short-term exposure to PM, and in furthering our management of PM exposure to limit severity of viral infections. These projects will focus on quantifying the association of PM concentrations (by zip code and/or geocoding) and the incidence of C19 related morbidity and mortality.

## Figures and Tables

**Table 1:** Study Characteristics [30,31,32,33,34,35,36,37,38,39,40,41,42,43,44,45,46,47,48,49,50,51,52,53,54,55,56,57,58,59,60,61,62,63,64,65,66,67,68,69,70,71,72,73,74,75].

Study Author [Ref.]			Country	Exposure/ Design	Study Size/Time Period	Specimen/ Assay	End Points	Additional Findings
**1**	**ASIA**	Kan [30]	China	PM_10_ Cohort Study	Beijing/N = 37/ 25 April–31 May 2003	RT-PCR	Mortality	Mortality (RR) significant for PM_10_ and NO_2_ No association was seen with SO_2_
**2**		Li [31]	China	PM_2.5 and 10_ Time series Study	Wuhan and Xiaogan 26 January–29 February 2020	RT-PCR	Incidence	Correlation between the PM_2.5 and 10_ and incidencewas seen in Wuhan (R^2^ = 0.105, 0.174, respectively) Ambient air pollutants showed a positive association with incidence.
**3**		Lu [32]	China	PM_2.5_ Cohort study	41 cities/N = 22,970/ 20 January–29 February 2020	RT-PCR	Incidence	Incidence and ambient PM_2.5_ correlation were stronger for cities inside Hubei than those outside (highest RR at lag 0–14)
**4**		Sahoo [33]	India	PM_2.5 and 10_ Time series study	32 states and union territories/N = 21,700/30 January–24 April 2020	RT-PCR	Incidence	10 µg/m^3^ increase in PM_2.5and10_ resulted in 2.21% (95%CI: 1.13–3.29), 2.67% (95% CI: 0.33–5.01), increase in daily counts of cases, respectively.
**5**		Sangkham [34]	Thailand	PM_2.5, and 10_ Cohort Study	Bangkok City 30 March 2020	RT-PCR	Incidence	Significant negative association between PM and C19 cases (PM_10_ rs = −0.506, PM_2.5_ rs = −0.460)
**6**		Shao [35]	China	PM_2.5 and 10_ Cohort Study	Wuhan City 23 January–7 April 2020	RT-PCR	Mortality	Significant positive correlation between PM_2.5_ and the number of deaths per day.
**7**		Yao [36]	China	PM_2.5 and 10_ Cohort Study	49 cities February 2020	RT-PCR	Case fatality rate (CFR)	Positive associations between PM pollution and CFR around Hubei Province. Every 10 µg/m^3^ increase in PM_2.5 and 10_, the C19 CFR increased by 0.24% (0.01–0.48%) and 0.26% (0.00–0.51%), respectively.
**8**		Beig [37]	India	PM_2.5_ Cohort Study	6 Cities May 2022	RT-PCR	Mortality	PM_2.5_ baseline level and mortality/0.1 million population indicates significant correlation (r = 0.84 with *p*-value < 0.05) at 90% CI
**9**		Laxmipriya [38]	India	PM_2.5 and 10_ Cohort study	11 stations in Chennai city/July 2020	RT-PCR	Incidence	Areas with PM concentrations ranging from (38 to 90 mg/m^3^) reported with fewer positive cases (<5 cases). Areas covering above 91 to 195 mg/m^3^) had a positive association.
**10**	**EUROPE**	Frontera [25]	Italy	PM_2.5_ Cohort study	21 territories/ March 2020	RT-PCR	Prevalence, ICU admissions, Mortality	Positive Correlation between mean PM_2.5_ and total number of hospitalized patients (r = 0.62 *p* = 0.008), ICU admissions (r = 0.53 *p* = 0.005), total cases (r = 0.64 *p* = 0.007) and deaths r = 0.53 *p* = 0.032
**11**		Bianconii [39]	Italy	PM_2.5 and 10_ Cohort Study	20 provinces/N = 105,792 31 March 2020	RT-PCR	Incidence proportion and mortality	PM_2.5_ and PM_10_ were associated with: Incidence proportion irrespective of confounders (ß = 0.71, *p* = 0.003 and ß = 0.61, *p* = 0.031, respectively). Death rates across Italian regions PM_2.5_; ß = 0.68, *p* = 0.004 PM_10_; ß = 0.61, *p* = 0.029)
**12**		Dragone [40]	Italy	PM_2.5 and 10_ Cohort study	Lombard region/ N = 42,283/ 1 February–31 March 2020	RT-PCR	Prevalence	Positive correlation between spatial distribution of Prevalence with PM_10_ (r = 0.54) and PM_2.5_ (r = 0.51)
**13**		Fattorini [41]	Italy	PM_2.5 and 10_ Cohort study	N = 18,000 February–April 2020	RT-PCR	Incidence	Statistical correlation between cases and the air quality parameters in Italy PM_10_ r = 0.5168 *p* < 0.01, PM_2.5_ r = 0.5827 *p* < 0.01.
**14**		Rovetta [42]	Italy	PM_2.5 and 10_ Cohort study	Lombardy; N = 82,992/ February–March 2020	Unclear	Mortality rate	A statistically significant correlation between SARS-CoV-2 spread and PM_2.5_ in Lombardy during the first two weeks of March, (Correlation coefficient ρ = 0.56)
**15**		Moshammer [43]	Austria	PM_10_ Time Series study	Vienna/N = 1665/ March–April 2020	RT-PCR	Incidence	PM_10_ levels positively correlated (r = 0.014) with the risk of infection
**16**		Dettori [44]	Italy	PM_10_ Cohort Study	N = 60,359,546/ June 2020	RT-PCR	Standardized Mortality Ratio	PM_10_ (*p* = 0.001, 95% CI: 0.059–0.234) was independently associated with the case status. (r = 0.147 *p*-value = 0.001 95% CI 0.059–0.234)
**17**		Meo [45]	United Kingdom	PM_2.5_ Cohort Study	Cases 24 February–2 November 2020	RT-PCR	Incidence and Mortality	Cases significantly augmented with a rise in the levels of PM_2.5_ (ρ = 0.176, *p* < 0.001). Statistically insignificant relationship between PM_2.5_ and mortality (ρ = 0.029, *p* = 0.270)
**18**		Scalsky [46]	United Kingdom	PM_2.5_ Cohort	UK biobank; N = 15,156/16 March 2020–16 March 2021	RT-PCR	Incidence	PM_2.5_ levels were significantly associated with an increase in SARS-CoV-2 positive testing likelihood (OR = 1.063, 95% CI = 1.04–1.09)
**19**		Kogevinas [47]	Spain	PM_2.5_ Cohort Study	Catalonia/N = 9605/June–November 2020	RT-PCR	Incidence	Air pollution levels were not statistically significantly associated with SARS-CoV-2 infection.
**20**		Marques [48]	Spain	PM_10_ Cohort Study	Catalan hospitals N = 2112 April–June 2020	RT-PCR	C19 severity and mortality	PM_10_ showed the highest effects estimates (1.65, 95% CI 1.32–2.06) on severity and (2.37, 95% CI 1.71–3.32) mortality. An increase of 1 µg/m^3^ in PM_10_ causes an increase in 3.06% (95% CI 1.11–4.25%) of patients suffering severe disease and an increase of 2.68% (95% CI 0.53–5.58%) of deaths.
**21**		Veronesi [49]	Italy	PM_2.5 and 10_ Cohort Study	Varese; N = 62,848 25 February 2020–13 March 2021	RT-PCR	Incidence	PM_2.5_ was associated with a 5.1% increase in the incidence (95% CI 2.7% to 7.5%), corresponding to 294 additional cases per 100,000 person-years.
**22**		Zoran [50]	Spain	PM_2.5 and 10_ Cohort Study	6.61 million Inhabitants January 2020–July 2021	RT-PCR	Incidence, Prevalence and Mortality	Statistically significant correlation between PM_2.5_ and total cases (r = 0.20 *p* < 0.05). Significant correlation between PM_10_ and total cases (r = 0.27 *p* < 0.05) and daily new cases (r = 0.14 *p* < 0.05)
**23**		Semczuk–Kaczmarek [51]	Poland	PM_2.5 and 10_ Cohort Study	N = 18,016 4 March–15 May 2020	RT-PCR	Mortality and Morbidity	Statistically significant correlation between cases (per 100,000 population) and annual average concentration of PM_2.5_ (R^2^ = 0.367, *p* = 0.016), PM_10_ (R^2^ = 0.415, *p* = 0.009). Long-term exposure to air pollution, especially PM_2.5_ and 10, seems to play an essential role in prevalence and mortality
**24**		Di Ciaula [52]	Italy	PM_10_ Cohort Study	10 cities; N = 147 March–April 2020	RT-PCR	Mortality	PM_10_ exposure has no significant effect on mortality
**25**		Czwojdzinska [53]	Poland	PM_2.5 and 10_ Cohort study	N = 38,411,148 4 March–18 November 2020	RT-PCR	Incidence and mortality	Incidence independent of PM concentration
**26**	**USA**	Berg [54]	USA	PM_2.5_ Cohort study	Colorado N = 34,439 1 March–31 August 2020	RT-PCR	Incidence, hospitalization and mortality	1 µg/m^3^ increase in long-term PM_2.5_ concentrations is associated with a statistically significant 26% (RR: 1.26, 95% CI: 1.06–1.48) increase in the relative risk of hospitalizations, a 34% increase in mortality RR: 1.34, 95% CI: 1.02–1.77. Positive, insignificant increase in the RR of infections (1.10, 95% CI: 0.98–1.24).
**27**		Bozack [55]	USA	PM_2.5_ Cohort Study	Seven NYC hospitals N = 6542 8 March–30 August 2020	RT-PCR	Mortality, ICU admission, Intubation	PM_2.5_ exposure was not associated with the risk of intubation and mechanical ventilation (PM_2.5_: RR, 1.05 [95% CI: 0.91–1.20] per 1-µg/m^3^ increase.
**28**		Fang [56]	USA	3096 counties; PM_2.5_ Cohort Study	Cumulative Cases: 1st [May: 20,764] and 2nd [September: 34,596] surge in 2020	RT-PCR	Incidence	1 µg/m^3^ increase in annual average concentration of PM_2.5_ was associated with 7.60% increase in the cumulative risk, 95% CI between 3.82% and 11.51%.
**29**		Kiser [57]	USA	Nevada/PM_2.5_/Cohort Study	Regional hospital, Reno/15 May–20 October 2020	RT-PCR	Incidence	10 µg/m^3^ increase in the 7-day average PM_2.5_ concentration was associated with a 6.3% relative increase in the SARS-CoV-2 test positivity rate, with a 95% CI of 2.5 to 10.3%.
**30**		Mendy [58]	USA	PM_2.5_/Cohort study	Cincinnati/N = 14,783/ 13 March–30 September 2020	RT-PCR	Disease Severity	1 µg/m^3^ increase in 10-year annual average PM_2.5_ was associated with 18% higher hospitalization and 14% higher hospitalization
**31**		Meo [59]	USA	PM_2.5_/Cohort study	5 regions; N = 1192 13 March–31 December 2020	RT-PCR	Incidence and Mortality	For every 1 unit increase in PM_2.5_, the # of C19 infections significantly increased by 0.1%. PM_2.5_ and mortality were not statistically significant (ρ = 0.029, *p* = 0.270)
**32**		Meo [60]	USA	PM_2.5_/Cohort Study	California 20 March–16 September 2020	RT-PCR	Incidence, Prevalence and mortality	Significant positive correlation environmental pollutants PM_2.5_ and the number of daily cases PM_2.5_ µm and daily deaths had no relationship (r = −0.015, *p* = 0.842).
**33**		Meo [61]	USA	California /PM_2.5_/ Cohort study	California/19 March–15 August 2020	RT-PCR	Incidence and mortality	The rho-coefficient relation showed a significantly increased number of new cases 0.403 (*p* value < 0.001) and deaths 0.171 (*p* value < 0.001) with increasing levels of PM_2.5_
**34**		Meo [62]	USA	PM_2.5_ Cohort study	17 countries 25 January 2020–11 July 2021	RT-PCR	Incidence and Mortality	PM_2.5 and 10_, were significantly decreased (*p* < 0.0001) in environmentally highly green space countries compared to less-green countries. SARS-CoV-2- 2 cases and deaths were also significantly decreased in highly green countries compared to less-green countries.
**35**		Adhikari [63]	USA	PM_2.5_ Cohort study	New York/N = 42,023 cases/April 2020	RT-PCR	Incidence Rate Ratio, Mortality	Significant negative association between PM_2.5_ and new daily confirmed cases. One-unit increase in average PM_2.5_ (µg/m^3^) was associated with a 33.11% (95% CI: 31.04–35.22) decrease in daily new cases.
**36**		Gujral [64]	USA	PM_2.5_ Cohort study	California January–July 2020	RT-PCR	Incidence	Exposure to particulates, PM_2.5 and 10_, depicts a negative Association.
**37**	**AUSTRALIA**	Cortes-Ramirez [65]	Australia	PM_10_ Cohort study	New South Wales/ 2 March–2 August 2020	RT-PCR	Incidence	Higher wildfire burned areas were associated with higher incidence in both the random effects and spatial models after adjustment for sociodemographic factors (posterior mean = 1.32 (99% CI: 1.05–1.67) and 1.31 (99%CI: 1.03–1.65)). No association between the average PM_10_ level and incidence was found.
**38**	**MIDDLE EAST**	Maatoug [66]	Saudi Arabia	PM_10_ Cohort study	Riyadh, Jeddah, Makkah/N = 354,813 9 March–9 November 2020	RT-PCR	Incidence	Short-term exposure to PM_10_, NO_2_, O_3_ positively correlated with daily cases.
**39**		Hadei [67]	Iran	PM_2.5 and 10_ Cohort study	N = 114,964 February–January 2021	RT-PCR	Mortality and morbidity	Meta-analysis estimated that the RR for mortality, due to PM_2.5_ exposure was 1.06 (95% CI: 0.99, 1.13)
**40**		Meo [68]	Saudi Arabia	PM_2.5_ Cohort Study	Riyadh 20 February–2 April 2021	RT-PCR	Incidence and mortality	Increased PM_2.5_, NO_2_, CO, O_3_ was associated with a significant increase in cases. Association with mortality was insignificant
**41**		Ghanim [69]	Saudi Arabia	PM_10_ Cohort study	13 regions; N = 194,255 June 2020	RT-PCR	Incidence and Mortality	Positive correlation between mean PM_10_ and total number of cases r = 0.178 *p* = 0.623
**42**		Akan [70]	Turkey	PM_2.5 and 10_ Cohort study	15 Provinces N = 42,618,331 8 February–8 May 2021	RT-PCR	Incidence	PM_2.5 and 10_ displayed statistically significant negative associations with the number of cases. The spearman correlation coefficients for PM_10_ ranged between −0.02 and −0.62 and −0.03 to −0.34 for PM_2.5_
**43**		Norouzi [71]	Iran	PM_2.5_ Cohort study	12 cities/N = 73,080/ 1 March 2019–31 August 2020	RT-PCR	Incidence	Increased PM_2.5_ was not a predictor of mortality. PM_10_ excluded from the models due to an insignificant association with mortality.
**44**	**LATIN AMERICA**	Bolano-Ortiz [72]	Latin America and Caribbean	PM_2.5 and 10_ Cohort study	Ten cities/N = 56.95 million/1 April–31 May 2020	Unknown	Incidence rate and mortality	Negative correlation between total cases and PM_10_ (−0.44; *p* < 0.05;) in Mexico City and PM_2.5_ (−0.70; *p* < 0.01) in Bogota New and total cases showed the highest positive correlations with particulate matter PM_10_ (Sao Paulo and Santiago (0.35; *p* < 0.01; and Buenos Aires 0.54; *p* < 0.01)
**45**		Lopez-Feldman [73]	Mexico	PM_2.5_ Cohort study	Residents of (Hidalgo, and Mexico City) 7 October 2020	RT-PCR	Mortality	Three models used to analyze the relationship between long-term exposure and mortality. An average marginal effect of 0.0076 was noted.
**46**		Salgado [74]	Chile	PM_2.5 and 10_ Cohort Study	188 communes/N = 4574 May 2021	RT-PCR	Incidence and mortality	For each microgram per cubic meter increase, the incidence rate increased by 1.3% for PM_2.5_ and 0.9% for PM_10_. No statistically significant relationship with mortality rate

Subdivided by: 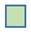 Positive association 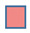 Negative association 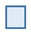 Unclear/Equivocal association 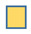 Late Pandemic 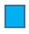 SARS-1.

## Data Availability

No new data were created in this study.

## Supplementary Materials

The following supporting information can be downloaded at: https://www.mdpi.com/2075-1729/13/2/538/s1. Supplementary Table S1. EMBASE Identified Manuscripts (N = 572); Supplementary Table S2. PUBMED Identified Manuscripts (N = 334); Supplementary Table S3. Manuscript Exclusions. S3a. Case reports/series; S3b. Commentary/Expert opinion/Conference Abstracts; S3c. Duplicates S3d. in vitro/Cell studies; S3e. Letters to the editor; S3f. Non-English Manuscripts; S3g. Pediatric Studies; S3h. Pilot studies/Study designs; S3i. Reviews/Meta-analysis; S3j. Unrelated.

## Abbreviations

PM	Particulate matter
SARS	Severe acute respiratory syndrome
CoV	Coronaviruses
ARDS	Acute respiratory distress syndrome
C19	COVID-19
PEO	Population, Exposure, Outcome
RoB	Risk of bias
RT-PCR	Reverse transcription polymerase chain reaction
RAAS	Renin angiotensin aldosterone system
TMPRSS2	Transmembrane serine protease 2
ACE	Angiotensin converting enzyme
KKS	(Kallikrein-kinin) systems
COPD	Chronic Obstructive Pulmonary Disease
PRISMA	Preferred Reporting Items for Systematic Reviews and Meta-Analyses
UK	United Kingdom
US	United States
CFR	Case Fatality Rate
