# Correction: Podury et al. Severe Acute Respiratory Syndrome and Particulate Matter Exposure: A Systematic Review. *Life* 2023, *13*, 538

**DOI:** 10.3390/life13091903

**Published:** 2023-09-13

**Authors:** Sanjiti Podury, Sophia Kwon, Urooj Javed, Muhammad S. Farooqi, Yiwei Li, Mengling Liu, Gabriele Grunig, Anna Nolan

**Affiliations:** 1 Department of Medicine, Division of Pulmonary, Critical Care and Sleep Medicine, New York University Grossman School of Medicine (NYUGSoM), New York, NY 10016, USA; sanjiti.podury@nyulangone.org (S.P.); sophia.kwon@nyulangone.org (S.K.); urooj.javed@nyulangone.org (U.J.);; 2 Department of Population Health, Division of Biostatistics, New York University Grossman School of Medicine (NYUGSoM), New York, NY 10016, USA; 3 Department of Medicine, Division of Environmental Medicine, New York University Grossman School of Medicine (NYUGSoM), New York, NY 10016, USA

## Error in Figure

In the original publication [1], there was an omission in Figure 3 as published. Figure 3A,C are missing coordinates for correlation coefficients and odds ratios. The corrected Figure 3 appears below. The authors state that the scientific conclusions are unaffected. This correction was approved by the Academic Editor. The original publication has also been updated.

## Figures and Tables

**Figure 3:**
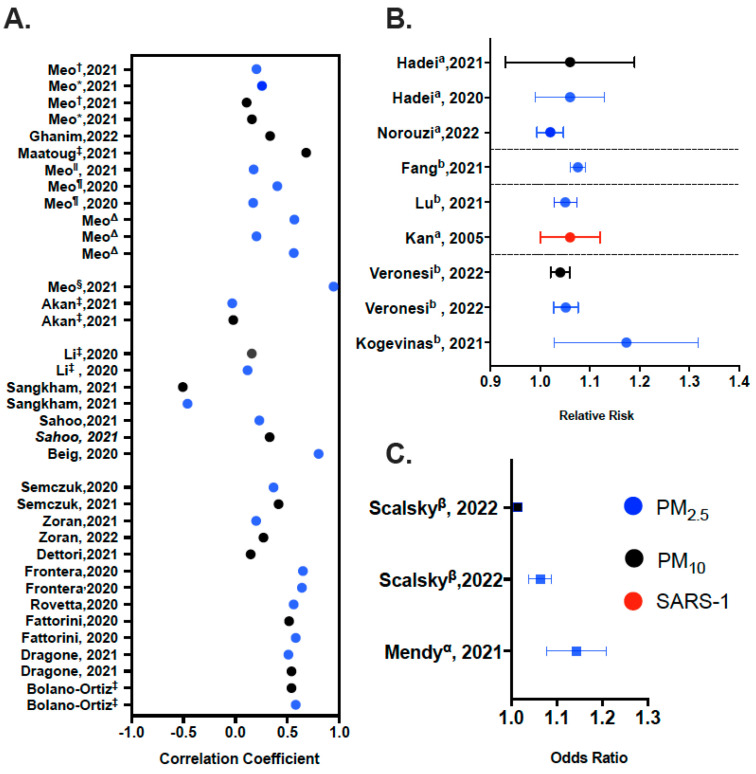
Overview of Data Synthesis. (**A**) Correlation coefficients were estimate for: PM_2.5 and 10_ and C19 Incidence and mortality for * Low green space countries and ^†^ High green space countries: Meo [62]; PM_2.5 and 10_ and C19 Incidence in Akan, Li, Sahoo and Fattorini, Sangkham; PM_2.5 and 10_ and C19 Prevalence in Zoran, Dragone; PM_2.5_ and C19 Incidence in Meo ^§^ [68], Meo ^‖^ [59], Meo ^Δ^ [60]; Bolano Ortiz; PM_2.5_ and C19 Prevalence in Semczuk; PM_2.5_ and C19 Mortality in Beig; PM_2.5_ and C19 Incidence, Prevalence, Mortality in Meo ^¶^ [61]; PM_2.5_ and C19 spread in Rovetta; PM_2.5_ and C19 Morbidity, Prevalence in Frontera; PM_10_ and C19 Incidence in Maatoug; PM10 and Mortality in Ghanim; PM_10_ and C19 Standardized (age) mortality ratio in Dettori. ^‡^ For studies where more than one city was analyzed, the highest correlation coefficient was plotted. Data grouped by region. (**B**) Relative risk of ^a^ mortality from C19 due to PM exposure and ^b^ Incidence of C19 due to PM exposure. Studies are grouped based on regions. (**C**) Odds ratios of ^α^ Hospitalization from C19 due to PM exposure and ^β^ Incidence of C19 due to PM exposure. Additional Information provided for relevant articles within each panel description.
