# Supplementary material for: Severe Acute Respiratory Syndrome and Particulate Matter Exposure: A Systematic Review
Source: Life (Basel). 2023 Feb 15;13(2):538. doi: 10.3390/life13020538 (PMC9962044; doi:10.3390/life13020538)
Supplement: Supplementary file 1 [file life-13-00538-s001.zip › life-2188539-supplementary.pdf]

Supplementary Table S1. EMBASE Identified Manuscripts (N= 572)

1	Abdalrhman AS, Wang C, Manalac A, Weersink M, Yassine AA, Betz V, et al. Modeling the efficiency of UV at 254nm for disinfecting the different layers within N95 respirators. Journal of biophotonics. 2021;14(10):e202100135.
2	Abrams RMC, Simpson DM, Navis A, Jette N, Zhou L, Shin SC. Small fiber neuropathy associated with SARS-CoV-2 infection. Muscle and Nerve. 2021.
**3**	Abrishami M, Daneshvar R, Emamverdian Z, Tohidinezhad F, Eslami S. Optic Nerve Head Parameters and Peripapillary Retinal Nerve Fiber Layer Thickness in Patients with Coronavirus Disease 2019. Ocular Immunology and Inflammation. 2021.
4	Accarino G, Lorenzetti S, Aloisio G. Assessing correlations between short-term exposure to atmospheric pollutants and COVID-19 spread in all Italian territorial areas. Environ Pollut. 2021;268(Pt A):115714.
5	Aggarwal S, Balaji S, Singh T, Menon GR, Mandal S, Madhumathi J, et al. Association between ambient air pollutants and meteorological factors with SARS-CoV-2 transmission and mortality in India: an exploratory study. Environ Health. 2021;20(1):120.
6	Aggarwal S, Balaji S, Singh T, Menon GR, Mandal S, Madhumathi J, et al. Association between ambient air pollutants and meteorological factors with SARS-CoV-2 transmission and mortality in India: an exploratory study. Environmental Health: A Global Access Science Source. 2021;20(1) (no pagination).
7	Agnihotri SP, Luis CVS, Kazamel M. Autonomic neuropathy as post-acute sequela of SARS-CoV-2 infection: a case report. Journal of NeuroVirology. 2022.
8	Akgun Y, Wu Y. Plasma exchange for acute motor axonal neuropathy variant guillain-barre syndrome in a pregnant woman with active systemic lupus erythematosus. Journal of Clinical Apheresis. 2021;36(2):269-70.
9	Alava JJ, Singh GG. Changing air pollution and CO<inf>2</inf> emissions during the COVID-19 pandemic: Lesson learned and future equity concerns of post-COVID recovery. Environmental Science and Policy. 2022;130:1-8.
10	Alharbi M, Burstin H. Small fiber neuropathy pots and gottron sign is COVID19 the culprit. Journal of General Internal Medicine. 2021;36(SUPPL 1):S269.
11	Ali A, Mathew R, Jadeja S, Schey R, Masri G. Small intestinal bacterial overgrowth after COVID-19 infection. American Journal of Gastroenterology. 2021;116(SUPPL):S1243-S4.
12	Ali K, Raja M. Coronavirus disease 2019 (COVID-19): challenges and management of aerosol-generating procedures in dentistry. Evidence-based dentistry. 2020;21(2):44-5.
13	Ali N, Fariha KA, Islam F, Mishu MA, Mohanto NC, Hosen MJ, et al. Exposure to air pollution and COVID-19 severity: A review of current insights, management, and challenges. Integr Environ Assess Manag. 2021;17(6):1114-22.
14	Allen JG, Ibrahim AM. Indoor Air Changes and Potential Implications for SARS-CoV-2 Transmission. JAMA - Journal of the American Medical Association. 2021;325(20):2112-3.
15	Alqadi GO, Saxena AK. Smoke and particulate filters in endoscopic surgery reviewed during COVID-19 pandemic. Journal of Pediatric Endoscopic Surgery. 2020;2(2):61-7.
16	Amundson DE, Shah US, de Necochea-Campion R, Jacobs M, LaRosa SP, Fisher CJ. Removal of COVID-19 Spike Protein, Whole Virus, Exosomes, and Exosomal MicroRNAs by the Hemopurifier Lectin-Affinity Cartridge in Critically Ill Patients With COVID-19 Infection. Frontiers in Medicine. 2021;8 (no pagination).
17	Anand U, Adelodun B, Pivato A, Suresh S, Indari O, Jakhmola S, et al. A review of the presence of SARS-CoV-2 RNA in wastewater and airborne particulates and its use for virus spreading surveillance. Environmental Research. 2021;196 (no pagination).
18	Anand U, Cabreros C, Mal J, Ballesteros F, Sillanpaa M, Tripathi V, et al. Novel coronavirus disease 2019 (COVID-19) pandemic: From transmission to control with an interdisciplinary vision. Environmental Research. 2021;197 (no pagination).
19	Anghel L, Popovici CG, Statescu C, Sascau R, Verdes M, Ciocan V, et al. Impact of hvac-systems on the dispersion of infectious aerosols in a cardiac intensive care unit. International Journal of Environmental Research and Public Health. 2020;17(18):1-17.
20	Anser MK, Godil DI, Khan MA, Nassani AA, Zaman K, Abro MMQ. The impact of coal combustion, nitrous oxide emissions, and traffic emissions on COVID-19 cases: a Markov-switching approach. Environ Sci Pollut Res Int. 2021;28(45):64882-91.
21	Anser MK, Godil DI, Khan MA, Nassani AA, Zaman K, Abro MMQ. The impact of coal combustion, nitrous oxide emissions, and traffic emissions on COVID-19 cases: a Markov-switching approach. Environmental science and pollution research international. 2021;28(45):64882-91.
22	Ansori ANM. A mini-review of the medicinal properties of Okra (Abelmoschus esculentus l.) and potential benefit against SARS-CoV-2. Indian Journal of Forensic Medicine and Toxicology. 2021;15(1):852-6.
23	Assaf SM, Tarasevych SP, Diamant Z, Hanania NA. Asthma and severe acute respiratory syndrome coronavirus 2019: Current evidence and knowledge gaps. Current Opinion in Pulmonary Medicine. 2021;27(1):45-53.
24	Assini A, Gandoglia I, Damato V, Rikani K, Evoli A, Del Sette M. Response to: "MuSK-positive myasthenia may be triggered not only by SARS-CoV-2". European Journal of Neurology. 2021;28(10):e82-e3.
25	Atiyani R, Mustafa S, Alsari S, Darwish A, Janahi EM. Clearing the air about airborne transmission of SARS-CoV-2. European Review for Medical and Pharmacological Sciences. 2021;25(21):6745-66.
26	Augenbraun BL, Lasner ZD, Mitra D, Prabhu S, Raval S, Sawaoka H, et al. Assessment and mitigation of aerosol airborne SARS-CoV-2 transmission in laboratory and office environments. Journal of occupational and environmental hygiene. 2020;17(10):447-56.
27	Aykaç N, Etiler N. COVID-19 mortality in Istanbul in association with air pollution and socioeconomic status: an ecological study. Environ Sci Pollut Res Int. 2022;29(9):13700-8.
28	Aykac N, Etiler N. COVID-19 mortality in Istanbul in association with air pollution and socioeconomic status: an ecological study. Environmental science and pollution research international. 2022;29(9):13700-8.

29	Aylward R, Bieber B, Guedes M, Pisoni R, Koranteng Tannor E, Dreyer G, et al. Pos-926 in-Centre Haemodialysis Centres Variably Affected by the Coronavirus-2019 Pandemic in Different Regions of the World: The International Society of Nephrology-Dialysis Outcomes Practice Patterns Study Survey. Kidney International Reports. 2022;7(2 Supplement):S404-S5.
30	Aylward R, Bieber B, Guedes M, Pisoni R, Tannor EK, Dreyer G, et al. The Global Impact of the COVID-19 Pandemic on In-Center Hemodialysis Services: An ISN-Dialysis Outcomes Practice Patterns Study Survey. Kidney International Reports. 2022.
31	Azuma K, Kagi N, Kim H, Hayashi M. Impact of climate and ambient air pollution on the epidemic growth during COVID-19 outbreak in Japan. Environmental Research. 2020;190 (no pagination).
32	Babaahmadi V, Amid H, Naeimirad M, Ramakrishna S. Biodegradable and multifunctional surgical face masks: A brief review on demands during COVID-19 pandemic, recent developments, and future perspectives. Science of the Total Environment. 2021;798 (no pagination).
33	Babkina AS, Ostrova IV, Yadgarov MY, Kuzovlev AN, Grechko AV, Volkov AV, et al. The Role of Von Willebrand Factor in the Pathogenesis of Pulmonary Vascular Thrombosis in COVID-19. Viruses. 2022;14(2) (no pagination).
34	Badani KK, Okhawere KE, Chen T, Korn TG, Razdan S, Meilika KN, et al. SARS-CoV-2 RNA Detected in Abdominal Insufflation Samples During Laparoscopic Surgery. European Urology. 2022;81(1):125-7.
35	Baldelli G, Aliano MP, Amagliani G, Magnani M, Brandi G, Pennino C, et al. Airborne Microorganism Inactivation by a UV-C LED and Ionizer-Based Continuous Sanitation Air (CSA) System in Train Environments. International Journal of Environmental Research and Public Health. 2022;19(3) (no pagination).
36	Barakat T, Muylkens B, Su BL. Is Particulate Matter of Air Pollution a Vector of Covid-19 Pandemic? Matter. 2020;3(4):977-80.
37	Barbieri P, Zupin L, Licen S, Torboli V, Semeraro S, Cozzutto S, et al. Molecular detection of SARS-CoV-2 from indoor air samples in environmental monitoring needs adequate temporal coverage and infectivity assessment. Environmental Research. 2021;198 (no pagination).
38	Barcala-Furelos R, Abelairas-Gomez C, Alonso-Calvete A, Cano-Noguera F, Carballo-Fazanes A, Martinez-Isasi S, et al. Safe On-Boat Resuscitation by Lifeguards in COVID-19 Era: A Pilot Study Comparing Three Sets of Personal Protective Equipment. Prehospital and disaster medicine. 2021;36(2):163-9.
39	Barcia RN, O'Rourke B, Nguyen SH, Tilles AW, Garg P, Gemmiti CV, et al. Clinical relevance of AKI trial data for severe COVID-19 patients. Journal of the American Society of Nephrology. 2020;31:307.
40	Baron YM. Are there medium to short-term multifaceted effects of the airborne pollutant PM(2.5) determining the emergence of SARS-CoV-2 variants? Med Hypotheses. 2021;158:110718.
41	Barros A, Queiruga-Pineiro J, Lozano-Sanroma J, Alcalde I, Gallar J, Fernandez-Vega Cueto L, et al. Small fiber neuropathy in the cornea of Covid-19 patients associated with the generation of ocular surface disease. Ocular Surface. 2022;23:40-8.
42	Batah SS, Benatti M, Syung L, Telini W, Capelozzi V, Cetlin A, et al. Respiratory disease, and treatment / thematic poster session COVID-19 outcome-based pathology reveals the birth of fibrosing interstitial pneumonitis. American Journal of Respiratory and Critical Care Medicine Conference: American Thoracic Society International Conference, ATS. 2021;203(9).
43	Bazzazpour S, Rahmatinia M, Mohebbi SR, Hadei M, Shahsavani A, Hopke PK, et al. The detection of SARS-CoV-2 RNA in indoor air of dental clinics during the COVID-19 pandemic. Environmental science and pollution research international. 2021;03.
44	Beesoon S, Behary N, Perwuelz A. Universal masking during COVID-19 pandemic: Can textile engineering help public health? Narrative review of the evidence. Preventive Medicine. 2020;139 (no pagination).
45	Bello-Lopez JM, Silva-Bermudez P, Prado G, Martinez A, Ibanez-Cervantes G, Cureno-Diaz MA, et al. Biocide effect against SARS-CoV-2 and ESKAPE pathogens of a noncytotoxic silver-copper nanofilm. Biomedical Materials (Bristol). 2022;17(1) (no pagination).
46	Belosi F, Conte M, Gianelle V, Santachiara G, Contini D. On the concentration of SARS-CoV-2 in outdoor air and the interaction with pre-existing atmospheric particles. Environ Res. 2021;193:110603.
47	Belosi F, Conte M, Gianelle V, Santachiara G, Contini D. On the concentration of SARS-CoV-2 in outdoor air and the interaction with pre-existing atmospheric particles. Environmental Research. 2021;193 (no pagination).
48	Beni AN, Dehghani A, Kianersi F, Ghanbari H, Habibi Z, Memarzadeh E, et al. Retinal findings of COVID-19 patients using Ocular coherence tomography angiography two to three months after infection: Ocular appearance recovered COVID-19 patient. Photodiagnosis and photodynamic therapy. 2022:102726.
49	Berg K, Romer Present P, Richardson K. Long-term air pollution and other risk factors associated with COVID-19 at the census tract level in Colorado. Environmental Pollution. 2021;287 (no pagination).
50	Bhattarai B, Sahulka SQ, Podder A, Hong S, Li H, Gilcrease E, et al. Prevalence of SARS-CoV-2 genes in water reclamation facilities: From influent to anaerobic digester. Science of the Total Environment. 2021;796 (no pagination).
51	Bian S, Shang M, Sawan M. Rapid biosensing SARS-CoV-2 antibodies in vaccinated healthy donors. Biosensors and Bioelectronics. 2022;204 (no pagination).
52	Bianconi V, Bronzo P, Banach M, Sahebkar A, Mannarino MR, Pirro M. Particulate matter pollution and the COVID-19 outbreak: results from Italian regions and provinces. Arch Med Sci. 2020;16(5):985-92.
53	Bitirgen G, Korkmaz C, Zamani A, Ozkagnici A, Zengin N, Ponirakis G, et al. Corneal confocal microscopy identifies corneal nerve fibre loss and increased dendritic cells in patients with long COVID. British Journal of Ophthalmology. 2021.
54	Bodnar B, Patel K, Ho W, Luo JJ, Hu W. Cellular mechanisms underlying neurological/neuropsychiatric manifestations of COVID-19. Journal of Medical Virology. 2021;93(4):1983-98.
55	Bondy SC, Wu M, Prasad KN. Attenuation of acute and chronic inflammation using compounds derived from plants. Experimental Biology and Medicine. 2021;246(4):406-13.
56	Bontempi E. A global assessment of COVID-19 diffusion based on a single indicator: Some considerations about air pollution and COVID-19 spread. Environmental Research. 2022;Part B. 204 (no pagination).

57	Bontempi E, Coccia M. International trade as critical parameter of COVID-19 spread that outclasses demographic, economic, environmental, and pollution factors. Environmental Research. 2021;201 (no pagination).
58	Borak J. Airborne Transmission of COVID-19. Occupational medicine (Oxford, England). 2020;70(5):297-9.
59	Boraldi F, Lofaro FD, Cossarizza A, Quaglino D. The "Elastic Perspective" of SARS-CoV-2 Infection and the Role of Intrinsic and Extrinsic Factors. International Journal of Molecular Sciences. 2022;23(3) (no pagination).
60	Borisova T, Komisarenko S. Air pollution particulate matter as a potential carrier of SARS-CoV-2 to the nervous system and/or neurological symptom enhancer: arguments in favor. Environmental science and pollution research international. 2021;28(30):40371-7.
61	Borisova T, Komisarenko S. Air pollution particulate matter as a potential carrier of SARS-CoV-2 to the nervous system and/or neurological symptom enhancer: arguments in favor. Environ Sci Pollut Res Int. 2021;28(30):40371-7.
62	Borro M, Di Girolamo P, Gentile G, De Luca O, Preissner R, Marcolongo A, et al. Evidence-Based Considerations Exploring Relations between SARS-CoV-2 Pandemic and Air Pollution: Involvement of PM2.5-Mediated Up-Regulation of the Viral Receptor ACE-2. Int J Environ Res Public Health. 2020;17(15).
63	Bossak BH, Andritsch S. COVID-19 and Air Pollution: A Spatial Analysis of Particulate Matter Concentration and Pandemic-Associated Mortality in the US. International Journal of Environmental Research and Public Health. 2022;19(1) (no pagination).
64	Bossak BH, Andritsch S. COVID-19 and Air Pollution: A Spatial Analysis of Particulate Matter Concentration and Pandemic-Associated Mortality in the US. Int J Environ Res Public Health. 2022;19(1).
65	Bozack A, Pierre S, DeFelice N, Colicino E, Jack D, Chillrud SN, et al. Long-Term Air Pollution Exposure and COVID-19 Mortality: A Patient-Level Analysis from New York City. Am J Respir Crit Care Med. 2021.
66	Braga F, Scarpa GM, Brando VE, Manfe G, Zaggia L. COVID-19 lockdown measures reveal human impact on water transparency in the Venice Lagoon. Science of the Total Environment. 2020;736 (no pagination).
67	Brandt EB, Beck AF, Mersha TB. Air pollution, racial disparities, and COVID-19 mortality. Journal of Allergy and Clinical Immunology. 2020;146(1):61-3.
68	Brandt EB, Mersha TB. Environmental Determinants of Coronavirus Disease 2019 (COVID-19). Curr Allergy Asthma Rep. 2021;21(3):15.
69	Brant-Zawadzki GM, Ockerse P, Brunson JR, Smith JL, McRae BR, Fonnesbeck A, et al. An Aerosol Containment and Filtration Tent for Intubation During the COVID-19 Pandemic. Surgical Innovation. 2021;28(2):226-30.
70	Bratosiewicz-Wasik J. Neuro-COVID-19: an insidious virus in action. Neurologia i neurochirurgia polska. 2021;13.
71	Brégeon F, Papazian L, Delpierre S, Kajikawa O, Payan MJ, Martin TR, et al. Role of proinflammatory activity contained in gastric juice from intensive care unit patients to induce lung injury in a rabbit aspiration model. Crit Care Med. 2008;36(12):3205-12.
72	Briguglio M, Bona A, Porta M, Dell'Osso B, Pregliasco FE, Banfi G. Disentangling the Hypothesis of Host Dysosmia and SARS-CoV-2: The Bait Symptom That Hides Neglected Neurophysiological Routes. Frontiers in Physiology. 2020;11 (no pagination).
73	Briz-Redon A, Belenguer-Sapina C, Serrano-Aroca A. Changes in air pollution during COVID-19 lockdown in Spain: A multi-city study. Journal of environmental sciences (China). 2021;101:16-26.
74	Briz-Redón Á, Belenguer-Sapiña C, Serrano-Aroca Á. A city-level analysis of PM(2.5) pollution, climate and COVID-19 early spread in Spain. J Environ Health Sci Eng. 2022:1-9.
75	Brocke S, Taft-Benz S, Robinette C, Knight N, Heise M, Jaspers I. Effects of Particulate Matter on SARS-CoV-2 Induced Antiviral Responses in Human Nasal Epithelial Cells. American Journal of Respiratory and Critical Care Medicine Conference: American Thoracic Society International Conference, ATS. 2021;203(9).
76	Brocke SA, Billings GT, Taft-Benz S, Alexis NE, Heise MT, Jaspers I. Woodsmoke particle exposure prior to SARS-CoV-2 infection alters antiviral response gene expression in human nasal epithelial cells in a sex-dependent manner. American journal of physiology Lung cellular and molecular physiology. 2022;02.
77	Brocke SA, Billings GT, Taft-Benz S, Alexis NE, Heise MT, Jaspers I. Woodsmoke particle exposure prior to SARS-CoV-2 infection alters antiviral response gene expression in human nasal epithelial cells in a sex-dependent manner. Am J Physiol Lung Cell Mol Physiol. 2022.
78	Brune Z, Kuschner CE, Mootz J, Davidson KW, Pena RCF, Ghanem MH, et al. Effectiveness of sars-cov-2 decontamination and containment in a covid-19 icu. International Journal of Environmental Research and Public Health. 2021;18(5):1-9.
79	Buja A, Manfredi M, De Luca G, Zampieri C, Zanovello S, Perkovic D, et al. Using failure mode, effect and criticality analysis to improve safety in the covid mass vaccination campaign. Vaccines. 2021;9(8) (no pagination).
80	Buonanno M, Welch D, Shuryak I, Brenner DJ. Far-UVC light (222nm) efficiently and safely inactivates airborne human coronaviruses. Scientific reports. 2020;10(1):10285.
81	Burgos-Blasco B, Guemes-Villahoz N, Morales-Fernandez L, Callejas-Caballero I, Perez-Garcia P, Donate-Lopez J, et al. Retinal nerve fibre layer and ganglion cell layer changes in children who recovered from COVID-19: A cohort study. Archives of Disease in Childhood. 2022;107(2):175-9.
82	Burgos-Blasco B, Guemes-Villahoz N, Vidal-Villegas B, Martinez-de-la-Casa JM, Donate-Lopez J, Martin-Sanchez FJ, et al. Optic nerve and macular optical coherence tomography in recovered COVID-19 patients. European Journal of Ophthalmology. 2022;32(1):628-36.
83	Calderon-Garciduenas L, Gonzalez-Maciel A, Reynoso-Robles R, Rodriguez-Lopez JL, Silva-Pereyra HG, Labrada-Delgado GJ, et al. Environmental Fe, Ti, Al, Cu, Hg, Bi, and Si nanoparticles in the atrioventricular conduction axis and the associated ultrastructural damage in young urbanites: Cardiac arrhythmias caused by anthropogenic, industrial, e-waste, and indoor nanoparticles. Environmental Science and Technology. 2021;55(12):8203-14.
84	Calderon-Garciduenas L, Torres-Jardon R, Franco-Lira M, Kulesza R, Gonzalez-Maciel A, Reynoso-Robles R, et al. Environmental Nanoparticles, SARS-CoV-2 Brain Involvement, and Potential Acceleration of Alzheimer's and

	Parkinson's Diseases in Young Urbanites Exposed to Air Pollution. Journal of Alzheimer's Disease. 2020;78(2):479-503.
85	Calderon-Garciduenas L, Torres-Jardon R, Franco-Lira M, Kulesza R, Gonzalez-Maciel A, Reynoso-Robles R, et al. Environmental Nanoparticles, SARS-CoV-2 Brain Involvement, and Potential Acceleration of Alzheimer's and Parkinson's Diseases in Young Urbanites Exposed to Air Pollution. Advances in Alzheimer's Disease. 2020;8:567-91.
86	Carbone M, Lednicky J, Xiao SY, Venditti M, Bucci E. Coronavirus 2019 Infectious Disease Epidemic: Where We Are, What Can Be Done and Hope For. Journal of Thoracic Oncology. 2021;16(4):546-71.
87	Carey M, Waheed W. New-onset dysesthesias following COVID-19 inoculation. Annals of Neurology. 2021;90(SUPPL 27):S200.
88	Carteni A, Di Francesco L, Martino M. How mobility habits influenced the spread of the COVID-19 pandemic: Results from the Italian case study. Science of the Total Environment. 2020;741 (no pagination).
89	Carteni A, Di Francesco L, Martino M. The role of transport accessibility within the spread of the Coronavirus pandemic in Italy. Safety Science. 2021;133 (no pagination).
90	Carugno M, Fedrizzi L, Borroni E, Consonni D, Pesatori AC. Air pollution exposure, SARS-CoV-2 infection, and immune response in a cohort of 3,700 healthcare workers. Safety and Health at Work. 2022;13(Supplement):S189-S90.
91	Caseiro A, von Schneidemesser E. APExpose_DE, an air quality exposure dataset for Germany 2010-2019. Scientific data. 2021;8(1):287.
92	Cazzolla Gatti R, Velichevskaya A, Tateo A, Amoroso N, Monaco A. Machine learning reveals that prolonged exposure to air pollution is associated with SARS-CoV-2 mortality and infectivity in Italy. Environ Pollut. 2020;267:115471.
93	Cazzolla Gatti R, Velichevskaya A, Tateo A, Amoroso N, Monaco A. Machine learning reveals that prolonged exposure to air pollution is associated with SARS-CoV-2 mortality and infectivity in Italy. Environmental Pollution. 2020;267 (no pagination).
94	Cennamo G, Reibaldi M, Montorio D, D'Andrea L, Fallico M, Triassi M. Optical Coherence Tomography Angiography Features in Post-COVID-19 Pneumonia Patients: A Pilot Study. American Journal of Ophthalmology. 2021;227:182-90.
95	Cennamo N, D'Agostino G, Perri C, Arcadio F, Chiaretti G, Parisio EM, et al. Proof of Concept for a Quick and Highly Sensitive On-Site Detection of SARS-CoV-2 by Plasmonic Optical Fibers and Molecularly Imprinted Polymers. Sensors. 2021;21(5).
96	Cennamo N, Pasquardini L, Arcadio F, Lunelli L, Vanzetti L, Carafa V, et al. SARS-CoV-2 spike protein detection through a plasmonic D-shaped plastic optical fiber aptasensor. Talanta. 2021;233:122532.
97	Chadeau-Hyam M, Bodinier B, Elliott J, Whitaker MD, Tzoulaki I, Vermeulen R, et al. Risk factors for positive and negative COVID-19 tests: A cautious and in-depth analysis of UK biobank data. International Journal of Epidemiology. 2020;49(5):1454-67.
98	Chakraborty P, Pasupuleti M, Jai Shankar MR, Bharat GK, Krishnasamy S, Dasgupta SC, et al. First surveillance of SARS-CoV-2 and organic tracers in community wastewater during post lockdown in Chennai, South India: Methods, occurrence and concurrence. Science of the Total Environment. 2021;778 (no pagination).
99	Chaovavanich A, Wongsawat J, Dowell SF, Inthong Y, Sangsajja C, Sanguanwongse N, et al. Early containment of severe acute respiratory syndrome (SARS); experience from Bamrasnaradura Institute, Thailand. Journal of the Medical Association of Thailand. 2004;87(10):1182-7.
100	Chari DA, Workman AD, Chen JX, Jung DH, Abdul-Aziz D, Kozin ED, et al. Aerosol Dispersion During Mastoidectomy and Custom Mitigation Strategies for Otologic Surgery in the COVID-19 Era. Otolaryngology - Head and Neck Surgery (United States). 2021;164(1):67-73.
101	Charitos IA, Ballini A, Bottalico L, Cantore S, Passarelli PC, Inchingolo F, et al. Special features of SARS-CoV-2 in daily practice. World Journal of Clinical Cases. 2020;8(18):3920-33.
102	Cheng VCC, Fung KSC, Siu GKH, Wong SC, Cheng LSK, Wong MS, et al. Nosocomial Outbreak of Coronavirus Disease 2019 by Possible Airborne Transmission Leading to a Superspreading Event. Clinical infectious diseases : an official publication of the Infectious Diseases Society of America. 2021;73(6):e1356-e64.
103	Chennakesavulu K, Reddy GR. The effect of latitude and PM(2.5) on spreading of SARS-CoV-2 in tropical and temperate zone countries. Environ Pollut. 2020;266(Pt 3):115176.
104	Cheung YH, Ma K, Van Leeuwen HC, Wasson MC, Wang X, Idrees KB, et al. Immobilized Regenerable Active Chlorine within a Zirconium-Based MOF Textile Composite to Eliminate Biological and Chemical Threats. Journal of the American Chemical Society. 2021;143(40):16777-85.
105	Ching J, Kajino M. Rethinking air quality and climate change after covid-19. International Journal of Environmental Research and Public Health. 2020;17(14):1-11.
106	Chirizzi D, Conte M, Feltracco M, Dinoi A, Gregoris E, Barbaro E, et al. SARS-CoV-2 concentrations and virus-laden aerosol size distributions in outdoor air in north and south of Italy. Environment International. 2021;146 (no pagination).
107	Chirumbolo S, Bjorklund G. The bimodal SARS-CoV-2 outbreak in Italy as an effect of environmental and allergic causes. Journal of Allergy and Clinical Immunology. 2020;146(2):331-2.
108	Christopherson DA, Yao WC, Lu M, Vijayakumar R, Sedaghat AR. High-Efficiency Particulate Air Filters in the Era of COVID-19: Function and Efficacy. Otolaryngology - Head and Neck Surgery (United States). 2020;163(6):1153-5.
109	Christophi CA, Sotos-Prieto M, Lan FY, Delgado-Velandia M, Efthymiou V, Gaviola GC, et al. Ambient temperature and subsequent COVID-19 mortality in the OECD countries and individual United States. Scientific reports. 2021;11(1):8710.
110	Chuang HC, Chen YY, Hsiao TC, Chou HC, Kuo HP, Feng PH, et al. Alteration in angiotensin-converting enzyme 2 by PM(1) during the development of emphysema in rats. ERJ Open Res. 2020;6(4).

111	Cilhoroz BT, DeRuisseau LR. Safety protocols in an exercise facility result in no detectable sars-CoV2 spread: A case study. Physiological Reports. 2021;9(14) (no pagination).
112	Coccia M. Factors determining the diffusion of COVID-19 and suggested strategy to prevent future accelerated viral infectivity similar to COVID. Sci Total Environ. 2020;729:138474.
113	Coccia M. How do low wind speeds and high levels of air pollution support the spread of COVID-19? Atmos Pollut Res. 2021;12(1):437-45.
114	Coccia M. Effects of the spread of COVID-19 on public health of polluted cities: results of the first wave for explaining the dejà vu in the second wave of COVID-19 pandemic and epidemics of future vital agents. Environ Sci Pollut Res Int. 2021;28(15):19147-54.
115	Coelho WEGDS, Perrechil F, Pedreira MLG, Lopes JL, Santos MVLD, Gabrieloni MC, et al. Safety and structural integrity of N95/PFF2 respirators decontamination. American Journal of Infection Control. 2021;49(10):1221-6.
116	Cognetti JS, Steiner DJ, Abedin M, Bryan MR, Shanahan C, Tokranova N, et al. Disposable photonics for cost-effective clinical bioassays: application to COVID-19 antibody testing. Lab on a Chip. 2021;21(15):2913-21.
117	Cohen SL, Liu G, Abrao M, Smart N, Heniford T. Perspectives on Surgery in the Time of COVID-19: Safety First. Journal of Minimally Invasive Gynecology. 2020;27(4):792-3.
118	Colacci A, Bortone G, Maffei G, Marchesi S, Mescoli A, Parmagnani F, et al. Environmental pollution and COVID-19: the molecular terms and predominant disease outcomes of their sweetheart agreement. Epidemiol Prev. 2020;44(5-6 Suppl 2):169-82.
119	Colacci A, Bortone G, Maffei G, Marchesi S, Mescoli A, Parmagnani F, et al. Inquinamento ambientale e COVID-19: le basi molecolari della loro interazione, Environmental pollution and COVID-19: the molecular terms and predominant disease outcomes of their sweetheart agreement. Epidemiologia e prevenzione. 2020;44(56 Supplement 2):169-82.
120	Collins DB, Farmer DK. Unintended Consequences of Air Cleaning Chemistry. Environmental Science and Technology. 2021;55(18):12172-9.
121	Collivignarelli MC, Abba A, Caccamo FM, Bertanza G, Pedrazzani R, Baldi M, et al. Can particulate matter be identified as the primary cause of the rapid spread of CoViD-19 in some areas of Northern Italy? Environmental science and pollution research international. 2021;26.
122	Comisi JC, Ravenel TD, Kelly A, Teich ST, Renne W. Aerosol and spatter mitigation in dentistry: Analysis of the effectiveness of 13 setups. Journal of Esthetic and Restorative Dentistry. 2021;33(3):466-79.
123	Comunian S, Dongo D, Milani C, Palestini P. Air Pollution and Covid-19: The Role of Particulate Matter in the Spread and Increase of Covid-19's Morbidity and Mortality. Int J Environ Res Public Health. 2020;17(12).
124	Conticini E, Frediani B, Caro D. Can atmospheric pollution be considered a co-factor in extremely high level of SARS-CoV-2 lethality in Northern Italy? Environmental Pollution. 2020;261 (no pagination).
125	Copat C, Cristaldi A, Fiore M, Grasso A, Zuccarello P, Signorelli SS, et al. The role of air pollution (PM and NO(2)) in COVID-19 spread and lethality: A systematic review. Environ Res. 2020;191:110129.
126	Copat C, Cristaldi A, Fiore M, Grasso A, Zuccarello P, Signorelli SS, et al. The role of air pollution (PM and NO<inf>2</inf>) in COVID-19 spread and lethality: A systematic review. Environmental Research. 2020;191 (no pagination).
127	Cortes MF, Espinoza EPS, Noguera SLV, Silva AA, de Medeiros MESA, Villas Boas LS, et al. Decontamination and re-use of surgical masks and respirators during the COVID-19 pandemic. International Journal of Infectious Diseases. 2021;104:320-8.
128	Cortes-Ramirez J, Michael RN, Knibbs LD, Bambrick H, Haswell MR, Wraith D. The association of wildfire air pollution with COVID-19 incidence in New South Wales, Australia. Science of the Total Environment. 2022;809 (no pagination).
129	Crane-Godreau MA, Clem KJ, Payne P, Fiering S. Vitamin D Deficiency and Air Pollution Exacerbate COVID-19 Through Suppression of Antiviral Peptide LL37. Front Public Health. 2020;8:232.
130	Crosby DL, Sharma A. Evidence-Based Guidelines for Management of Head and Neck Mucosal Malignancies during the COVID-19 Pandemic. Otolaryngology - Head and Neck Surgery (United States). 2020;163(1):16-24.
131	Crotty T, Sehgal R, Grundy J, Cahill R, Brennan D, Conneely J, et al. Cytoreductive surgery (Crs) and hyperthermic intraperitoneal chemotherapy (hipec) for peritoneal malignancy during the covid-19 pandemic. Irish Medical Journal. 2021;114(5) (no pagination).
132	Cruz R, Lima-Silva AE, Bertuzzi R, Hoinaski L. Exercising under particulate matter exposure: Providing theoretical support for lung deposition and its relationship with COVID-19. Environ Res. 2021;202:111755.
133	Cruz R, Lima-Silva AE, Bertuzzi R, Hoinaski L. Exercising under particulate matter exposure: Providing theoretical support for lung deposition and its relationship with COVID-19. Environmental Research. 2021;202 (no pagination).
134	Cui Y, Zhang ZF, Froines J, Zhao J, Wang H, Yu SZ, et al. Air pollution and case fatality of SARS in the People's Republic of China: an ecologic study. Environmental health : a global access science source. 2003;2(1) (no pagination).
135	Cui Y, Zhang ZF, Froines J, Zhao J, Wang H, Yu SZ, et al. Air pollution and case fatality of SARS in the People's Republic of China: an ecologic study. Environ Health. 2003;2(1):15.
136	Curtis L. PM<inf>2.5</inf>, NO<inf>2</inf>, wildfires, and other environmental exposures are linked to higher Covid 19 incidence, severity, and death rates. Environmental science and pollution research international. 2021;28(39):54429-47.
137	Curtius J, Granzin M, Schrod J. Testing mobile air purifiers in a school classroom: Reducing the airborne transmission risk for SARS-CoV-2. Aerosol Science and Technology. 2021;55(5):586-99.
138	Czwojdzińska M, Terpińska M, Kuźniarski A, Płaczkowska S, Piwowar A. Exposure to PM2.5 and PM10 and COVID-19 infection rates and mortality: A one-year observational study in Poland. Biomed J. 2021.
139	Dag Seker E, Erbahceci Timur IE. COVID-19: more than a respiratory virus, an optical coherence tomography study. International Ophthalmology. 2021;41(11):3815-24.
140	Daoud AK, Hall JK, Petrick H, Strong A, Piggott C. The Potential for Cloth Masks to Protect Health Care Clinicians From SARS-CoV-2: A Rapid Review. Annals of family medicine. 2021;19(1):55-62.

141	Datta M, Singh DD, Naqvi AR. Molecular Diagnostic Tools for the Detection of SARS-CoV-2. International Reviews of Immunology. 2021;40(1-2):143-56.
142	Deschasaux-Tanguy M, Srour B, Bourhis L, Arnault N, Druesne-Pecollo N, Esseddik Y, et al. Nutritional risk factors for SARS-CoV-2 infection: a prospective study within the NutriNet-Sante cohort. BMC Medicine. 2021;19(1) (no pagination).
143	Dettori M, Deiana G, Balletto G, Borruso G, Murgante B, Arghittu A, et al. Air pollutants and risk of death due to COVID-19 in Italy. Environ Res. 2021;192:110459.
144	Dey P, Saha SK, Sarkar S. Study of the interactions of sneezing droplets with particulate matter in a polluted environment. Phys Fluids (1994). 2021;33(11):113310.
145	Di Cerbo A. Air pollution and SARS-CoV-2 in the Po Valley: possible environmental persistence? Minerva Med. 2020;111(4):306-7.
146	Di Ciaula A, Bonfrate L, Portincasa P, Appice C, Belfiore A, Binetti M, et al. Nitrogen dioxide pollution increases vulnerability to COVID-19 through altered immune function. Environmental science and pollution research international. 2022;08.
147	Dicerbo A. Air pollution and SARS-CoV-2 in the Po Valley: Possible environmental persistence? Minerva Medica. 2020;111(4):306-7.
148	Din AR, Hindocha A, Patel T, Sudarshan S, Cagney N, Koched A, et al. Quantitative analysis of particulate matter release during orthodontic procedures: a pilot study. British dental journal. 2020;12.
149	Dolatshahi M, Sabahi M, Aarabi MH. Pathophysiological Clues to How the Emergent SARS-CoV-2 Can Potentially Increase the Susceptibility to Neurodegeneration. Molecular Neurobiology. 2021;58(5):2379-94.
150	Domingo JL, Marques M. The effects of some essential and toxic metals/metalloids in COVID-19: A review. Food and Chemical Toxicology. 2021;152 (no pagination).
151	Domingo JL, Marquès M, Rovira J. Influence of airborne transmission of SARS-CoV-2 on COVID-19 pandemic. A review. Environ Res. 2020;188:109861.
152	Domingo JL, Rovira J. Effects of air pollutants on the transmission and severity of respiratory viral infections. Environ Res. 2020;187:109650.
153	Domínguez-Amarillo S, Fernández-Agüera J, Cesteros-García S, González-Lezcano RA. Bad Air Can Also Kill: Residential Indoor Air Quality and Pollutant Exposure Risk during the COVID-19 Crisis. Int J Environ Res Public Health. 2020;17(19).
154	Dondi A, Betti L, Carbone C, Dormi A, Paglione M, Rinaldi M, et al. Understanding the environmental factors related to the decrease in Pediatric Emergency Department referrals for acute asthma during the SARS-CoV-2 pandemic. Pediatric Pulmonology. 2022;57(1):66-74.
155	Donia A, Hassan SU, Zhang X, Al-Madboly L, Bokhari H. Covid-19 crisis creates opportunity towards global monitoring & surveillance. Pathogens. 2021;10(3):1-28.
156	Dragone R, Licciardi G, Grasso G, Del Gaudio C, Chanussot J. Analysis of the chemical and physical environmental aspects that promoted the spread of sars-cov-2 in the lombard area. International Journal of Environmental Research and Public Health. 2021;18(3):1-21.
157	Dubey A, Kotnala G, Mandal TK, Sonkar SC, Singh VK, Guru SA, et al. Evidence of the presence of SARS-CoV-2 virus in atmospheric air and surfaces of a dedicated COVID hospital. Journal of Medical Virology. 2021;93(9):5339-49.
158	Duill FF, Schulz F, Jain A, Krieger L, van Wachem B, Beyrau F. The impact of large mobile air purifiers on aerosol concentration in classrooms and the reduction of airborne transmission of sars-cov-2. International Journal of Environmental Research and Public Health. 2021;18(21) (no pagination).
159	Dunker S, Hornick T, Szczepankiewicz G, Maier M, Bastl M, Bumberger J, et al. No SARS-CoV-2 detected in air samples (pollen and particulate matter) in Leipzig during the first spread. Sci Total Environ. 2021;755(Pt 1):142881.
160	Dutheil F, Baker JS, Navel V. COVID-19 as a factor influencing air pollution? Environmental Pollution. 2020;Part A. 263 (no pagination).
161	Dutheil F, Trousselard M, Navel V. SARS-CoV-2 as a protective factor for cardiovascular mortality? Atherosclerosis. 2020;304:64-5.
162	Duval JFL, van Leeuwen HP, Norde W, Town RM. Chemodynamic features of nanoparticles: Application to understanding the dynamic life cycle of SARS-CoV-2 in aerosols and aqueous biointerfacial zones. Advances in colloid and interface science. 2021;290:102400.
163	Dwari K. COVID-19 pandemic: A positive influence on lifestyle, economy, administration & environment; an overview. Indian Journal of Forensic Medicine and Toxicology. 2020;14(4):6605-10.
164	Edwards L, Rutter G, Iverson L, Wilson L, Chadha TS, Wilkinson P, et al. Personal exposure monitoring of PM<inf>2.5</inf> among US diplomats in Kathmandu during the COVID-19 lockdown, March to June 2020. Science of the Total Environment. 2021;772 (no pagination).
165	Ehtezazi T. The Potential Use of Cyclosporine Ultrafine Solution Pressurised Metered-Dose Inhaler in the Treatment of COVID-19 Patients. Recent advances in drug delivery and formulation. 2021;21.
166	Eke UA, Eke AC. Personal protective equipment in the siege of respiratory viral pandemics: strides made and next steps. Expert Review of Respiratory Medicine. 2021;15(4):441-52.
167	El-Zein RS, Cardinali S, Murphy C, Keeling T. COVID-19-associated meningoencephalitis treated with intravenous immunoglobulin. BMJ Case Reports. 2020;13(9) (no pagination).
168	Elkoundi A, Azzouzi A, El Wali A, Baite A, Bensghir M. Novel technique for safe fibreoptic tracheal intubation in COVID-19 patients. Anaesthesia Critical Care and Pain Medicine. 2020;39(4):465-6.
169	Elliott J, Bodinier B, Whitaker M, Delpierre C, Vermeulen R, Tzoulaki I, et al. COVID-19 mortality in the UK Biobank cohort: revisiting and evaluating risk factors. European Journal of Epidemiology. 2021;36(3):299-309.
170	Espejo W, Celis JE, Chiang G, Bahamonde P. Environment and COVID-19: Pollutants, impacts, dissemination, management and recommendations for facing future epidemic threats. Sci Total Environ. 2020;747:141314.
171	Esser C, Hochrath K, Schikowski T, Haarmann-Stemmann T. COVID-19 research: toxicological input urgently needed! Archives of Toxicology. 2020;94(7):2547-8.

172	Fang F, Mu L, Zhu Y, Rao J, Heymann J, Zhang ZF. Long-term exposure to pm<inf>2.5</inf>, facemask mandates, stay home orders and COVID-19 incidence in the united states. International Journal of Environmental Research and Public Health. 2021;18(12) (no pagination).
173	Farhangrazi ZS, Sancini G, Hunter AC, Moghimi SM. Airborne Particulate Matter and SARS-CoV-2 Partnership: Virus Hitchhiking, Stabilization and Immune Cell Targeting - A Hypothesis. Front Immunol. 2020;11:579352.
174	Farhangrazi ZS, Sancini G, Hunter AC, Moghimi SM. Airborne Particulate Matter and SARS-CoV-2 Partnership: Virus Hitchhiking, Stabilization and Immune Cell Targeting - A Hypothesis. Frontiers in Immunology. 2020;11 (no pagination).
175	Fedullo AL, Schiattarella A, Morlando M, Raguzzini A, Toti E, De Franciscis P, et al. Mediterranean diet for the prevention of gestational diabetes in the covid-19 era: Implications of Il-6 in diabesity. International Journal of Molecular Sciences. 2021;22(3):1-22.
176	Filippini T, Rothman KJ, Cocchio S, Narne E, Mantoan D, Saia M, et al. Associations between mortality from COVID-19 in two Italian regions and outdoor air pollution as assessed through tropospheric nitrogen dioxide. Sci Total Environ. 2021;760:143355.
177	Finsterer J. SARS-CoV-2 vaccinations may not only be complicated by GBS but also by distal small fibre neuropathy. Journal of Neuroimmunology. 2021;360 (no pagination).
178	Finsterer J. Neurological side effects of SARS-CoV-2 vaccinations. Acta Neurologica Scandinavica. 2022;145(1):5-9.
179	Finsterer J, Scorza FA, Scorza CA, Fiorini AC. SARS-CoV-2 and myasthenia. Journal of Medical Virology. 2021;93(7):4133-5.
180	Fiorito S, Soligo M, Gao Y, Ogulur I, Akdis CA, Bonini S. Is epithelial barrier hypothesis the key to understanding the higher incidence and excess mortality during COVID-19 pandemic? The case of Northern Italy. Allergy: European Journal of Allergy and Clinical Immunology. 2022.
181	Foladori P, Cutrupi F, Cadonna M, Manara S. Coronaviruses and SARS-CoV-2 in sewerage and their removal: Step by step in wastewater treatment plants. Environmental Research. 2022;207 (no pagination).
182	Folcarelli L, Del Giudice GM, Corea F, Angelillo IF. Intention to Receive the COVID-19 Vaccine Booster Dose in a University Community in Italy. Vaccines. 2022;10(2) (no pagination).
183	Fongaro G, Stoco PH, Souza DSM, Grisard EC, Magri ME, Rogovski P, et al. The presence of SARS-CoV-2 RNA in human sewage in Santa Catarina, Brazil, November 2019. Science of the Total Environment. 2021;778 (no pagination).
184	Forouzandeh P, O'Dowd K, Pillai SC. Face masks and respirators in the fight against the COVID-19 pandemic: An overview of the standards and testing methods. Safety Science. 2021;133 (no pagination).
185	Freire-Paspuel B, Vega-Marino P, Velez A, Castillo P, Gomez-Santos EE, Cruz M, et al. Cotton-Tipped Plastic Swabs for SARS-CoV-2 RT-qPCR Diagnosis to Prevent Supply Shortages. Frontiers in Cellular and Infection Microbiology. 2020;10 (no pagination).
186	Frontera A, Cianfanelli L, Vlachos K, Landoni G, Cremona G. Severe air pollution links to higher mortality in COVID-19 patients: The "double-hit" hypothesis. J Infect. 2020;81(2):255-9.
187	Frontera A, Cianfanelli L, Vlachos K, Landoni G, Cremona G. Severe air pollution links to higher mortality in COVID-19 patients: The "double-hit" hypothesis. Journal of Infection. 2020;81(2):255-9.
188	Fronza R, Lusic M, Schmidt M, Lucic B. Spatial-Temporal Variations in Atmospheric Factors Contribute to SARS-CoV-2 Outbreak. Viruses. 2020;12(6).
189	Gallo O. Risk for COVID-19 infection in patients with tobacco smoke-associated cancers of the upper and lower airway. Eur Arch Otorhinolaryngol. 2021;278(8):2695-702.
190	Gao YD, Ding M, Dong X, Zhang JJ, Kursat Azkur A, Azkur D, et al. Risk factors for severe and critically ill COVID-19 patients: A review. Allergy: European Journal of Allergy and Clinical Immunology. 2021;76(2):428-55.
191	Garbey M, Joerger G, Furr S. A Systems Approach to Assess Transport and Diffusion of Hazardous Airborne Particles in a Large Surgical Suite: Potential Impacts on Viral Airborne Transmission. Int J Environ Res Public Health. 2020;17(15).
192	Garg RK, Paliwal VK. Spectrum of neurological complications following COVID-19 vaccination. Neurological Sciences. 2022;43(1):3-40.
193	Gemignani F. Small Fiber Neuropathy and SARS-CoV-2 Infection. Another piece in the long COVID puzzle? Muscle and Nerve. 2022.
194	Generoso JS, de Quevedo JLB, Cattani M, Lodetti BF, Sousa L, Collodel A, et al. Neurobiology of COVID-19: how can the virus affect the brain? Brazilian Journal of Psychiatry. 2021;43(6):650-64.
195	George B, Megally M, Mrejen-Shakin K. Spontaneous Pneumothorax and Spontaneous Pneumomediastinum in Non-Intubated Patients in the Setting of Severe Acute Respiratory Syndrome Coronavirus 2. Chest. 2020;158(4 Supplement):A1653-A4.
196	Gettings J, Czarnik M, Morris E, Haller E, Thompson-Paul AM, Rasberry C, et al. Mask Use and Ventilation Improvements to Reduce COVID-19 Incidence in Elementary Schools - Georgia, November 16-December 11, 2020. Mmwr. 2021;Morbidity and mortality weekly report. 70(21):779-84.
197	Ghaffari HR, Farshidi H, Alipour V, Dindarloo K, Azad MH, Jamalidoust M, et al. Detection of SARS-CoV-2 in the indoor air of intensive care unit (ICU) for severe COVID-19 patients and its surroundings: considering the role of environmental conditions. Environmental science and pollution research international. 2021;05.
198	Ghanim AAJ. Analyzing the severity of coronavirus infections in relation to air pollution: evidence-based study from Saudi Arabia. Environmental science and pollution research international. 2021;27.
199	Ghoshal U, Vasanth S, Tejan N. A guide to laboratory diagnosis of Corona Virus Disease-19 for the gastroenterologists. Indian Journal of Gastroenterology. 2020;39(3):236-42.
200	Gianquintieri L, Brovelli MA, Pagliosa A, Bonora R, Sechi GM, Caiani EG. Geospatial Correlation Analysis between Air Pollution Indicators and Estimated Speed of COVID-19 Diffusion in the Lombardy Region (Italy). Int J Environ Res Public Health. 2021;18(22).

201	Gianquintieri L, Brovelli MA, Pagliosa A, Bonora R, Sechi GM, Caiani EG. Geospatial correlation analysis between air pollution indicators and estimated speed of covid-19 diffusion in the lombardy region (Italy). International Journal of Environmental Research and Public Health. 2021;18(22) (no pagination).
202	Gill AS, Oakley G, Error M, Kelly K, Orlandi R, Alt JA. Optimizing clinical productivity in the otolaryngology clinic during the COVID-19 pandemic. International Forum of Allergy and Rhinology. 2021;11(7):1121-3.
203	Girbardt C, Busch C, Al-Sheikh M, Gunzinger JM, Invernizzi A, Xhepa A, et al. Retinal vascular events after mrna and adenoviral-vectored covid-19 vaccines-a case series. Vaccines. 2021;9(11) (no pagination).
204	Gola M, Caggiano G, De Giglio O, Napoli C, Diella G, Carlucci M, et al. SARS-CoV-2 indoor contamination: considerations on anti-COVID-19 management of ventilation systems, and finishing materials in healthcare facilities. Annali di igiene : medicina preventiva e di comunita. 2021;33(4):381-92.
205	Gonçalves J, Koritnik T, Paragi M. Assessment of weather and atmospheric pollution as a co-factor in the spread of SARS-CoV-2. Acta Biomed. 2021;92(3):e2021094.
206	Goncalves J, Koritnik T, Paragi M. Assessment of weather and atmospheric pollution as a co-factor in the spread of SARS-CoV-2. Acta Biomedica. 2021;92(3) (no pagination).
207	Gotts JE, Chun L, Abbott J, Fang X, Takasaka N, Nishimura SL, et al. Cigarette smoke exposure worsens acute lung injury in antibiotic-treated bacterial pneumonia in mice. Am J Physiol Lung Cell Mol Physiol. 2018;315(1):L25-l40.
208	Gregorio PHP, Mariani AW, Brito JMLT, Santos BJM, Pego-Fernandes PM. Indoor Air Quality and Environmental Sampling as Support Tools to Detect SARS-CoV-2 in the Healthcare Setting. Journal of occupational and environmental medicine. 2021;63(11):956-62.
209	Grinshpun SA, Yermakov M, Kano M. Evaluation of AccuFIT 9000: A Novel Apparatus for Quantitative Fit Testing of Particulate Respirators. Annals of work exposures and health. 2021;65(4):458-62.
210	Guemes-Villahoz N, Burgos-Blasco B, Arribi-Vilela A, Arriola-Villalobos P, Vidal-Villegas B, Mendez-Fernandez R, et al. SARS-CoV-2 RNA detection in tears and conjunctival secretions of COVID-19 patients with conjunctivitis. Journal of Infection. 2020;81(3):452-82.
211	Gujral H, Sinha A. Association between exposure to airborne pollutants and COVID-19 in Los Angeles, United States with ensemble-based dynamic emission model. Environ Res. 2021;194:110704.
212	Gujral H, Sinha A. Association between exposure to airborne pollutants and COVID-19 in Los Angeles, United States with ensemble-based dynamic emission model. Environmental Research. 2021;194 (no pagination).
213	Gupta S, Dubey H, Rai A, Singh P, Jhunjhunwala N, Singh S. Desirable and Undesirable Effects of Air Purifier in Clinical Settings during Covid-19 Pandemic. European Journal of Molecular and Clinical Medicine. 2020;7(6):233-6.
214	Gupta S, Kalra J, Goyal H, Kumar V. Analysis on worldwide coronavirus (COVID-19) cases. Eastern Journal of Medicine. 2020;25(4):591-9.
215	Hakami AR, Dobie G. Studying the effect of particulate matter as SARS-CoV-2 transmitters. J Public Health Res. 2021;11(1).
216	Hammond A, Khalid T, Thornton HV, Woodall CA, Hay AD. Should homes and workplaces purchase portable air filters to reduce the transmission of SARS-CoV-2 and other respiratory infections? A systematic review. PLoS ONE. 2021;16(4 April 2021) (no pagination).
217	Hansell AL, Villeneuve PJ. Invited Perspective: Ambient Air Pollution and SARS-CoV-2: Research Challenges and Public Health Implications. Environ Health Perspect. 2021;129(11):111303.
218	Hansell AL, Villeneuve PJ. Invited perspective: Ambient air pollution and sars-cov-2: Research challenges and public health implications. Environmental Health Perspectives. 2021;129(11) (no pagination).
219	Hao W, Wu J, Zhao X, Liang D, Yu X, Cao H, et al. Quantitative Evaluation of Aerosol Generation from Non-contact Tonometry and its Correlation with Tear Film Characteristics. Advances in Therapy. 2021;38(6):3066-76.
220	Hawrylkowicz V, Lietz-Kijak D, Kazmierczak-Siedlecka K, Solek-Pastuszka J, Stachowska L, Folwarski M, et al. Patient nutrition and probiotic therapy in covid-19: What do we know in 2021? Nutrients. 2021;13(10) (no pagination).
221	Hazan SC, Daniels J. The role of roseburia in COVID-19 pathogenesis. American Journal of Gastroenterology. 2021;116(SUPPL):S70.
222	He LH, Ren LF, Li JF, Wu YN, Li X, Zhang L. Intestinal Flora as a Potential Strategy to Fight SARS-CoV-2 Infection. Frontiers in Microbiology. 2020;11 (no pagination).
223	Heijink IH, Hackett TL, Pouwels SD. Effects of cigarette smoking on SARS-CoV-2 receptor ACE2 expression in the respiratory epithelium⁺. Journal of Pathology. 2021;253(4):351-4.
224	Helm Ii S, Harmon PC, Noe C, Calodney AK, Abd-Elsayed A, Knezevic NN, et al. Transforaminal Epidural Steroid Injections: A Systematic Review and Meta-Analysis of Efficacy and Safety. Pain physician. 2021;24(S1):S209-S32.
225	Helm S, Harmon PC, Noe C, Calodney A, Abd-Elsayed A, Knezevic NN, et al. Transforaminal epidural steroid injections: A systematic review and meta-analysis of efficacy and safety. Pain Physician. 2021;24(S1):209-32.
226	Hemsath JR, Liaci AM, Rubin JD, Parrett BJ, Lu SC, Nguyen TV, et al. Ex Vivo and In Vivo CD46 Receptor Utilization by Species D Human Adenovirus Serotype 26 (HAdV26). Journal of Virology. 2022;96(3) (no pagination).
227	Herghelegiu AM, Nuta CR, Bajenaru OL, Taranu SM, Ilie AC, Alexa ID, et al. COVID-19 and neurocognitive function. European Geriatric Medicine. 2021;12(SUPPL 1):S94-S5.
228	Hill WC, Hull MS, MacCuspie RI. Testing of Commercial Masks and Respirators and Cotton Mask Insert Materials using SARS-CoV-2 Virion-Sized Particulates: Comparison of Ideal Aerosol Filtration Efficiency versus Fitted Filtration Efficiency. Nano letters. 2020;20(10):7642-7.
229	Ho CY, Salimian M, Holler J, Burke A, Ames H, Hegert J, et al. Mechanism of Olfactory Dysfunction in COVID-19 Infection. Journal of Neuropathology and Experimental Neurology. 2021;80(6):587.
230	Howard BE. High-Risk Aerosol-Generating Procedures in COVID-19: Respiratory Protective Equipment Considerations. Otolaryngology - Head and Neck Surgery (United States). 2020;163(1):98-103.
231	Hu L, Deng WJ, Ying GG, Hong H. Environmental perspective of COVID-19: Atmospheric and wastewater environment in relation to pandemic. Ecotoxicology and Environmental Safety. 2021;219 (no pagination).

232	Huang J, Zheng M, Tang X, Chen Y, Tong A, Zhou L. Potential of SARS-CoV-2 to Cause CNS Infection: Biologic Fundamental and Clinical Experience. Frontiers in Neurology. 2020;11 (no pagination).
233	Huang JC, Chang YF, Chen KH, Su LC, Lee CW, Chen CC, et al. Detection of severe acute respiratory syndrome (SARS) coronavirus nucleocapsid protein in human serum using a localized surface plasmon coupled fluorescence fiber-optic biosensor. Biosensors and Bioelectronics. 2009;25(2):320-5.
234	Huang WC, Zhou S, He X, Chiem K, Mabrouk MT, Nissly RH, et al. SARS-CoV-2 RBD Neutralizing Antibody Induction is Enhanced by Particulate Vaccination. Advanced materials (Deerfield Beach, Fla). 2020;32(50):e2005637.
235	Humphreys H, Fitzpatrick F. Airborne transmission of covid-19: Implications for irish hospitals. Irish Medical Journal. 2020;113(7):1-3.
236	Husain-Syed F, Birk HW, Wilhelm J, Ronco C, Ranieri VM, Karle B, et al. Extracorporeal Carbon Dioxide Removal Using a Renal Replacement Therapy Platform to Enhance Lung-Protective Ventilation in Hypercapnic Patients With Coronavirus Disease 2019-Associated Acute Respiratory Distress Syndrome. Frontiers in Medicine. 2020;7 (no pagination).
237	Ibarra-Espinosa S, Dias de Freitas E, Ropkins K, Dominici F, Rehbein A. Negative-Binomial and quasi-poisson regressions between COVID-19, mobility and environment in Sao Paulo, Brazil. Environmental Research. 2022;Part D. 204 (no pagination).
238	Ilardi A, Chieffi S, Ilardi CR. Predictive Role of Population Density and Use of Public Transport for Major Outcomes of SARS-CoV-2 Infection in the Italian Population: An Ecological Study. Journal of Research in Health Sciences. 2021;21(2) (no pagination).
239	Ionita C, Marcelli D, Nita C, Anton C, Berca S, Vacar S, et al. Comparison of antibody response to two different mRNA Covid-19 vaccines in patients on hemodialysis. Journal of Nephrology. 2022;35(1):143-51.
240	Irandoust F, Dini S. A new perspective of aroma face mask on COVID-19 pandemic. Journal of Medical Engineering and Technology. 2022.
241	Islam MT, Hossen M, Kamaz Z, Zali A, Kumar M, Docea AO, et al. The role of hmgb1 in the immune response to sars-cov-2 infection: From pathogenesis towards a new potential therapeutic target. Farmacia. 2021;69(4):621-34.
242	Jagtap PK, Kolla V. A comprehensive report on critical aspects of the virus that caged us. European Journal of Molecular and Clinical Medicine. 2020;7(3):2587-601.
243	Jai Rexlin PE, Anjali AK, Roy A. Has lockdown led to the restoration of nature-a review. International Journal of Pharmaceutical Research. 2021;13(1):1653-8.
244	Jain A, Talwar D, Kumar S. Spectrum of respiratory involvement in COVID 19 era; an overview. Indian Journal of Forensic Medicine and Toxicology. 2020;14(4):6593-9.
245	Jakubowski B, Mehta R. A Unique Case of COVID Pneumonia, Tuberculosis, and Silicosis. American Journal of Respiratory and Critical Care Medicine Conference: American Thoracic Society International Conference, ATS. 2021;203(9).
246	Jeican II, Gheban D, Barbu-Tudoran L, Inisca P, Albu C, Ilies M, et al. Respiratory nasal mucosa in chronic rhinosinusitis with nasal polyps versus covid-19: Histopathology, electron microscopy analysis and assessing of tissue interleukin-33. Journal of Clinical Medicine. 2021;10(18) (no pagination).
247	Jephcote C, Hansell AL, Adams K, Gulliver J. Changes in air quality during COVID-19 'lockdown' in the United Kingdom. Environmental Pollution. 2021;272 (no pagination).
248	Jones GW, Monopoli MP, Campagnolo L, Pietroiusti A, Tran L, Fadeel B. No small matter: A perspective on nanotechnology-enabled solutions to fight COVID-19. Nanomedicine. 2020;15(24):2411-27.
249	Jones HA, Salib RJ, Harries PG. Reducing Aerosolised Particles and Droplet Spread in Endoscopic Sinus Surgery during COVID-19. The Laryngoscope. 2020;15.
250	Jones HAS, Salib RJ, Harries PG. Reducing Aerosolized Particles and Droplet Spread in Endoscopic Sinus Surgery during COVID-19. Laryngoscope. 2021;131(5):956-60.
251	Jones RM, Brosseau LM. Aerosol transmission of infectious disease. Journal of Occupational and Environmental Medicine. 2015;57(5):501-8.
252	Kallivoulos S, Giantzi V, Parissis D, Grigoriadis N. MRI-negative acute transverse myelitis during COVID-19 pandemic: A case report. European Journal of Neurology. 2021;28(SUPPL 1):590.
253	Kan HD, Chen BH, Fu CW, Yu SZ, Mu LN. Relationship between ambient air pollution and daily mortality of SARS in Beijing. Biomed Environ Sci. 2005;18(1):1-4.
254	Karan A, Ali K, Teelucksingh S, Sakhamuri S. The impact of air pollution on the incidence and mortality of COVID-19. Glob Health Res Policy. 2020;5:39.
255	Kaya K, Khalil M, Fetrow B, Fritz H, Jagadesan P, Bondu V, et al. Rapid and Effective Inactivation of SARS-CoV-2 with a Cationic Conjugated Oligomer with Visible Light: Studies of Antiviral Activity in Solutions and on Supports. ACS applied materials & interfaces. 2022;14(4):4892-8.
256	Kerboua KE. The perplexing question of trained immunity vs adaptive memory in COVID-19. Journal of Medical Virology. 2020;92(10):1858-63.
257	Khallouli A, Lagneb C, Choura R, Saidane R, Gouider D, Maalej A, et al. Retinal microvascular impairment assessed by OCT-Angiography after SARS-CoV-2 infection. Ophthalmologica Conference: European Society of Retina Specialists Congress, EURETINA. 2021;244(SUPPL 1).
258	Khan T, Withers C, Martin E, Bonilla N. Approaches for effective negative pressure isolation space control to minimize airborne transmission of contaminants in residential homes. Indoor and Built Environment. 2022.
259	Khan TR, Parker DS, Withers C. Mitigation of airborne contaminant spread through simple interventions in an occupied single-family home. International Journal of Environmental Research and Public Health. 2021;18(11) (no pagination).
260	Khan YA. The COVID-19 pandemic and its impact on environment: the case of the major cities in Pakistan. Environmental science and pollution research international. 2021;28(39):54728-43.
261	Khatoon F, Prasad K, Kumar V. COVID-19 associated nervous system manifestations. Sleep Medicine. 2021.

262	Kim D, Ko JH, Peck KR, Baek JY, Moon HW, Ki HK, et al. A covid-19 exposure at a dental clinic where healthcare workers routinely use particulate filtering respirators. International Journal of Environmental Research and Public Health. 2021;18(12) (no pagination).
263	Kim HS. Do an altered gut microbiota and an associated leaky gut affect COVID-19 severity? mBio. 2021;12(1):1-9.
264	Kim JH, Kim J, Kim WJ, Choi YH, Yang SR, Hong SH. Diesel Particulate Matter 2.5 Induces Epithelial-to-Mesenchymal Transition and Upregulation of SARS-CoV-2 Receptor during Human Pluripotent Stem Cell-Derived Alveolar Organoid Development. Int J Environ Res Public Health. 2020;17(22).
265	Kim JH, Kim J, Kim WJ, Choi YH, Yang SR, Hong SH. Diesel particulate matter 2.5 induces epithelial-to-mesenchymal transition and upregulation of sars-cov-2 receptor during human pluripotent stem cell-derived alveolar organoid development. International Journal of Environmental Research and Public Health. 2020;17(22):1-15.
266	Kiser D, Elhanan G, Metcalf WJ, Schnieder B, Grzymski JJ. SARS-CoV-2 test positivity rate in Reno, Nevada: association with PM2.5 during the 2020 wildfire smoke events in the western United States. J Expo Sci Environ Epidemiol. 2021;31(5):797-803.
267	Kma L. Transforming growth factor-beta1 as lung injury biomarker: A review. International Journal of Life Sciences Biotechnology and Pharma Research. 2013;2(3):39-58.
268	Kogevinas M, Castano-Vinyals G, Karachaliou M, Espinosa A, de Cid R, Garcia-Aymerich J, et al. Ambient air pollution in relation to SARS-CoV-2 infection, antibody response, and COVID-19 disease: A cohort study in Catalonia, Spain (COVICAT study). Environmental Health Perspectives. 2021;129(11) (no pagination).
269	Koike H, Chiba A, Katsuno M. Emerging Infection, Vaccination, and Guillain-Barre Syndrome: A Review. Neurology and Therapy. 2021;10(2):523-37.
270	Konwar C, Asiimwe R, Inkster AM, Merrill SM, Negri GL, Aristizabal MJ, et al. Risk-focused differences in molecular processes implicated in SARS-CoV-2 infection: corollaries in DNA methylation and gene expression. Epigenetics and Chromatin. 2021;14(1) (no pagination).
271	Kostoff RN, Briggs MB, Kanduc D, Shores DR, Kovatsi L, Drakoulis N, et al. Contributing factors common to COVID-19 and gastrointestinal cancer. Oncology Reports. 2022;47(1) (no pagination).
272	Krajewska J, Krajewski W, Zub K, Zatonski T. Review of practical recommendations for otolaryngologists and head and neck surgeons during the COVID-19 pandemic. Auris Nasus Larynx. 2020;47(4):544-58.
273	Krawitz BD, Sirinek P, Doobin D, Nanda T, Ghiassi M, Horowitz JD, et al. The Challenge of Managing Bilateral Acute Angle-closure Glaucoma in the Presence of Active SARS-CoV-2 Infection. Journal of Glaucoma. 2021;30(3):e50-e3.
274	Kunz Y, Horninger W, Pinggera GM. Are urologists in trouble with SARS-CoV-2? Reflections and recommendations for specific interventions. BJU International. 2020;126(6):670-8.
275	Kusaba Y, Izumi S, Takasaki J, Suzuki M, Katagiri D, Katsuno T, et al. Successful recovery from COVID-19-associated acute respiratory failure with polymyxin b-immobilized fiber column-direct hemoperfusion. Internal Medicine. 2020;59(19):2405-8.
276	Kusaba Y, Izumi S, Takasaki J, Suzuki M, Katagiri D, Katsuno T, et al. Successful recovery from COVID-19-associated acute respiratory failure with polymyxin B-immobilized fiber column-direct hemoperfusion. Internal Medicine. 2020;59(19):2405-8.
277	Lai A, Chang ML, O'Donnell RP, Zhou C, Sumner JA, Hsiai TK. Association of COVID-19 transmission with high levels of ambient pollutants: Initiation and impact of the inflammatory response on cardiopulmonary disease. Sci Total Environ. 2021;779:146464.
278	Landry SA, Barr JJ, MacDonald MI, Subedi D, Mansfield D, Hamilton GS, et al. Viable virus aerosol propagation by positive airway pressure circuit leak and mitigation with a ventilated patient hood. European Respiratory Journal. 2021;57(6) (no pagination).
279	Lani-Louzada R, do Val Ferreira Ramos C, Cordeiro RM, Sadun AA. Retinal changes in COVID-19 hospitalized cases. PLoS ONE. 2020;15(12 December) (no pagination).
280	Latorre A, Rothwell JC. Myoclonus and COVID-19: A Challenge for the Present, a Lesson for the Future. Movement Disorders Clinical Practice. 2020;7(8):888-90.
281	Laxmipriya S, Narayanan RM. COVID-19 and its relationship to particulate matter pollution - Case study from part of greater Chennai, India. Mater Today Proc. 2021;43:1634-9.
282	Leal-Martinez F, Abarca-Bernal L, Garcia-Perez A, Gonzalez-Tolosa D, Cruz-Cazares G, Montell-Garcia M, et al. Effect of a Nutritional Support System to Increase Survival and Reduce Mortality in Patients with COVID-19 in Stage III and Comorbidities: A Blinded Randomized Controlled Clinical Trial. International Journal of Environmental Research and Public Health. 2022;19(3) (no pagination).
283	Leão MLP, Penteado JO, Ulguim SM, Gabriel RR, Dos Santos M, Brum AN, et al. Health impact assessment of air pollutants during the COVID-19 pandemic in a Brazilian metropolis. Environ Sci Pollut Res Int. 2021;28(31):41843-50.
284	Leao MLP, Penteado JO, Ulguim SM, Gabriel RR, Dos Santos M, Brum AN, et al. Health impact assessment of air pollutants during the COVID-19 pandemic in a Brazilian metropolis. Environmental science and pollution research international. 2021;28(31):41843-50.
285	Lelieveld J, Helleis F, Borrmann S, Cheng Y, Drewnick F, Haug G, et al. Model calculations of aerosol transmission and infection risk of covid-19 in indoor environments. International Journal of Environmental Research and Public Health. 2020;17(21):1-18.
286	Lembo R, Landoni G, Cianfanelli L, Frontera A. Air pollutants and SARS-CoV-2 in 33 European countries. Acta Biomed. 2021;92(1):e2021166.
287	Lembo R, Landoni G, Cianfanelli L, Frontera A. Air pollutants and sars-cov-2 in 33 european countries. Acta Biomedica. 2021;92(1) (no pagination).
288	Leung C. Guillain-Barre syndrome should be monitored upon mass vaccination against SARS-CoV-2. Human Vaccines and Immunotherapeutics. 2021;17(9):2957-8.
289	Li H, Xu XL, Dai DW, Huang ZY, Ma Z, Guan YJ. Air pollution and temperature are associated with increased COVID-19 incidence: A time series study. International Journal of Infectious Diseases. 2020;97:278-82.

290	Li HH, Liu CC, Hsu TW, Lin JH, Hsu JW, Li AF, et al. Upregulation of ACE2 and TMPRSS2 by particulate matter and idiopathic pulmonary fibrosis: a potential role in severe COVID-19. Part Fibre Toxicol. 2021;18(1):11.
291	Li HH, Liu CC, Hsu TW, Lin JH, Hsu JW, Li AFY, et al. Upregulation of ACE2 and TMPRSS2 by particulate matter and idiopathic pulmonary fibrosis: a potential role in severe COVID-19. Particle and Fibre Toxicology. 2021;18(1) (no pagination).
292	Li L, Gu J, Gong E, Li X, Shao H, Jiang H, et al. Biosafety level 3 laboratory for autopsies of patients with severe acute respiratory syndrome: Principles, practices, and prospects. Clinical Infectious Diseases. 2005;41(6):815-21.
293	Li M, Yang Y, He T, Wei R, Qi T, Han T, et al. Detection of SARS-CoV-2 in the ocular surface in different phases of COVID-19 patients in Shanghai, China. Annals of Translational Medicine. 2021;9(2) (no pagination).
294	Li W, Joshi MD, Singhania S, Ramsey KH, Murthy AK. Peptide vaccine: Progress and challenges. Vaccines. 2014;2(3):515-36.
295	Li Y, Wu J, Wang S, Li X, Zhou J, Huang B, et al. Progression to fibrosing diffuse alveolar damage in a series of 30 minimally invasive autopsies with COVID-19 pneumonia in Wuhan, China. Histopathology. 2021;78(4):542-55.
296	Liccardi G, Martini M, Bilo MB, Milanese M, Rogliani P. Use of face masks and allergic nasal symptoms: Why not mention pollen count and air pollution data? American journal of otolaryngology. 2021:103363.
297	Lin S, Wei D, Sun Y, Chen K, Yang L, Liu B, et al. Region-specific air pollutants and meteorological parameters influence COVID-19: A study from mainland China. Ecotoxicol Environ Saf. 2020;204:111035.
298	Lin Z, Shu H, Jiang D, He Y, Xia H, Liu Y, et al. Ward renovation and PPE use procedures to protect medical staff from COVID-19 infection. Journal of Infection in Developing Countries. 2020;14(6):554-8.
299	Lindsley WG, Derk RC, Coyle JP, Martin SB, Mead KR, Blachere FM, et al. Efficacy of Portable Air Cleaners and Masking for Reducing Indoor Exposure to Simulated Exhaled SARS-CoV-2 Aerosols - United States, 2021. Mmwr. 2021;Morbidity and mortality weekly report. 70(27):972-6.
300	Linillos-Pradillo B, Rancan L, Ramiro ED, Vara E, Artíñano B, Arias J. Determination of SARS-CoV-2 RNA in different particulate matter size fractions of outdoor air samples in Madrid during the lockdown. Environ Res. 2021;195:110863.
301	Linillos-Pradillo B, Rancan L, Ramiro ED, Vara E, Artinano B, Arias J. Determination of SARS-CoV-2 RNA in different particulate matter size fractions of outdoor air samples in Madrid during the lockdown. Environmental Research. 2021;195 (no pagination).
302	Linneberg A, Kampmann FB, Israelsen SB, Andersen LR, Jorgensen HL, Sandholt H, et al. The association of low vitamin k status with mortality in a cohort of 138 hospitalized patients with covid-19. Nutrients. 2021;13(6) (no pagination).
303	Liu BM, Yang QQ, Zhao LY, Xie W, Si XY. Epidemiological characteristics of COVID-19 patients in convalescence period. Epidemiology and Infection. 2020.
304	Liu D, Thompson JR, Carducci A, Bi X. Potential secondary transmission of SARS-CoV-2 via wastewater. Science of the Total Environment. 2020;749 (no pagination).
305	Liu DT, Philips KM, Speth MM, Besser G, Mueller CA, Sedaghat AR. Portable HEPA Purifiers to Eliminate Airborne SARS-CoV-2: A Systematic Review. Otolaryngology Head and Neck Surgery. 2021.
306	Liu DT, Phillips KM, Speth M, Besser G, Mueller CA, Sedaghat AR. Portable HEPA purifiers to eliminate airborne SARS-CoV-2: A systematic review. Otolaryngology - Head and Neck Surgery. 2021;165(1 SUPPL):P140.
307	Lopez JH, Romo AS, Molina DC, Hernandez GA, Cureno ABG, Acosta MA, et al. Detection of Sars-Cov-2 in the air of two hospitals in Hermosillo, Sonora, Mexico, utilizing a low-cost environmental monitoring system. International Journal of Infectious Diseases. 2021;102:478-82.
308	López-Feldman A, Heres D, Marquez-Padilla F. Air pollution exposure and COVID-19: A look at mortality in Mexico City using individual-level data. Sci Total Environ. 2021;756:143929.
309	Lopez-Feldman A, Heres D, Marquez-Padilla F. Air pollution exposure and COVID-19: A look at mortality in Mexico City using individual-level data. Science of the Total Environment. 2021;756 (no pagination).
310	Losilla-Rodriguez B, Maldonado N, Moreno-Mellado E, Lopez-Diaz A. COVID-19 natural herd immunity and risk of neuropsychiatric disorders. Revista de Psiquiatria y Salud Mental. 2020;13(4):228-9.
311	Lu B, Wu N, Jiang J, Li X. Associations of acute exposure to airborne pollutants with COVID-19 infection: evidence from China. Environ Sci Pollut Res Int. 2021;28(36):50554-64.
312	Lubrano C, Risi R, Masi D, Gnessi L, Colao A. Is obesity the missing link between COVID-19 severity and air pollution? Environmental Pollution. 2020;Part 3. 266 (no pagination).
313	Lym Y, Kim KJ. Exploring the effects of PM<inf>2.5</inf> and temperature on COVID-19 transmission in Seoul, South Korea. Environmental Research. 2022;203 (no pagination).
314	Lym Y, Kim KJ. Exploring the effects of PM(2.5) and temperature on COVID-19 transmission in Seoul, South Korea. Environ Res. 2022;203:111810.
315	Macias-Verde D, Lara PC, Burgos-Burgos J. Same pollution sources for climate change might be hyperactivating the NLRP3 inflammasome and exacerbating neuroinflammation and SARS mortality. Med Hypotheses. 2021;146:110396.
316	Maheswari S, Pethannan R, Sabarimurugan S. Air pollution enhances susceptibility to novel coronavirus (COVID-19) infection - An impact study. Environmental Health and Toxicology. 2020;35(4):1-7.
317	Makovitzki A, Lerer E, Kafri Y, Adar Y, Cherry L, Lupu E, et al. Evaluation of a downstream process for the recovery and concentration of a Cell-Culture-Derived rVSV-Spike COVID-19 vaccine candidate. Vaccine. 2021;39(48):7044-51.
318	Maleki M, Anvari E, Hopke PK, Noorimotlagh Z, Mirzaee SA. An updated systematic review on the association between atmospheric particulate matter pollution and prevalence of SARS-CoV-2. Environ Res. 2021;195:110898.
319	Manivannan J, Sundaresan L. Systems level insights into the impact of airborne exposure on SARS-CoV-2 pathogenesis and COVID-19 outcome - A multi-omics big data study. Gene Reports. 2021;25 (no pagination).
320	Marcos-Garcia P, Carmona-Moreno C, Lopez-Puga J, Ruiz-Ruano Garcia AM. COVID-19 pandemic in Africa: Is it time for water, sanitation and hygiene to climb up the ladder of global priorities? Science of the Total Environment. 2021;791 (no pagination).
321	Marquès M, Correig E, Ibarretxe D, Anoro E, Antonio Arroyo J, Jericó C, et al. Long-term exposure to PM(10) above WHO guidelines exacerbates COVID-19 severity and mortality. Environ Int. 2022;158:106930.

322	Marques M, Correig E, Ibarretxe D, Anoro E, Antonio Arroyo J, Jerico C, et al. Long-term exposure to PM<inf>10</inf> above WHO guidelines exacerbates COVID-19 severity and mortality. Environment International. 2022;158 (no pagination).
323	Marques M, Domingo JL. Positive association between outdoor air pollution and the incidence and severity of COVID-19. A review of the recent scientific evidences. Environmental Research. 2022;203 (no pagination).
324	Marques M, Rovira J, Nadal M, Domingo JL. Effects of air pollution on the potential transmission and mortality of COVID-19: A preliminary case-study in Tarragona Province (Catalonia, Spain). Environmental Research. 2021;192 (no pagination).
325	Martin-Quintero I, Cervera-Sabater A, Tapias-Perero V, Nieto-Sanchez I, de la Cruz-Perez J. Air particulate concentration during orthodontic procedures: a pilot study. BMC oral health. 2021;21(1):361.
326	Martorell-Marugán J, Villatoro-García JA, García-Moreno A, López-Domínguez R, Requena F, Merelo JJ, et al. DatAC: A visual analytics platform to explore climate and air quality indicators associated with the COVID-19 pandemic in Spain. Sci Total Environ. 2021;750:141424.
327	Martorell-Marugan J, Villatoro-Garcia JA, Garcia-Moreno A, Lopez-Dominguez R, Requena F, Merelo JJ, et al. DatAC: A visual analytics platform to explore climate and air quality indicators associated with the COVID-19 pandemic in Spain. Science of the Total Environment. 2021;750 (no pagination).
328	Marwah M, Agrawala PK. COVID-19 lockdown and environmental pollution: an Indian multi-state investigation. Environmental Monitoring and Assessment. 2022;194(2) (no pagination).
329	Mehmel M, Jovanovic N, Spitz U. Nicotinamide riboside-the current state of research and therapeutic uses. Nutrients. 2020;12(6) (no pagination).
330	Mehmood K, Bao Y, Petropoulos GP, Abbas R, Abrar MM, Saifullah, et al. Investigating connections between COVID-19 pandemic, air pollution and community interventions for Pakistan employing geoinformation technologies. Chemosphere. 2021;272 (no pagination).
331	Menchaca M, Pagone F, Erdal S. Comparison of positive SARS-CoV-2 incidence rate with environmental and socioeconomic factors in northern Illinois. Heliyon. 2021;7(8):e07806.
332	Menculini G, Bernardini F, Attademo L, Balducci PM, Sciarma T, Moretti P, et al. The influence of the urban environment on mental health during the covid-19 pandemic: Focus on air pollution and migration-a narrative review. International Journal of Environmental Research and Public Health. 2021;18(8) (no pagination).
333	Mendez-Espinosa JF, Rojas NY, Vargas J, Pachon JE, Belalcazar LC, Ramirez O. Air quality variations in Northern South America during the COVID-19 lockdown. Science of the Total Environment. 2020;749 (no pagination).
334	Mendoza DL, Benney TM, Bares R, Crosman ET. Intra-city variability of fine particulate matter during COVID-19 lockdown: A case study from Park City, Utah. Environmental Research. 2021;201 (no pagination).
335	Mendy A, Wu X, Keller JL, Fassler CS, Apewokin S, Mersha TB, et al. Long-term exposure to fine particulate matter and hospitalization in COVID-19 patients. Respir Med. 2021;178:106313.
336	Mendy A, Wu X, Keller JL, Fassler CS, Apewokin S, Mersha TB, et al. Air pollution and the pandemic: Long-term PM(2.5) exposure and disease severity in COVID-19 patients. Respirology. 2021;26(12):1181-7.
337	Mendy A, Wu X, Keller JL, Fassler CS, Apewokin S, Mersha TB, et al. Air pollution and the pandemic: Long-term PM<inf>2.5</inf> exposure and disease severity in COVID-19 patients. Respirology. 2021;26(12):1181-7.
338	Mendy A, Wu X, Keller JL, Fassler CS, Apewokin S, Mersha TB, et al. Long-term exposure to fine particulate matter and hospitalization in COVID-19 patients. Respiratory Medicine. 2021;178 (no pagination).
339	Menendez JA. Metformin and SARS-CoV-2: mechanistic lessons on air pollution to weather the cytokine/thrombotic storm in COVID-19. Aging. 2020;12(10):8760-5.
340	Meo SA, Abukhalaf AA, Alessa OM, Alarifi AS, Sami W, Klonoff DC. Effect of Environmental Pollutants PM2.5, CO, NO(2), and O(3) on the Incidence and Mortality of SARS-CoV-2 Infection in Five Regions of the USA. Int J Environ Res Public Health. 2021;18(15).
341	Meo SA, Abukhalaf AA, Alomar AA, Alessa OM. Wildfire and COVID-19 pandemic: Effect of environmental pollution PM-2.5 and carbon monoxide on the dynamics of daily cases and deaths due to SARS-COV-2 infection in San-Francisco USA. European Review for Medical and Pharmacological Sciences. 2020;24(19):10286-92.
342	Meo SA, Abukhalaf AA, Alomar AA, Alessa OM, Sami W, Klonoff DC. Effect of environmental pollutants PM-2.5, carbon monoxide, and ozone on the incidence and mortality of SARS-COV-2 infection in ten wildfire affected counties in California. Sci Total Environ. 2021;757:143948.
343	Meo SA, Abukhalaf AA, Alomar AA, Alessa OM, Sami W, Klonoff DC. Effect of environmental pollutants PM-2.5, carbon monoxide, and ozone on the incidence and mortality of SARS-COV-2 infection in ten wildfire affected counties in California. Science of the Total Environment. 2021;757 (no pagination).
344	Meo SA, Ahmed Alqahtani S, Saad Binmeather F, Abdulrhman AlRasheed R, Mohammed Aljedaie G, Mohammed Albarrak R. Effect of environmental pollutants PM2.5, CO, O(3) and NO(2), on the incidence and mortality of SARS-COV-2 in largest metropolitan cities, Delhi, Mumbai and Kolkata, India. J King Saud Univ Sci. 2022;34(1):101687.
345	Meo SA, Al-Khlaiwi T, Ullah CH. Effect of ambient air pollutants PM2.5 and PM10 on COVID-19 incidence and mortality: observational study. Eur Rev Med Pharmacol Sci. 2021;25(23):7553-64.
346	Meo SA, Almutairi FJ, Abukhalaf AA, Alessa OM, Al-Khlaiwi T, Meo AS. Sandstorm and its effect on particulate matter PM 2.5, carbon monoxide, nitrogen dioxide, ozone pollutants and SARS-CoV-2 cases and deaths. Sci Total Environ. 2021;795:148764.
347	Meo SA, Almutairi FJ, Abukhalaf AA, Alessa OM, Al-Khlaiwi T, Meo AS. Sandstorm and its effect on particulate matter PM 2.5, carbon monoxide, nitrogen dioxide, ozone pollutants and SARS-CoV-2 cases and deaths. Science of the Total Environment. 2021;795 (no pagination).
348	Meo SA, Almutairi FJ, Abukhalaf AA, Usmani AM. Effect of Green Space Environment on Air Pollutants PM2.5, PM10, CO, O(3), and Incidence and Mortality of SARS-CoV-2 in Highly Green and Less-Green Countries. Int J Environ Res Public Health. 2021;18(24).

349	Meo SA, Almutairi FJ, Abukhalaf AA, Usmani AM. Effect of green space environment on air pollutants PM2.5, PM10, CO, O<inf>3</inf>, and incidence and mortality of SARS-CoV-2 in highly green and less-green countries. International Journal of Environmental Research and Public Health. 2021;18(24) (no pagination).
350	Mescoli A, Maffei G, Pillo G, Bortone G, Marchesi S, Morandi E, et al. The Secretive Liaison of Particulate Matter and SARS-CoV-2. A Hypothesis and Theory Investigation. Front Genet. 2020;11:579964.
351	Mescoli A, Maffei G, Pillo G, Bortone G, Marchesi S, Morandi E, et al. The Secretive Liaison of Particulate Matter and SARS-CoV-2. A Hypothesis and Theory Investigation. Frontiers in Genetics. 2020;11 (no pagination).
352	Milone I, Vento R, Ippolito L, Paroni S, Vento MG. Therapeutic support protocol for patient with dysosmia with or without dysgeusia related to the SARS-CoV2 virus infection. Acta Biomedica. 2020;91(4):1-2.
353	Mimura T, Noma H, Matsumoto K, Kawashima M, Kitsu K, Itoh E, et al. Concentration of droplets from patients during normal breathing and speech and their importance in protection from coronavirus sars-cov-2 (Covid-19) infection. Open Ophthalmology Journal. 2021;15(1):109-7.
354	Min YG, Ju W, Ha YE, Ban JJ, Lee SA, Sung JJ, et al. Sensory Guillain-Barre syndrome following the ChAdOx1 nCov-19 vaccine: Report of two cases and review of literature. Journal of Neuroimmunology. 2021;359 (no pagination).
355	Miyashita L, Foley G, Grigg J. Exposure to Particulate Matter Increases Expression of the Angiotensin converting enzyme-2 (ACE2) Receptor. Journal of Allergy and Clinical Immunology. 2022;149(2 Supplement):AB30.
356	Moccia F, Gerbino A, Lionetti V, Miragoli M, Munaron LM, Pagliaro P, et al. COVID-19-associated cardiovascular morbidity in older adults: a position paper from the Italian Society of Cardiovascular Researches. GeroScience. 2020;42(4):1021-49.
357	Moelling K, Broecker F. Air Microbiome and Pollution: Composition and Potential Effects on Human Health, Including SARS Coronavirus Infection. J Environ Public Health. 2020;2020:1646943.
358	Moreno-Duarte I, Evans AS, Alder AC, Vernon MC, Szmuk P, Rebstock S. An unexpected COVID-19 diagnosis during emergency surgery in a neonate. Paediatric Anaesthesia. 2021;31(5):613-5.
359	Moschetta L, Fasolino G, Kuijpers RW. Non-arteritic anterior ischaemic optic neuropathy sequential to SARS-CoV-2 virus pneumonia: Preventable by endothelial protection? BMJ Case Reports. 2021;14(7) (no pagination).
360	Moshammer H, Poteser M, Hutter HP. COVID-19 and air pollution in Vienna-a time series approach. Wiener Klinische Wochenschrift. 2021;133(17-18):951-7.
361	Mukherjee S, Boral S, Siddiqi H, Mishra A, Meikap BC. Present cum future of SARS-CoV-2 virus and its associated control of virus-laden air pollutants leading to potential environmental threat - A global review. J Environ Chem Eng. 2021;9(2):104973.
362	Mulder C, Conti E, Saccone S, Federico C. Beyond virology: environmental constraints of the first wave of COVID-19 cases in Italy. Environ Sci Pollut Res Int. 2021;28(24):31996-2004.
363	Mulder C, Conti E, Saccone S, Federico C. Beyond virology: environmental constraints of the first wave of COVID-19 cases in Italy. Environmental science and pollution research international. 2021;28(24):31996-2004.
364	Nagu P, Parashar A, Behl T, Mehta V. CNS implications of COVID-19: A comprehensive review. Reviews in the Neurosciences. 2021;32(2):219-34.
365	Nahama A, Ramachandran R, Cisternas AF, Ji H. The role of afferent pulmonary innervation in ARDS associated with COVID-19 and potential use of resiniferatoxin to improve prognosis: A review. Medicine in Drug Discovery. 2020;5 (no pagination).
366	Naidoo P, Ghazi T, Chuturgoon AA, Naidoo RN, Ramsuran V, Mpaka-Mbatha MN, et al. SARS-CoV-2 and helminth co-infections, and environmental pollution exposure: An epidemiological and immunological perspective. Environment International. 2021;156 (no pagination).
367	Navarro KM, Clark KA, Hardt DJ, Reid CE, Lahm PW, Domitrovich JW, et al. Wildland firefighter exposure to smoke and COVID-19: A new risk on the fire line. Sci Total Environ. 2021;760:144296.
368	Ng WF, To KF, Lam WWL, Ng TK, Lee KC. The comparative pathology of severe acute respiratory syndrome and avian influenza A subtype H5N1 - A review. Human Pathology. 2006;37(4):381-90.
369	Nguyen TPM, Bui TH, Nguyen MK, Nguyen TH, Vu VT, Pham HL. Impact of Covid-19 partial lockdown on PM<inf>2.5</inf>, SO<inf>2</inf>, NO<inf>2</inf>, O<inf>3</inf>, and trace elements in PM<inf>2.5</inf> in Hanoi, Vietnam. Environmental science and pollution research international. 2021;08.
370	Niccoli G, Luescher TF, Crea F. Decreasedmyocardial infarction admissions during covid times: What can we learn? Cardiovascular Research. 2020;116(10):E126-E8.
371	Nihei Y, Nagasawa H, Fukao Y, Kihara M, Ueda S, Gohda T, et al. Continuous extracorporeal treatments in a dialysis patient with COVID-19. CEN Case Reports. 2021;10(2):172-7.
372	Nor NSM, Yip CW, Ibrahim N, Jaafar MH, Rashid ZZ, Mustafa N, et al. Particulate matter (PM(2.5)) as a potential SARS-CoV-2 carrier. Sci Rep. 2021;11(1):2508.
373	Nor NSM, Yip CW, Ibrahim N, Jaafar MH, Rashid ZZ, Mustafa N, et al. Particulate matter (PM<inf>2.5</inf>) as a potential SARS-CoV-2 carrier. Scientific reports. 2021;11(1):2508.
374	Norouzi N, Asadi Z. Air pollution impact on the Covid-19 mortality in Iran considering the comorbidity (obesity, diabetes, and hypertension) correlations. Environ Res. 2022;204(Pt A):112020.
375	Nozza E, Valentini S, Melzi G, Vecchi R, Corsini E. Advances on the immunotoxicity of outdoor particulate matter: A focus on physical and chemical properties and respiratory defence mechanisms. Science of the Total Environment. 2021;780 (no pagination).
376	Nunez-Delgado A, Bontempi E, Coccia M, Kumar M, Farkas K, Domingo JL. SARS-CoV-2 and other pathogenic microorganisms in the environment. Environmental Research. 2021;201 (no pagination).
377	Oaklander AL. Clinical significance of angiotensin-converting enzyme 2 receptors for severe acute respiratory syndrome coronavirus 2 (COVID-19) on peripheral small-fiber sensory neurons is unknown today. Pain. 2020;161(11):2431-3.
378	Orak NH, Ozdemir O. The impacts of COVID-19 lockdown on PM<inf>10</inf> and SO<inf>2</inf> concentrations and association with human mobility across Turkey. Environmental Research. 2021;197 (no pagination).

379	Oren B, Aksoy Aydemir G, Aydemir E, Atesoglu HI, Goker YS, Kiziltoprak H, et al. Quantitative assessment of retinal changes in COVID-19 patients. Clinical & experimental optometry. 2021;104(6):717-22.
380	Owen L, Shivkumar M, Laird K. The Stability of Model Human Coronaviruses on Textiles in the Environment and during Health Care Laundering. mSphere. 2021;6(2):1-15.
381	Ozbas M, Demirayak B, Vural A, Karabela Y, Yigit FU. Investigation of Retinal Alterations in Patients Recovered from COVID-19: A Comparative Study. Ocular Immunology and Inflammation. 2022.
382	Páez-Osuna F, Valencia-Castañeda G, Rebolledo UA. The link between COVID-19 mortality and PM(2.5) emissions in rural and medium-size municipalities considering population density, dust events, and wind speed. Chemosphere. 2022;286(Pt 1):131634.
383	Paez-Osuna F, Valencia-Castaneda G, Rebolledo UA. The link between COVID-19 mortality and PM<inf>2.5</inf> emissions in rural and medium-size municipalities considering population density, dust events, and wind speed. Chemosphere. 2022;Part 1. 286 (no pagination).
384	Pajo AT, Espiritu AI, Apor ADAO, Jamora RDG. Neuropathologic findings of patients with COVID-19: a systematic review. Neurological Sciences. 2021;42(4):1255-66.
385	Palmeri V, Le Turdu-Chicot C, Garin B, Auguste A, Roger PM. Seroprevalence of SARS-CoV-2 IgG antibodies among workers in the University Hospital of Guadeloupe. Clinica Terapeutica. 2021;172(5):461-6.
386	Pandey AS, Ringer AJ, Rai AT, Kan P, Jabbour P, Siddiqui AH, et al. Minimizing SARS-CoV-2 exposure when performing surgical interventions during the COVID-19 pandemic. Journal of NeuroInterventional Surgery. 2020;12(7):643-7.
387	Peddinti BST, Morales-Gagnon N, Pourdeyhimi B, Scholle F, Spontak RJ, Ghiladi RA. Photodynamic Coatings on Polymer Microfibers for Pathogen Inactivation: Effects of Application Method and Composition. ACS applied materials & interfaces. 2021;13(1):155-63.
388	Pednekar P, Amoah K, homer R, Ryu C, Lutchmansingh D. Bullous Lung Disease Following Covid-19 Infection. Chest. 2021;160(4 Supplement):A356.
389	Peng S, Cao F, Xia Y, Gao XD, Dai L, Yan J, et al. Particulate Alum via Pickering Emulsion for an Enhanced COVID-19 Vaccine Adjuvant. Advanced materials (Deerfield Beach, Fla). 2020;32(40):e2004210.
390	Pereira A. Long-Term Neurological Threats of COVID-19: A Call to Update the Thinking About the Outcomes of the Coronavirus Pandemic. Frontiers in Neurology. 2020;11 (no pagination).
391	Perez J, Santillana C, Corrales O, Gonzalez D, Lacaille S, Sitahal R. An Unusual Variant of Guillain- Barre Syndrome with Covid-19. Chest. 2021;160(4 Supplement):A2344.
392	Perret J, Dharmage S. COVID-19 hospitalizations: Another adverse impact of ambient air pollution? Respirology. 2021;26(12):1101-2.
393	Perret J, Dharmage S. COVID-19 hospitalizations: Another adverse impact of ambient air pollution? Respirology. 2021;26(12):1101-2.
394	Phu HT, Park Y, Andrews AJ, Marabella I, Abraham A, Mimmack R, et al. Design and evaluation of a portable negative pressure hood with HEPA filtration to protect health care workers treating patients with transmissible respiratory infections. American Journal of Infection Control. 2020;48(10):1237-43.
395	Pivato A, Amoruso I, Formenton G, Di Maria F, Bonato T, Vanin S, et al. Evaluating the presence of SARS-CoV-2 RNA in the particulate matters during the peak of COVID-19 in Padua, northern Italy. Science of the Total Environment. 2021;784 (no pagination).
396	Pons MN, Louis P, Vignati D. Effect of lockdown on wastewater characteristics: A comparison of two large urban areas. Water Science and Technology. 2020;82(12):2813-22.
397	Popov T, Josling P, DuBuske L. Regular Use of Nasally Applied Methyl-cellulose Powder During the Pollen Season Protects Against Sars-Covid-2 Infections. Journal of Allergy and Clinical Immunology. 2022;149(2 Supplement):AB100.
398	Poyraz BM, Engin ED, Engin AB, Engin A. The effect of environmental diesel exhaust pollution on SARS-CoV-2 infection: The mechanism of pulmonary ground glass opacity. Environ Toxicol Pharmacol. 2021;86:103657.
399	Poyraz BM, Engin ED, Engin AB, Engin A. The effect of environmental diesel exhaust pollution on SARS-CoV-2 infection: The mechanism of pulmonary ground glass opacity. Environmental Toxicology and Pharmacology. 2021;86 (no pagination).
400	Pozzer A, Dominici F, Haines A, Witt C, Münzel T, Lelieveld J. Regional and global contributions of air pollution to risk of death from COVID-19. Cardiovasc Res. 2020;116(14):2247-53.
401	Przekwas A, Chen Z. Washing hands and the face may reduce COVID-19 infection. Medical Hypotheses. 2020;144 (no pagination).
402	Qu JH, Leirs K, Maes W, Imbrechts M, Callewaert N, Lagrou K, et al. Innovative FO-SPR Label-free Strategy for Detecting Anti-RBD Antibodies in COVID-19 Patient Serum and Whole Blood. ACS sensors. 2022;21.
403	Querol X, Massague J, Alastuey A, Moreno T, Gangoiti G, Mantilla E, et al. Lessons from the COVID-19 air pollution decrease in Spain: Now what? Science of the Total Environment. 2021;779 (no pagination).
404	Rabaan AA, Al-Ahmed SH, Al-Malkey MK, Alsubki RA, Ezzikouri S, Al-Hababi FH, et al. Airborne transmission of SARS-CoV-2 is the dominant route of transmission: Droplets and aerosols. Infezioni in Medicina. 2021;29(1):10-9.
405	Raboud J, Shigayeva A, McGeer A, Bontovics E, Chapman M, Gravel D, et al. Risk factors for SARS transmission from patients requiring intubation: A multicentre investigation in Toronto, Canada. PLoS ONE. 2010;5(5) (no pagination).
406	Racine-Brzostek SE, Yang HS, Jack GA, Chen Z, Chadburn A, Ketas TJ, et al. Postconvalescent sars-cov-2 igg and neutralizing antibodies are elevated in individuals with poor metabolic health. Journal of Clinical Endocrinology and Metabolism. 2021;106(5):E2025-E34.
407	Raciti L, Calabro RS. Can volcanic trace elements facilitate Covid-19 diffusion? A hypothesis stemming from the Mount Etna area, Sicily. Medical Hypotheses. 2020;144 (no pagination).
408	Rajajee V, Williamson CA. Use of a Novel Negative-Pressure Tent During Bedside Tracheostomy in COVID-19 Patients. Neurocritical Care. 2020;33(2):597-603.

409	Rathnasinghe R, Karlicek RF, Schotsaert M, Koffas M, Arduini BL, Jangra S, et al. Scalable, effective, and rapid decontamination of SARS-CoV-2 contaminated N95 respirators using germicidal ultraviolet C (UVC) irradiation device. Scientific reports. 2021;11(1):19970.
410	Rathore DS, Nagda C, Shaktawat BS, Kain T, Chouhan CS, Purohit R, et al. COVID-19 lockdown: a boon in boosting the air quality of major Indian Metropolitan Cities. Aerobiologia. 2021;37(1):79-103.
411	Recchioni A, Makanvand M, Wallace GR, Poonit N, Bloss W, Rauz S. Impact of the United Kingdom (UK) COVID-19 lockdown measures on air pollution and ocular surface disease symptomatology amongst shielding patients. Investigative Ophthalmology and Visual Science Conference: Annual Meeting Association for Research in Vision and Ophthalmology, ARVO. 2021;62(8).
412	Reis NM, Needs SH, Jegouic SM, Gill KK, Sirivisoot S, Howard S, et al. Gravity-Driven Microfluidic Siphons: Fluidic Characterization and Application to Quantitative Immunoassays. ACS sensors. 2021;6(12):4338-48.
413	Resmi CT, Nishanth T, Satheesh Kumar MK, Manoj MG, Balachandramohan M, Valsaraj KT. Air quality improvement during triple-lockdown in the coastal city of Kannur, Kerala to combat Covid-19 transmission. PeerJ. 2020;8 (no pagination).
414	Rexhepi I, Mangifesta R, Santilli M, Guri S, Di Carlo P, D'Addazio G, et al. Effects of natural ventilation and saliva standard ejectors during the covid-19 pandemic: A quantitative analysis of aerosol produced during dental procedures. International Journal of Environmental Research and Public Health. 2021;18(14) (no pagination).
415	Reyes J, Stiehl B, Delgado J, Kinzel M, Ahmed K. Human Research Study of Particulate Propagation Distance from Human Respiratory Function. The Journal of infectious diseases. 2022;12.
416	Reyes MSS, Medina PMB. Environmental pollutant exposure can exacerbate COVID-19 neurologic symptoms. Med Hypotheses. 2020;144:110136.
417	Reyes MSS, Medina PMB. Environmental pollutant exposure can exacerbate COVID-19 neurologic symptoms. Medical Hypotheses. 2020;144 (no pagination).
418	Ricco M, Ranzieri S, Balzarini F, Bragazzi NL, Corradi M. SARS-CoV-2 infection and air pollutants: Correlation or causation? Science of the Total Environment. 2020;734 (no pagination).
419	Rishi P, Thakur K, Vij S, Rishi L, Singh A, Kaur IP, et al. Diet, Gut Microbiota and COVID-19. Indian Journal of Microbiology. 2020;60(4):420-9.
420	Rivera-Rios JC, Joo T, Takeuchi M, Orlando TM, Bevington T, Mathis JW, et al. In-flight particulate matter concentrations in commercial flights are likely lower than other indoor environments. Indoor air. 2021;31(5):1484-94.
421	Rizzo E, Maggiotto G. Correlation between atmospheric particulate matter and antibiotic resistance: A hypothesis. Medical Hypotheses. 2020;141 (no pagination).
422	Roa Gomez G, Diaz-Fuentes G, Venkatram S. Fiber-Optic Bronchoscopy in Patients Infected with Covid-19: A Case Series. Chest. 2020;158(4 Supplement):A1943.
423	Robotto A, Civra A, Quaglino P, Polato D, Brizio E, Lembo D. SARS-CoV-2 airborne transmission: A validated sampling and analytical method. Environmental Research. 2021;200 (no pagination).
424	Rocha CA, Marques EV, Dos Santos RP, de Santiago IS, Cavalcante CLA, Cassiano DR, et al. A better understanding of air quality resulting from the effects of the 2020 pandemic in a city in the equatorial region (Fortaleza, Brazil). Environmental science and pollution research international. 2021;08.
425	Rodriguez M, Palop ML, Sesena S, Rodriguez A. Are the Portable Air Cleaners (PAC) really effective to terminate airborne SARS-CoV-2? Science of the Total Environment. 2021;785 (no pagination).
426	Rubas NC, Maunakea A. Medical School Hotline: Immunoepigenetic-Microbiome Axis: Implications for Health Disparities Research in Native Hawaiians and Pacific Islanders. Hawai'i journal of health & social welfare. 2021;80(8):195-8.
427	Rugani B, Conticini E, Frediani B, Caro D. Decrease in life expectancy due to COVID-19 disease not offset by reduced environmental impacts associated with lockdowns in Italy. Environmental Pollution. 2022;Part A. 292 (no pagination).
428	Sabahgoulian CB, Manvelyan HM. Clinical observation of rare neurological complications of covid-19: Acute demyelinating polyneuropathy and critical illness neuropathy. New Armenian Medical Journal. 2021;15(3):16-21.
429	Sadeghmousavi S, Rezaei N. COVID-19 and Multiple Sclerosis: Predisposition and Precautions in Treatment. SN Comprehensive Clinical Medicine. 2020;2(10):1802-7.
430	Safdar N, Crnich CJ, Maki DG. The pathogenesis of ventilator-associated pneumonia: its relevance to developing effective strategies for prevention. Respiratory care. 2005;50(6):725-39; discussion 39-41.
431	Safdar N, Crnich CJ, Maki DG. The pathogenesis of ventilator-associated pneumonia: Its relevance to developing effective strategies for prevention. Respiratory Care. 2005;50(6):725-39.
432	Sagawa T, Tsujikawa T, Honda A, Miyasaka N, Tanaka M, Kida T, et al. Exposure to particulate matter upregulates ACE2 and TMPRSS2 expression in the murine lung. Environ Res. 2021;195:110722.
433	Sahih M, Schultz A, Wilson A, Alakeson R, Taylor E, Mullins B, et al. Paediatric headbox as aerosol and droplet barrier. Archives of Disease in Childhood. 2022;107(1):65-7.
434	Sahoo MM. Significance between air pollutants, meteorological factors, and COVID-19 infections: probable evidences in India. Environ Sci Pollut Res Int. 2021;28(30):40474-95.
435	Sajdel-Sulkowska EM. Neuropsychiatric Ramifications of COVID-19: Short-Chain Fatty Acid Deficiency and Disturbance of Microbiota-Gut-Brain Axis Signaling. BioMed Research International. 2021;2021 (no pagination).
436	Sakamoto K, Ozaki T, Kadomatsu K. Axonal Regeneration by Glycosaminoglycan. Frontiers in Cell and Developmental Biology. 2021;9 (no pagination).
437	Salazar A, Gonzalez A, Murray NP, Castro C. Atypical presentation of COVID-19: Chronic bilateral testicular pain with lower extremity peripheral polyneuropathy, case report. Urology Case Reports. 2022;40 (no pagination).
438	Saleh Y, Antherieu S, Dusautoir R, L YA, Sotty J, De Sousa C, et al. Exposure to Atmospheric Ultrafine Particles Induces Severe Lung Inflammatory Response and Tissue Remodeling in Mice. Int J Environ Res Public Health. 2019;16(7).

439	Samet JM, Burke TA, Lakdawala SS, Lowe JJ, Marr LC, Prather KA, et al. SARS-CoV-2 indoor air transmission is a threat that can be addressed with science. Proceedings of the National Academy of Sciences of the United States of America. 2021;118(45) (no pagination).
440	Sami S, Horter L, Valencia D, Thomas I, Pomeroy M, Walker B, et al. Investigation of SARS-CoV-2 Transmission Associated With a Large Indoor Convention - New York City, November-December 2021. Mmwr. 2022;Morbidity and mortality weekly report. 71(7):243-8.
441	Samillan VJ, Flores-León D, Rojas E, Zutta BR. Environmental and climatic impact on the infection and mortality of SARS-CoV-2 in Peru. J Basic Clin Physiol Pharmacol. 2021;32(5):935-42.
442	Samillan VJ, Flores-Leon D, Rojas E, Zutta BR. Environmental and climatic impact on the infection and mortality of SARS-CoV-2 in Peru. Journal of Basic and Clinical Physiology and Pharmacology. 2021;32(5):935-42.
443	Sangkham S, Thongtip S, Vongruang P. Influence of air pollution and meteorological factors on the spread of COVID-19 in the Bangkok Metropolitan Region and air quality during the outbreak. Environ Res. 2021;197:111104.
444	Sangkham S, Thongtip S, Vongruang P. Influence of air pollution and meteorological factors on the spread of COVID-19 in the Bangkok Metropolitan Region and air quality during the outbreak. Environmental Research. 2021;197 (no pagination).
445	Sanjay S, Agrawal S, Jayadev C, Kawali A, Gowda PB, Shetty R, et al. Posterior segment manifestations and imaging features post-COVID-19. Medical Hypothesis, Discovery, and Innovation in Ophthalmology. 2021;10(3):95-106.
446	SanJuan-Reyes S, Gomez-Olivan LM, Islas-Flores H. COVID-19 in the environment. Chemosphere. 2021;263 (no pagination).
447	Santurtún A, Colom ML, Fdez-Arroyabe P, Real Á D, Fernández-Olmo I, Zarrabeitia MT. Exposure to particulate matter: Direct and indirect role in the COVID-19 pandemic. Environ Res. 2022;206:112261.
448	Sarapultseva M, Hu D, Sarapultsev A. SARS-CoV-2 Seropositivity among Dental Staff and the Role of Aspirating Systems. JDR clinical and translational research. 2021;6(2):132-8.
449	Savastano A, Crincoli E, Savastano MC, Younis S, Gambini G, De Vico U, et al. Peripapillary retinal vascular involvement in early post-covid-19 patients. Journal of Clinical Medicine. 2020;9(9):1-16.
450	Saw LH, Leo BF, Nor NSM, Yip CW, Ibrahim N, Hamid HHA, et al. Modeling aerosol transmission of SARS-CoV-2 from human-exhaled particles in a hospital ward. Environmental science and pollution research international. 2021;28(38):53478-92.
451	Scalsky RJ, Chen YJ, Ying Z, Perry JA, Hong CC. The Social and Natural Environment's Impact on SARS-CoV-2 Infections in the UK Biobank. Int J Environ Res Public Health. 2022;19(1).
452	Sciomer S, Moscucci F, Magrì D, Badagliacca R, Piccirillo G, Agostoni P. SARS-CoV-2 spread in Northern Italy: what about the pollution role? Environ Monit Assess. 2020;192(6):325.
453	Sciomer S, Moscucci F, Magri D, Badagliacca R, Piccirillo G, Agostoni P. SARS-CoV-2 spread in Northern Italy: what about the pollution role? Environmental Monitoring and Assessment. 2020;192(6) (no pagination).
454	Scuto M, Trovato Salinaro A, Caligiuri I, Ontario ML, Greco V, Sciuto N, et al. Redox modulation of vitagenes via plant polyphenols and vitamin D: Novel insights for chemoprevention and therapeutic interventions based on organoid technology. Mechanisms of Ageing and Development. 2021;199 (no pagination).
455	Seto WH, Conly J, Cookson B, Pittet D, Holmes A, Chu M, et al. Use of medical face masks versus particulate respirators as a component of personal protective equipment for health care workers in the context of the COVID-19 pandemic. Antimicrobial Resistance and Infection Control. 2020;9(1) (no pagination).
456	Setti L, Passarini F, De Gennaro G, Barbieri P, Pallavicini A, Ruscio M, et al. Searching for SARS-COV-2 on Particulate Matter: A Possible Early Indicator of COVID-19 Epidemic Recurrence. Int J Environ Res Public Health. 2020;17(9).
457	Setti L, Passarini F, De Gennaro G, Barbieri P, Pallavicini A, Ruscio M, et al. Searching for SARS-COV-2 on particulate matter: A possible early indicator of COVID-19 epidemic recurrence. International Journal of Environmental Research and Public Health. 2020;17(9) (no pagination).
458	Setti L, Passarini F, De Gennaro G, Barbieri P, Perrone MG, Borelli M, et al. Airborne transmission route of covid-19: Why 2 meters/6 feet of inter-personal distance could not be enough. International Journal of Environmental Research and Public Health. 2020;17(8) (no pagination).
459	Setti L, Passarini F, De Gennaro G, Barbieri P, Perrone MG, Borelli M, et al. SARS-Cov-2RNA found on particulate matter of Bergamo in Northern Italy: First evidence. Environ Res. 2020;188:109754.
460	Shah SM, Alsaab HO, Rawas-Qalaji MM, Uddin MN. A review on current covid-19 vaccines and evaluation of particulate vaccine delivery systems. Vaccines. 2021;9(10) (no pagination).
461	Shahdad S, Patel T, Hindocha A, Cagney N, Mueller JD, Seoudi N, et al. The efficacy of an extraoral scavenging device on reduction of splatter contamination during dental aerosol generating procedures: an exploratory study. British dental journal. 2020;11.
462	Shao L, Cao Y, Jones T, Santosh M, Silva LFO, Ge S, et al. COVID-19 mortality and exposure to airborne PM(2.5): A lag time correlation. Sci Total Environ. 2022;806(Pt 3):151286.
463	Sharma A, Kudchadkar US, Shirodkar R, Usgaonkar UPS, Naik A. Unilateral inferior altitudinal visual field defect related to COVID-19. Indian journal of ophthalmology. 2021;69(4):989-91.
464	Sharma AK, Balyan P. Air pollution and COVID-19: Is the connect worth its weight? Indian J Public Health. 2020;64(Supplement):S132-s4.
465	Sharma D, Campiti VJ, Ye MJ, Saltagi M, Carroll AE, Ting JY, et al. Aerosol generation during cadaveric simulation of otologic surgery and live cochlear implantation. Laryngoscope Investigative Otolaryngology. 2021;6(1):129-36.
466	Shekhtman B, Rizk T, Siebenaler C. COVID-19 and guillain-barre: A case report and literature review. PM and R. 2021;13:S124.
467	Shirazi J, Donzanti MJ, Nelson KM, Zurakowski R, Fromen CA, Gleghorn JP. Significant Unresolved Questions and Opportunities for Bioengineering in Understanding and Treating COVID-19 Disease Progression. Cellular and Molecular Bioengineering. 2020;13(4):259-84.

468	Shokri-Mashhadi N, Kazemi M, Saadat S, Moradi S. Effects of select dietary supplements on the prevention and treatment of viral respiratory tract infections: a systematic review of randomized controlled trials. Expert Review of Respiratory Medicine. 2021;15(6):805-21.
469	Shukla S, Khan R, Saxena A, Sekar S, Ali EF, Shaheen SM. Appraisal of COVID-19 lockdown and unlocking effects on the air quality of North India. Environmental Research. 2022;Part B. 204 (no pagination).
470	Silva ACT, Branco PTBS, Sousa SIV. Impact of COVID-19 Pandemic on Air Quality: A Systematic Review. International Journal of Environmental Research and Public Health. 2022;19(4) (no pagination).
471	Sitovskaia D, Zabrodskaia Y, Verbitskiy O, Petrova Y, Sokolova T. Morphological changes in the spinal cord tissue in patients with Guillain-Barre syndrome associated with COVID-19. Journal of the Neurological Sciences. 2021;Conference: World Congress of Neurology(WCN 2021 . Rome Italy. 429 Supplement).
472	Sivaraman D, Pradeep PS, Sundar Manoharan S, Ramachandra Bhat C, Leela KV, Venugopal V. Current strategies and approaches in combating SARS-CoV-2 virus that causes COVID-19. Letters in Drug Design and Discovery. 2020;17(5):670-2.
473	Skubacz K, Hildebrandt R, Zgorska A, Dyduch Z, Samolej K, Smolinski A. Transport of Aerosols in Underground Mine Workings in Terms of SARS-CoV-2 Virus Threat. Molecules. 2021;26(12).
474	Smith JC, Sausville EL, Girish V, Yuan ML, Vasudevan A, John KM, et al. Cigarette Smoke Exposure and Inflammatory Signaling Increase the Expression of the SARS-CoV-2 Receptor ACE2 in the Respiratory Tract. Dev Cell. 2020;53(5):514-29.e3.
475	Soto Insuga V, Cantarin-Extremera V, Solis-Munoz I, Buendia-Martinez S, Atencia-Ballesteros M, Bernardino B, et al. Pseudotumor Cerebri Caused by SARS-CoV-2 Infection in a Boy. Journal of Pediatric Neurology. 2021;19(3):207-9.
476	Srivastava A. COVID-19 and air pollution and meteorology-an intricate relationship: A review. Chemosphere. 2021;263 (no pagination).
477	Stapleton EM, Welch JL, Ubeda EA, Xiang J, Zabner J, Thornell IM, et al. Urban Particulate Matter Impairment of Airway Surface Liquid-Mediated Coronavirus Inactivation. J Infect Dis. 2022;225(2):214-8.
478	Stapleton EM, Welch JL, Ubeda EA, Xiang J, Zabner J, Thornell IM, et al. Urban Particulate Matter Impairment of Airway Surface Liquid-Mediated Coronavirus Inactivation. The Journal of infectious diseases. 2022;225(2):214-8.
479	Stern R, Koutrakis P, Martins M, Lemos B, Dowd S, Sunderland E, et al. Characterization of airborne sars-cov-2 in a veterans affairs medical center. American Journal of Respiratory and Critical Care Medicine Conference: American Thoracic Society International Conference, ATS. 2021;203(9).
480	Stern RA, Koutrakis P, Martins MAG, Lemos B, Dowd SE, Sunderland EM, et al. Characterization of hospital airborne SARS-CoV-2. Respiratory Research. 2021;22(1) (no pagination).
481	Steward JE, Kitley WR, Schmidt CM, Sundaram CP. Urologic Surgery and COVID-19: How the Pandemic Is Changing the Way We Operate. Journal of Endourology. 2020;34(5):541-9.
482	Stout S, Pandya A, Murphy H, Yeh HW, Portnoy J. Factors Leading to Reduced Unscheduled Pediatric Asthma Visits During COVID-19. Journal of Allergy and Clinical Immunology. 2022;149(2 Supplement):AB183.
483	Stuempfig ND, Nadir NA. A comparison of intubation barrier devices. Academic Emergency Medicine. 2021;28(SUPPL 1):S373-S4.
484	Stufano A, Lisco S, Bartolomeo N, Marsico A, Lucchese G, Jahantigh H, et al. COVID19 outbreak in Lombardy, Italy: An analysis on the short-term relationship between air pollution, climatic factors and the susceptibility to SARS-CoV-2 infection. Environ Res. 2021;198:111197.
485	Stufano A, Lisco S, Bartolomeo N, Marsico A, Lucchese G, Jahantigh H, et al. COVID19 outbreak in Lombardy, Italy: An analysis on the short-term relationship between air pollution, climatic factors and the susceptibility to SARS-CoV-2 infection. Environmental Research. 2021;198 (no pagination).
486	Subat YW, Guntupalli SK, Sajgalik P, Hainy ME, Torgerud KD, Helgeson SA, et al. Aerosol generation during peak flow testing: Clinical implications for COVID-19. Respiratory Care. 2021;66(8):1291-8.
487	Subat YW, Hainy ME, Torgerud KD, Sajgalik P, Guntupalli SK, Johnson BD, et al. Aerosol generation and mitigation during methacholine bronchoprovocation testing: Infection control implications in the era of covid-19. Respiratory Care. 2021;66(12):1858-65.
488	Sullivan JR, Rademaker M, Goodman G, Bekhor P, Al-Niaimi F. Guidance on infection control and plume management with Laser and Energy-Based Devices taking into consideration COVID-19. Australasian Journal of Dermatology. 2021;62(1):37-40.
489	Sumbana J, Sacarlal J, Rubino S. Air pollution and other risk factors might buffer COVID-19 severity in Mozambique. Journal of Infection in Developing Countries. 2020;14(9):994-1000.
490	Sundaram ME, Calzavara A, Mishra S, Kustra R, Chan AK, Hamilton MA, et al. Individual and social determinants of sars-cov-2 testing and positivity in ontario, canada: A population-wide study. Cmaj. 2021;193(20):E723-E34.
491	Szeto CH. Ways to tackle the highly transmissible delta virus. Respirology. 2021;26(SUPPL 3):122-3.
492	Szydelko-Pasko U, Przezdziecka-Dolyk J, Krecicka J, Malecki R, Misiuk-Hojlo M, Turno-Krecicka A. Arteritic Anterior Ischemic Optic Neuropathy in the Course of Giant Cell Arteritis After COVID-19. American Journal of Case Reports. 2022;23(1) (no pagination).
493	Tadano YS, Potgieter-Vermaak S, Kachba YR, Chiroli DMG, Casacio L, Santos-Silva JC, et al. Dynamic model to predict the association between air quality, COVID-19 cases, and level of lockdown. Environmental Pollution. 2021;Part B. 268 (no pagination).
494	Takagi H. Risk and protective factors of SARS-CoV-2 infection. Journal of Medical Virology. 2021;93(2):649-51.
495	Tamama K. Potential benefits of dietary seaweeds as protection against COVID-19. Nutrition Reviews. 2021;79(7):814-23.
496	Tang C, Ling L, Zhang WX. Visualizing Trace Pollutants in Solids at Nanoscale via Electron Tomography. Environmental Science and Technology. 2021;55(17):11533-7.

497	Tang Y, Hu L, Liu Y, Zhou B, Qin X, Ye J, et al. Possible mechanisms of cholesterol elevation aggravating covid-19. International Journal of Medical Sciences. 2021;18(15):3533-43.
498	Tanwar V, Adelstein JM, Wold LE. Double trouble: Combined cardiovascular effects of particulate matter exposure and coronavirus disease 2019. Cardiovascular Research. 2021;117(1):85-95.
499	Tateo F, Fiorino S, Peruzzo L, Zippi M, De Biase D, Lari F, et al. Effects of environmental parameters and their interactions on the spreading of SARS-CoV-2 in North Italy under different social restrictions. A new approach based on multivariate analysis. Environ Res. 2022;210:112921.
500	Tateo F, Fiorino S, Peruzzo L, Zippi M, De Biase D, Lari F, et al. Effects of environmental parameters and their interactions on the spreading of SARS-CoV-2 in North Italy under different social restrictions. A new approach based on multivariate analysis. Environmental Research. 2022;210 (no pagination).
501	Tavella RA, da Silva Junior FMR. Watch out for trends: did ozone increased or decreased during the COVID-19 pandemic? Environmental science and pollution research international. 2021;28(47):67880-5.
502	Thakur AK, Sathyamurthy R, Ramalingam V, Lynch I, Sharshir SW, Ma Z, et al. A case study of SARS-CoV-2 transmission behavior in a severely air-polluted city (Delhi, India) and the potential usage of graphene based materials for filtering air-pollutants and controlling/monitoring the COVID-19 pandemic. Environmental Science: Processes and Impacts. 2021;23(7):923-46.
503	Tivey DR, Davis SS, Kovoor JG, Babidge WJ, Tan L, Hugh TJ, et al. Safe surgery during the coronavirus disease 2019 crisis. ANZ journal of surgery. 2020;90(9):1553-7.
504	Tiwari SK, Dicks LMT, Popov IV, Karaseva A, Ermakov AM, Suvorov A, et al. Probiotics at War Against Viruses: What Is Missing From the Picture? Frontiers in Microbiology. 2020;11 (no pagination).
505	Toczylowski K, Wietlicka-Piszcz M, Grabowska M, Sulik A. Cumulative effects of particulate matter pollution and meteorological variables on the risk of influenza-like illness. Viruses. 2021;13(4) (no pagination).
506	Tomchaney M, Contoli M, Mayo J, Baraldo S, Li S, Cabel CR, et al. Paradoxical effects of cigarette smoke and COPD on SARS-CoV-2 infection and disease. BMC Pulm Med. 2021;21(1):275.
507	Traina G, Barbalace A, Betti F, Bolzacchini E, Bonini M, Contini D, et al. What impact of air pollution in pediatric respiratory allergic diseases. Pediatric Allergy and Immunology. 2020;31(S26):26-8.
508	Travaglio M, Yu Y, Popovic R, Selley L, Leal NS, Martins LM. Links between air pollution and COVID-19 in England. Environ Pollut. 2021;268(Pt A):115859.
509	Travaglio M, Yu Y, Popovic R, Selley L, Leal NS, Martins LM. Links between air pollution and COVID-19 in England. Environmental Pollution. 2021;Part A. 268 (no pagination).
510	Tsatsakis A, Petrakis D, Nikolouzakis TK, Docea AO, Calina D, Vinceti M, et al. COVID-19, an opportunity to reevaluate the correlation between long-term effects of anthropogenic pollutants on viral epidemic/pandemic events and prevalence. Food and Chemical Toxicology. 2020;141 (no pagination).
511	Tsui BCH, Pan S. Distanced-based dynamic behaviour of aerosol particles during aerosol-generating medical procedures. British Journal of Anaesthesia. 2020;125(5):e426-e8.
512	Tung NT, Cheng PC, Chi KH, Hsiao TC, Jones T, BéruBé K, et al. Particulate matter and SARS-CoV-2: A possible model of COVID-19 transmission. Sci Total Environ. 2021;750:141532.
513	Tung NT, Cheng PC, Chi KH, Hsiao TC, Jones T, BeruBe K, et al. Particulate matter and SARS-CoV-2: A possible model of COVID-19 transmission. Science of the Total Environment. 2021;750 (no pagination).
514	Uncini A, Foresti C, Frigeni B, Storti B, Servalli MC, Gazzina S, et al. Electrophysiological features of acute inflammatory demyelinating polyneuropathy associated with SARS-CoV-2 infection. Neurophysiologie Clinique. 2021;51(2):183-91.
515	Urrutia-Pereira M, Mello-da-Silva CA, Sole D. COVID-19 and air pollution: A dangerous association? Allergologia et Immunopathologia. 2020;48(5):496-9.
516	Urrutia-Pereira M, Mello-da-Silva CA, Solé D. Household pollution and COVID-19: irrelevant association? Allergol Immunopathol (Madr). 2021;49(1):146-9.
517	Vasquez-Apestegui V, Parras-Garrido E, Tapia V, Paz-Aparicio VM, Rojas JP, Sánchez-Ccoyllo OR, et al. Association Between Air Pollution in Lima and the High Incidence of COVID-19: Findings from a Post Hoc Analysis. Res Sq. 2020.
518	Veronesi G, De Matteis S, Calori G, Pepe N, Ferrario M. Long term exposure to air pollution and COVID-19 incidence in the city of Varese, Northern Italy: A complete-year, individual-level analysis. Occupational and Environmental Medicine. 2021;78(SUPPL 1):A85-A6.
519	Veronica Lopez M, Vinzon SE, Cafferata EGA, Nunez FJ, Soto A, Sanchez-Lamas M, et al. A single dose of a hybrid hadv5-based anti-covid-19 vaccine induces a long-lasting immune response and broad coverage against voc. Vaccines. 2021;9(10) (no pagination).
520	Versace V, Sebastianelli L, Ferrazzoli D, Saltuari L, Kofler M, Loscher W, et al. Case Report: Myopathy in Critically Ill COVID-19 Patients: A Consequence of Hyperinflammation? Frontiers in Neurology. 2021;12 (no pagination).
521	Vikhe DM, Dhope SV, Mhaske PN, Shah SV, Palekar UG. "Pravara tent"- An innovative protective device to control aerosol in dental clinics during the COVID-19 pandemic. Journal of Clinical and Diagnostic Research. 2020;14(11):ZH01-ZH3.
522	Villanueva F, Notario A, Cabanas B, Martin P, Salgado S, Gabriel MF. Assessment of CO<inf>2</inf> and aerosol (PM<inf>2.5</inf>, PM<inf>10</inf>, UFP) concentrations during the reopening of schools in the COVID-19 pandemic: The case of a metropolitan area in Central-Southern Spain. Environmental Research. 2021;197 (no pagination).
523	Viteri G, Diaz de Mera Y, Rodriguez A, Rodriguez D, Tajuelo M, Escalona A, et al. Impact of SARS-CoV-2 lockdown and de-escalation on air-quality parameters. Chemosphere. 2021;265 (no pagination).
524	Vuorio A, Budowle B, Kovanen PT. Airborne particles and cardiovascular morbidity in severe inherited hypercholesterolemia: Vulnerable endothelium under multiple attacks. BioEssays. 2021.
525	Waheed W, Carey ME, Tandan SR, Tandan R. Post COVID-19 vaccine small fiber neuropathy. Muscle and Nerve. 2021;64(1):E1-E2.

526	Wang C, Wu Z, Liu B, Zhang P, Lu J, Li J, et al. Track-etched membrane microplate and smartphone immunosensing for SARS-CoV-2 neutralizing antibody. Biosensors and Bioelectronics. 2021;192 (no pagination).
527	Wang C, Xie J, Zhao L, Fei X, Zhang H, Tan Y, et al. Alveolar macrophage dysfunction and cytokine storm in the pathogenesis of two severe COVID-19 patients. EBioMedicine. 2020;57 (no pagination).
528	Wang J, Lv M, Xia H, Du J, Zhao Y, Li H, et al. Minimalist Design for a Hand-Held SARS-Cov-2 Sensor: Peptide-Induced Covalent Assembly of Hydrogel Enabling Facile Fiber-Optic Detection of a Virus Marker Protein. ACS sensors. 2021;6(6):2465-71.
529	Wang L, Wang K, Zhong H, Zhao N, Xu W, Yang Y, et al. The Effect of Coronavirus 2019 Disease Control Measures on the Incidence of Respiratory Infectious Disease and Air Pollutant Concentrations in the Yangtze River Delta Region, China. International Journal of Environmental Research and Public Health. 2022;19(3) (no pagination).
530	Wang Y, Xu G, Huang YW. Modeling the load of SARS-CoV-2 virus in human expelled particles during coughing and speaking. PLoS ONE. 2020;15(10 October) (no pagination).
531	Wannaz ED, Larrea Valdivia AE, Reyes Larico JA, Salcedo Peña J, Valenzuela Huillca C. PM(10) correlates with COVID-19 infections 15 days later in Arequipa, Peru. Environ Sci Pollut Res Int. 2021;28(29):39648-54.
532	Wannaz ED, Larrea Valdivia AE, Reyes Larico JA, Salcedo Pena J, Valenzuela Huillca C. PM<inf>10</inf> correlates with COVID-19 infections 15 days later in Arequipa, Peru. Environmental science and pollution research international. 2021;28(29):39648-54.
533	Watad A, De Marco G, Mahajna H, Druyan A, Eltity M, Hijazi N, et al. Immune-mediated disease flares or new-onset disease in 27 subjects following mrna/dna sars-cov-2 vaccination. Vaccines. 2021;9(5) (no pagination).
534	Wathore R, Gupta A, Bherwani H, Labhasetwar N. Understanding air and water borne transmission and survival of coronavirus: Insights and way forward for SARS-CoV-2. Science of the Total Environment. 2020;749 (no pagination).
535	Wei P, Lyu W, Wan T, Zheng Q, Tang W, Li J, et al. COVID-19: a novel risk factor for perioperative neurocognitive disorders. British Journal of Anaesthesia. 2021;127(3):e113-e5.
536	Widiasta A, Sribudiani Y, Nugrahapraja H, Hilmanto D, Sekarwana N, Rachmadi D. Potential role of ACE2-related microRNAs in COVID-19-associated nephropathy. Non-coding RNA Research. 2020;5(4):153-66.
537	Woodby B, Arnold MM, Valacchi G. SARS-CoV-2 infection, COVID-19 pathogenesis, and exposure to air pollution: What is the connection? Annals of the New York Academy of Sciences. 2021;1486(1):15-38.
538	Workman AD, Jafari A, Welling DB, Varvares MA, Gray ST, Holbrook EH, et al. Airborne Aerosol Generation During Endonasal Procedures in the Era of COVID-19: Risks and Recommendations. Otolaryngology - Head and Neck Surgery (United States). 2020;163(3):465-70.
539	Workman AD, Jafari A, Xiao R, Bleier BS. Airborne aerosol olfactory deposition contributes to anosmia in COVID-19. PLoS ONE. 2021;16(2 February) (no pagination).
540	Wu J, Tang Y. Revisiting the Immune Balance Theory: A Neurological Insight Into the Epidemic of COVID-19 and Its Alike. Frontiers in Neurology. 2020;11 (no pagination).
541	Xia Y, S D, Jiang S, Fan R, Wang Y, Wang Y, et al. YiQiFuMai lyophilized injection attenuates particulate matter-induced acute lung injury in mice via TLR4-mTOR-autophagy pathway. Biomed Pharmacother. 2018;108:906-13.
542	Xu L, Taylor JE, Kaiser J. Short-term air pollution exposure and COVID-19 infection in the United States. Environ Pollut. 2022;292(Pt B):118369.
543	Xu L, Taylor JE, Kaiser J. Short-term air pollution exposure and COVID-19 infection in the United States. Environmental Pollution. 2022;Part B. 292 (no pagination).
544	Xu R, Rahmandad H, Gupta M, DiGennaro C, Ghaffarzadegan N, Amini H, et al. Weather, air pollution, and SARS-CoV-2 transmission: a global analysis. The Lancet Planetary Health. 2021;5(10):e671-e80.
545	Xu W, Liu J, Song D, Li C, Zhu A, Long F. Rapid, label-free, and sensitive point-of-care testing of anti-SARS-CoV-2 IgM/IgG using all-fiber Fresnel reflection microfluidic biosensor. Mikrochimica acta. 2021;188(8):261.
546	Xu Y, Zhuang Y, Kang L. A Review of Neurological Involvement in Patients with SARS-CoV-2 Infection. Medical Science Monitor. 2021;27 (no pagination).
547	yang H, Sun G, Tang F, Peng M, Gao Y, Peng J, et al. Clinical features and outcomes of pregnant women suspected of coronavirus disease 2019. Journal of Infection. 2020;81(1):e40-e4.
548	Yang Y, Liu J, Zhou X. A CRISPR-based and post-amplification coupled SARS-CoV-2 detection with a portable evanescent wave biosensor. Biosensors and Bioelectronics. 2021;190 (no pagination).
549	Yao Y, Lawrence DA. Susceptibility to COVID-19 in populations with health disparities: Posited involvement of mitochondrial disorder, socioeconomic stress, and pollutants. Journal of Biochemical and Molecular Toxicology. 2021;35(1) (no pagination).
550	Yao Y, Pan J, Liu Z, Kan H, Qiu Y, Meng X, et al. Association of particulate matter pollution and case fatality rate of COVID-19 in 49 Chinese cities. Science of the Total Environment. 2020;741 (no pagination).
551	Yapici-Eser H, Koroglu YE, Oztop-Cakmak O, Keskin O, Gursoy A, Gursoy-Ozdemir Y. Neuropsychiatric Symptoms of COVID-19 Explained by SARS-CoV-2 Proteins' Mimicry of Human Protein Interactions. Frontiers in Human Neuroscience. 2021;15 (no pagination).
552	Yepez JB, Murati FA, Petitto M, De Yepez J, Galue JM, Revilla J, et al. Vogt-koyanagi-harada disease following COVID-19 infection. Case Reports in Ophthalmology. 2021;12(3):804-8.
553	Young IR, Cowman MK, Kirsch T, Crowley G, Nolan A. COVID-19 and pm exposure: Identifying and mitigating the synergistic deleterious effects. American Journal of Respiratory and Critical Care Medicine Conference: American Thoracic Society International Conference, ATS. 2021;203(9).
554	Yousefi B, Banihashemian S, Feyzabadi Z, Hasanpour S, Kokhaei P, Abdolshahi A, et al. Potential therapeutic effect of oxygen-ozone in controlling of COVID-19 disease. Medical Gas Research. 2022;12(2):33-40.
555	Zangari Del Balzo G. Methodological notes on pandemic virus SARS-CoV-2 research. Theory in biosciences = Theorie in den Biowissenschaften. 2021;140(3):279-94.
556	Zangmeister CD, Radney JG, Vicenzi EP, Weaver JL. Filtration Efficiencies of Nanoscale Aerosol by Cloth Mask Materials Used to Slow the Spread of SARS-CoV-2. ACS nano. 2020;14(7):9188-200.

557	Zhan J, Liu QS, Sun Z, Zhou Q, Hu L, Qu G, et al. Environmental impacts on the transmission and evolution of COVID-19 combing the knowledge of pathogenic respiratory coronaviruses. Environmental Pollution. 2020;267 (no pagination).
558	Zhang J, Zhao C, Zhao W. Virus Caused Imbalance of Type I IFN Responses and Inflammation in COVID-19. Frontiers in Immunology. 2021;12 (no pagination).
559	Zhang R, Zhao H. Small-Angle Particle Counting Coupled Photometry for Real-Time Detection of Respirable Particle Size Segmentation Mass Concentration. Sensors. 2021;21(17).
560	Zhang S, Chen S, Xiao G, Zhao M, Li J, Dong W, et al. The associations between air pollutant exposure and neutralizing antibody titers of an inactivated SARS-CoV-2 vaccine. Environmental science and pollution research international. 2022;29(9):13720-8.
561	Zhang X, Zhang Z, Xia N, Zhao Q. Carbohydrate-containing nanoparticles as vaccine adjuvants. Expert Review of Vaccines. 2021;20(7):797-810.
562	Zhang XS, Duchaine C. SARS-CoV-2 and health care worker protection in low-risk settings: A review of modes of transmission and a novel airborne model involving inhalable particles. Clinical Microbiology Reviews. 2020;34(1):1-29.
563	Zhao C, Fang X, Feng Y, Fang X, He J, Pan H. Emerging role of air pollution and meteorological parameters in COVID-19. J Evid Based Med. 2021;14(2):123-38.
564	Zhao M, Liu Y, Gyilbag A. Assessment of Meteorological Variables and Air Pollution Affecting COVID-19 Cases in Urban Agglomerations: Evidence from China. Int J Environ Res Public Health. 2022;19(1).
565	Zhao M, Liu Y, Gyilbag A. Assessment of Meteorological Variables and Air Pollution Affecting COVID-19 Cases in Urban Agglomerations: Evidence from China. International Journal of Environmental Research and Public Health. 2022;19(1) (no pagination).
566	Zhou N, Dai H, Zha W, Lv Y. The impact of meteorological factors and PM2.5 on COVID-19 transmission. Epidemiology and Infection. 2022.
567	Zhu TY, Qiu H, Cao QQ, Duan ZL, Liu FL, Song TZ, et al. Particulate matter exposure exacerbates susceptibility to SARS-CoV-2 infection in humanized ACE2 mice. Zool Res. 2021;42(3):335-8.
568	Zor KR, Yildirim Bicer G, Tutas Gunaydin N, Kucuk E, Yilmaz U. Can the coronavirus disease 2019 (COVID-19) cause choroiditis and optic neuropathy? European Journal of Inflammation. 2021;19(no pagination).
569	Zoran MA, Savastru RS, Savastru DM, Tautan MN. Assessing the relationship between surface levels of PM2.5 and PM10 particulate matter impact on COVID-19 in Milan, Italy. Sci Total Environ. 2020;738:139825.
570	Zoran MA, Savastru RS, Savastru DM, Tautan MN. Assessing the relationship between ground levels of ozone (O<inf>3</inf>) and nitrogen dioxide (NO<inf>2</inf>) with coronavirus (COVID-19) in Milan, Italy. Science of the Total Environment. 2020;740 (no pagination).
571	Zoran MA, Savastru RS, Savastru DM, Tautan MN. Assessing the relationship between surface levels of PM2.5 and PM10 particulate matter impact on COVID-19 in Milan, Italy. Science of the Total Environment. 2020;738 (no pagination).
572	Zuniga-Montanez R, Coil DA, Eisen JA, Pechacek R, Guerrero RG, Kim M, et al. The challenge of SARS-CoV-2 environmental monitoring in schools using floors and portable HEPA filtration units: Fresh or relic RNA? medRxiv. 2021;15.

Supplementary Table S2. PUBMED Identified Manuscripts (N= 334)

1	Zoran MA, Savastru RS, Savastru DM, Tautan MN. Assessing the relationship between surface levels of PM2.5 and PM10 particulate matter impact on COVID-19 in Milan, Italy. Sci Total Environ. 2020;738:139825.
2	Zhu TY, Qiu H, Cao QQ, Duan ZL, Liu FL, Song TZ, et al. Particulate matter exposure exacerbates susceptibility to SARS-CoV-2 infection in humanized ACE2 mice. Zool Res. 2021;42(3):335-8.
3	Zhu C, Maharajan K, Liu K, Zhang Y. Role of atmospheric particulate matter exposure in COVID-19 and other health risks in human: A review. Environ Res. 2021;198:111281.
4	Zhao M, Liu Y, Gyilbag A. Assessment of Meteorological Variables and Air Pollution Affecting COVID-19 Cases in Urban Agglomerations: Evidence from China. Int J Environ Res Public Health. 2022;19(1).
5	Zhao L, Zhang FS, Wang K, Zhu J. Chemical properties of heavy metals in typical hospital waste incinerator ashes in China. Waste Manag. 2009;29(3):1114-21.
6	Zhao C, Fang X, Feng Y, Fang X, He J, Pan H. Emerging role of air pollution and meteorological parameters in COVID-19. J Evid Based Med. 2021;14(2):123-38.
7	Zhang Z, Ji D, He H, Ramakrishna S. Electrospun ultrafine fibers for advanced face masks. Mater Sci Eng R Rep. 2021;143:100594.
8	Zhang YH, Guo GH, Shen GL, Han W, Zhao XY, Lin W, et al. [Analysis on treatment of extremely severe burn patients with severe inhalation injury in August 2nd Kunshan factory aluminum dust explosion accident]. Zhonghua Shao Shang Za Zhi. 2018;34(7):455-8.
9	Zhang XS, Duchaine C. SARS-CoV-2 and Health Care Worker Protection in Low-Risk Settings: a Review of Modes of Transmission and a Novel Airborne Model Involving Inhalable Particles. Clin Microbiol Rev. 2020;34(1).
10	Zhang R, Zhao H. Small-Angle Particle Counting Coupled Photometry for Real-Time Detection of Respirable Particle Size Segmentation Mass Concentration. Sensors (Basel). 2021;21(17).
11	Zerefos CS, Solomos S, Kapsomenakis J, Poupkou A, Dimitriadou L, Polychroni ID, et al. Lessons learned and questions raised during and post-COVID-19 anthropopause period in relation to the environment and climate. Environ Dev Sustain. 2021;23(7):10623-45.
12	Zangari Del Balzo G. Methodological notes on pandemic virus SARS-CoV-2 research. Theory Biosci. 2021;140(3):279-94.
13	Yuan JT, Jiang SIB. Urgent safety considerations for dermatologic surgeons in the COVID-19 pandemic. Dermatol Online J. 2020;26(8).
14	Yokoe T, Kita M, Odaka T, Fujisawa J, Hisamatsu Y, Okada H. Detection of human coronavirus RNA in surgical smoke generated by surgical devices. J Hosp Infect. 2021;117:89-95.
15	Yao Y, Pan J, Wang W, Liu Z, Kan H, Qiu Y, et al. Association of particulate matter pollution and case fatality rate of COVID-19 in 49 Chinese cities. Sci Total Environ. 2020;741:140396.
16	Yao Y, Pan J, Liu Z, Meng X, Wang W, Kan H, et al. Ambient nitrogen dioxide pollution and spreadability of COVID-19 in Chinese cities. Ecotoxicol Environ Saf. 2021;208:111421.
17	Yang FW, Xin HM, Zhu JH, Feng XY, Jiang XC, Gong ZY, et al. [Treatment of patients with different degree of acute respiratory distress syndrome caused by inhalation of white smoke]. Zhonghua Shao Shang Za Zhi. 2017;33(12):760-5.
18	Xu L, Taylor JE, Kaiser J. Short-term air pollution exposure and COVID-19 infection in the United States. Environ Pollut. 2022;292(Pt B):118369.
19	Xie F, Zhang X, Xie L. Prognostic value of serum zinc levels in patients with acute HC/zinc chloride smoke inhalation. Medicine (Baltimore). 2017;96(39):e8156.
20	Xiao LI, Sakagami H, Miwa N. A New Method for Testing Filtration Efficiency of Mask Materials Under Sneeze-like Pressure. In Vivo. 2020;34(3 Suppl):1637-44.
21	Xia Y, S D, Jiang S, Fan R, Wang Y, Wang Y, et al. YiQiFuMai lyophilized injection attenuates particulate matter-induced acute lung injury in mice via TLR4-mTOR-autophagy pathway. Biomed Pharmacother. 2018;108:906-13.
22	Workman AD, Jafari A, Welling DB, Varvares MA, Gray ST, Holbrook EH, et al. Airborne Aerosol Generation During Endonasal Procedures in the Era of COVID-19: Risks and Recommendations. Otolaryngol Head Neck Surg. 2020;163(3):465-70.
23	Woodby B, Arnold MM, Valacchi G. SARS-CoV-2 infection, COVID-19 pathogenesis, and exposure to air pollution: What is the connection? Ann N Y Acad Sci. 2021;1486(1):15-38.
24	Wohnhaas CT, Gindele JA, Kiechle T, Shen Y, Leparc GG, Stierstorfer B, et al. Cigarette Smoke Specifically Affects Small Airway Epithelial Cell Populations and Triggers the Expansion of Inflammatory and Squamous Differentiation Associated Basal Cells. Int J Mol Sci. 2021;22(14).
25	Wathore R, Gupta A, Bherwani H, Labhasetwar N. Understanding air and water borne transmission and survival of coronavirus: Insights and way forward for SARS-CoV-2. Sci Total Environ. 2020;749:141486.
26	Wannaz ED, Larrea Valdivia AE, Reyes Larico JA, Salcedo Peña J, Valenzuela Huillca C. PM(10) correlates with COVID-19 infections 15 days later in Arequipa, Peru. Environ Sci Pollut Res Int. 2021;28(29):39648-54.
27	Wang Y, Xue Q. The implications of COVID-19 in the ambient environment and psychological conditions. NanoImpact. 2021;21:100295.
28	Wang C, Wolters PJ, Calfee CS, Liu S, Balmes JR, Zhao Z, et al. Long-term ozone exposure is positively associated with telomere length in critically ill patients. Environ Int. 2020;141:105780.
29	Vuorio A, Budowle B, Kovanen PT. Airborne particles and cardiovascular morbidity in severe inherited hypercholesterolemia: Vulnerable endothelium under multiple attacks. Bioessays. 2022;44(3):e2100273.

30	Viteri G, Díaz de Mera Y, Rodríguez A, Rodríguez D, Tajuelo M, Escalona A, et al. Impact of SARS-CoV-2 lockdown and de-escalation on air-quality parameters. Chemosphere. 2021;265:129027.
31	Villanueva F, Notario A, Cabañas B, Martín P, Salgado S, Gabriel MF. Assessment of CO(2) and aerosol (PM(2.5), PM(10), UFP) concentrations during the reopening of schools in the COVID-19 pandemic: The case of a metropolitan area in Central-Southern Spain. Environ Res. 2021;197:111092.
32	Vasquez-Apestegui V, Parras-Garrido E, Tapia V, Paz-Aparicio VM, Rojas JP, Sánchez-Ccoyllo OR, et al. Association Between Air Pollution in Lima and the High Incidence of COVID-19: Findings from a Post Hoc Analysis. Res Sq. 2020.
33	Valdés Salgado M, Smith P, Opazo MA, Huneeus N. Long-Term Exposure to Fine and Coarse Particulate Matter and COVID-19 Incidence and Mortality Rate in Chile during 2020. Int J Environ Res Public Health. 2021;18(14).
34	Urrutia-Pereira M, Mello-da-Silva CA, Solé D. Household pollution and COVID-19: irrelevant association? Allergol Immunopathol (Madr). 2021;49(1):146-9.
35	Urrutia-Pereira M, Mello-da-Silva CA, Solé D. COVID-19 and air pollution: A dangerous association? Allergol Immunopathol (Madr). 2020;48(5):496-9.
36	Tung NT, Cheng PC, Chi KH, Hsiao TC, Jones T, BéruBé K, et al. Particulate matter and SARS-CoV-2: A possible model of COVID-19 transmission. Sci Total Environ. 2021;750:141532.
37	Travaglio M, Yu Y, Popovic R, Selley L, Leal NS, Martins LM. Links between air pollution and COVID-19 in England. Environ Pollut. 2021;268(Pt A):115859.
38	Traina G, Barbalace A, Betti F, Bolzacchini E, Bonini M, Contini D, et al. What impact of air pollution in pediatric respiratory allergic diseases. Pediatr Allergy Immunol. 2020;31 Suppl 26:26-8.
39	Tomei Torres FA. [Strategies in the prevention and control of the Covid-19 pandemic caused by SARS-CoV-2. Environmental factors.]. Rev Esp Salud Publica. 2020;94.
40	Tomchaney M, Contoli M, Mayo J, Baraldo S, Li S, Cabel CR, et al. Paradoxical effects of cigarette smoke and COPD on SARS-CoV-2 infection and disease. BMC Pulm Med. 2021;21(1):275.
41	Toczylowski K, Wietlicka-Piszcz M, Grabowska M, Sulik A. Cumulative Effects of Particulate Matter Pollution and Meteorological Variables on the Risk of Influenza-Like Illness. Viruses. 2021;13(4).
42	Tobías A, Carnerero C, Reche C, Massagué J, Via M, Minguillón MC, et al. Changes in air quality during the lockdown in Barcelona (Spain) one month into the SARS-CoV-2 epidemic. Sci Total Environ. 2020;726:138540.
43	Teixidó O, Tobías A, Massagué J, Mohamed R, Ekaabi R, Hamed HI, et al. The influence of COVID-19 preventive measures on the air quality in Abu Dhabi (United Arab Emirates). Air Qual Atmos Health. 2021;14(7):1071-9.
44	Tavella RA, da Silva Júnior FMR. Watch out for trends: did ozone increased or decreased during the COVID-19 pandemic? Environ Sci Pollut Res Int. 2021;28(47):67880-5.
45	Tateo F, Fiorino S, Peruzzo L, Zippi M, De Biase D, Lari F, et al. Effects of environmental parameters and their interactions on the spreading of SARS-CoV-2 in North Italy under different social restrictions. A new approach based on multivariate analysis. Environ Res. 2022;210:112921.
46	Tanwar V, Adelstein JM, Wold LE. Double trouble: combined cardiovascular effects of particulate matter exposure and coronavirus disease 2019. Cardiovasc Res. 2021;117(1):85-95.
47	Takagi H. Risk and protective factors of SARS-CoV-2 infection. J Med Virol. 2021;93(2):649-51.
48	Tadano YS, Potgieter-Vermaak S, Kachba YR, Chiroli DMG, Casacio L, Santos-Silva JC, et al. Dynamic model to predict the association between air quality, COVID-19 cases, and level of lockdown. Environ Pollut. 2021;268(Pt B):115920.
49	Sullivan JR, Rademaker M, Goodman G, Bekhor P, Al-Niaimi F. Guidance on infection control and plume management with Laser and Energy-Based Devices taking into consideration COVID-19. Australas J Dermatol. 2021;62(1):37-40.
50	Subat YW, Hainy ME, Torgerud KD, Sajgalik P, Guntupalli SK, Johnson BD, et al. Aerosol Generation and Mitigation During Methacholine Bronchoprovocation Testing: Infection Control Implications in the Era of COVID-19. Respir Care. 2021;66(12):1858-65.
51	Subat YW, Guntupalli SK, Sajgalik P, Hainy ME, Torgerud KD, Helgeson SA, et al. Aerosol Generation During Peak Flow Testing: Clinical Implications for COVID-19. Respir Care. 2021;66(8):1291-8.
52	Su X, Sutarlie L, Loh XJ. Sensors and Analytical Technologies for Air Quality: Particulate Matters and Bioaerosols. Chem Asian J. 2020;15(24):4241-55.
53	Stufano A, Lisco S, Bartolomeo N, Marsico A, Lucchese G, Jahantigh H, et al. COVID19 outbreak in Lombardy, Italy: An analysis on the short-term relationship between air pollution, climatic factors and the susceptibility to SARS-CoV-2 infection. Environ Res. 2021;198:111197.
54	Stevens KA, Bryer TA, Yu H. Air Quality Enhancement Districts: democratizing data to improve respiratory health. J Environ Stud Sci. 2021;11(4):702-7.
55	Stern RA, Koutrakis P, Martins MAG, Lemos B, Dowd SE, Sunderland EM, et al. Characterization of hospital airborne SARS-CoV-2. Respir Res. 2021;22(1):73.
56	Stenlo M, Silva IAN, Hyllén S, Bölükbas DA, Niroomand A, Grins E, et al. Monitoring lung injury with particle flow rate in LPS- and COVID-19-induced ARDS. Physiol Rep. 2021;9(13):e14802.
57	Stapleton EM, Welch JL, Ubeda EA, Xiang J, Zabner J, Thornell IM, et al. Urban Particulate Matter Impairment of Airway Surface Liquid-Mediated Coronavirus Inactivation. J Infect Dis. 2022;225(2):214-8.
58	Srivastava A. COVID-19 and air pollution and meteorology-an intricate relationship: A review. Chemosphere. 2021;263:128297.
59	Smith JC, Sausville EL, Girish V, Yuan ML, Vasudevan A, John KM, et al. Cigarette Smoke Exposure and Inflammatory Signaling Increase the Expression of the SARS-CoV-2 Receptor ACE2 in the Respiratory Tract. Dev Cell. 2020;53(5):514-29.e3.
60	Skubacz K, Hildebrandt R, Zgórska A, Dyduch Z, Samolej K, Smolinski A. Transport of Aerosols in Underground Mine Workings in Terms of SARS-CoV-2 Virus Threat. Molecules. 2021;26(12).

61	Sinnige JS, Kooij FO, van Schuppen H, Hollmann MW, Sperna Weiland NH. Protection of healthcare workers during aerosol-generating procedures with local exhaust ventilation. Br J Anaesth. 2021;126(6):e220-e2.
62	Singh A, Salunke P, Chhabra R, Sethi S, Sahoo SK, Karthigeyan M, et al. The Risk of Spread of Infection During Craniotomy/Craniostomy on Patients with Active Coronavirus Disease 2019 (COVID-19) Infection: Myth or Fact? World Neurosurg. 2021;147:e272-e4.
63	Shukla S, Khan R, Saxena A, Sekar S, Ali EF, Shaheen SM. Appraisal of COVID-19 lockdown and unlocking effects on the air quality of North India. Environ Res. 2022;204(Pt B):112107.
64	Shimazaki Y, Okubo M, Yamamoto T. Three-dimensional Numerical Simulation of Gas-particulate Flow around Breathing Human and Particulate Inhalation. AIP Conf Proc. 2006;832(1):439-44.
65	Sharma AK, Balyan P. Air pollution and COVID-19: Is the connect worth its weight? Indian J Public Health. 2020;64(Supplement):S132-s4.
66	Shao L, Cao Y, Jones T, Santosh M, Silva LFO, Ge S, et al. COVID-19 mortality and exposure to airborne PM(2.5): A lag time correlation. Sci Total Environ. 2022;806(Pt 3):151286.
67	Setti L, Passarini F, De Gennaro G, Barbieri P, Perrone MG, Borelli M, et al. SARS-Cov-2RNA found on particulate matter of Bergamo in Northern Italy: First evidence. Environ Res. 2020;188:109754.
68	Setti L, Passarini F, De Gennaro G, Barbieri P, Perrone MG, Borelli M, et al. Airborne Transmission Route of COVID-19: Why 2 Meters/6 Feet of Inter-Personal Distance Could Not Be Enough. Int J Environ Res Public Health. 2020;17(8).
69	Setti L, Passarini F, De Gennaro G, Barbieri P, Pallavicini A, Ruscio M, et al. Searching for SARS-COV-2 on Particulate Matter: A Possible Early Indicator of COVID-19 Epidemic Recurrence. Int J Environ Res Public Health. 2020;17(9).
70	Senatore V, Zarra T, Buonerba A, Choo KH, Hasan SW, Korshin G, et al. Indoor versus outdoor transmission of SARS-COV-2: environmental factors in virus spread and underestimated sources of risk. EuroMediterr J Environ Integr. 2021;6(1):30.
71	Selvam S, Muthukumar P, Venkatramanan S, Roy PD, Manikanda Bharath K, Jesuraja K. SARS-CoV-2 pandemic lockdown: Effects on air quality in the industrialized Gujarat state of India. Sci Total Environ. 2020;737:140391.
72	Sciomer S, Moscucci F, Magrì D, Badagliacca R, Piccirillo G, Agostoni P. SARS-CoV-2 spread in Northern Italy: what about the pollution role? Environ Monit Assess. 2020;192(6):325.
73	Scheier T, Shah C, Huber M, Sax H, Hasse B, Günthard HF, et al. Do we cause false positives? An experimental series on droplet or airborne SARS-CoV-2 contamination of sampling tubes during swab collection in a test center. Antimicrob Resist Infect Control. 2021;10(1):51.
74	Scalsky RJ, Chen YJ, Ying Z, Perry JA, Hong CC. The Social and Natural Environment's Impact on SARS-CoV-2 Infections in the UK Biobank. Int J Environ Res Public Health. 2022;19(1).
75	Santurtún A, Colom ML, Fdez-Arroyabe P, Real Á D, Fernández-Olmo I, Zarrabeitia MT. Exposure to particulate matter: Direct and indirect role in the COVID-19 pandemic. Environ Res. 2022;206:112261.
76	Sangkham S, Thongtip S, Vongruang P. Influence of air pollution and meteorological factors on the spread of COVID-19 in the Bangkok Metropolitan Region and air quality during the outbreak. Environ Res. 2021;197:111104.
77	Samillan VJ, Flores-León D, Rojas E, Zutta BR. Environmental and climatic impact on the infection and mortality of SARS-CoV-2 in Peru. J Basic Clin Physiol Pharmacol. 2021;32(5):935-42.
78	Saleh Y, Antherieu S, Dusautoir R, L YA, Sotty J, De Sousa C, et al. Exposure to Atmospheric Ultrafine Particles Induces Severe Lung Inflammatory Response and Tissue Remodeling in Mice. Int J Environ Res Public Health. 2019;16(7).
79	Sahoo MM. Significance between air pollutants, meteorological factors, and COVID-19 infections: probable evidences in India. Environ Sci Pollut Res Int. 2021;28(30):40474-95.
80	Sagawa T, Tsujikawa T, Honda A, Miyasaka N, Tanaka M, Kida T, et al. Exposure to particulate matter upregulates ACE2 and TMPRSS2 expression in the murine lung. Environ Res. 2021;195:110722.
81	Ruran HB, Adamkiewicz G, Cunningham A, Petty CR, Greco KF, Gunnlaugsson S, et al. Air quality, Environment and Respiratory Outcomes in Bronchopulmonary Dysplasia, the AERO-BPD cohort study: design and adaptation during the SARS-CoV-2 pandemic. BMJ Open Respir Res. 2021;8(1).
82	Rugani B, Conticini E, Frediani B, Caro D. Decrease in life expectancy due to COVID-19 disease not offset by reduced environmental impacts associated with lockdowns in Italy. Environ Pollut. 2022;292(Pt A):118224.
83	Roy S, Saha M, Dhar B, Pandit S, Nasrin R. Geospatial analysis of COVID-19 lockdown effects on air quality in the South and Southeast Asian region. Sci Total Environ. 2021;756:144009.
84	Roviello V, Roviello GN. Lower COVID-19 mortality in Italian forested areas suggests immunoprotection by Mediterranean plants. Environ Chem Lett. 2020:1-12.
85	Rovetta A, Castaldo L. Relationships between Demographic, Geographic, and Environmental Statistics and the Spread of Novel Coronavirus Disease (COVID-19) in Italy. Cureus. 2020;12(11):e11397.
86	Rovetta A, Bhagavathula AS, Castaldo L. Modeling the Epidemiological Trend and Behavior of COVID-19 in Italy. Cureus. 2020;12(8):e9884.
87	Rodríguez-Urrego D, Rodríguez-Urrego L. Air quality during the COVID-19: PM(2.5) analysis in the 50 most polluted capital cities in the world. Environ Pollut. 2020;266(Pt 1):115042.
88	Robertson-More C, Wu T. A knowledge gap unmasked: viral transmission in surgical smoke: a systematic review. Surg Endosc. 2021;35(6):2428-39.
89	Rivera-Rios JC, Joo T, Takeuchi M, Orlando TM, Bevington T, Mathis JW, et al. In-flight particulate matter concentrations in commercial flights are likely lower than other indoor environments. Indoor Air. 2021;31(5):1484-94.
90	Reyes MSS, Medina PMB. Environmental pollutant exposure can exacerbate COVID-19 neurologic symptoms. Med Hypotheses. 2020;144:110136.
91	Rexhepi I, Mangifesta R, Santilli M, Guri S, Di Carlo P, D'Addazio G, et al. Effects of Natural Ventilation and Saliva Standard Ejectors during the COVID-19 Pandemic: A Quantitative Analysis of Aerosol Produced during Dental Procedures. Int J Environ Res Public Health. 2021;18(14).

92	Reilly JP, Zhao Z, Shashaty MGS, Koyama T, Christie JD, Lanken PN, et al. Low to Moderate Air Pollutant Exposure and Acute Respiratory Distress Syndrome after Severe Trauma. Am J Respir Crit Care Med. 2019;199(1):62-70.
93	Rebuli ME, Brocke SA, Jaspers I. Impact of inhaled pollutants on response to viral infection in controlled exposures. J Allergy Clin Immunol. 2021;148(6):1420-9.
94	Ray RL, Singh VP, Singh SK, Acharya BS, He Y. What is the impact of COVID-19 pandemic on global carbon emissions? Sci Total Environ. 2022;816:151503.
95	Ravenel TD, Kessler R, Comisi JC, Kelly A, Renne WG, Teich ST. Evaluation of the spatter-reduction effectiveness and aerosol containment of eight dry-field isolation techniques. Quintessence Int. 2020;51(8):660-70.
96	Ram K, Thakur RC, Singh DK, Kawamura K, Shimouchi A, Sekine Y, et al. Why airborne transmission hasn't been conclusive in case of COVID-19? An atmospheric science perspective. Sci Total Environ. 2021;773:145525.
97	Rajajee V, Williamson CA. Use of a Novel Negative-Pressure Tent During Bedside Tracheostomy in COVID-19 Patients. Neurocrit Care. 2020;33(2):597-603.
98	Querol X, Massagué J, Alastuey A, Moreno T, Gangoiti G, Mantilla E, et al. Lessons from the COVID-19 air pollution decrease in Spain: Now what? Sci Total Environ. 2021;779:146380.
99	Pozzer A, Dominici F, Haines A, Witt C, Münzel T, Lelieveld J. Regional and global contributions of air pollution to risk of death from COVID-19. Cardiovasc Res. 2020;116(14):2247-53.
100	Poyraz BM, Engin ED, Engin AB, Engin A. The effect of environmental diesel exhaust pollution on SARS-CoV-2 infection: The mechanism of pulmonary ground glass opacity. Environ Toxicol Pharmacol. 2021;86:103657.
101	Ponce de Leon S, Lazcano A. Panspermia--true or false? Lancet. 2003;362(9381):406-7; author reply 7-8.
102	Pivato A, Amoruso I, Formenton G, Di Maria F, Bonato T, Vanin S, et al. Evaluating the presence of SARS-CoV-2 RNA in the particulate matters during the peak of COVID-19 in Padua, northern Italy. Sci Total Environ. 2021;784:147129.
103	Pierpaoli M, Giosuè C, Czerwińska N, Rycewicz M, Wieloszyńska A, Bogdanowicz R, et al. Characterization and Filtration Efficiency of Sustainable PLA Fibers Obtained via a Hybrid 3D-Printed/Electrospinning Technique. Materials (Basel). 2021;14(22).
104	Perret J, Dharmage S. COVID-19 hospitalizations: Another adverse impact of ambient air pollution? Respirology. 2021;26(12):1101-2.
105	Pearce E, Campen MJ, Baca JT, Blewett JP, Femling J, Hanson DT, et al. Aerosol generation with various approaches to oxygenation in healthy volunteers in the emergency department. J Am Coll Emerg Physicians Open. 2021;2(2):e12390.
106	Pavan N, Crestani A, Abrate A, De Nunzio C, Esperto F, Giannarini G, et al. Risk of Virus Contamination Through Surgical Smoke During Minimally Invasive Surgery: A Systematic Review of the Literature on a Neglected Issue Revived in the COVID-19 Pandemic Era. Eur Urol Focus. 2020;6(5):1058-69.
107	Patel H, Talbot N, Salmond J, Dirks K, Xie S, Davy P. Implications for air quality management of changes in air quality during lockdown in Auckland (New Zealand) in response to the 2020 SARS-CoV-2 epidemic. Sci Total Environ. 2020;746:141129.
108	Pasquier J, Villalta O, Sarria Lamorú S, Balagué C, Vilallonga R, Targarona EM. Are Smoke and Aerosols Generated During Laparoscopic Surgery a Biohazard? A Systematic Evidence-Based Review. Surg Innov. 2021;28(4):485-95.
109	Parida BR, Bar S, Roberts G, Mandal SP, Pandey AC, Kumar M, et al. Improvement in air quality and its impact on land surface temperature in major urban areas across India during the first lockdown of the pandemic. Environ Res. 2021;199:111280.
110	Pandey AS, Ringer AJ, Rai AT, Kan P, Jabbour P, Siddiqui AH, et al. Minimizing SARS-CoV-2 exposure when performing surgical interventions during the COVID-19 pandemic. J Neurointerv Surg. 2020;12(7):643-7.
111	Paital B, Agrawal PK. Air pollution by NO(2) and PM(2.5) explains COVID-19 infection severity by overexpression of angiotensin-converting enzyme 2 in respiratory cells: a review. Environ Chem Lett. 2021;19(1):25-42.
112	Páez-Osuna F, Valencia-Castañeda G, Rebolledo UA. The link between COVID-19 mortality and PM(2.5) emissions in rural and medium-size municipalities considering population density, dust events, and wind speed. Chemosphere. 2022;286(Pt 1):131634.
113	Orak NH, Ozdemir O. The impacts of COVID-19 lockdown on PM(10) and SO(2) concentrations and association with human mobility across Turkey. Environ Res. 2021;197:111018.
114	Olloquequi J. COVID-19 Susceptibility in chronic obstructive pulmonary disease. Eur J Clin Invest. 2020;50(10):e13382.
115	Nugroho A, Saunar R, Lalisang TJM, Wiradisuria E. Local adaptation of laparoscopic smoke evacuator in COVID-19 pandemic situation. Asian J Endosc Surg. 2021;14(3):620-3.
116	Nozza E, Valentini S, Melzi G, Vecchi R, Corsini E. Advances on the immunotoxicity of outdoor particulate matter: A focus on physical and chemical properties and respiratory defence mechanisms. Sci Total Environ. 2021;780:146391.
117	Norouzi N, Asadi Z. Air pollution impact on the Covid-19 mortality in Iran considering the comorbidity (obesity, diabetes, and hypertension) correlations. Environ Res. 2022;204(Pt A):112020.
118	Nor NSM, Yip CW, Ibrahim N, Jaafar MH, Rashid ZZ, Mustafa N, et al. Particulate matter (PM(2.5)) as a potential SARS-CoV-2 carrier. Sci Rep. 2021;11(1):2508.
119	Navarro KM, Clark KA, Hardt DJ, Reid CE, Lahm PW, Domitrovich JW, et al. Wildland firefighter exposure to smoke and COVID-19: A new risk on the fire line. Sci Total Environ. 2021;760:144296.
120	Naqvi HR, Datta M, Mutreja G, Siddiqui MA, Naqvi DF, Naqvi AR. Improved air quality and associated mortalities in India under COVID-19 lockdown. Environ Pollut. 2021;268(Pt A):115691.
121	Naidoo P, Ghazi T, Chuturgoon AA, Naidoo RN, Ramsuran V, Mpaka-Mbatha MN, et al. SARS-CoV-2 and helminth co-infections, and environmental pollution exposure: An epidemiological and immunological perspective. Environ Int. 2021;156:106695.

122	Mulder C, Conti E, Saccone S, Federico C. Beyond virology: environmental constraints of the first wave of COVID-19 cases in Italy. Environ Sci Pollut Res Int. 2021;28(24):31996-2004.
123	Mukherjee S, Boral S, Siddiqi H, Mishra A, Meikap BC. Present cum future of SARS-CoV-2 virus and its associated control of virus-laden air pollutants leading to potential environmental threat - A global review. J Environ Chem Eng. 2021;9(2):104973.
124	Moshammer H, Poteser M, Hutter HP. COVID-19 and air pollution in Vienna-a time series approach. Wien Klin Wochenschr. 2021;133(17-18):951-7.
125	Moelling K, Broecker F. Air Microbiome and Pollution: Composition and Potential Effects on Human Health, Including SARS Coronavirus Infection. J Environ Public Health. 2020;2020:1646943.
126	Mintz Y, Arezzo A, Boni L, Baldari L, Cassinotti E, Brodie R, et al. The risk of COVID-19 transmission by laparoscopic smoke may be lower than for laparotomy: a narrative review. Surg Endosc. 2020;34(8):3298-305.
127	Miller PW, Reesman C, Grossman MK, Nelson SA, Liu V, Wang P. Marginal warming associated with a COVID-19 quarantine and the implications for disease transmission. Sci Total Environ. 2021;780:146579.
128	Mettias B, Mair M, Conboy P. COVID-19 Cross-Infection Rate After Surgical Procedures: Incidence and Outcome. Laryngoscope. 2021;131(11):E2749-e54.
129	Mescoli A, Maffei G, Pillo G, Bortone G, Marchesi S, Morandi E, et al. The Secretive Liaison of Particulate Matter and SARS-CoV-2. A Hypothesis and Theory Investigation. Front Genet. 2020;11:579964.
130	Meo SA, Almutairi FJ, Abukhalaf AA, Usmani AM. Effect of Green Space Environment on Air Pollutants PM2.5, PM10, CO, O(3), and Incidence and Mortality of SARS-CoV-2 in Highly Green and Less-Green Countries. Int J Environ Res Public Health. 2021;18(24).
131	Meo SA, Almutairi FJ, Abukhalaf AA, Alessa OM, Al-Khlaiwi T, Meo AS. Sandstorm and its effect on particulate matter PM 2.5, carbon monoxide, nitrogen dioxide, ozone pollutants and SARS-CoV-2 cases and deaths. Sci Total Environ. 2021;795:148764.
132	Meo SA, Al-Khlaiwi T, Ullah CH. Effect of ambient air pollutants PM2.5 and PM10 on COVID-19 incidence and mortality: observational study. Eur Rev Med Pharmacol Sci. 2021;25(23):7553-64.
133	Meo SA, Ahmed Alqahtani S, Saad Binmeather F, Abdulrhman AlRasheed R, Mohammed Aljedaie G, Mohammed Albarrak R. Effect of environmental pollutants PM2.5, CO, O(3) and NO(2), on the incidence and mortality of SARS-COV-2 in largest metropolitan cities, Delhi, Mumbai and Kolkata, India. J King Saud Univ Sci. 2022;34(1):101687.
134	Meo SA, Adnan Abukhalaf A, Sami W, Hoang TD. Effect of environmental pollution PM2.5, carbon monoxide, and ozone on the incidence and mortality due to SARS-CoV-2 infection in London, United Kingdom. J King Saud Univ Sci. 2021;33(3):101373.
135	Meo SA, Abukhalaf AA, Alomar AA, Alessa OM, Sami W, Klonoff DC. Effect of environmental pollutants PM-2.5, carbon monoxide, and ozone on the incidence and mortality of SARS-COV-2 infection in ten wildfire affected counties in California. Sci Total Environ. 2021;757:143948.
136	Meo SA, Abukhalaf AA, Alomar AA, Alessa OM. Wildfire and COVID-19 pandemic: effect of environmental pollution PM-2.5 and carbon monoxide on the dynamics of daily cases and deaths due to SARS-COV-2 infection in San-Francisco USA. Eur Rev Med Pharmacol Sci. 2020;24(19):10286-92.
137	Meo SA, Abukhalaf AA, Alessa OM, Alarifi AS, Sami W, Klonoff DC. Effect of Environmental Pollutants PM2.5, CO, NO(2), and O(3) on the Incidence and Mortality of SARS-CoV-2 Infection in Five Regions of the USA. Int J Environ Res Public Health. 2021;18(15).
138	Menendez JA. Metformin and SARS-CoV-2: mechanistic lessons on air pollution to weather the cytokine/thrombotic storm in COVID-19. Aging (Albany NY). 2020;12(10):8760-5.
139	Mendy A, Wu X, Keller JL, Fassler CS, Apewokin S, Mersha TB, et al. Air pollution and the pandemic: Long-term PM(2.5) exposure and disease severity in COVID-19 patients. Respirology. 2021;26(12):1181-7.
140	Mendy A, Wu X, Keller JL, Fassler CS, Apewokin S, Mersha TB, et al. Long-term exposure to fine particulate matter and hospitalization in COVID-19 patients. Respir Med. 2021;178:106313.
141	Mendoza DL, Benney TM, Bares R, Crosman ET. Intra-city variability of fine particulate matter during COVID-19 lockdown: A case study from Park City, Utah. Environ Res. 2021;201:111471.
142	Mendez-Espinosa JF, Rojas NY, Vargas J, Pachón JE, Belalcazar LC, Ramírez O. Air quality variations in Northern South America during the COVID-19 lockdown. Sci Total Environ. 2020;749:141621.
143	Menchaca M, Pagone F, Erdal S. Comparison of positive SARS-CoV-2 incidence rate with environmental and socioeconomic factors in northern Illinois. Heliyon. 2021;7(8):e07806.
144	Mehmood K, Bao Y, Petropoulos GP, Abbas R, Abrar MM, Saifullah, et al. Investigating connections between COVID-19 pandemic, air pollution and community interventions for Pakistan employing geoinformation technologies. Chemosphere. 2021;272:129809.
145	Marwah M, Agrawala PK. COVID-19 lockdown and environmental pollution: an Indian multi-state investigation. Environ Monit Assess. 2022;194(2):49.
146	Martorell-Marugán J, Villatoro-García JA, García-Moreno A, López-Domínguez R, Requena F, Merelo JJ, et al. DatAC: A visual analytics platform to explore climate and air quality indicators associated with the COVID-19 pandemic in Spain. Sci Total Environ. 2021;750:141424.
147	Martins G, Gogola JL, Budni LH, Janegitz BC, Marcolino-Junior LH, Bergamini MF. 3D-printed electrode as a new platform for electrochemical immunosensors for virus detection. Anal Chim Acta. 2021;1147:30-7.
148	Marquès M, Rovira J, Nadal M, Domingo JL. Effects of air pollution on the potential transmission and mortality of COVID-19: A preliminary case-study in Tarragona Province (Catalonia, Spain). Environ Res. 2021;192:110315.
149	Marquès M, Domingo JL. Positive association between outdoor air pollution and the incidence and severity of COVID-19. A review of the recent scientific evidences. Environ Res. 2022;203:111930.
150	Marquès M, Correig E, Ibarretxe D, Anoro E, Antonio Arroyo J, Jericó C, et al. Long-term exposure to PM(10) above WHO guidelines exacerbates COVID-19 severity and mortality. Environ Int. 2022;158:106930.

151	Manivannan J, Sundaresan L. Systems level insights into the impact of airborne exposure on SARS-CoV-2 pathogenesis and COVID-19 outcome - A multi-omics big data study. Gene Rep. 2021;25:101312.
152	Maleki M, Anvari E, Hopke PK, Noorimotlagh Z, Mirzaee SA. An updated systematic review on the association between atmospheric particulate matter pollution and prevalence of SARS-CoV-2. Environ Res. 2021;195:110898.
153	Maestre JP, Jarma D, Yu JF, Siegel JA, Horner SD, Kinney KA. Distribution of SARS-CoV-2 RNA signal in a home with COVID-19 positive occupants. Sci Total Environ. 2021;778:146201.
154	Macias-Verde D, Lara PC, Burgos-Burgos J. Same pollution sources for climate change might be hyperactivating the NLRP3 inflammasome and exacerbating neuroinflammation and SARS mortality. Med Hypotheses. 2021;146:110396.
155	Ma Q, Qi Y, Shan Q, Liu S, He H. Understanding the knowledge gaps between air pollution controls and health impacts including pathogen epidemic. Environ Res. 2020;189:109949.
156	Lym Y, Kim KJ. Exploring the effects of PM(2.5) and temperature on COVID-19 transmission in Seoul, South Korea. Environ Res. 2022;203:111810.
157	Lu B, Wu N, Jiang J, Li X. Associations of acute exposure to airborne pollutants with COVID-19 infection: evidence from China. Environ Sci Pollut Res Int. 2021;28(36):50554-64.
158	Lovrić M, Pavlović K, Vuković M, Grange SK, Haberl M, Kern R. Understanding the true effects of the COVID-19 lockdown on air pollution by means of machine learning. Environ Pollut. 2021;274:115900.
159	López-Feldman A, Heres D, Marquez-Padilla F. Air pollution exposure and COVID-19: A look at mortality in Mexico City using individual-level data. Sci Total Environ. 2021;756:143929.
160	López JH, Romo Á S, Molina DC, Hernández G, Cureño Á BG, Acosta MA, et al. Detection of Sars-Cov-2 in the air of two hospitals in Hermosillo, Sonora, México, utilizing a low-cost environmental monitoring system. Int J Infect Dis. 2021;102:478-82.
161	López A, Fuentes E, Yusà V, López-Labrador FX, Camaró M, Peris-Martinez C, et al. Indoor Air Quality including Respiratory Viruses. Toxics. 2021;9(11).
162	Litchfield IJ, Ayres JG, Jaakkola JJK, Mohammed NI. Is ambient air pollution associated with onset of sudden infant death syndrome: a case-crossover study in the UK. BMJ Open. 2018;8(4):e018341.
163	Linillos-Pradillo B, Rancan L, Ramiro ED, Vara E, Artíñano B, Arias J. Determination of SARS-CoV-2 RNA in different particulate matter size fractions of outdoor air samples in Madrid during the lockdown. Environ Res. 2021;195:110863.
164	Lin S, Wei D, Sun Y, Chen K, Yang L, Liu B, et al. Region-specific air pollutants and meteorological parameters influence COVID-19: A study from mainland China. Ecotoxicol Environ Saf. 2020;204:111035.
165	Li HH, Liu CC, Hsu TW, Lin JH, Hsu JW, Li AF, et al. Upregulation of ACE2 and TMPRSS2 by particulate matter and idiopathic pulmonary fibrosis: a potential role in severe COVID-19. Part Fibre Toxicol. 2021;18(1):11.
166	Li H, Xu XL, Dai DW, Huang ZY, Ma Z, Guan YJ. Air pollution and temperature are associated with increased COVID-19 incidence: A time series study. Int J Infect Dis. 2020;97:278-82.
167	Lepore E, Aguilera Benito P, Piña Ramírez C, Viccione G. Indoors ventilation in times of confinement by SARS-CoV-2 epidemic: A comparative approach between Spain and Italy. Sustain Cities Soc. 2021;72:103051.
168	Lembo R, Landoni G, Cianfanelli L, Frontera A. Air pollutants and SARS-CoV-2 in 33 European countries. Acta Biomed. 2021;92(1):e2021166.
169	Leão MLP, Penteado JO, Ulguim SM, Gabriel RR, Dos Santos M, Brum AN, et al. Health impact assessment of air pollutants during the COVID-19 pandemic in a Brazilian metropolis. Environ Sci Pollut Res Int. 2021;28(31):41843-50.
170	Laxmipriya S, Narayanan RM. COVID-19 and its relationship to particulate matter pollution - Case study from part of greater Chennai, India. Mater Today Proc. 2021;43:1634-9.
171	Lawrence RJ, O'Donoghue GM, Kitterick P, Hartley DEH. Use of a novel drape 'tent' as an infection prevention control measure for mastoid surgery. J Laryngol Otol. 2020;134(12):1115-7.
172	Land WG. Role of Damage-Associated Molecular Patterns in Light of Modern Environmental Research: A Tautological Approach. Int J Environ Res. 2020;14(5):583-604.
173	Lai AC, Poon CK, Cheung AC. Effectiveness of facemasks to reduce exposure hazards for airborne infections among general populations. J R Soc Interface. 2012;9(70):938-48.
174	Lai A, Chang ML, O'Donnell RP, Zhou C, Sumner JA, Hsiai TK. Association of COVID-19 transmission with high levels of ambient pollutants: Initiation and impact of the inflammatory response on cardiopulmonary disease. Sci Total Environ. 2021;779:146464.
175	Kyomba GK, Konde JNN, Saila-Ngita D, Solo TK, Kiyombo GM. Assessing the management of healthcare waste for disease prevention and environment protection at selected hospitals in Kinshasa, Democratic Republic of Congo. Waste Manag Res. 2021;39(10):1237-44.
176	Kumari P, Toshniwal D. Impact of lockdown on air quality over major cities across the globe during COVID-19 pandemic. Urban Clim. 2020;34:100719.
177	Kumar P, Hama S, Omidvarborna H, Sharma A, Sahani J, Abhijith KV, et al. Temporary reduction in fine particulate matter due to 'anthropogenic emissions switch-off' during COVID-19 lockdown in Indian cities. Sustain Cities Soc. 2020;62:102382.
178	Kudryashova OB, Muravlev EV, Antonnikova AA, Titov SS. Propagation of viral bioaerosols indoors. PLoS One. 2021;16(1):e0244983.
179	Konwar C, Asiimwe R, Inkster AM, Merrill SM, Negri GL, Aristizabal MJ, et al. Risk-focused differences in molecular processes implicated in SARS-CoV-2 infection: corollaries in DNA methylation and gene expression. Epigenetics Chromatin. 2021;14(1):54.
180	Kogevinas M, Castaño-Vinyals G, Karachaliou M, Espinosa A, de Cid R, Garcia-Aymerich J, et al. Ambient Air Pollution in Relation to SARS-CoV-2 Infection, Antibody Response, and COVID-19 Disease: A Cohort Study in Catalonia, Spain (COVICAT Study). Environ Health Perspect. 2021;129(11):117003.

181	Kiser D, Elhanan G, Metcalf WJ, Schnieder B, Grzymski JJ. SARS-CoV-2 test positivity rate in Reno, Nevada: association with PM2.5 during the 2020 wildfire smoke events in the western United States. J Expo Sci Environ Epidemiol. 2021;31(5):797-803.
182	Kim JH, Kim J, Kim WJ, Choi YH, Yang SR, Hong SH. Diesel Particulate Matter 2.5 Induces Epithelial-to-Mesenchymal Transition and Upregulation of SARS-CoV-2 Receptor during Human Pluripotent Stem Cell-Derived Alveolar Organoid Development. Int J Environ Res Public Health. 2020;17(22).
183	Khan YA. The COVID-19 pandemic and its impact on environment: the case of the major cities in Pakistan. Environ Sci Pollut Res Int. 2021;28(39):54728-43.
184	Khan TR, Parker DS, Withers C. Mitigation of Airborne Contaminant Spread through Simple Interventions in an Occupied Single-Family Home. Int J Environ Res Public Health. 2021;18(11).
185	Kerimray A, Baimatova N, Ibragimova OP, Bukenov B, Kenessov B, Plotitsyn P, et al. Assessing air quality changes in large cities during COVID-19 lockdowns: The impacts of traffic-free urban conditions in Almaty, Kazakhstan. Sci Total Environ. 2020;730:139179.
186	Kayalar Ö, Arı A, Babuççu G, Konyalılar N, Doğan Ö, Can F, et al. Existence of SARS-CoV-2 RNA on ambient particulate matter samples: A nationwide study in Turkey. Sci Total Environ. 2021;789:147976.
187	Katoto P, Brand AS, Bakan B, Obadia PM, Kuhangana C, Kayembe-Kitenge T, et al. Environ Health. 2021;20(1):41.
188	Kasloff SB, Leung A, Strong JE, Funk D, Cutts T. Stability of SARS-CoV-2 on critical personal protective equipment. Sci Rep. 2021;11(1):984.
189	Karan A, Ali K, Teelucksingh S, Sakhamuri S. The impact of air pollution on the incidence and mortality of COVID-19. Glob Health Res Policy. 2020;5:39.
190	Kan HD, Chen BH, Fu CW, Yu SZ, Mu LN. Relationship between ambient air pollution and daily mortality of SARS in Beijing. Biomed Environ Sci. 2005;18(1):1-4.
191	Jephcote C, Hansell AL, Adams K, Gulliver J. Changes in air quality during COVID-19 'lockdown' in the United Kingdom. Environ Pollut. 2021;272:116011.
192	Jakovljević I, Štrukil ZS, Godec R, Davila S, Pehnec G. Influence of lockdown caused by the COVID-19 pandemic on air pollution and carcinogenic content of particulate matter observed in Croatia. Air Qual Atmos Health. 2021;14(4):467-72.
193	Islam MS, Larpruenrudee P, Saha SC, Pourmehran O, Paul AR, Gemci T, et al. How severe acute respiratory syndrome coronavirus-2 aerosol propagates through the age-specific upper airways. Phys Fluids (1994). 2021;33(8):081911.
194	Ishmatov A. "SARS-CoV-2 is transmitted by particulate air pollution": Misinterpretations of statistical data, skewed citation practices, and misuse of specific terminology spreading the misconception. Environ Res. 2022;204(Pt B):112116.
195	In 't Veen J, Kappen JH, van Schayck OCP. [Air pollution: a determinant for COVID-19?]. Ned Tijdschr Geneeskd. 2020;164.
196	Ibarra-Espinosa S, Dias de Freitas E, Ropkins K, Dominici F, Rehbein A. Negative-Binomial and quasi-poisson regressions between COVID-19, mobility and environment in São Paulo, Brazil. Environ Res. 2022;204(Pt D):112369.
197	Huang W, Morawska L. Face masks could raise pollution risks. Nature. 2019;574(7776):29-30.
198	Huang KL, Chen CW, Chu SJ, Perng WC, Wu CP. Systemic inflammation caused by white smoke inhalation in a combat exercise. Chest. 2008;133(3):722-8.
199	Hokajärvi AM, Rytkönen A, Tiwari A, Kauppinen A, Oikarinen S, Lehto KM, et al. The detection and stability of the SARS-CoV-2 RNA biomarkers in wastewater influent in Helsinki, Finland. Sci Total Environ. 2021;770:145274.
200	Hill WC, Hull MS, MacCuspie RI. Testing of Commercial Masks and Respirators and Cotton Mask Insert Materials using SARS-CoV-2 Virion-Sized Particulates: Comparison of Ideal Aerosol Filtration Efficiency versus Fitted Filtration Efficiency. Nano Lett. 2020;20(10):7642-7.
201	He S, Han J. Electrostatic fine particles emitted from laser printers as potential vectors for airborne transmission of COVID-19. Environ Chem Lett. 2020:1-8.
202	Hao W, Wu J, Zhao X, Liang D, Yu X, Cao H, et al. Quantitative Evaluation of Aerosol Generation from Non-contact Tonometry and its Correlation with Tear Film Characteristics. Adv Ther. 2021;38(6):3066-76.
203	Hansell AL, Villeneuve PJ. Invited Perspective: Ambient Air Pollution and SARS-CoV-2: Research Challenges and Public Health Implications. Environ Health Perspect. 2021;129(11):111303.
204	Hakami AR, Dobie G. Studying the effect of particulate matter as SARS-CoV-2 transmitters. J Public Health Res. 2021;11(1).
205	Häfner SJ. This is not a pipe - But how harmful is electronic cigarette smoke. Biomed J. 2021;44(3):227-34.
206	Hadei M, Hopke PK, Shahsavani A, Raeisi A, Jafari AJ, Yarahmadi M, et al. Effect of short-term exposure to air pollution on COVID-19 mortality and morbidity in Iranian cities. J Environ Health Sci Eng. 2021;19(2):1-10.
207	Guo M, Xu P, Xiao T, He R, Dai M, Miller SL. Review and comparison of HVAC operation guidelines in different countries during the COVID-19 pandemic. Build Environ. 2021;187:107368.
208	Gujral H, Sinha A. Association between exposure to airborne pollutants and COVID-19 in Los Angeles, United States with ensemble-based dynamic emission model. Environ Res. 2021;194:110704.
209	Guibas GV, Makris M, Papadopoulos NG. Acute asthma exacerbations in childhood: risk factors, prevention and treatment. Expert Rev Respir Med. 2012;6(6):629-38.
210	Gregorio PHP, Mariani AW, Brito J, Santos BJM, Pêgo-Fernandes PM. Indoor Air Quality and Environmental Sampling as Support Tools to Detect SARS-CoV-2 in the Healthcare Setting. J Occup Environ Med. 2021;63(11):956-62.
211	Gotts JE, Chun L, Abbott J, Fang X, Takasaka N, Nishimura SL, et al. Cigarette smoke exposure worsens acute lung injury in antibiotic-treated bacterial pneumonia in mice. Am J Physiol Lung Cell Mol Physiol. 2018;315(1):L25-l40.
212	Gonçalves J, Koritnik T, Paragi M. Assessment of weather and atmospheric pollution as a co-factor in the spread of SARS-CoV-2. Acta Biomed. 2021;92(3):e2021094.
213	Gola M, Caggiano G, De Giglio O, Napoli C, Diella G, Carlucci M, et al. SARS-CoV-2 indoor contamination: considerations on anti-COVID-19 management of ventilation systems, and finishing materials in healthcare facilities. Ann Ig. 2021;33(4):381-92.

214	Gianquintieri L, Brovelli MA, Pagliosa A, Bonora R, Sechi GM, Caiani EG. Geospatial Correlation Analysis between Air Pollution Indicators and Estimated Speed of COVID-19 Diffusion in the Lombardy Region (Italy). Int J Environ Res Public Health. 2021;18(22).
215	Ghanim AAJ. Analyzing the severity of coronavirus infections in relation to air pollution: evidence-based study from Saudi Arabia. Environ Sci Pollut Res Int. 2022;29(4):6267-77.
216	Ghaffari HR, Farshidi H, Alipour V, Dindarloo K, Azad MH, Jamalidoust M, et al. Detection of SARS-CoV-2 in the indoor air of intensive care unit (ICU) for severe COVID-19 patients and its surroundings: considering the role of environmental conditions. Environ Sci Pollut Res Int. 2021:1-7.
217	Garbey M, Joerger G, Furr S. A Systems Approach to Assess Transport and Diffusion of Hazardous Airborne Particles in a Large Surgical Suite: Potential Impacts on Viral Airborne Transmission. Int J Environ Res Public Health. 2020;17(15).
218	Gallo O. Risk for COVID-19 infection in patients with tobacco smoke-associated cancers of the upper and lower airway. Eur Arch Otorhinolaryngol. 2021;278(8):2695-702.
219	Gallo M, Street ME, Guerra F, Fanos V, Marcialis MA. A review of current knowledge on Pollution, Cigarette Smoking and COVID-19 diffusion and their relationship with inflammation. Acta Biomed. 2020;91(4):e2020148.
220	Fronza R, Lusic M, Schmidt M, Lucic B. Spatial-Temporal Variations in Atmospheric Factors Contribute to SARS-CoV-2 Outbreak. Viruses. 2020;12(6).
221	Frontera A, Cianfanelli L, Vlachos K, Landoni G, Cremona G. Severe air pollution links to higher mortality in COVID-19 patients: The "double-hit" hypothesis. J Infect. 2020;81(2):255-9.
222	Fiorito S, Soligo M, Gao Y, Ogulur I, Akdis CA, Bonini S. Is epithelial barrier hypothesis the key to understanding the higher incidence and excess mortality during COVID-19 pandemic? The case of Northern Italy. Allergy. 2022.
223	Filippini T, Rothman KJ, Cocchio S, Narne E, Mantoan D, Saia M, et al. Associations between mortality from COVID-19 in two Italian regions and outdoor air pollution as assessed through tropospheric nitrogen dioxide. Sci Total Environ. 2021;760:143355.
224	Feng Z, Cao SJ, Wang J, Kumar P, Haghighat F. Indoor airborne disinfection with electrostatic disinfector (ESD): Numerical simulations of ESD performance and reduction of computing time. Build Environ. 2021;200:107956.
225	Feng S, Jia C, Liu Z, Lyu X. [Advances in the research of pathogenesis and treatment of severe smoke inhalation injury]. Zhonghua Shao Shang Za Zhi. 2016;32(2):122-5.
226	Félix-Arellano EE, Schilmann A, Hurtado-Díaz M, Texcalac-Sangrado JL, Riojas-Rodríguez H. [Quick review: air pollution and morbi-mortalityby Covid-19.]. Salud Publica Mex. 2020;62(5):582-9.
227	Fattorini D, Regoli F. Role of the chronic air pollution levels in the Covid-19 outbreak risk in Italy. Environ Pollut. 2020;264:114732.
228	Farhangrazi ZS, Sancini G, Hunter AC, Moghimi SM. Airborne Particulate Matter and SARS-CoV-2 Partnership: Virus Hitchhiking, Stabilization and Immune Cell Targeting - A Hypothesis. Front Immunol. 2020;11:579352.
229	Fang F, Mu L, Zhu Y, Rao J, Heymann J, Zhang ZF. Long-Term Exposure to PM(2.5), Facemask Mandates, Stay Home Orders and COVID-19 Incidence in the United States. Int J Environ Res Public Health. 2021;18(12).
230	Fabiani L, Saroglia M, Galatà G, De Santis R, Fillo S, Luca V, et al. Magnetic beads combined with carbon black-based screen-printed electrodes for COVID-19: A reliable and miniaturized electrochemical immunosensor for SARS-CoV-2 detection in saliva. Biosens Bioelectron. 2021;171:112686.
231	Espejo W, Celis JE, Chiang G, Bahamonde P. Environment and COVID-19: Pollutants, impacts, dissemination, management and recommendations for facing future epidemic threats. Sci Total Environ. 2020;747:141314.
232	Elsaie ML, Nada HA. Insights into laser safety considerations during COVID 19 pandemic. Dermatol Ther. 2020;33(5):e13777.
233	Edwards L, Rutter G, Iverson L, Wilson L, Chadha TS, Wilkinson P, et al. Personal exposure monitoring of PM(2.5) among US diplomats in Kathmandu during the COVID-19 lockdown, March to June 2020. Sci Total Environ. 2021;772:144836.
234	Duval JFL, van Leeuwen HP, Norde W, Town RM. Chemodynamic features of nanoparticles: Application to understanding the dynamic life cycle of SARS-CoV-2 in aerosols and aqueous biointerfacial zones. Adv Colloid Interface Sci. 2021;290:102400.
235	Dunker S, Hornick T, Szczepankiewicz G, Maier M, Bastl M, Bumberger J, et al. No SARS-CoV-2 detected in air samples (pollen and particulate matter) in Leipzig during the first spread. Sci Total Environ. 2021;755(Pt 1):142881.
236	Duffy C, Kidd A, Francis S, Tsim S, McNaughton L, Ferguson K, et al. Chest drain aerosol generation in COVID-19 and emission reduction using a simple anti-viral filter. BMJ Open Respir Res. 2020;7(1).
237	Dubey A, Kotnala G, Mandal TK, Sonkar SC, Singh VK, Guru SA, et al. Evidence of the presence of SARS-CoV-2 virus in atmospheric air and surfaces of a dedicated COVID hospital. J Med Virol. 2021;93(9):5339-49.
238	Dragone R, Licciardi G, Grasso G, Del Gaudio C, Chanussot J. Analysis of the Chemical and Physical Environmental Aspects that Promoted the Spread of SARS-CoV-2 in the Lombard Area. Int J Environ Res Public Health. 2021;18(3).
239	Dondi A, Betti L, Carbone C, Dormi A, Paglione M, Rinaldi M, et al. Understanding the environmental factors related to the decrease in Pediatric Emergency Department referrals for acute asthma during the SARS-CoV-2 pandemic. Pediatr Pulmonol. 2022;57(1):66-74.
240	Domínguez-Amarillo S, Fernández-Agüera J, Cesteros-García S, González-Lezcano RA. Bad Air Can Also Kill: Residential Indoor Air Quality and Pollutant Exposure Risk during the COVID-19 Crisis. Int J Environ Res Public Health. 2020;17(19).
241	Domingo JL, Rovira J. Effects of air pollutants on the transmission and severity of respiratory viral infections. Environ Res. 2020;187:109650.
242	Domingo JL, Marquès M, Rovira J. Influence of airborne transmission of SARS-CoV-2 on COVID-19 pandemic. A review. Environ Res. 2020;188:109861.
243	Doggett N, Chow CW, Mubareka S. Characterization of Experimental and Clinical Bioaerosol Generation During Potential Aerosol-Generating Procedures. Chest. 2020;158(6):2467-73.

244	Din AR, Hindocha A, Patel T, Sudarshan S, Cagney N, Koched A, et al. Quantitative analysis of particulate matter release during orthodontic procedures: a pilot study. Br Dent J. 2020:1-7.
245	Di Ciaula A, Bonfrate L, Portincasa P, Appice C, Belfiore A, Binetti M, et al. Nitrogen dioxide pollution increases vulnerability to COVID-19 through altered immune function. Environ Sci Pollut Res Int. 2022.
246	Di Cerbo A. Air pollution and SARS-CoV-2 in the Po Valley: possible environmental persistence? Minerva Med. 2020;111(4):306-7.
247	Dey P, Saha SK, Sarkar S. Study of the interactions of sneezing droplets with particulate matter in a polluted environment. Phys Fluids (1994). 2021;33(11):113310.
248	Dettori M, Deiana G, Balletto G, Borruso G, Murgante B, Arghittu A, et al. Air pollutants and risk of death due to COVID-19 in Italy. Environ Res. 2021;192:110459.
249	Derrick JL, Gomersall CD. Surgical helmets and SARS infection. Emerg Infect Dis. 2004;10(2):277-9.
250	Delikhoon M, Guzman MI, Nabizadeh R, Norouzian Baghani A. Modes of Transmission of Severe Acute Respiratory Syndrome-Coronavirus-2 (SARS-CoV-2) and Factors Influencing on the Airborne Transmission: A Review. Int J Environ Res Public Health. 2021;18(2).
251	de Rooij MMT, Hakze-Van der Honing RW, Hulst MM, Harders F, Engelsma M, van de Hoef W, et al. Occupational and environmental exposure to SARS-CoV-2 in and around infected mink farms. Occup Environ Med. 2021;78(12):893-9.
252	De Matteis S, Forastiere F, Baldacci S, Maio S, Tagliaferro S, Fasola S, et al. Issue 1 - "Update on adverse respiratory effects of outdoor air pollution". Part 1): Outdoor air pollution and respiratory diseases: A general update and an Italian perspective. Pulmonology. 2022.
253	Dave GS, Rakholiya KD, Kaneria MJ, Galvadiya BP, Vyas SR, Kanbi VH, et al. High affinity interaction of Solanum tuberosum and Brassica juncea residue smoke water compounds with proteins involved in coronavirus infection. Phytother Res. 2020;34(12):3400-10.
254	Datta M, Singh DD, Naqvi AR. Molecular Diagnostic Tools for the Detection of SARS-CoV-2. Int Rev Immunol. 2021;40(1-2):143-56.
255	Das D, Ramachandran G. Risk analysis of different transport vehicles in India during COVID-19 pandemic. Environ Res. 2021;199:111268.
256	Das A, Mitra S, Kumar S, Sengupta A. Two-drape closed pocket technique: minimizing aerosolization in mastoid exploration during COVID-19 pandemic. Eur Arch Otorhinolaryngol. 2020;277(12):3529-32.
257	Daoud AK, Hall JK, Petrick H, Strong A, Piggott C. The Potential for Cloth Masks to Protect Health Care Clinicians From SARS-CoV-2: A Rapid Review. Ann Fam Med. 2021;19(1):55-62.
258	da Costa KM, Saxena AK. Coronavirus disease 2019 pandemic and identifying insufflators with desufflation mode and surgical smoke evacuators for safe CO(2) removal. Asian J Endosc Surg. 2021;14(2):165-9.
259	Czwojdzińska M, Terpińska M, Kuźniarski A, Płaczkowska S, Piwowar A. Exposure to PM2.5 and PM10 and COVID-19 infection rates and mortality: A one-year observational study in Poland. Biomed J. 2021.
260	Curtis L. PM(2.5), NO(2), wildfires, and other environmental exposures are linked to higher Covid 19 incidence, severity, and death rates. Environ Sci Pollut Res Int. 2021;28(39):54429-47.
261	Cui Y, Zhang ZF, Froines J, Zhao J, Wang H, Yu SZ, et al. Air pollution and case fatality of SARS in the People's Republic of China: an ecologic study. Environ Health. 2003;2(1):15.
262	Cruz R, Lima-Silva AE, Bertuzzi R, Hoinaski L. Exercising under particulate matter exposure: Providing theoretical support for lung deposition and its relationship with COVID-19. Environ Res. 2021;202:111755.
263	Crane-Godreau MA, Clem KJ, Payne P, Fiering S. Vitamin D Deficiency and Air Pollution Exacerbate COVID-19 Through Suppression of Antiviral Peptide LL37. Front Public Health. 2020;8:232.
264	Cortes-Ramirez J, Michael RN, Knibbs LD, Bambrick H, Haswell MR, Wraith D. The association of wildfire air pollution with COVID-19 incidence in New South Wales, Australia. Sci Total Environ. 2022;809:151158.
265	Copat C, Cristaldi A, Fiore M, Grasso A, Zuccarello P, Signorelli SS, et al. The role of air pollution (PM and NO(2)) in COVID-19 spread and lethality: A systematic review. Environ Res. 2020;191:110129.
266	Comunian S, Dongo D, Milani C, Palestini P. Air Pollution and Covid-19: The Role of Particulate Matter in the Spread and Increase of Covid-19's Morbidity and Mortality. Int J Environ Res Public Health. 2020;17(12).
267	Collivignarelli MC, Abbà A, Caccamo FM, Bertanza G, Pedrazzani R, Baldi M, et al. Can particulate matter be identified as the primary cause of the rapid spread of CoViD-19 in some areas of Northern Italy? Environ Sci Pollut Res Int. 2021;28(25):33120-32.
268	Collivignarelli MC, Abbà A, Bertanza G, Pedrazzani R, Ricciardi P, Carnevale Miino M. Lockdown for CoViD-2019 in Milan: What are the effects on air quality? Sci Total Environ. 2020;732:139280.
269	Colacci A, Bortone G, Maffei G, Marchesi S, Mescoli A, Parmagnani F, et al. Environmental pollution and COVID-19: the molecular terms and predominant disease outcomes of their sweetheart agreement. Epidemiol Prev. 2020;44(5-6 Suppl 2):169-82.
270	Coker ES, Cavalli L, Fabrizi E, Guastella G, Lippo E, Parisi ML, et al. The Effects of Air Pollution on COVID-19 Related Mortality in Northern Italy. Environ Resour Econ (Dordr). 2020;76(4):611-34.
271	Coccia M. Effects of the spread of COVID-19 on public health of polluted cities: results of the first wave for explaining the dejà vu in the second wave of COVID-19 pandemic and epidemics of future vital agents. Environ Sci Pollut Res Int. 2021;28(15):19147-54.
272	Coccia M. How do low wind speeds and high levels of air pollution support the spread of COVID-19? Atmos Pollut Res. 2021;12(1):437-45.
273	Coccia M. Factors determining the diffusion of COVID-19 and suggested strategy to prevent future accelerated viral infectivity similar to COVID. Sci Total Environ. 2020;729:138474.

274	Ciglenečki I, Orlović-Leko P, Vidović K, Tasić V. The possible role of the surface active substances (SAS) in the airborne transmission of SARS-CoV-2. Environ Res. 2021;198:111215.
275	Cicuttin E, Cobianchi L, Chiarugi M, Catena F, Coccolini F, Pietrabissa A. Detect to protect: pneumoperitoneum gas samples for SARS-CoV-2 and biohazard testing. Surg Endosc. 2020;34(7):2863-5.
276	Chuang HC, Chen YY, Hsiao TC, Chou HC, Kuo HP, Feng PH, et al. Alteration in angiotensin-converting enzyme 2 by PM(1) during the development of emphysema in rats. ERJ Open Res. 2020;6(4).
277	Christophi CA, Sotos-Prieto M, Lan FY, Delgado-Velandia M, Efthymiou V, Gaviola GC, et al. Ambient temperature and subsequent COVID-19 mortality in the OECD countries and individual United States. Sci Rep. 2021;11(1):8710.
278	Christopherson DA, Yao WC, Lu M, Vijayakumar R, Sedaghat AR. High-Efficiency Particulate Air Filters in the Era of COVID-19: Function and Efficacy. Otolaryngol Head Neck Surg. 2020;163(6):1153-5.
279	Chow TT, Kwan A, Lin Z, Bai W. Conversion of operating theatre from positive to negative pressure environment. J Hosp Infect. 2006;64(4):371-8.
280	Chiu YJ, Ma H, Liao WC, Shih YC, Chen MC, Shih CC, et al. Extracorporeal membrane oxygenation support may be a lifesaving modality in patients with burn and severe acute respiratory distress syndrome: Experience of Formosa Water Park dust explosion disaster in Taiwan. Burns. 2018;44(1):118-23.
281	Chia PY, Coleman KK, Tan YK, Ong SWX, Gum M, Lau SK, et al. Detection of air and surface contamination by SARS-CoV-2 in hospital rooms of infected patients. Nat Commun. 2020;11(1):2800.
282	Cheruiyot I, Sehmi P, Ngure B, Misiani M, Karau P, Olabu B, et al. Laparoscopic surgery during the COVID-19 pandemic: detection of SARS-COV-2 in abdominal tissues, fluids, and surgical smoke. Langenbecks Arch Surg. 2021;406(4):1007-14.
283	Chennakesavulu K, Reddy GR. The effect of latitude and PM(2.5) on spreading of SARS-CoV-2 in tropical and temperate zone countries. Environ Pollut. 2020;266(Pt 3):115176.
284	Chen Y, Zhang S, Peng C, Shi G, Tian M, Huang RJ, et al. Impact of the COVID-19 pandemic and control measures on air quality and aerosol light absorption in Southwestern China. Sci Total Environ. 2020;749:141419.
285	Chen B, Jia P, Han J. Role of indoor aerosols for COVID-19 viral transmission: a review. Environ Chem Lett. 2021;19(3):1953-70.
286	Chakraborty S, Dey T, Jun Y, Lim CY, Mukherjee A, Dominici F. A Spatiotemporal Analytical Outlook of the Exposure to Air Pollution and COVID-19 Mortality in the USA. J Agric Biol Environ Stat. 2022:1-21.
287	Chadeau-Hyam M, Bodinier B, Elliott J, Whitaker MD, Tzoulaki I, Vermeulen R, et al. Risk factors for positive and negative COVID-19 tests: a cautious and in-depth analysis of UK biobank data. Int J Epidemiol. 2020;49(5):1454-67.
288	Cazzolla Gatti R, Velichevskaya A, Tateo A, Amoroso N, Monaco A. Machine learning reveals that prolonged exposure to air pollution is associated with SARS-CoV-2 mortality and infectivity in Italy. Environ Pollut. 2020;267:115471.
289	Caseiro A, von Schneidemesser E. APExpose_DE, an air quality exposure dataset for Germany 2010-2019. Sci Data. 2021;8(1):287.
290	Calfee CS, Matthay MA, Eisner MD, Benowitz N, Call M, Pittet JF, et al. Active and passive cigarette smoking and acute lung injury after severe blunt trauma. Am J Respir Crit Care Med. 2011;183(12):1660-5.
291	Cahyadi MN, Handayani HH, Warmadewanthi I, Rokhmana CA, Sulistiawan SS, Waloedjo CS, et al. Spatiotemporal Analysis for COVID-19 Delta Variant Using GIS-Based Air Parameter and Spatial Modeling. Int J Environ Res Public Health. 2022;19(3).
292	Buonanno M, Welch D, Shuryak I, Brenner DJ. Far-UVC light (222 nm) efficiently and safely inactivates airborne human coronaviruses. Sci Rep. 2020;10(1):10285.
293	Bui TT, Shin MK, Jee SY, Long DX, Hong J, Kim MG. Ferroelectric PVDF nanofiber membrane for high-efficiency PM0.3 air filtration with low air flow resistance. Colloids Surf A Physicochem Eng Asp. 2022;640:128418.
294	Bryant J, Tobias JD. Enclosure with augmented airflow to decrease risk of exposure to aerosolized pathogens including coronavirus during endotracheal intubation. Can the reduction in aerosolized particles be quantified? Paediatr Anaesth. 2020;30(8):900-4.
295	Brocke SA, Billings GT, Taft-Benz S, Alexis NE, Heise MT, Jaspers I. Woodsmoke particle exposure prior to SARS-CoV-2 infection alters antiviral response gene expression in human nasal epithelial cells in a sex-dependent manner. Am J Physiol Lung Cell Mol Physiol. 2022.
296	Briz-Redón Á, Belenguer-Sapiña C, Serrano-Aroca Á. A city-level analysis of PM(2.5) pollution, climate and COVID-19 early spread in Spain. J Environ Health Sci Eng. 2022:1-9.
297	Briz-Redón Á, Belenguer-Sapiña C, Serrano-Aroca Á. Changes in air pollution during COVID-19 lockdown in Spain: A multi-city study. J Environ Sci (China). 2021;101:16-26.
298	Brégeon F, Papazian L, Delpierre S, Kajikawa O, Payan MJ, Martin TR, et al. Role of proinflammatory activity contained in gastric juice from intensive care unit patients to induce lung injury in a rabbit aspiration model. Crit Care Med. 2008;36(12):3205-12.
299	Brandt EB, Mersha TB. Environmental Determinants of Coronavirus Disease 2019 (COVID-19). Curr Allergy Asthma Rep. 2021;21(3):15.
300	Bozack A, Pierre S, DeFelice N, Colicino E, Jack D, Chillrud SN, et al. Long-Term Air Pollution Exposure and COVID-19 Mortality: A Patient-Level Analysis from New York City. Am J Respir Crit Care Med. 2021.
301	Bostanci Ceran B, Karakoç A, Taciroğlu E. Airborne pathogen projection during ophthalmic examination. Graefes Arch Clin Exp Ophthalmol. 2020;258(10):2275-82.
302	Bossak BH, Andritsch S. COVID-19 and Air Pollution: A Spatial Analysis of Particulate Matter Concentration and Pandemic-Associated Mortality in the US. Int J Environ Res Public Health. 2022;19(1).
303	Borro M, Di Girolamo P, Gentile G, De Luca O, Preissner R, Marcolongo A, et al. Evidence-Based Considerations Exploring Relations between SARS-CoV-2 Pandemic and Air Pollution: Involvement of PM2.5-Mediated Up-Regulation of the Viral Receptor ACE-2. Int J Environ Res Public Health. 2020;17(15).

304	Borisova T, Komisarenko S. Air pollution particulate matter as a potential carrier of SARS-CoV-2 to the nervous system and/or neurological symptom enhancer: arguments in favor. Environ Sci Pollut Res Int. 2021;28(30):40371-7.
305	Borak J. Airborne Transmission of COVID-19. Occup Med (Lond). 2020;70(5):297-9.
306	Bogani G, Ditto A, De Cecco L, Lopez S, Guerrisi R, Piccioni F, et al. Transmission of SARS-CoV-2 in Surgical Smoke during Laparoscopy: A Prospective, Proof-of-concept Study. J Minim Invasive Gynecol. 2021;28(8):1519-25.
307	Bianconi V, Bronzo P, Banach M, Sahebkar A, Mannarino MR, Pirro M. Particulate matter pollution and the COVID-19 outbreak: results from Italian regions and provinces. Arch Med Sci. 2020;16(5):985-92.
308	Bherwani H, Gautam S, Gupta A. Qualitative and quantitative analyses of impact of COVID-19 on sustainable development goals (SDGs) in Indian subcontinent with a focus on air quality. Int J Environ Sci Technol (Tehran). 2021:1-10.
309	Berg K, Romer Present P, Richardson K. Long-term air pollution and other risk factors associated with COVID-19 at the census tract level in Colorado. Environ Pollut. 2021;287:117584.
310	Belosi F, Conte M, Gianelle V, Santachiara G, Contini D. On the concentration of SARS-CoV-2 in outdoor air and the interaction with pre-existing atmospheric particles. Environ Res. 2021;193:110603.
311	Beig G, Korhale N, Rathod A, Maji S, Sahu SK, Dole S, et al. On modelling growing menace of household emissions under COVID-19 in Indian metros. Environ Pollut. 2021;272:115993.
312	Baron YM. Could changes in the airborne pollutant particulate matter acting as a viral vector have exerted selective pressure to cause COVID-19 evolution? Med Hypotheses. 2021;146:110401.
313	Baron YM. Are there medium to short-term multifaceted effects of the airborne pollutant PM(2.5) determining the emergence of SARS-CoV-2 variants? Med Hypotheses. 2021;158:110718.
314	Barakat T, Muylkens B, Su BL. Is Particulate Matter of Air Pollution a Vector of Covid-19 Pandemic? Matter. 2020;3(4):977-80.
315	Banik RK, Ulrich A. Evidence of Short-Range Aerosol Transmission of SARS-CoV-2 and Call for Universal Airborne Precautions for Anesthesiologists During the COVID-19 Pandemic. Anesth Analg. 2020;131(2):e102-e4.
316	Azuma K, Kagi N, Kim H, Hayashi M. Impact of climate and ambient air pollution on the epidemic growth during COVID-19 outbreak in Japan. Environ Res. 2020;190:110042.
317	Aykaç N, Etiler N. COVID-19 mortality in Istanbul in association with air pollution and socioeconomic status: an ecological study. Environ Sci Pollut Res Int. 2022;29(9):13700-8.
318	Atiyani R, Mustafa S, Alsari S, Darwish A, Janahi EM. Clearing the air about airborne transmission of SARS-CoV-2. Eur Rev Med Pharmacol Sci. 2021;25(21):6745-66.
319	Anser MK, Godil DI, Khan MA, Nassani AA, Zaman K, Abro MMQ. The impact of coal combustion, nitrous oxide emissions, and traffic emissions on COVID-19 cases: a Markov-switching approach. Environ Sci Pollut Res Int. 2021;28(45):64882-91.
320	Anil I, Alagha O. The impact of COVID-19 lockdown on the air quality of Eastern Province, Saudi Arabia. Air Qual Atmos Health. 2021;14(1):117-28.
321	Anil I, Alagha O. Source Apportionment of Ambient Black Carbon During the COVID-19 Lockdown. Int J Environ Res Public Health. 2020;17(23).
322	Andersen ZJ, Hoffmann B, Morawska L, Adams M, Furman E, Yorgancioglu A, et al. Air pollution and COVID-19: clearing the air and charting a post-pandemic course: a joint workshop report of ERS, ISEE, HEI and WHO. Eur Respir J. 2021;58(2).
323	Anand U, Cabreros C, Mal J, Ballesteros F, Jr., Sillanpää M, Tripathi V, et al. Novel coronavirus disease 2019 (COVID-19) pandemic: From transmission to control with an interdisciplinary vision. Environ Res. 2021;197:111126.
324	Amoroso N, Cilli R, Maggipinto T, Monaco A, Tangaro S, Bellotti R. Satellite data and machine learning reveal a significant correlation between NO(2) and COVID-19 mortality. Environ Res. 2022;204(Pt A):111970.
325	Amoatey P, Omidvarborna H, Baawain MS, Al-Mamun A. Impact of building ventilation systems and habitual indoor incense burning on SARS-CoV-2 virus transmissions in Middle Eastern countries. Sci Total Environ. 2020;733:139356.
326	Alvarez AE, Marson FA, Bertuzzo CS, Arns CW, Ribeiro JD. Epidemiological and genetic characteristics associated with the severity of acute viral bronchiolitis by respiratory syncytial virus. J Pediatr (Rio J). 2013;89(6):531-43.
327	Aloufi N, Traboulsi H, Ding J, Fonseca GJ, Nair P, Huang SK, et al. Angiotensin-converting enzyme 2 expression in COPD and IPF fibroblasts: the forgotten cell in COVID-19. Am J Physiol Lung Cell Mol Physiol. 2021;320(1):L152-l7.
328	Ali SM, Malik F, Anjum MS, Siddiqui GF, Anwar MN, Lam SS, et al. Exploring the linkage between PM(2.5) levels and COVID-19 spread and its implications for socio-economic circles. Environ Res. 2021;193:110421.
329	Ali N, Fariha KA, Islam F, Mishu MA, Mohanto NC, Hosen MJ, et al. Exposure to air pollution and COVID-19 severity: A review of current insights, management, and challenges. Integr Environ Assess Manag. 2021;17(6):1114-22.
330	Aggarwal S, Balaji S, Singh T, Menon GR, Mandal S, Madhumathi J, et al. Association between ambient air pollutants and meteorological factors with SARS-CoV-2 transmission and mortality in India: an exploratory study. Environ Health. 2021;20(1):120.
331	Adams MD. Air pollution in Ontario, Canada during the COVID-19 State of Emergency. Sci Total Environ. 2020;742:140516.
332	Accarino G, Lorenzetti S, Aloisio G. Assessing correlations between short-term exposure to atmospheric pollutants and COVID-19 spread in all Italian territorial areas. Environ Pollut. 2021;268(Pt A):115714.
333	Abrams EM, Sinha I, Fernandes RM, Hawcutt DB. Pediatric asthma and COVID-19: The known, the unknown, and the controversial. Pediatr Pulmonol. 2020;55(12):3573-8.
334	[Not Available]. Recenti Prog Med. 2020;111(6):383-4.

Supplementary Table S3a: Manuscripts Excluded because they are Case reports/series	
1	Kallivoulos S, Giantzi V, Parissis D, Grigoriadis N. MRI-negative acute transverse myelitis during COVID-19 pandemic: A case report. European Journal of Neurology. 2021;28(SUPPL 1):590.
2	Yepez JB, Murati FA, Petitto M, De Yepez J, Galue JM, Revilla J, et al. Vogt-koyanagi-harada disease following COVID-19 infection. Case Reports in Ophthalmology. 2021;12(3):804-8.
3	Wang C, Xie J, Zhao L, Fei X, Zhang H, Tan Y, et al. Alveolar macrophage dysfunction and cytokine storm in the pathogenesis of two severe COVID-19 patients. EBioMedicine. 2020;57 (no pagination).
4	Versace V, Sebastianelli L, Ferrazzoli D, Saltuari L, Kofler M, Loscher W, et al. Case Report: Myopathy in Critically Ill COVID-19 Patients: A Consequence of Hyperinflammation? Frontiers in Neurology. 2021;12 (no pagination).
5	Szydelko-Pasko U, Przezdziecka-Dolyk J, Krecicka J, Malecki R, Misiuk-Hojlo M, Turno-Krecicka A. Arteritic Anterior Ischemic Optic Neuropathy in the Course of Giant Cell Arteritis After COVID-19. American Journal of Case Reports. 2022;23(1) (no pagination).
6	Soto Insuga V, Cantarin-Extremera V, Solis-Munoz I, Buendia-Martinez S, Atencia-Ballesteros M, Bernardino B, et al. Pseudotumor Cerebri Caused by SARS-CoV-2 Infection in a Boy. Journal of Pediatric Neurology. 2021;19(3):207-9.
7	Sharma A, Kudchadkar US, Shirodkar R, Usgaonkar UPS, Naik A. Unilateral inferior altitudinal visual field defect related to COVID-19. Indian journal of ophthalmology. 2021;69(4):989-91.
8	Salazar A, Gonzalez A, Murray NP, Castro C. Atypical presentation of COVID-19: Chronic bilateral testicular pain with lower extremity peripheral polyneuropathy, case report. Urology Case Reports. 2022;40 (no pagination).
9	Roa Gomez G, Diaz-Fuentes G, Venkatram S. Fiber-Optic Bronchoscopy in Patients Infected with Covid-19: A Case Series. Chest. 2020;158(4 Supplement):A1943.
10	Pednekar P, Amoah K, homer R, Ryu C, Lutchmansingh D. Bullous Lung Disease Following Covid-19 Infection. Chest. 2021;160(4 Supplement):A356.
11	Nihei Y, Nagasawa H, Fukao Y, Kihara M, Ueda S, Gohda T, et al. Continuous extracorporeal treatments in a dialysis patient with COVID-19. CEN Case Reports. 2021;10(2):172-7.
12	Moschetta L, Fasolino G, Kuijpers RW. Non-arteritic anterior ischaemic optic neuropathy sequential to SARS-CoV-2 virus pneumonia: Preventable by endothelial protection? BMJ Case Reports. 2021;14(7) (no pagination).
13	Moreno-Duarte I, Evans AS, Alder AC, Vernon MC, Szmuk P, Rebstock S. An unexpected COVID-19 diagnosis during emergency surgery in a neonate. Paediatric Anaesthesia. 2021;31(5):613-5.
14	Li Y, Wu J, Wang S, Li X, Zhou J, Huang B, et al. Progression to fibrosing diffuse alveolar damage in a series of 30 minimally invasive autopsies with COVID-19 pneumonia in Wuhan, China. Histopathology. 2021;78(4):542-55.
15	Kusaba Y, Izumi S, Takasaki J, Suzuki M, Katagiri D, Katsuno T, et al. Successful recovery from COVID-19-associated acute respiratory failure with polymyxin B-immobilized fiber column-direct hemoperfusion. Internal Medicine. 2020;59(19):2405-8.
16	Krawitz BD, Sirinek P, Doobin D, Nanda T, Ghiassi M, Horowitz JD, et al. The Challenge of Managing Bilateral Acute Angle-closure Glaucoma in the Presence of Active SARS-CoV-2 Infection. Journal of Glaucoma. 2021;30(3):e50-e3.
17	Kim D, Ko JH, Peck KR, Baek JY, Moon HW, Ki HK, et al. A covid-19 exposure at a dental clinic where healthcare workers routinely use particulate filtering respirators. International Journal of Environmental Research and Public Health. 2021;18(12) (no pagination).
18	Jakubowski B, Mehta R. A Unique Case of COVID Pneumonia, Tuberculosis, and Silicosis. American Journal of Respiratory and Critical Care Medicine Conference: American Thoracic Society International Conference, ATS. 2021;203(9).
19	Girbardt C, Busch C, Al-Sheikh M, Gunzinger JM, Invernizzi A, Xhepa A, et al. Retinal vascular events after mrna and adenoviral-vectored covid-19 vaccines-a case series. Vaccines. 2021;9(11) (no pagination).
20	El-Zein RS, Cardinali S, Murphy C, Keeling T. COVID-19-associated meningoencephalitis treated with intravenous immunoglobulin. BMJ Case Reports. 2020;13(9) (no pagination).
21	Demir E, Bilen S. Ocular myasthenia graves in a patient with COVID-19; neurological overview of autoimmune complications of COVID-19. Journal of the Neurological Sciences. 2021;Conference: World Congress of Neurology(WCN 2021 . Rome Italy. 429 Supplement).
22	De Giglio L, Sadun F, Roberti C, Polidori L, Gilardi M, Altavista MC, et al. Post-COVID simultaneous onset of Graves' disease and ocular myasthenia gravis in a patient with a complex ocular motility impairment. European Journal of Ophthalmology. 2022.
23	Cilhoroz BT, DeRuisseau LR. Safety protocols in an exercise facility result in no detectable sars-CoV2 spread: A case study. Physiological Reports. 2021;9(14) (no pagination).
24	Carey M, Waheed W. New-onset dysesthesias following COVID-19 inoculation. Annals of Neurology. 2021;90(SUPPL 27):S200.
25	Amundson DE, Shah US, de Necochea-Campion R, Jacobs M, LaRosa SP, Fisher CJ. Removal of COVID-19 Spike Protein, Whole Virus, Exosomes, and Exosomal MicroRNAs by the Hemopurifier Lectin-Affinity Cartridge in Critically Ill Patients With COVID-19 Infection. Frontiers in Medicine. 2021;8 (no pagination).
26	Ali A, Mathew R, Jadeja S, Schey R, Masri G. Small intestinal bacterial overgrowth after COVID-19 infection. American Journal of Gastroenterology. 2021;116(SUPPL):S1243-S4.
27	Akgun Y, Wu Y. Plasma exchange for acute motor axonal neuropathy variant guillain-barre syndrome in a pregnant woman with active systemic lupus erythematosus. Journal of Clinical Apheresis. 2021;36(2):269-70.
28	Agnihotri SP, Luis CVS, Kazamel M. Autonomic neuropathy as post-acute sequela of SARS-CoV-2 infection: a case report. Journal of NeuroVirology. 2022.

Supplementary Table S3b: Manuscripts Excluded because they are Commentary/Expert opinion/ Conference Abstracts	
1	Young IR, Cowman MK, Kirsch T, Crowley G, Nolan A. COVID-19 and pm exposure: Identifying and mitigating the synergistic deleterious effects. American Journal of Respiratory and Critical Care Medicine Conference: American Thoracic Society International Conference, ATS. 2021;203(9).
2	Vuorio A, Budowle B, Kovanen PT. Airborne particles and cardiovascular morbidity in severe inherited hypercholesterolemia: Vulnerable endothelium under multiple attacks. BioEssays. 2021.
3	Veronesi G, De Matteis S, Calori G, Pepe N, Ferrario M. Long term exposure to air pollution and COVID-19 incidence in the city of Varese, Northern Italy: A complete-year, individual-level analysis. Occupational and Environmental Medicine. 2021;78(SUPPL 1):A85-A6.
4	Urrutia-Pereira M, Mello-da-Silva CA, Sole D. COVID-19 and air pollution: A dangerous association? Allergologia et Immunopathologia. 2020;48(5):496-9.
5	Szeto CH. Ways to tackle the highly transmissible delta virus. Respirology. 2021;26(SUPPL 3):122-3.
6	Stuempfig ND, Nadir NA. A comparison of intubation barrier devices. Academic Emergency Medicine. 2021;28(SUPPL 1):S373-S4.
7	Stout S, Pandya A, Murphy H, Yeh HW, Portnoy J. Factors Leading to Reduced Unscheduled Pediatric Asthma Visits During COVID-19. Journal of Allergy and Clinical Immunology. 2022;149(2 Supplement):AB183.
8	Stern R, Koutrakis P, Martins M, Lemos B, Dowd S, Sunderland E, et al. Characterization of airborne sars-cov-2 in a veterans affairs medical center. American Journal of Respiratory and Critical Care Medicine Conference: American Thoracic Society International Conference, ATS. 2021;203(9).
9	Sitovskaia D, Zabrodskaia Y, Verbitskiy O, Petrova Y, Sokolova T. Morphological changes in the spinal cord tissue in patients with Guillain-Barre syndrome associated with COVID-19. Journal of the Neurological Sciences. 2021;Conference: World Congress of Neurology(WCN 2021 . Rome Italy. 429 Supplement).
10	Sharma AK, Balyan P. Air pollution and COVID-19: Is the connect worth its weight? Indian journal of public health. 2020;64(Supplemententement):S132-S4.
11	Seto WH, Conly J, Cookson B, Pittet D, Holmes A, Chu M, et al. Use of medical face masks versus particulate respirators as a component of personal protective equipment for health care workers in the context of the COVID-19 pandemic. Antimicrobial Resistance and Infection Control. 2020;9(1) (no pagination).
12	Recchioni A, Makanvand M, Wallace GR, Poonit N, Bloss W, Rauz S. Impact of the United Kingdom (UK) COVID-19 lockdown measures on air pollution and ocular surface disease symptomatology amongst shielding patients. Investigative Ophthalmology and Visual Science Conference: Annual Meeting Association for Research in Vision and Ophthalmology, ARVO. 2021;62(8).
13	Popov T, Josling P, DuBuske L. Regular Use of Nasally Applied Methyl-cellulose Powder During the Pollen Season Protects Against Sars-Covid-2 Infections. Journal of Allergy and Clinical Immunology. 2022;149(2 Supplement):AB100.
14	Ponzano M, Bergamaschi R, Pisoni E, De Rossi N, Schiavetti I, Carmisciano L, et al. The impact of PM2.5 on COVID-19 severity among Italian MS patients. Multiple Sclerosis Journal. 2021;27(2 SUPPL):369-70.
15	Perez J, Santillana C, Corrales O, Gonzalez D, Lacaille S, Sitahal R. An Unusual Variant of Guillain- Barre Syndrome with Covid-19. Chest. 2021;160(4 Supplement):A2344.
16	Pereira A. Long-Term Neurological Threats of COVID-19: A Call to Update the Thinking About the Outcomes of the Coronavirus Pandemic. Frontiers in Neurology. 2020;11 (no pagination).
17	Moccia F, Gerbino A, Lionetti V, Miragoli M, Munaron LM, Pagliaro P, et al. COVID-19-associated cardiovascular morbidity in older adults: a position paper from the Italian Society of Cardiovascular Researches. GeroScience. 2020;42(4):1021-49.
18	Miyashita L, Foley G, Grigg J. Exposure to Particulate Matter Increases Expression of the Angiotensin converting enzyme-2 (ACE2) Receptor. Journal of Allergy and Clinical Immunology. 2022;149(2 Supplement):AB30.
19	Leung C. Guillain-Barre syndrome should be monitored upon mass vaccination against SARS-CoV-2. Human Vaccines and Immunotherapeutics. 2021;17(9):2957-8.
20	Khallouli A, Lagneb C, Choura R, Saidane R, Gouider D, Maalej A, et al. Retinal microvascular impairment assessed by OCT-Angiography after SARS-CoV-2 infection. Ophthalmologica Conference: European Society of Retina Specialists Congress, EURETINA. 2021;244(SUPPL 1).
21	Ho CY, Salimian M, Holler J, Burke A, Ames H, Hegert J, et al. Mechanism of Olfactory Dysfunction in COVID-19 Infection. Journal of Neuropathology and Experimental Neurology. 2021;80(6):587.
22	Herghelegiu AM, Nuta CR, Bajenaru OL, Taranu SM, Ilie AC, Alexa ID, et al. COVID-19 and neurocognitive function. European Geriatric Medicine. 2021;12(SUPPL 1):S94-S5.
23	Heijink IH, Hackett TL, Pouwels SD. Effects of cigarette smoking on SARS-CoV-2 receptor ACE2 expression in the respiratory epithelium⁺. Journal of Pathology. 2021;253(4):351-4.
24	Farhangrazi ZS, Sancini G, Hunter AC, Moghimi SM. Airborne Particulate Matter and SARS-CoV-2 Partnership: Virus Hitchhiking, Stabilization and Immune Cell Targeting - A Hypothesis. Frontiers in Immunology. 2020;11 (no pagination).
25	De Vries JJ, Visser C, Geers L, Slotman JA, Endeman H, Kruip MJHA, et al. Does Fibrin Structure Contribute to the Increased Risk of Thrombosis in COVID-19 ICU Patients? Blood. 2021;138(Supplement 1):3208.
26	Christopherson DA, Yao WC, Lu M, Vijayakumar R, Sedaghat AR. High-Efficiency Particulate Air Filters in the Era of COVID-19: Function and Efficacy. Otolaryngology - Head and Neck Surgery (United States). 2020;163(6):1153-5.

27	Carugno M, Fedrizzi L, Borroni E, Consonni D, Pesatori AC. Air pollution exposure, SARS-CoV-2 infection, and immune response in a cohort of 3,700 healthcare workers. Safety and Health at Work. 2022;13(Supplement):S189-S90.
28	Batah SS, Benatti M, Syung L, Telini W, Capelozzi V, Cetlin A, et al. Respiratory disease, and treatment / thematic poster session COVID-19 outcome-based pathology reveals the birth of fibrosing interstitial pneumonitis. American Journal of Respiratory and Critical Care Medicine Conference: American Thoracic Society International Conference, ATS. 2021;203(9).
29	Aylward R, Bieber B, Guedes M, Pisoni R, Koranteng Tannor E, Dreyer G, et al. Pos-926 in-Centre Haemodialysis Centres Variably Affected by the Coronavirus-2019 Pandemic in Different Regions of the World: The International Society of Nephrology-Dialysis Outcomes Practice Patterns Study Survey. Kidney International Reports. 2022;7(2 Supplement):S404-S5.
30	Assini A, Gandoglia I, Damato V, Rikani K, Evoli A, Del Sette M. Response to: "MuSK-positive myasthenia may be triggered not only by SARS-CoV-2". European Journal of Neurology. 2021;28(10):e82-e3.
31	Alharbi M, Burstin H. Small fiber neuropathy pots and gottron sign is COVID19 the culprit. Journal of General Internal Medicine. 2021;36(SUPPL 1):S269.
32	Vuorio A, Budowle B, Kovanen PT. Airborne particles and cardiovascular morbidity in severe inherited hypercholesterolemia: Vulnerable endothelium under multiple attacks. Bioessays. 2022;44(3):e2100273.
33	Sinnige JS, Kooij FO, van Schuppen H, Hollmann MW, Sperna Weiland NH. Protection of healthcare workers during aerosol-generating procedures with local exhaust ventilation. Br J Anaesth. 2021;126(6):e220-e2.
34	Huang W, Morawska L. Face masks could raise pollution risks. Nature. 2019;574(7776):29-30.
35	Elsaie ML, Nada HA. Insights into laser safety considerations during COVID 19 pandemic. Dermatol Ther. 2020;33(5):e13777.
36	Andersen ZJ, Hoffmann B, Morawska L, Adams M, Furman E, Yorgancioglu A, et al. Air pollution and COVID-19: clearing the air and charting a post-pandemic course: a joint workshop report of ERS, ISEE, HEI and WHO. Eur Respir J. 2021;58(2).

Supplementary Table S3c: Manuscripts Excluded because they are Duplicates

1	Yao Y, Pan J, Liu Z, Kan H, Qiu Y, Meng X, et al. Association of particulate matter pollution and case fatality rate of COVID-19 in 49 Chinese cities. Science of the Total Environment. 2020;741 (no pagination).
2	Wannaz ED, Larrea Valdivia AE, Reyes Larico JA, Salcedo Pena J, Valenzuela Huillca C. PM<inf>10</inf> correlates with COVID-19 infections 15 days later in Arequipa, Peru. Environmental science and pollution research international. 2021;28(29):39648-54.
3	Viteri G, Diaz de Mera Y, Rodriguez A, Rodriguez D, Tajuelo M, Escalona A, et al. Impact of SARS-CoV-2 lockdown and de-escalation on air-quality parameters. Chemosphere. 2021;265 (no pagination).
4	Villanueva F, Notario A, Cabanas B, Martin P, Salgado S, Gabriel MF. Assessment of CO<inf>2</inf> and aerosol (PM<inf>2.5</inf>, PM<inf>10</inf>, UFP) concentrations during the reopening of schools in the COVID-19 pandemic: The case of a metropolitan area in Central-Southern Spain. Environmental Research. 2021;197 (no pagination).
5	Urrutia-Pereira M, Mello-da-Silva CA, Sole D. COVID-19 and air pollution: A dangerous association? Allergologia et Immunopathologia. 2020;48(5):496-9.
6	Tung NT, Cheng PC, Chi KH, Hsiao TC, Jones T, BeruBe K, et al. Particulate matter and SARS-CoV-2: A possible model of COVID-19 transmission. Science of the Total Environment. 2021;750 (no pagination).
7	Tavella RA, da Silva Junior FMR. Watch out for trends: did ozone increased or decreased during the COVID-19 pandemic? Environmental science and pollution research international. 2021;28(47):67880-5.
8	Skubacz K, Hildebrandt R, Zgorska A, Dyduch Z, Samolej K, Smolinski A. Transport of Aerosols in Underground Mine Workings in Terms of SARS-CoV-2 Virus Threat. Molecules. 2021;26(12).
9	Sciomer S, Moscucci F, Magri D, Badagliacca R, Piccirillo G, Agostoni P. SARS-CoV-2 spread in Northern Italy: what about the pollution role? Environmental Monitoring and Assessment. 2020;192(6) (no pagination).
10	Samillan VJ, Flores-Leon D, Rojas E, Zutta BR. Environmental and climatic impact on the infection and mortality of SARS-CoV-2 in Peru. Journal of Basic and Clinical Physiology and Pharmacology. 2021;32(5):935-42.
11	Safdar N, Crnich CJ, Maki DG. The pathogenesis of ventilator-associated pneumonia: its relevance to developing effective strategies for prevention. Respiratory care. 2005;50(6):725-39; discussion 39-41.
12	Querol X, Massague J, Alastuey A, Moreno T, Gangoiti G, Mantilla E, et al. Lessons from the COVID-19 air pollution decrease in Spain: Now what? Science of the Total Environment. 2021;779 (no pagination).
13	Pozzer A, Dominici F, Haines A, Witt C, Munzel T, Lelieveld J. Regional and global contributions of air pollution to risk of death from COVID-19. Cardiovascular Research. 2020;116(14):2247-53.
14	Paez-Osuna F, Valencia-Castaneda G, Rebolledo UA. The link between COVID-19 mortality and PM<inf>2.5</inf> emissions in rural and medium-size municipalities considering population density, dust events, and wind speed. Chemosphere. 2022;Part 1. 286 (no pagination).
15	Orak NH, Ozdemir O. The impacts of COVID-19 lockdown on PM<inf>10</inf> and SO<inf>2</inf> concentrations and association with human mobility across Turkey. Environmental Research. 2021;197 (no pagination).
16	Meo SA, Abukhalaf AA, Alomar AA, Alessa OM, Sami W, Klonoff DC. Effect of environmental pollutants PM-2.5, carbon monoxide, and ozone on the incidence and mortality of SARS-COV-2 infection in ten wildfire affected counties in California. Science of the Total Environment. 2021;757 (no pagination).
17	Meo SA, Abukhalaf AA, Alessa OM, Alarifi AS, Sami W, Klonoff DC. Effect of environmental pollutants PM2.5, CO, No<inf>2</inf>, and O<inf>3</inf> on the incidence and mortality of SARS-CoV-2 infection in five regions of the USA. International Journal of Environmental Research and Public Health. 2021;18(15) (no pagination).
18	Mendy A, Wu X, Keller JL, Fassler CS, Apewokin S, Mersha TB, et al. Long-term exposure to fine particulate matter and hospitalization in COVID-19 patients. Respiratory Medicine. 2021;178 (no pagination).
19	Mendy A, Wu X, Keller JL, Fassler CS, Apewokin S, Mersha TB, et al. Air pollution and the pandemic: Long-term PM<inf>2.5</inf> exposure and disease severity in COVID-19 patients. Respirology. 2021;26(12):1181-7.
20	Mendez-Espinosa JF, Rojas NY, Vargas J, Pachon JE, Belalcazar LC, Ramirez O. Air quality variations in Northern South America during the COVID-19 lockdown. Science of the Total Environment. 2020;749 (no pagination).
21	Martorell-Marugan J, Villatoro-Garcia JA, Garcia-Moreno A, Lopez-Dominguez R, Requena F, Merelo JJ, et al. DatAC: A visual analytics platform to explore climate and air quality indicators associated with the COVID-19 pandemic in Spain. Science of the Total Environment. 2021;750 (no pagination).
22	Marques M, Rovira J, Nadal M, Domingo JL. Effects of air pollution on the potential transmission and mortality of COVID-19: A preliminary case-study in Tarragona Province (Catalonia, Spain). Environmental Research. 2021;192 (no pagination).
23	Marques M, Domingo JL. Positive association between outdoor air pollution and the incidence and severity of COVID-19. A review of the recent scientific evidences. Environmental Research. 2022;203 (no pagination).
24	Marques M, Correig E, Ibarretxe D, Anoro E, Antonio Arroyo J, Jerico C, et al. Long-term exposure to PM<inf>10</inf> above WHO guidelines exacerbates COVID-19 severity and mortality. Environment International. 2022;158 (no pagination).
25	Lym Y, Kim KJ. Exploring the effects of PM<inf>2.5</inf> and temperature on COVID-19 transmission in Seoul, South Korea. Environmental Research. 2022;203 (no pagination).

26	Lopez-Feldman A, Heres D, Marquez-Padilla F. Air pollution exposure and COVID-19: A look at mortality in Mexico City using individual-level data. Science of the Total Environment. 2021;756 (no pagination).
27	Liu DT, Philips KM, Speth MM, Besser G, Mueller CA, Sedaghat AR. Portable HEPA Purifiers to Eliminate Airborne SARS-CoV-2: A Systematic Review. Otolaryngology Head and Neck Surgery. 2021.
28	Linillos-Pradillo B, Rancan L, Ramiro ED, Vara E, Artinano B, Arias J. Determination of SARS-CoV-2 RNA in different particulate matter size fractions of outdoor air samples in Madrid during the lockdown. Environmental Research. 2021;195 (no pagination).
29	Leao MLP, Penteado JO, Ulguim SM, Gabriel RR, Dos Santos M, Brum AN, et al. Health impact assessment of air pollutants during the COVID-19 pandemic in a Brazilian metropolis. Environmental science and pollution research international. 2021;28(31):41843-50.
30	Kusaba Y, Izumi S, Takasaki J, Suzuki M, Katagiri D, Katsuno T, et al. Successful recovery from COVID-19-associated acute respiratory failure with polymyxin b-immobilized fiber column-direct hemoperfusion. Internal Medicine. 2020;59(19):2405-8.
31	Khan TR, Parker DS, Withers C. Mitigation of airborne contaminant spread through simple interventions in an occupied single-family home. International Journal of Environmental Research and Public Health. 2021;18(11) (no pagination).
32	Helm Ii S, Harmon PC, Noe C, Calodney AK, Abd-Elsayed A, Knezevic NN, et al. Transforaminal Epidural Steroid Injections: A Systematic Review and Meta-Analysis of Efficacy and Safety. Pain physician. 2021;24(S1):S209-S32.
33	Goncalves J, Koritnik T, Paragi M. Assessment of weather and atmospheric pollution as a co-factor in the spread of SARS-CoV-2. Acta Biomedica. 2021;92(3) (no pagination).
34	Fang F, Mu L, Zhu Y, Rao J, Heymann J, Zhang ZF. Long-term exposure to pm<inf>2.5</inf>, facemask mandates, stay home orders and COVID-19 incidence in the united states. International Journal of Environmental Research and Public Health. 2021;18(12) (no pagination).
35	Dragone R, Licciardi G, Grasso G, Del Gaudio C, Chanussot J. Analysis of the chemical and physical environmental aspects that promoted the spread of sars-cov-2 in the lombard area. International Journal of Environmental Research and Public Health. 2021;18(3):1-21.
36	Dominguez-amarillo S, Fernandez-aguera J, Cesteros-garcia S, Gonzalez-lezcano RA. Bad air can also kill: Residential indoor air quality and pollutant exposure risk during the covid-19 crisis. International Journal of Environmental Research and Public Health. 2020;17(19):1-34.
37	Domingo JL, Marques M, Rovira J. Influence of airborne transmission of SARS-CoV-2 on COVID-19 pandemic. A review. Environmental Research. 2020;188 (no pagination).
38	Curtis L. PM<inf>2.5</inf>, NO<inf>2</inf>, wildfires, and other environmental exposures are linked to higher Covid 19 incidence, severity, and death rates. Environmental science and pollution research international. 2021;28(39):54429-47.
39	Cui Y, Zhang ZF, Froines J, Zhao J, Wang H, Yu SZ, et al. Air pollution and case fatality of SARS in the People's Republic of China: an ecologic study. Environmental health : a global access science source. 2003;2(1) (no pagination).
40	Comunian S, Dongo D, Milani C, Palestini P. Air pollution and covid-19: The role of particulate matter in the spread and increase of covid-19's morbidity and mortality. International Journal of Environmental Research and Public Health. 2020;17(12):1-22.
41	Collivignarelli MC, Abba A, Caccamo FM, Bertanza G, Pedrazzani R, Baldi M, et al. Can particulate matter be identified as the primary cause of the rapid spread of CoViD-19 in some areas of Northern Italy? Environmental science and pollution research international. 2021;26.
42	Coccia M. Effects of the spread of COVID-19 on public health of polluted cities: results of the first wave for explaining the deja vu in the second wave of COVID-19 pandemic and epidemics of future vital agents. Environmental science and pollution research international. 2021;28(15):19147-54.
43	Coccia M. Factors determining the diffusion of COVID-19 and suggested strategy to prevent future accelerated viral infectivity similar to COVID. Science of the Total Environment. 2020;729 (no pagination).
44	Calderon-Garciduenas L, Torres-Jardon R, Franco-Lira M, Kulesza R, Gonzalez-Maciel A, Reynoso-Robles R, et al. Environmental Nanoparticles, SARS-CoV-2 Brain Involvement, and Potential Acceleration of Alzheimer's and Parkinson's Diseases in Young Urbanites Exposed to Air Pollution. Journal of Alzheimer's Disease. 2020;78(2):479-503.
45	Brocke SA, Billings GT, Taft-Benz S, Alexis NE, Heise MT, Jaspers I. Woodsmoke particle exposure prior to SARS-CoV-2 infection alters antiviral response gene expression in human nasal epithelial cells in a sex-dependent manner. American journal of physiology Lung cellular and molecular physiology. 2022;02.
46	Briz-Redon A, Belenguer-Sapina C, Serrano-Aroca A. Changes in air pollution during COVID-19 lockdown in Spain: A multi-city study. Journal of environmental sciences (China). 2021;101:16-26.
47	Brandt EB, Mersha TB. Environmental Determinants of Coronavirus Disease 2019 (COVID-19). Current Allergy and Asthma Reports. 2021;21(3) (no pagination).
48	Bozack A, Pierre S, DeFelice N, Colicino E, Jack D, Chillrud SN, et al. Long-Term Air Pollution Exposure and COVID-19 Mortality: A Patient-Level Analysis from New York City. American journal of respiratory and critical care medicine. 2021;09.
49	Bossak BH, Andritsch S. COVID-19 and Air Pollution: A Spatial Analysis of Particulate Matter Concentration and Pandemic-Associated Mortality in the US. International Journal of Environmental Research and Public Health. 2022;19(1) (no pagination).
50	Borro M, Di Girolamo P, Gentile G, De Luca O, Preissner R, Marcolongo A, et al. Evidence-based considerations exploring relations between sars-cov-2 pandemic and air pollution: Involvement of pm2.5-

	mediated up-regulation of the viral receptor ace-2. International Journal of Environmental Research and Public Health. 2020;17(15):1-13.
51	Borisova T, Komisarenko S. Air pollution particulate matter as a potential carrier of SARS-CoV-2 to the nervous system and/or neurological symptom enhancer: arguments in favor. Environmental science and pollution research international. 2021;28(30):40371-7.
52	Bianconi V, Bronzo P, Banach M, Sahebkar A, Mannarino MR, Pirro M. Particulate matter pollution and the Covid-19 outbreak: Results from Italian regions and provinces. Archives of Medical Science. 2020;16(5):985-92.
53	Aykac N, Etiler N. COVID-19 mortality in Istanbul in association with air pollution and socioeconomic status: an ecological study. Environmental science and pollution research international. 2022;29(9):13700-8.
54	Anand U, Cabreros C, Mal J, Ballesteros F, Sillanpaa M, Tripathi V, et al. Novel coronavirus disease 2019 (COVID-19) pandemic: From transmission to control with an interdisciplinary vision. Environmental Research. 2021;197 (no pagination).
55	Zoran MA, Savastru RS, Savastru DM, Tautan MN. Assessing the relationship between surface levels of PM2.5 and PM10 particulate matter impact on COVID-19 in Milan, Italy. Sci Total Environ. 2020;738:139825.
56	Zhu C, Maharajan K, Liu K, Zhang Y. Role of atmospheric particulate matter exposure in COVID-19 and other health risks in human: A review. Environ Res. 2021;198:111281.
57	Zhao M, Liu Y, Gyilbag A. Assessment of Meteorological Variables and Air Pollution Affecting COVID-19 Cases in Urban Agglomerations: Evidence from China. Int J Environ Res Public Health. 2022;19(1).
58	Zhao C, Fang X, Feng Y, Fang X, He J, Pan H. Emerging role of air pollution and meteorological parameters in COVID-19. J Evid Based Med. 2021;14(2):123-38.
59	Zhang XS, Duchaine C. SARS-CoV-2 and Health Care Worker Protection in Low-Risk Settings: a Review of Modes of Transmission and a Novel Airborne Model Involving Inhalable Particles. Clin Microbiol Rev. 2020;34(1).
60	Zhang R, Zhao H. Small-Angle Particle Counting Coupled Photometry for Real-Time Detection of Respirable Particle Size Segmentation Mass Concentration. Sensors (Basel). 2021;21(17).
61	Zangari Del Balzo G. Methodological notes on pandemic virus SARS-CoV-2 research. Theory Biosci. 2021;140(3):279-94.
62	Xu L, Taylor JE, Kaiser J. Short-term air pollution exposure and COVID-19 infection in the United States. Environ Pollut. 2022;292(Pt B):118369.
63	Workman AD, Jafari A, Welling DB, Varvares MA, Gray ST, Holbrook EH, et al. Airborne Aerosol Generation During Endonasal Procedures in the Era of COVID-19: Risks and Recommendations. Otolaryngol Head Neck Surg. 2020;163(3):465-70.
64	Woodby B, Arnold MM, Valacchi G. SARS-CoV-2 infection, COVID-19 pathogenesis, and exposure to air pollution: What is the connection? Ann N Y Acad Sci. 2021;1486(1):15-38.
65	Wathore R, Gupta A, Bherwani H, Labhasetwar N. Understanding air and water borne transmission and survival of coronavirus: Insights and way forward for SARS-CoV-2. Sci Total Environ. 2020;749:141486.
66	Valdés Salgado M, Smith P, Opazo MA, Huneeus N. Long-Term Exposure to Fine and Coarse Particulate Matter and COVID-19 Incidence and Mortality Rate in Chile during 2020. Int J Environ Res Public Health. 2021;18(14).
67	Travaglio M, Yu Y, Popovic R, Selley L, Leal NS, Martins LM. Links between air pollution and COVID-19 in England. Environ Pollut. 2021;268(Pt A):115859.
68	Traina G, Barbalace A, Betti F, Bolzacchini E, Bonini M, Contini D, et al. What impact of air pollution in pediatric respiratory allergic diseases. Pediatr Allergy Immunol. 2020;31 Suppl 26:26-8.
69	Toczylowski K, Wietlicka-Piszcz M, Grabowska M, Sulik A. Cumulative Effects of Particulate Matter Pollution and Meteorological Variables on the Risk of Influenza-Like Illness. Viruses. 2021;13(4).
70	Tateo F, Fiorino S, Peruzzo L, Zippi M, De Biase D, Lari F, et al. Effects of environmental parameters and their interactions on the spreading of SARS-CoV-2 in North Italy under different social restrictions. A new approach based on multivariate analysis. Environ Res. 2022;210:112921.
71	Tanwar V, Adelstein JM, Wold LE. Double trouble: combined cardiovascular effects of particulate matter exposure and coronavirus disease 2019. Cardiovasc Res. 2021;117(1):85-95.
72	Takagi H. Risk and protective factors of SARS-CoV-2 infection. J Med Virol. 2021;93(2):649-51.
73	Tadano YS, Potgieter-Vermaak S, Kachba YR, Chiroli DMG, Casacio L, Santos-Silva JC, et al. Dynamic model to predict the association between air quality, COVID-19 cases, and level of lockdown. Environ Pollut. 2021;268(Pt B):115920.
74	Sullivan JR, Rademaker M, Goodman G, Bekhor P, Al-Niaimi F. Guidance on infection control and plume management with Laser and Energy-Based Devices taking into consideration COVID-19. Australas J Dermatol. 2021;62(1):37-40.
75	Subat YW, Hainy ME, Torgerud KD, Sajgalik P, Guntupalli SK, Johnson BD, et al. Aerosol Generation and Mitigation During Methacholine Bronchoprovocation Testing: Infection Control Implications in the Era of COVID-19. Respir Care. 2021;66(12):1858-65.
76	Subat YW, Guntupalli SK, Sajgalik P, Hainy ME, Torgerud KD, Helgeson SA, et al. Aerosol Generation During Peak Flow Testing: Clinical Implications for COVID-19. Respir Care. 2021;66(8):1291-8.
77	Stufano A, Lisco S, Bartolomeo N, Marsico A, Lucchese G, Jahantigh H, et al. COVID19 outbreak in Lombardy, Italy: An analysis on the short-term relationship between air pollution, climatic factors and the susceptibility to SARS-CoV-2 infection. Environ Res. 2021;198:111197.

78	Stern RA, Koutrakis P, Martins MAG, Lemos B, Dowd SE, Sunderland EM, et al. Characterization of hospital airborne SARS-CoV-2. Respir Res. 2021;22(1):73.
79	Stapleton EM, Welch JL, Ubeda EA, Xiang J, Zabner J, Thornell IM, et al. Urban Particulate Matter Impairment of Airway Surface Liquid-Mediated Coronavirus Inactivation. J Infect Dis. 2022;225(2):214-8.
80	Srivastava A. COVID-19 and air pollution and meteorology-an intricate relationship: A review. Chemosphere. 2021;263:128297.
81	Shukla S, Khan R, Saxena A, Sekar S, Ali EF, Shaheen SM. Appraisal of COVID-19 lockdown and unlocking effects on the air quality of North India. Environ Res. 2022;204(Pt B):112107.
82	Sharma AK, Balyan P. Air pollution and COVID-19: Is the connect worth its weight? Indian J Public Health. 2020;64(Supplement):S132-s4.
83	Setti L, Passarini F, De Gennaro G, Barbieri P, Perrone MG, Borelli M, et al. Airborne Transmission Route of COVID-19: Why 2 Meters/6 Feet of Inter-Personal Distance Could Not Be Enough. Int J Environ Res Public Health. 2020;17(8).
84	Setti L, Passarini F, De Gennaro G, Barbieri P, Pallavicini A, Ruscio M, et al. Searching for SARS-COV-2 on Particulate Matter: A Possible Early Indicator of COVID-19 Epidemic Recurrence. Int J Environ Res Public Health. 2020;17(9).
85	Scalsky RJ, Chen YJ, Ying Z, Perry JA, Hong CC. The Social and Natural Environment's Impact on SARS-CoV-2 Infections in the UK Biobank. Int J Environ Res Public Health. 2022;19(1).
86	Santurtún A, Colom ML, Fdez-Arroyabe P, Real A D, Fernández-Olmo I, Zarrabeitia MT. Exposure to particulate matter: Direct and indirect role in the COVID-19 pandemic. Environ Res. 2022;206:112261.
87	Sangkham S, Thongtip S, Vongruang P. Influence of air pollution and meteorological factors on the spread of COVID-19 in the Bangkok Metropolitan Region and air quality during the outbreak. Environ Res. 2021;197:111104.
88	Sahoo MM. Significance between air pollutants, meteorological factors, and COVID-19 infections: probable evidences in India. Environ Sci Pollut Res Int. 2021;28(30):40474-95.
89	Ruran HB, Adamkiewicz G, Cunningham A, Petty CR, Greco KF, Gunnlaugsson S, et al. Air quality, Environment and Respiratory Outcomes in Bronchopulmonary Dysplasia, the AERO-BPD cohort study: design and adaptation during the SARS-CoV-2 pandemic. BMJ Open Respir Res. 2021;8(1).
90	Rugani B, Conticini E, Frediani B, Caro D. Decrease in life expectancy due to COVID-19 disease not offset by reduced environmental impacts associated with lockdowns in Italy. Environ Pollut. 2022;292(Pt A):118224.
91	Rivera-Rios JC, Joo T, Takeuchi M, Orlando TM, Bevington T, Mathis JW, et al. In-flight particulate matter concentrations in commercial flights are likely lower than other indoor environments. Indoor Air. 2021;31(5):1484-94.
92	Reyes MSS, Medina PMB. Environmental pollutant exposure can exacerbate COVID-19 neurologic symptoms. Med Hypotheses. 2020;144:110136.
93	Rexhepi I, Mangifesta R, Santilli M, Guri S, Di Carlo P, D'Addazio G, et al. Effects of Natural Ventilation and Saliva Standard Ejectors during the COVID-19 Pandemic: A Quantitative Analysis of Aerosol Produced during Dental Procedures. Int J Environ Res Public Health. 2021;18(14).
94	Ram K, Thakur RC, Singh DK, Kawamura K, Shimouchi A, Sekine Y, et al. Why airborne transmission hasn't been conclusive in case of COVID-19? An atmospheric science perspective. Sci Total Environ. 2021;773:145525.
95	Rajajee V, Williamson CA. Use of a Novel Negative-Pressure Tent During Bedside Tracheostomy in COVID-19 Patients. Neurocrit Care. 2020;33(2):597-603.
96	Poyraz BM, Engin ED, Engin AB, Engin A. The effect of environmental diesel exhaust pollution on SARS-CoV-2 infection: The mechanism of pulmonary ground glass opacity. Environ Toxicol Pharmacol. 2021;86:103657.
97	Pivato A, Amoruso I, Formenton G, Di Maria F, Bonato T, Vanin S, et al. Evaluating the presence of SARS-CoV-2 RNA in the particulate matters during the peak of COVID-19 in Padua, northern Italy. Sci Total Environ. 2021;784:147129.
98	Perret J, Dharmage S. COVID-19 hospitalizations: Another adverse impact of ambient air pollution? Respirology. 2021;26(12):1101-2.
99	Pandey AS, Ringer AJ, Rai AT, Kan P, Jabbour P, Siddiqui AH, et al. Minimizing SARS-CoV-2 exposure when performing surgical interventions during the COVID-19 pandemic. J Neurointerv Surg. 2020;12(7):643-7.
100	Nozza E, Valentini S, Melzi G, Vecchi R, Corsini E. Advances on the immunotoxicity of outdoor particulate matter: A focus on physical and chemical properties and respiratory defence mechanisms. Sci Total Environ. 2021;780:146391.
101	Norouzi N, Asadi Z. Air pollution impact on the Covid-19 mortality in Iran considering the comorbidity (obesity, diabetes, and hypertension) correlations. Environ Res. 2022;204(Pt A):112020.
102	Nor NSM, Yip CW, Ibrahim N, Jaafar MH, Rashid ZZ, Mustafa N, et al. Particulate matter (PM(2.5)) as a potential SARS-CoV-2 carrier. Sci Rep. 2021;11(1):2508.
103	Navarro KM, Clark KA, Hardt DJ, Reid CE, Lahm PW, Domitrovich JW, et al. Wildland firefighter exposure to smoke and COVID-19: A new risk on the fire line. Sci Total Environ. 2021;760:144296.
104	Naidoo P, Ghazi T, Chuturgoon AA, Naidoo RN, Ramsuran V, Mpaka-Mbatha MN, et al. SARS-CoV-2 and helminth co-infections, and environmental pollution exposure: An epidemiological and immunological perspective. Environ Int. 2021;156:106695.
105	Mulder C, Conti E, Saccone S, Federico C. Beyond virology: environmental constraints of the first wave of COVID-19 cases in Italy. Environ Sci Pollut Res Int. 2021;28(24):31996-2004.

106	Moshammer H, Poteser M, Hutter HP. COVID-19 and air pollution in Vienna-a time series approach. Wien Klin Wochenschr. 2021;133(17-18):951-7.
107	Moelling K, Broecker F. Air Microbiome and Pollution: Composition and Potential Effects on Human Health, Including SARS Coronavirus Infection. J Environ Public Health. 2020;2020:1646943.
108	Mescoli A, Maffei G, Pillo G, Bortone G, Marchesi S, Morandi E, et al. The Secretive Liaison of Particulate Matter and SARS-CoV-2. A Hypothesis and Theory Investigation. Front Genet. 2020;11:579964.
109	Meo SA, Almutairi FJ, Abukhalaf AA, Usmani AM. Effect of Green Space Environment on Air Pollutants PM2.5, PM10, CO, O(3), and Incidence and Mortality of SARS-CoV-2 in Highly Green and Less-Green Countries. Int J Environ Res Public Health. 2021;18(24).
110	Meo SA, Almutairi FJ, Abukhalaf AA, Alessa OM, Al-Khlaiwi T, Meo AS. Sandstorm and its effect on particulate matter PM 2.5, carbon monoxide, nitrogen dioxide, ozone pollutants and SARS-CoV-2 cases and deaths. Sci Total Environ. 2021;795:148764.
111	Meo SA, Al-Khlaiwi T, Ullah CH. Effect of ambient air pollutants PM2.5 and PM10 on COVID-19 incidence and mortality: observational study. Eur Rev Med Pharmacol Sci. 2021;25(23):7553-64.
112	Meo SA, Abukhalaf AA, Alomar AA, Alessa OM. Wildfire and COVID-19 pandemic: effect of environmental pollution PM-2.5 and carbon monoxide on the dynamics of daily cases and deaths due to SARS-COV-2 infection in San-Francisco USA. Eur Rev Med Pharmacol Sci. 2020;24(19):10286-92.
113	Menendez JA. Metformin and SARS-CoV-2: mechanistic lessons on air pollution to weather the cytokine/thrombotic storm in COVID-19. Aging (Albany NY). 2020;12(10):8760-5.
114	Mendy A, Wu X, Keller JL, Fassler CS, Apewokin S, Mersha TB, et al. Air pollution and the pandemic: Long-term PM(2.5) exposure and disease severity in COVID-19 patients. Respirology. 2021;26(12):1181-7.
115	Mendoza DL, Benney TM, Bares R, Crosman ET. Intra-city variability of fine particulate matter during COVID-19 lockdown: A case study from Park City, Utah. Environ Res. 2021;201:111471.
116	Mehmood K, Bao Y, Petropoulos GP, Abbas R, Abrar MM, Saifullah, et al. Investigating connections between COVID-19 pandemic, air pollution and community interventions for Pakistan employing geoinformation technologies. Chemosphere. 2021;272:129809.
117	Marwah M, Agrawala PK. COVID-19 lockdown and environmental pollution: an Indian multi-state investigation. Environ Monit Assess. 2022;194(2):49.
118	Manivannan J, Sundaresan L. Systems level insights into the impact of airborne exposure on SARS-CoV-2 pathogenesis and COVID-19 outcome - A multi-omics big data study. Gene Rep. 2021;25:101312.
119	Maleki M, Anvari E, Hopke PK, Noorimotlagh Z, Mirzaee SA. An updated systematic review on the association between atmospheric particulate matter pollution and prevalence of SARS-CoV-2. Environ Res. 2021;195:110898.
120	Macias-Verde D, Lara PC, Burgos-Burgos J. Same pollution sources for climate change might be hyperactivating the NLRP3 inflammasome and exacerbating neuroinflammation and SARS mortality. Med Hypotheses. 2021;146:110396.
121	Lu B, Wu N, Jiang J, Li X. Associations of acute exposure to airborne pollutants with COVID-19 infection: evidence from China. Environ Sci Pollut Res Int. 2021;28(36):50554-64.
122	López JH, Romo A S, Molina DC, Hernández G, Cureño A BG, Acosta MA, et al. Detection of Sars-Cov-2 in the air of two hospitals in Hermosillo, Sonora, México, utilizing a low-cost environmental monitoring system. Int J Infect Dis. 2021;102:478-82.
123	Li HH, Liu CC, Hsu TW, Lin JH, Hsu JW, Li AF, et al. Upregulation of ACE2 and TMPRSS2 by particulate matter and idiopathic pulmonary fibrosis: a potential role in severe COVID-19. Part Fibre Toxicol. 2021;18(1):11.
124	Li H, Xu XL, Dai DW, Huang ZY, Ma Z, Guan YJ. Air pollution and temperature are associated with increased COVID-19 incidence: A time series study. Int J Infect Dis. 2020;97:278-82.
125	Lembo R, Landoni G, Cianfanelli L, Frontera A. Air pollutants and SARS-CoV-2 in 33 European countries. Acta Biomed. 2021;92(1):e2021166.
126	Lai A, Chang ML, O'Donnell RP, Zhou C, Sumner JA, Hsiai TK. Association of COVID-19 transmission with high levels of ambient pollutants: Initiation and impact of the inflammatory response on cardiopulmonary disease. Sci Total Environ. 2021;779:146464.
127	Konwar C, Asiimwe R, Inkster AM, Merrill SM, Negri GL, Aristizabal MJ, et al. Risk-focused differences in molecular processes implicated in SARS-CoV-2 infection: corollaries in DNA methylation and gene expression. Epigenetics Chromatin. 2021;14(1):54.
128	Kiser D, Elhanan G, Metcalf WJ, Schnieder B, Grzymski JJ. SARS-CoV-2 test positivity rate in Reno, Nevada: association with PM2.5 during the 2020 wildfire smoke events in the western United States. J Expo Sci Environ Epidemiol. 2021;31(5):797-803.
129	Kim JH, Kim J, Kim WJ, Choi YH, Yang SR, Hong SH. Diesel Particulate Matter 2.5 Induces Epithelial-to-Mesenchymal Transition and Upregulation of SARS-CoV-2 Receptor during Human Pluripotent Stem Cell-Derived Alveolar Organoid Development. Int J Environ Res Public Health. 2020;17(22).
130	Khan YA. The COVID-19 pandemic and its impact on environment: the case of the major cities in Pakistan. Environ Sci Pollut Res Int. 2021;28(39):54728-43.
131	Jephcote C, Hansell AL, Adams K, Gulliver J. Changes in air quality during COVID-19 'lockdown' in the United Kingdom. Environ Pollut. 2021;272:116011.
132	Ibarra-Espinosa S, Dias de Freitas E, Ropkins K, Dominici F, Rehbein A. Negative-Binomial and quasi-poisson regressions between COVID-19, mobility and environment in São Paulo, Brazil. Environ Res. 2022;204(Pt D):112369.

133	Hill WC, Hull MS, MacCuspie RI. Testing of Commercial Masks and Respirators and Cotton Mask Insert Materials using SARS-CoV-2 Virion-Sized Particulates: Comparison of Ideal Aerosol Filtration Efficiency versus Fitted Filtration Efficiency. Nano Lett. 2020;20(10):7642-7.
134	Hao W, Wu J, Zhao X, Liang D, Yu X, Cao H, et al. Quantitative Evaluation of Aerosol Generation from Non-contact Tonometry and its Correlation with Tear Film Characteristics. Adv Ther. 2021;38(6):3066-76.
135	Hansell AL, Villeneuve PJ. Invited Perspective: Ambient Air Pollution and SARS-CoV-2: Research Challenges and Public Health Implications. Environ Health Perspect. 2021;129(11):111303.
136	Gujral H, Sinha A. Association between exposure to airborne pollutants and COVID-19 in Los Angeles, United States with ensemble-based dynamic emission model. Environ Res. 2021;194:110704.
137	Gianquintieri L, Brovelli MA, Pagliosa A, Bonora R, Sechi GM, Caiani EG. Geospatial Correlation Analysis between Air Pollution Indicators and Estimated Speed of COVID-19 Diffusion in the Lombardy Region (Italy). Int J Environ Res Public Health. 2021;18(22).
138	Ghaffari HR, Farshidi H, Alipour V, Dindarloo K, Azad MH, Jamalidoust M, et al. Detection of SARS-CoV-2 in the indoor air of intensive care unit (ICU) for severe COVID-19 patients and its surroundings: considering the role of environmental conditions. Environ Sci Pollut Res Int. 2021:1-7.
139	Fronza R, Lusic M, Schmidt M, Lucic B. Spatial-Temporal Variations in Atmospheric Factors Contribute to SARS-CoV-2 Outbreak. Viruses. 2020;12(6).
140	Frontera A, Cianfanelli L, Vlachos K, Landoni G, Cremona G. Severe air pollution links to higher mortality in COVID-19 patients: The "double-hit" hypothesis. J Infect. 2020;81(2):255-9.
141	Fiorito S, Soligo M, Gao Y, Ogulur I, Akdis CA, Bonini S. Is epithelial barrier hypothesis the key to understanding the higher incidence and excess mortality during COVID-19 pandemic? The case of Northern Italy. Allergy. 2022.
142	Farhangrazi ZS, Sancini G, Hunter AC, Moghimi SM. Airborne Particulate Matter and SARS-CoV-2 Partnership: Virus Hitchhiking, Stabilization and Immune Cell Targeting - A Hypothesis. Front Immunol. 2020;11:579352.
143	Espejo W, Celis JE, Chiang G, Bahamonde P. Environment and COVID-19: Pollutants, impacts, dissemination, management and recommendations for facing future epidemic threats. Sci Total Environ. 2020;747:141314.
144	Edwards L, Rutter G, Iverson L, Wilson L, Chadha TS, Wilkinson P, et al. Personal exposure monitoring of PM(2.5) among US diplomats in Kathmandu during the COVID-19 lockdown, March to June 2020. Sci Total Environ. 2021;772:144836.
145	Duval JFL, van Leeuwen HP, Norde W, Town RM. Chemodynamic features of nanoparticles: Application to understanding the dynamic life cycle of SARS-CoV-2 in aerosols and aqueous biointerfacial zones. Adv Colloid Interface Sci. 2021;290:102400.
146	Dubey A, Kotnala G, Mandal TK, Sonkar SC, Singh VK, Guru SA, et al. Evidence of the presence of SARS-CoV-2 virus in atmospheric air and surfaces of a dedicated COVID hospital. J Med Virol. 2021;93(9):5339-49.
147	Dondi A, Betti L, Carbone C, Dormi A, Paglione M, Rinaldi M, et al. Understanding the environmental factors related to the decrease in Pediatric Emergency Department referrals for acute asthma during the SARS-CoV-2 pandemic. Pediatr Pulmonol. 2022;57(1):66-74.
148	Domingo JL, Rovira J. Effects of air pollutants on the transmission and severity of respiratory viral infections. Environ Res. 2020;187:109650.
149	Din AR, Hindocha A, Patel T, Sudarshan S, Cagney N, Koched A, et al. Quantitative analysis of particulate matter release during orthodontic procedures: a pilot study. Br Dent J. 2020:1-7.
150	Di Ciaula A, Bonfrate L, Portincasa P, Appice C, Belfiore A, Binetti M, et al. Nitrogen dioxide pollution increases vulnerability to COVID-19 through altered immune function. Environ Sci Pollut Res Int. 2022.
151	Di Cerbo A. Air pollution and SARS-CoV-2 in the Po Valley: possible environmental persistence? Minerva Med. 2020;111(4):306-7.
152	Dettori M, Deiana G, Balletto G, Borruso G, Murgante B, Arghittu A, et al. Air pollutants and risk of death due to COVID-19 in Italy. Environ Res. 2021;192:110459.
153	De Matteis S, Forastiere F, Baldacci S, Maio S, Tagliaferro S, Fasola S, et al. Issue 1 - "Update on adverse respiratory effects of outdoor air pollution". Part 1): Outdoor air pollution and respiratory diseases: A general update and an Italian perspective. Pulmonology. 2022.
154	Datta M, Singh DD, Naqvi AR. Molecular Diagnostic Tools for the Detection of SARS-CoV-2. Int Rev Immunol. 2021;40(1-2):143-56.
155	Daoud AK, Hall JK, Petrick H, Strong A, Piggott C. The Potential for Cloth Masks to Protect Health Care Clinicians From SARS-CoV-2: A Rapid Review. Ann Fam Med. 2021;19(1):55-62.
156	Cruz R, Lima-Silva AE, Bertuzzi R, Hoinaski L. Exercising under particulate matter exposure: Providing theoretical support for lung deposition and its relationship with COVID-19. Environ Res. 2021;202:111755.
157	Cortes-Ramirez J, Michael RN, Knibbs LD, Bambrick H, Haswell MR, Wraith D. The association of wildfire air pollution with COVID-19 incidence in New South Wales, Australia. Sci Total Environ. 2022;809:151158.
158	Copat C, Cristaldi A, Fiore M, Grasso A, Zuccarello P, Signorelli SS, et al. The role of air pollution (PM and NO(2)) in COVID-19 spread and lethality: A systematic review. Environ Res. 2020;191:110129.
159	Christophi CA, Sotos-Prieto M, Lan FY, Delgado-Velandia M, Efthymiou V, Gaviola GC, et al. Ambient temperature and subsequent COVID-19 mortality in the OECD countries and individual United States. Sci Rep. 2021;11(1):8710.
160	Christopherson DA, Yao WC, Lu M, Vijayakumar R, Sedaghat AR. High-Efficiency Particulate Air Filters in the Era of COVID-19: Function and Efficacy. Otolaryngol Head Neck Surg. 2020;163(6):1153-5.

161	Chadeau-Hyam M, Bodinier B, Elliott J, Whitaker MD, Tzoulaki I, Vermeulen R, et al. Risk factors for positive and negative COVID-19 tests: a cautious and in-depth analysis of UK biobank data. Int J Epidemiol. 2020;49(5):1454-67.
162	Cazzolla Gatti R, Velichevskaya A, Tateo A, Amoroso N, Monaco A. Machine learning reveals that prolonged exposure to air pollution is associated with SARS-CoV-2 mortality and infectivity in Italy. Environ Pollut. 2020;267:115471.
163	Caseiro A, von Schneidemesser E. APExpose_DE, an air quality exposure dataset for Germany 2010-2019. Sci Data. 2021;8(1):287.
164	Buonanno M, Welch D, Shuryak I, Brenner DJ. Far-UVC light (222 nm) efficiently and safely inactivates airborne human coronaviruses. Sci Rep. 2020;10(1):10285.
165	Borak J. Airborne Transmission of COVID-19. Occup Med (Lond). 2020;70(5):297-9.
166	Berg K, Romer Present P, Richardson K. Long-term air pollution and other risk factors associated with COVID-19 at the census tract level in Colorado. Environ Pollut. 2021;287:117584.
167	Belosi F, Conte M, Gianelle V, Santachiara G, Contini D. On the concentration of SARS-CoV-2 in outdoor air and the interaction with pre-existing atmospheric particles. Environ Res. 2021;193:110603.
168	Azuma K, Kagi N, Kim H, Hayashi M. Impact of climate and ambient air pollution on the epidemic growth during COVID-19 outbreak in Japan. Environ Res. 2020;190:110042.
169	Atiyani R, Mustafa S, Alsari S, Darwish A, Janahi EM. Clearing the air about airborne transmission of SARS-CoV-2. Eur Rev Med Pharmacol Sci. 2021;25(21):6745-66.
170	Anser MK, Godil DI, Khan MA, Nassani AA, Zaman K, Abro MMQ. The impact of coal combustion, nitrous oxide emissions, and traffic emissions on COVID-19 cases: a Markov-switching approach. Environ Sci Pollut Res Int. 2021;28(45):64882-91.
171	Ali N, Fariha KA, Islam F, Mishu MA, Mohanto NC, Hosen MJ, et al. Exposure to air pollution and COVID-19 severity: A review of current insights, management, and challenges. Integr Environ Assess Manag. 2021;17(6):1114-22.
172	Aggarwal S, Balaji S, Singh T, Menon GR, Mandal S, Madhumathi J, et al. Association between ambient air pollutants and meteorological factors with SARS-CoV-2 transmission and mortality in India: an exploratory study. Environ Health. 2021;20(1):120.
173	Accarino G, Lorenzetti S, Aloisio G. Assessing correlations between short-term exposure to atmospheric pollutants and COVID-19 spread in all Italian territorial areas. Environ Pollut. 2021;268(Pt A):115714.
174	Kogevinas M, Castano-Vinyals G, Karachaliou M, Espinosa A, de Cid R, Garcia-Aymerich J, et al. Ambient air pollution in relation to SARS-CoV-2 infection, antibody response, and COVID-19 disease: A cohort study in Catalonia, Spain (COVICAT study). Environmental Health Perspectives. 2021;129(11) (no pagination).

SupplementalryTable S3d: Manuscripts Excluded because they are *in vitro*/ cell studies	
1	Stapleton EM, Welch JL, Ubeda EA, Xiang J, Zabner J, Thornell IM, et al. Urban Particulate Matter Impairment of Airway Surface Liquid-Mediated Coronavirus Inactivation. The Journal of infectious diseases. 2022;225(2):214-8.
2	Makovitzki A, Lerer E, Kafri Y, Adar Y, Cherry L, Lupu E, et al. Evaluation of a downstream process for the recovery and concentration of a Cell-Culture-Derived rVSV-Spike COVID-19 vaccine candidate. Vaccine. 2021;39(48):7044-51.
3	Konwar C, Asiimwe R, Inkster AM, Merrill SM, Negri GL, Aristizabal MJ, et al. Risk-focused differences in molecular processes implicated in SARS-CoV-2 infection: corollaries in DNA methylation and gene expression. Epigenetics and Chromatin. 2021;14(1) (no pagination).
4	Kim JH, Kim J, Kim WJ, Choi YH, Yang SR, Hong SH. Diesel particulate matter 2.5 induces epithelial-to-mesenchymal transition and upregulation of sars-cov-2 receptor during human pluripotent stem cell-derived alveolar organoid development. International Journal of Environmental Research and Public Health. 2020;17(22):1-15.
5	Hemsath JR, Liaci AM, Rubin JD, Parrett BJ, Lu SC, Nguyen TV, et al. Ex Vivo and In Vivo CD46 Receptor Utilization by Species D Human Adenovirus Serotype 26 (HAdV26). Journal of Virology. 2022;96(3) (no pagination).
6	Hazan SC, Daniels J. The role of roseburia in COVID-19 pathogenesis. American Journal of Gastroenterology. 2021;116(SUPPL):S70.
7	Dag Seker E, Erbahceci Timur IE. COVID-19: more than a respiratory virus, an optical coherence tomography study. International Ophthalmology. 2021;41(11):3815-24.
8	Barcia RN, O'Rourke B, Nguyen SH, Tilles AW, Garg P, Gemmiti CV, et al. Clinical relevance of AKI trial data for severe COVID-19 patients. Journal of the American Society of Nephrology. 2020;31:307.
9	Wohnhaas CT, Gindele JA, Kiechle T, Shen Y, Leparc GG, Stierstorfer B, et al. Cigarette Smoke Specifically Affects Small Airway Epithelial Cell Populations and Triggers the Expansion of Inflammatory and Squamous Differentiation Associated Basal Cells. Int J Mol Sci. 2021;22(14).
10	Smith JC, Sausville EL, Girish V, Yuan ML, Vasudevan A, John KM, et al. Cigarette Smoke Exposure and Inflammatory Signaling Increase the Expression of the SARS-CoV-2 Receptor ACE2 in the Respiratory Tract. Dev Cell. 2020;53(5):514-29.e3.

Supplementary Table S3e: Manuscripts Excluded because they are Letters to the editor	
1	Wei P, Lyu W, Wan T, Zheng Q, Tang W, Li J, et al. COVID-19: a novel risk factor for perioperative neurocognitive disorders. British Journal of Anaesthesia. 2021;127(3):e113-e5.
2	Waheed W, Carey ME, Tandan SR, Tandan R. Post COVID-19 vaccine small fiber neuropathy. Muscle and Nerve. 2021;64(1):E1-E2.
3	Tsui BCH, Pan S. Distanced-based dynamic behaviour of aerosol particles during aerosol-generating medical procedures. British Journal of Anaesthesia. 2020;125(5):e426-e8.
4	Takagi H. Risk and protective factors of SARS-CoV-2 infection. Journal of Medical Virology. 2021;93(2):649-51.
5	Perret J, Dharmage S. COVID-19 hospitalizations: Another adverse impact of ambient air pollution? Respirology. 2021;26(12):1101-2.
6	Niccoli G, Luescher TF, Crea F. Decreasedmyocardial infarction admissions during covid times: What can we learn? Cardiovascular Research. 2020;116(10):E126-E8.
7	Liccardi G, Martini M, Bilo MB, Milanese M, Rogliani P. Use of face masks and allergic nasal symptoms: Why not mention pollen count and air pollution data? American journal of otolaryngology. 2021:103363.
8	Guemes-Villahoz N, Burgos-Blasco B, Arribi-Vilela A, Arriola-Villalobos P, Vidal-Villegas B, Mendez-Fernandez R, et al. SARS-CoV-2 RNA detection in tears and conjunctival secretions of COVID-19 patients with conjunctivitis. Journal of Infection. 2020;81(3):452-82.
9	Gemignani F. Small Fiber Neuropathy and SARS-CoV-2 Infection. Another piece in the long COVID puzzle? Muscle and Nerve. 2022.
10	Finsterer J, Scorza FA, Scorza CA, Fiorini AC. SARS-CoV-2 and myasthenia. Journal of Medical Virology. 2021;93(7):4133-5.
11	Finsterer J. SARS-CoV-2 vaccinations may not only be complicated by GBS but also by distal small fibre neuropathy. Journal of Neuroimmunology. 2021;360 (no pagination).
12	Elkoundi A, Azzouzi A, El Wali A, Baite A, Bensghir M. Novel technique for safe fibreoptic tracheal intubation in COVID-19 patients. Anaesthesia Critical Care and Pain Medicine. 2020;39(4):465-6.
13	Dutheil F, Trousselard M, Navel V. SARS-CoV-2 as a protective factor for cardiovascular mortality? Atherosclerosis. 2020;304:64-5.
14	Dutheil F, Baker JS, Navel V. COVID-19 as a factor influencing air pollution? Environmental Pollution. 2020;Part A. 263 (no pagination).
15	Chirumbolo S, Bjorklund G. The bimodal SARS-CoV-2 outbreak in Italy as an effect of environmental and allergic causes. Journal of Allergy and Clinical Immunology. 2020;146(2):331-2.
16	Badani KK, Okhawere KE, Chen T, Korn TG, Razdan S, Meilika KN, et al. SARS-CoV-2 RNA Detected in Abdominal Insufflation Samples During Laparoscopic Surgery. European Urology. 2022;81(1):125-7.
17	Häfner SJ. This is not a pipe - But how harmful is electronic cigarette smoke. Biomed J. 2021;44(3):227-34.

Supplementary Table S3f: Manuscripts Excluded because they are Non-English	
1	Ricco M, Ranzieri S, Balzarini F, Bragazzi NL, Corradi M. SARS-CoV-2 infection and air pollutants: Correlation or causation? Science of the Total Environment. 2020;734 (no pagination).
2	Losilla-Rodriguez B, Maldonado N, Moreno-Mellado E, Lopez-Diaz A. COVID-19 natural herd immunity and risk of neuropsychiatric disorders. Revista de Psiquiatria y Salud Mental. 2020;13(4):228-9.
3	Colacci A, Bortone G, Maffei G, Marchesi S, Mescoli A, Parmagnani F, et al. Inquinamento ambientale e COVID-19: le basi molecolari della loro interazione, Environmental pollution and COVID-19: the molecular terms and predominant disease outcomes of their sweetheart agreement. Epidemiologia e prevenzione. 2020;44(56 Supplement 2):169-82.
4	Tomei Torres FA. [Strategies in the prevention and control of the Covid-19 pandemic caused by SARS-CoV-2. Environmental factors.]. Rev Esp Salud Publica. 2020;94.

Supplementary Table S3g: Manuscripts Excluded because they are Pediatric studies	
1	Burgos-Blasco B, Guemes-Villahoz N, Morales-Fernandez L, Callejas-Caballero I, Perez-Garcia P, Donate-Lopez J, et al. Retinal nerve fibre layer and ganglion cell layer changes in children who recovered from COVID-19: A cohort study. Archives of Disease in Childhood. 2022;107(2):175-9.
2	Sahih M, Schultz A, Wilson A, Alakeson R, Taylor E, Mullins B, et al. Paediatric headbox as aerosol and droplet barrier. Archives of Disease in Childhood. 2022;107(1):65-7.
3	Dondi A, Betti L, Carbone C, Dormi A, Paglione M, Rinaldi M, et al. Understanding the environmental factors related to the decrease in Pediatric Emergency Department referrals for acute asthma during the SARS-CoV-2 pandemic. Pediatric Pulmonology. 2022;57(1):66-74.
4	Litchfield IJ, Ayres JG, Jaakkola JJK, Mohammed NI. Is ambient air pollution associated with onset of sudden infant death syndrome: a case-crossover study in the UK. BMJ Open. 2018;8(4):e018341.
5	Guibas GV, Makris M, Papadopoulos NG. Acute asthma exacerbations in childhood: risk factors, prevention and treatment. Expert Rev Respir Med. 2012;6(6):629-38.

Supplementary Table S3h: Manuscripts Excluded because they are Pilot studies/ Study designs	
1	Shahdad S, Patel T, Hindocha A, Cagney N, Mueller JD, Seoudi N, et al. The efficacy of an extraoral scavenging device on reduction of splatter contamination during dental aerosol generating procedures: an exploratory study. British dental journal. 2020;11.
2	Martin-Quintero I, Cervera-Sabater A, Tapias-Perero V, Nieto-Sanchez I, de la Cruz-Perez J. Air particulate concentration during orthodontic procedures: a pilot study. BMC oral health. 2021;21(1):361.
3	Din AR, Hindocha A, Patel T, Sudarshan S, Cagney N, Koched A, et al. Quantitative analysis of particulate matter release during orthodontic procedures: a pilot study. British dental journal. 2020;12.
4	Cennamo G, Reibaldi M, Montorio D, D'Andrea L, Fallico M, Triassi M. Optical Coherence Tomography Angiography Features in Post-COVID-19 Pneumonia Patients: A Pilot Study. American Journal of Ophthalmology. 2021;227:182-90.
5	Barcala-Furelos R, Abelairas-Gomez C, Alonso-Calvete A, Cano-Noguera F, Carballo-Fazanes A, Martinez-Isasi S, et al. Safe On-Boat Resuscitation by Lifeguards in COVID-19 Era: A Pilot Study Comparing Three Sets of Personal Protective Equipment. Prehospital and disaster medicine. 2021;36(2):163-9.
6	Marquès M, Rovira J, Nadal M, Domingo JL. Effects of air pollution on the potential transmission and mortality of COVID-19: A preliminary case-study in Tarragona Province (Catalonia, Spain). Environ Res. 2021;192:110315.
7	Garbey M, Joerger G, Furr S. A Systems Approach to Assess Transport and Diffusion of Hazardous Airborne Particles in a Large Surgical Suite: Potential Impacts on Viral Airborne Transmission. Int J Environ Res Public Health. 2020;17(15).
8	Bogani G, Ditto A, De Cecco L, Lopez S, Guerrisi R, Piccioni F, et al. Transmission of SARS-CoV-2 in Surgical Smoke during Laparoscopy: A Prospective, Proof-of-concept Study. J Minim Invasive Gynecol. 2021;28(8):1519-25.

Supplementary Table S3i: Manuscripts Excluded because they are Review and Meta-analysis	
1	Tanwar V, Adelstein JM, Wold LE. Double trouble: Combined cardiovascular effects of particulate matter exposure and coronavirus disease 2019. Cardiovascular Research. 2021;117(1):85-95.
2	Latorre A, Rothwell JC. Myoclonus and COVID-19: A Challenge for the Present, a Lesson for the Future. Movement Disorders Clinical Practice. 2020;7(8):888-90.
3	Tang C, Ling L, Zhang WX. Visualizing Trace Pollutants in Solids at Nanoscale via Electron Tomography. Environmental Science and Technology. 2021;55(17):11533-7.
4	Dwari K. COVID-19 pandemic: A positive influence on lifestyle, economy, administration & environment; an overview. Indian Journal of Forensic Medicine and Toxicology. 2020;14(4):6605-10.
5	Kunz Y, Horninger W, Pinggera GM. Are urologists in trouble with SARS-CoV-2? Reflections and recommendations for specific interventions. BJU International. 2020;126(6):670-8.
6	Zhu C, Maharajan K, Liu K, Zhang Y. Role of atmospheric particulate matter exposure in COVID-19 and other health risks in human: A review. Environmental Research. 2021;198 (no pagination).
7	Zhao C, Feng Y, Fang X, He J, Pan H. Emerging role of air pollution and meteorological parameters in COVID-19. Journal of Evidence-Based Medicine. 2021;14(2):123-38.
8	Zhang XS, Duchaine C. SARS-CoV-2 and health care worker protection in low-risk settings: A review of modes of transmission and a novel airborne model involving inhalable particles. Clinical Microbiology Reviews. 2020;34(1):1-29.
9	Zhang X, Zhang Z, Xia N, Zhao Q. Carbohydrate-containing nanoparticles as vaccine adjuvants. Expert Review of Vaccines. 2021;20(7):797-810.
10	Zhang J, Zhao C, Zhao W. Virus Caused Imbalance of Type I IFN Responses and Inflammation in COVID-19. Frontiers in Immunology. 2021;12 (no pagination).
11	Zhan J, Liu QS, Sun Z, Zhou Q, Hu L, Qu G, et al. Environmental impacts on the transmission and evolution of COVID-19 combing the knowledge of pathogenic respiratory coronaviruses. Environmental Pollution. 2020;267 (no pagination).
12	Zangari Del Balzo G. Methodological notes on pandemic virus SARS-CoV-2 research. Theory in biosciences = Theorie in den Biowissenschaften. 2021;140(3):279-94.
13	Yousefi B, Banihashemian S, Feyzabadi Z, Hasanpour S, Kokhaei P, Abdolshahi A, et al. Potential therapeutic effect of oxygen-ozone in controlling of COVID-19 disease. Medical Gas Research. 2022;12(2):33-40.
14	Yao Y, Lawrence DA. Susceptibility to COVID-19 in populations with health disparities: Posited involvement of mitochondrial disorder, socioeconomic stress, and pollutants. Journal of Biochemical and Molecular Toxicology. 2021;35(1) (no pagination).
15	Xu Y, Zhuang Y, Kang L. A Review of Neurological Involvement in Patients with SARS-CoV-2 Infection. Medical Science Monitor. 2021;27 (no pagination).
16	Wu J, Tang Y. Revisiting the Immune Balance Theory: A Neurological Insight Into the Epidemic of COVID-19 and Its Alike. Frontiers in Neurology. 2020;11 (no pagination).
17	Woodby B, Arnold MM, Valacchi G. SARS-CoV-2 infection, COVID-19 pathogenesis, and exposure to air pollution: What is the connection? Annals of the New York Academy of Sciences. 2021;1486(1):15-38.
18	Widiasta A, Sribudiani Y, Nugrahapraja H, Hilmanto D, Sekarwana N, Rachmadi D. Potential role of ACE2-related microRNAs in COVID-19-associated nephropathy. Non-coding RNA Research. 2020;5(4):153-66.
19	Villeneuve PJ, Goldberg MS. Methodological considerations for epidemiological studies of air pollution and the sars and COVID-19 coronavirus outbreaks. Environmental Health Perspectives. 2020;128(9):095001-1--13.
20	Tsatsakis A, Petrakis D, Nikolouzakis TK, Docea AO, Calina D, Vinceti M, et al. COVID-19, an opportunity to reevaluate the correlation between long-term effects of anthropogenic pollutants on viral epidemic/pandemic events and prevalence. Food and Chemical Toxicology. 2020;141 (no pagination).
21	Tiwari SK, Dicks LMT, Popov IV, Karaseva A, Ermakov AM, Suvorov A, et al. Probiotics at War Against Viruses: What Is Missing From the Picture? Frontiers in Microbiology. 2020;11 (no pagination).
22	Tivey DR, Davis SS, Kovoor JG, Babidge WJ, Tan L, Hugh TJ, et al. Safe surgery during the coronavirus disease 2019 crisis. ANZ journal of surgery. 2020;90(9):1553-7.
23	Thakur AK, Sathyamurthy R, Ramalingam V, Lynch I, Sharshir SW, Ma Z, et al. A case study of SARS-CoV-2 transmission behavior in a severely air-polluted city (Delhi, India) and the potential usage of graphene based materials for filtering air-pollutants and controlling/monitoring the COVID-19 pandemic. Environmental Science: Processes and Impacts. 2021;23(7):923-46.
24	Tang Y, Hu L, Liu Y, Zhou B, Qin X, Ye J, et al. Possible mechanisms of cholesterol elevation aggravating covid-19. International Journal of Medical Sciences. 2021;18(15):3533-43.
25	Tamama K. Potential benefits of dietary seaweeds as protection against COVID-19. Nutrition Reviews. 2021;79(7):814-23.
26	Suthar S, Das S, Nagpure A, Madhurantakam C, Tiwari SB, Gahlot P, et al. Epidemiology and diagnosis, environmental resources quality and socio-economic perspectives for COVID-19 pandemic. Journal of Environmental Management. 2021;280 (no pagination).
27	Sullivan JR, Rademaker M, Goodman G, Bekhor P, Al-Niaimi F. Guidance on infection control and plume management with Laser and Energy-Based Devices taking into consideration COVID-19. Australasian Journal of Dermatology. 2021;62(1):37-40.

28	Srivastava A. COVID-19 and air pollution and meteorology-an intricate relationship: A review. Chemosphere. 2021;263 (no pagination).
29	Sivaraman D, Pradeep PS, Sundar Manoharan S, Ramachandra Bhat C, Leela KV, Venugopal V. Current strategies and approaches in combating SARS-CoV-2 virus that causes COVID-19. Letters in Drug Design and Discovery. 2020;17(5):670-2.
30	Silva ACT, Branco PTBS, Sousa SIV. Impact of COVID-19 Pandemic on Air Quality: A Systematic Review. International Journal of Environmental Research and Public Health. 2022;19(4) (no pagination).
31	Shokri-Mashhadi N, Kazemi M, Saadat S, Moradi S. Effects of select dietary supplements on the prevention and treatment of viral respiratory tract infections: a systematic review of randomized controlled trials. Expert Review of Respiratory Medicine. 2021;15(6):805-21.
32	Shekhtman B, Rizk T, Siebenaler C. COVID-19 and guillain-barre: A case report and literature review. PM and R. 2021;13:S124.
33	Shah SM, Alsaab HO, Rawas-Qalaji MM, Uddin MN. A review on current covid-19 vaccines and evaluation of particulate vaccine delivery systems. Vaccines. 2021;9(10) (no pagination).
34	Setti L, Passarini F, De Gennaro G, Barbieri P, Perrone MG, Borelli M, et al. Airborne transmission route of covid-19: Why 2 meters/6 feet of inter-personal distance could not be enough. International Journal of Environmental Research and Public Health. 2020;17(8) (no pagination).
35	Setti L, Passarini F, De Gennaro G, Barbieri P, Pallavicini A, Ruscio M, et al. Searching for SARS-COV-2 on particulate matter: A possible early indicator of COVID-19 epidemic recurrence. International Journal of Environmental Research and Public Health. 2020;17(9) (no pagination).
36	Scuto M, Trovato Salinaro A, Caligiuri I, Ontario ML, Greco V, Sciuto N, et al. Redox modulation of vitagenes via plant polyphenols and vitamin D: Novel insights for chemoprevention and therapeutic interventions based on organoid technology. Mechanisms of Ageing and Development. 2021;199 (no pagination).
37	Santurtun A, Colom ML, Fdez-Arroyabe P, Real AD, Fernandez-Olmo I, Zarrabeitia MT. Exposure to particulate matter: Direct and indirect role in the COVID-19 pandemic. Environmental Research. 2022;206 (no pagination).
38	SanJuan-Reyes S, Gomez-Olivan LM, Islas-Flores H. COVID-19 in the environment. Chemosphere. 2021;263 (no pagination).
39	Sanjay S, Agrawal S, Jayadev C, Kawali A, Gowda PB, Shetty R, et al. Posterior segment manifestations and imaging features post-COVID-19. Medical Hypothesis, Discovery, and Innovation in Ophthalmology. 2021;10(3):95-106.
40	Sakamoto K, Ozaki T, Kadomatsu K. Axonal Regeneration by Glycosaminoglycan. Frontiers in Cell and Developmental Biology. 2021;9 (no pagination).
41	Sajdel-Sulkowska EM. Neuropsychiatric Ramifications of COVID-19: Short-Chain Fatty Acid Deficiency and Disturbance of Microbiota-Gut-Brain Axis Signaling. BioMed Research International. 2021;2021 (no pagination).
42	Sadeghmousavi S, Rezaei N. COVID-19 and Multiple Sclerosis: Predisposition and Precautions in Treatment. SN Comprehensive Clinical Medicine. 2020;2(10):1802-7.
43	Sabahgoulian CB, Manvelyan HM. Clinical observation of rare neurological complications of covid-19: Acute demyelinating polyneuropathy and critical illness neuropathy. New Armenian Medical Journal. 2021;15(3):16-21.
44	Rishi P, Thakur K, Vij S, Rishi L, Singh A, Kaur IP, et al. Diet, Gut Microbiota and COVID-19. Indian Journal of Microbiology. 2020;60(4):420-9.
45	Reyes MSS, Medina PMB. Environmental pollutant exposure can exacerbate COVID-19 neurologic symptoms. Medical Hypotheses. 2020;144 (no pagination).
46	Rabaan AA, Al-Ahmed SH, Al-Malkey MK, Alsubki RA, Ezzikouri S, Al-Hababi FH, et al. Airborne transmission of SARS-CoV-2 is the dominant route of transmission: Droplets and aerosols. Infezioni in Medicina. 2021;29(1):10-9.
47	Qu JH, Leirs K, Maes W, Imbrechts M, Callewaert N, Lagrou K, et al. Innovative FO-SPR Label-free Strategy for Detecting Anti-RBD Antibodies in COVID-19 Patient Serum and Whole Blood. ACS sensors. 2022;21.
48	Poyraz BM, Engin ED, Engin AB, Engin A. The effect of environmental diesel exhaust pollution on SARS-CoV-2 infection: The mechanism of pulmonary ground glass opacity. Environmental Toxicology and Pharmacology. 2021;86 (no pagination).
49	Petrarca C, Angelucci M, Pedata P, Bramante F, Mangifesta R. Environment and COVID: the consequence of exposure to air pollutants on the pathology of SARS-COV-2. Journal of Biological Regulators and Homeostatic Agents. 2021;35(Supplement 2):1-8.
50	Pajo AT, Espiritu AI, Apor ADAO, Jamora RDG. Neuropathologic findings of patients with COVID-19: a systematic review. Neurological Sciences. 2021;42(4):1255-66.
51	Paital B, Das K. Spike in pollution to ignite the bursting of COVID-19 second wave is more dangerous than spike of SAR-CoV-2 under environmental ignorance in long term: a review. Environmental science and pollution research international. 2021;14.
52	Ng WF, To KF, Lam WWL, Ng TK, Lee KC. The comparative pathology of severe acute respiratory syndrome and avian influenza A subtype H5N1 - A review. Human Pathology. 2006;37(4):381-90.
53	Navarro KM, Clark KA, Hardt DJ, Reid CE, Lahm PW, Domitrovich JW, et al. Wildland firefighter exposure to smoke and COVID-19: A new risk on the fire line. Science of the Total Environment. 2021;760 (no pagination).
54	Nahama A, Ramachandran R, Cisternas AF, Ji H. The role of afferent pulmonary innervation in ARDS associated with COVID-19 and potential use of resiniferatoxin to improve prognosis: A review. Medicine in Drug Discovery. 2020;5 (no pagination).

55	Nagu P, Parashar A, Behl T, Mehta V. CNS implications of COVID-19: A comprehensive review. Reviews in the Neurosciences. 2021;32(2):219-34.
56	Moelling K, Broecker F. Air Microbiome and Pollution: Composition and Potential Effects on Human Health, including SARS Coronavirus Infection. Journal of Environmental and Public Health. 2020;2020 (no pagination).
57	Min YG, Ju W, Ha YE, Ban JJ, Lee SA, Sung JJ, et al. Sensory Guillain-Barre syndrome following the ChAdOx1 nCov-19 vaccine: Report of two cases and review of literature. Journal of Neuroimmunology. 2021;359 (no pagination).
58	Meo SA, Al-Khlaiwi T, Ullah CH. Effect of ambient air pollutants PM2.5 and PM10 on COVID-19 incidence and mortality: Observational study. European Review for Medical and Pharmacological Sciences. 2021;25(23):7553-64.
60	Mehmel M, Jovanovic N, Spitz U. Nicotinamide riboside-the current state of research and therapeutic uses. Nutrients. 2020;12(6) (no pagination).
61	Marwah M, Agrawala PK. COVID-19 lockdown and environmental pollution: an Indian multi-state investigation. Environmental Monitoring and Assessment. 2022;194(2) (no pagination).
62	Maleki M, Anvari E, Hopke PK, Noorimotlagh Z, Mirzaee SA. An updated systematic review on the association between atmospheric particulate matter pollution and prevalence of SARS-CoV-2. Environmental Research. 2021;195 (no pagination).
63	Maheswari S, Pethannan R, Sabarimurugan S. Air pollution enhances susceptibility to novel coronavirus (COVID-19) infection - An impact study. Environmental Health and Toxicology. 2020;35(4):1-7.
64	Madurai Elavarasan R, Pugazhendhi R. Restructured society and environment: A review on potential technological strategies to control the COVID-19 pandemic. Science of the Total Environment. 2020;725 (no pagination).
65	Lubrano C, Risi R, Masi D, Gnessi L, Colao A. Is obesity the missing link between COVID-19 severity and air pollution? Environmental Pollution. 2020;Part 3. 266 (no pagination).
66	Liu DT, Phillips KM, Speth M, Besser G, Mueller CA, Sedaghat AR. Portable HEPA purifiers to eliminate airborne SARS-CoV-2: A systematic review. Otolaryngology - Head and Neck Surgery. 2021;165(1 SUPPL):P140.
67	Li W, Joshi MD, Singhania S, Ramsey KH, Murthy AK. Peptide vaccine: Progress and challenges. Vaccines. 2014;2(3):515-36.
68	Lai A, Chang ML, O'Donnell RP, Zhou C, Sumner JA, Hsiai TK. Association of COVID-19 transmission with high levels of ambient pollutants: Initiation and impact of the inflammatory response on cardiopulmonary disease. Science of the Total Environment. 2021;779 (no pagination).
69	Krajewska J, Krajewski W, Zub K, Zatonski T. Review of practical recommendations for otolaryngologists and head and neck surgeons during the COVID-19 pandemic. Auris Nasus Larynx. 2020;47(4):544-58.
70	Koike H, Chiba A, Katsuno M. Emerging Infection, Vaccination, and Guillain-Barre Syndrome: A Review. Neurology and Therapy. 2021;10(2):523-37.
71	Kma L. Transforming growth factor-beta1 as lung injury biomarker: A review. International Journal of Life Sciences Biotechnology and Pharma Research. 2013;2(3):39-58.
72	Kim HS. Do an altered gut microbiota and an associated leaky gut affect COVID-19 severity? mBio. 2021;12(1):1-9.
73	Khatoon F, Prasad K, Kumar V. COVID-19 associated nervous system manifestations. Sleep Medicine. 2021.
74	Khan T, Withers C, Martin E, Bonilla N. Approaches for effective negative pressure isolation space control to minimize airborne transmission of contaminants in residential homes. Indoor and Built Environment. 2022.
75	Kerboua KE. The perplexing question of trained immunity vs adaptive memory in COVID-19. Journal of Medical Virology. 2020;92(10):1858-63.
76	Kaya K, Khalil M, Fetrow B, Fritz H, Jagadesan P, Bondu V, et al. Rapid and Effective Inactivation of SARS-CoV-2 with a Cationic Conjugated Oligomer with Visible Light: Studies of Antiviral Activity in Solutions and on Supports. ACS applied materials & interfaces. 2022;14(4):4892-8.
78	Jones RM, Brosseau LM. Aerosol transmission of infectious disease. Journal of Occupational and Environmental Medicine. 2015;57(5):501-8.
79	Jain A, Talwar D, Kumar S. Spectrum of respiratory involvement in COVID 19 era; an overview. Indian Journal of Forensic Medicine and Toxicology. 2020;14(4):6593-9.
80	Jai Rexlin PE, Anjali AK, Roy A. Has lockdown led to the restoration of nature-a review. International Journal of Pharmaceutical Research. 2021;13(1):1653-8.
81	Islam MT, Hossen M, Kamaz Z, Zali A, Kumar M, Docea AO, et al. The role of hmgb1 in the immune response to sars-cov-2 infection: From pathogenesis towards a new potential therapeutic target. Farmacia. 2021;69(4):621-34.
82	Huang J, Zheng M, Tang X, Chen Y, Tong A, Zhou L. Potential of SARS-CoV-2 to Cause CNS Infection: Biologic Fundamental and Clinical Experience. Frontiers in Neurology. 2020;11 (no pagination).
83	Hu L, Deng WJ, Ying GG, Hong H. Environmental perspective of COVID-19: Atmospheric and wastewater environment in relation to pandemic. Ecotoxicology and Environmental Safety. 2021;219 (no pagination).
84	Helm S, Harmon PC, Noe C, Calodney A, Abd-Elsayed A, Knezevic NN, et al. Transforaminal epidural steroid injections: A systematic review and meta-analysis of efficacy and safety. Pain Physician. 2021;24(S1):209-32.
85	He LH, Ren LF, Li JF, Wu YN, Li X, Zhang L. Intestinal Flora as a Potential Strategy to Fight SARS-CoV-2 Infection. Frontiers in Microbiology. 2020;11 (no pagination).
86	Hawrylkowicz V, Lietz-Kijak D, Kazmierczak-Siedlecka K, Solek-Pastuszka J, Stachowska L, Folwarski M, et al. Patient nutrition and probiotic therapy in covid-19: What do we know in 2021? Nutrients. 2021;13(10) (no pagination).

87	Hammond A, Khalid T, Thornton HV, Woodall CA, Hay AD. Should homes and workplaces purchase portable air filters to reduce the transmission of SARS-CoV-2 and other respiratory infections? A systematic review. PLoS ONE. 2021;16(4 April 2021) (no pagination).
88	Gola M, Caggiano G, De Giglio O, Napoli C, Diella G, Carlucci M, et al. SARS-CoV-2 indoor contamination: considerations on anti-COVID-19 management of ventilation systems, and finishing materials in healthcare facilities. Annali di igiene : medicina preventiva e di comunita. 2021;33(4):381-92.
89	Ghoshal U, Vasanth S, Tejan N. A guide to laboratory diagnosis of Corona Virus Disease-19 for the gastroenterologists. Indian Journal of Gastroenterology. 2020;39(3):236-42.
90	Gettings J, Czarnik M, Morris E, Haller E, Thompson-Paul AM, Rasberry C, et al. Mask Use and Ventilation Improvements to Reduce COVID-19 Incidence in Elementary Schools - Georgia, November 16-December 11, 2020. Mmwr. 2021;Morbidity and mortality weekly report. 70(21):779-84.
91	Generoso JS, de Quevedo JLB, Cattani M, Lodetti BF, Sousa L, Collodel A, et al. Neurobiology of COVID-19: how can the virus affect the brain? Brazilian Journal of Psychiatry. 2021;43(6):650-64.
92	Garg RK, Paliwal VK. Spectrum of neurological complications following COVID-19 vaccination. Neurological Sciences. 2022;43(1):3-40.
93	Gao YD, Ding M, Dong X, Zhang JJ, Kursat Azkur A, Azkur D, et al. Risk factors for severe and critically ill COVID-19 patients: A review. Allergy: European Journal of Allergy and Clinical Immunology. 2021;76(2):428-55.
94	Forouzandeh P, O'Dowd K, Pillai SC. Face masks and respirators in the fight against the COVID-19 pandemic: An overview of the standards and testing methods. Safety Science. 2021;133 (no pagination).
95	Foladori P, Cutrupi F, Cadonna M, Manara S. Coronaviruses and SARS-CoV-2 in sewerage and their removal: Step by step in wastewater treatment plants. Environmental Research. 2022;207 (no pagination).
96	Fiorito S, Soligo M, Gao Y, Ogulur I, Akdis CA, Bonini S. Is epithelial barrier hypothesis the key to understanding the higher incidence and excess mortality during COVID-19 pandemic? The case of Northern Italy. Allergy: European Journal of Allergy and Clinical Immunology. 2022.
97	Finsterer J. Neurological side effects of SARS-CoV-2 vaccinations. Acta Neurologica Scandinavica. 2022;145(1):5-9.
98	Fernandez-Quintela A, Milton-Laskibar I, Trepiana J, Gomez-Zorita S, Kajarabille N, Leniz A, et al. Key aspects in nutritional management of covid-19 patients. Journal of Clinical Medicine. 2020;9(8):1-24.
99	Fedullo AL, Schiattarella A, Morlando M, Raguzzini A, Toti E, De Franciscis P, et al. Mediterranean diet for the prevention of gestational diabetes in the covid-19 era: Implications of Il-6 in diabesity. International Journal of Molecular Sciences. 2021;22(3):1-22.
100	Eke UA, Eke AC. Personal protective equipment in the siege of respiratory viral pandemics: strides made and next steps. Expert Review of Respiratory Medicine. 2021;15(4):441-52.
101	Ehtezazi T. The Potential Use of Cyclosporine Ultrafine Solution Pressurised Metered-Dose Inhaler in the Treatment of COVID-19 Patients. Recent advances in drug delivery and formulation. 2021;21.
102	Duval JFL, van Leeuwen HP, Norde W, Town RM. Chemodynamic features of nanoparticles: Application to understanding the dynamic life cycle of SARS-CoV-2 in aerosols and aqueous biointerfacial zones. Advances in colloid and interface science. 2021;290:102400.
103	Donia A, Hassan SU, Zhang X, Al-Madboly L, Bokhari H. Covid-19 crisis creates opportunity towards global monitoring & surveillance. Pathogens. 2021;10(3):1-28.
104	Domingo JL, Rovira J. Effects of air pollutants on the transmission and severity of respiratory viral infections. Environmental Research. 2020;187 (no pagination).
105	Domingo JL, Marques M. The effects of some essential and toxic metals/metalloids in COVID-19: A review. Food and Chemical Toxicology. 2021;152 (no pagination).
106	Dolatshahi M, Sabahi M, Aarabi MH. Pathophysiological Clues to How the Emergent SARS-CoV-2 Can Potentially Increase the Susceptibility to Neurodegeneration. Molecular Neurobiology. 2021;58(5):2379-94.
107	Demeke Teklemariam A, Samaddar M, Alharbi MG, Al-Hindi RR, Bhunia AK. Biosensor and molecular-based methods for the detection of human coronaviruses: A review. Molecular and Cellular Probes. 2020;54 (no pagination).
108	De Matteis S, Forastiere F, Baldacci S, Maio S, Tagliaferro S, Fasola S, et al. Issue 1 - "Update on adverse respiratory effects of outdoor air pollution". Part 1): Outdoor air pollution and respiratory diseases: A general update and an Italian perspective. Pulmonology. 2022.
109	de Albuquerque TL, Marques Junior JE, de Queiroz LP, Ricardo ADS, Rocha MVP. Polylactic acid production from biotechnological routes: A review. International Journal of Biological Macromolecules. 2021;186:933-51.
110	Datta M, Singh DD, Naqvi AR. Molecular Diagnostic Tools for the Detection of SARS-CoV-2. International Reviews of Immunology. 2021;40(1-2):143-56.
111	Daoud AK, Hall JK, Petrick H, Strong A, Piggott C. The Potential for Cloth Masks to Protect Health Care Clinicians From SARS-CoV-2: A Rapid Review. Annals of family medicine. 2021;19(1):55-62.
112	Copat C, Cristaldi A, Fiore M, Grasso A, Zuccarello P, Signorelli SS, et al. The role of air pollution (PM and NO<inf>2</inf>) in COVID-19 spread and lethality: A systematic review. Environmental Research. 2020;191 (no pagination).
113	Collins DB, Farmer DK. Unintended Consequences of Air Cleaning Chemistry. Environmental Science and Technology. 2021;55(18):12172-9.
114	Charitos IA, Ballini A, Bottalico L, Cantore S, Passarelli PC, Inchingolo F, et al. Special features of SARS-CoV-2 in daily practice. World Journal of Clinical Cases. 2020;8(18):3920-33.

115	Calderon-Garciduenas L, Torres-Jardon R, Franco-Lira M, Kulesza R, Gonzalez-Maciel A, Reynoso-Robles R, et al. Environmental Nanoparticles, SARS-CoV-2 Brain Involvement, and Potential Acceleration of Alzheimer's and Parkinson's Diseases in Young Urbanites Exposed to Air Pollution. Advances in Alzheimer's Disease. 2020;8:567-91.
116	Bratosiewicz-Wasik J. Neuro-COVID-19: an insidious virus in action. Neurologia i neurochirurgia polska. 2021;13.
117	Brandt EB, Beck AF, Mersha TB. Air pollution, racial disparities, and COVID-19 mortality. Journal of Allergy and Clinical Immunology. 2020;146(1):61-3.
118	Boraldi F, Lofaro FD, Cossarizza A, Quaglino D. The "Elastic Perspective" of SARS-CoV-2 Infection and the Role of Intrinsic and Extrinsic Factors. International Journal of Molecular Sciences. 2022;23(3) (no pagination).
119	Bondy SC, Wu M, Prasad KN. Attenuation of acute and chronic inflammation using compounds derived from plants. Experimental Biology and Medicine. 2021;246(4):406-13.
120	Bodnar B, Patel K, Ho W, Luo JJ, Hu W. Cellular mechanisms underlying neurological/neuropsychiatric manifestations of COVID-19. Journal of Medical Virology. 2021;93(4):1983-98.
121	Beesoon S, Behary N, Perwuelz A. Universal masking during COVID-19 pandemic: Can textile engineering help public health? Narrative review of the evidence. Preventive Medicine. 2020;139 (no pagination).
122	Babaahmadi V, Amid H, Naeimirad M, Ramakrishna S. Biodegradable and multifunctional surgical face masks: A brief review on demands during COVID-19 pandemic, recent developments, and future perspectives. Science of the Total Environment. 2021;798 (no pagination).
123	Atiyani R, Mustafa S, Alsari S, Darwish A, Janahi EM. Clearing the air about airborne transmission of SARS-CoV-2. European Review for Medical and Pharmacological Sciences. 2021;25(21):6745-66.
124	Assaf SM, Tarasevych SP, Diamant Z, Hanania NA. Asthma and severe acute respiratory syndrome coronavirus 2019: Current evidence and knowledge gaps. Current Opinion in Pulmonary Medicine. 2021;27(1):45-53.
125	Ansori ANM. A mini-review of the medicinal properties of Okra (Abelmoschus esculentus l.) and potential benefit against SARS-CoV-2. Indian Journal of Forensic Medicine and Toxicology. 2021;15(1):852-6.
126	Anand U, Adelodun B, Pivato A, Suresh S, Indari O, Jakhmola S, et al. A review of the presence of SARS-CoV-2 RNA in wastewater and airborne particulates and its use for virus spreading surveillance. Environmental Research. 2021;196 (no pagination).
127	Alqadi GO, Saxena AK. Smoke and particulate filters in endoscopic surgery reviewed during COVID-19 pandemic. Journal of Pediatric Endoscopic Surgery. 2020;2(2):61-7.
128	Ali N, Fariha KA, Islam F, Mishu MA, Mohanto NC, Hosen MJ, et al. Exposure to air pollution and COVID-19 severity: A review of current insights, management, and challenges. Integrated environmental assessment and management. 2021;17(6):1114-22.
129	Alava JJ, Singh GG. Changing air pollution and CO<inf>2</inf> emissions during the COVID-19 pandemic: Lesson learned and future equity concerns of post-COVID recovery. Environmental Science and Policy. 2022;130:1-8.
130	Ram K, Thakur RC, Singh DK, Kawamura K, Shimouchi A, Sekine Y, et al. Why airborne transmission hasn't been conclusive in case of COVID-19? An atmospheric science perspective. Science of the Total Environment. 2021;773 (no pagination).
131	Zhang Z, Ji D, He H, Ramakrishna S. Electrospun ultrafine fibers for advanced face masks. Mater Sci Eng R Rep. 2021;143:100594.
132	Wang Y, Xue Q. The implications of COVID-19 in the ambient environment and psychological conditions. NanoImpact. 2021;21:100295.
133	Tung NT, Cheng PC, Chi KH, Hsiao TC, Jones T, BéruBé K, et al. Particulate matter and SARS-CoV-2: A possible model of COVID-19 transmission. Sci Total Environ. 2021;750:141532.
134	Stevens KA, Bryer TA, Yu H. Air Quality Enhancement Districts: democratizing data to improve respiratory health. J Environ Stud Sci. 2021;11(4):702-7.
135	Senatore V, Zarra T, Buonerba A, Choo KH, Hasan SW, Korshin G, et al. Indoor versus outdoor transmission of SARS-COV-2: environmental factors in virus spread and underestimated sources of risk. EuroMediterr J Environ Integr. 2021;6(1):30.
136	Robertson-More C, Wu T. A knowledge gap unmasked: viral transmission in surgical smoke: a systematic review. Surg Endosc. 2021;35(6):2428-39.
137	Rebuli ME, Brocke SA, Jaspers I. Impact of inhaled pollutants on response to viral infection in controlled exposures. J Allergy Clin Immunol. 2021;148(6):1420-9.
138	Ray RL, Singh VP, Singh SK, Acharya BS, He Y. What is the impact of COVID-19 pandemic on global carbon emissions? Sci Total Environ. 2022;816:151503.
139	Pavan N, Crestani A, Abrate A, De Nunzio C, Esperto F, Giannarini G, et al. Risk of Virus Contamination Through Surgical Smoke During Minimally Invasive Surgery: A Systematic Review of the Literature on a Neglected Issue Revived in the COVID-19 Pandemic Era. Eur Urol Focus. 2020;6(5):1058-69.
140	Pasquier J, Villalta O, Sarria Lamorú S, Balagué C, Vilallonga R, Targarona EM. Are Smoke and Aerosols Generated During Laparoscopic Surgery a Biohazard? A Systematic Evidence-Based Review. Surg Innov. 2021;28(4):485-95.
141	Paital B, Agrawal PK. Air pollution by NO(2) and PM(2.5) explains COVID-19 infection severity by overexpression of angiotensin-converting enzyme 2 in respiratory cells: a review. Environ Chem Lett. 2021;19(1):25-42.

142	Olloquequi J. COVID-19 Susceptibility in chronic obstructive pulmonary disease. Eur J Clin Invest. 2020;50(10):e13382.
143	Mukherjee S, Boral S, Siddiqi H, Mishra A, Meikap BC. Present cum future of SARS-CoV-2 virus and its associated control of virus-laden air pollutants leading to potential environmental threat - A global review. J Environ Chem Eng. 2021;9(2):104973.
144	Mintz Y, Arezzo A, Boni L, Baldari L, Cassinotti E, Brodie R, et al. The risk of COVID-19 transmission by laparoscopic smoke may be lower than for laparotomy: a narrative review. Surg Endosc. 2020;34(8):3298-305.
145	Marquès M, Domingo JL. Positive association between outdoor air pollution and the incidence and severity of COVID-19. A review of the recent scientific evidences. Environ Res. 2022;203:111930.
146	Ma Q, Qi Y, Shan Q, Liu S, He H. Understanding the knowledge gaps between air pollution controls and health impacts including pathogen epidemic. Environ Res. 2020;189:109949.
147	Katoto P, Brand AS, Bakan B, Obadia PM, Kuhangana C, Kayembe-Kitenge T, et al. Environ Health. 2021;20(1):41.
148	Karan A, Ali K, Teelucksingh S, Sakhamuri S. The impact of air pollution on the incidence and mortality of COVID-19. Glob Health Res Policy. 2020;5:39.
149	Ishmatov A. "SARS-CoV-2 is transmitted by particulate air pollution": Misinterpretations of statistical data, skewed citation practices, and misuse of specific terminology spreading the misconception. Environ Res. 2022;204(Pt B):112116.
150	Gallo O. Risk for COVID-19 infection in patients with tobacco smoke-associated cancers of the upper and lower airway. Eur Arch Otorhinolaryngol. 2021;278(8):2695-702.
151	Gallo M, Street ME, Guerra F, Fanos V, Marcialis MA. A review of current knowledge on Pollution, Cigarette Smoking and COVID-19 diffusion and their relationship with inflammation. Acta Biomed. 2020;91(4):e2020148.
152	Feng S, Jia C, Liu Z, Lyu X. [Advances in the research of pathogenesis and treatment of severe smoke inhalation injury]. Zhonghua Shao Shang Za Zhi. 2016;32(2):122-5.
153	Félix-Arellano EE, Schilmann A, Hurtado-Díaz M, Texcalac-Sangrado JL, Riojas-Rodríguez H. [Quick review: air pollution and morbi-mortalityby Covid-19.]. Salud Publica Mex. 2020;62(5):582-9.
154	Fattorini D, Regoli F. Role of the chronic air pollution levels in the Covid-19 outbreak risk in Italy. Environ Pollut. 2020;264:114732.
155	Domingo JL, Marquès M, Rovira J. Influence of airborne transmission of SARS-CoV-2 on COVID-19 pandemic. A review. Environ Res. 2020;188:109861.
156	Delikhoon M, Guzman MI, Nabizadeh R, Norouzian Baghani A. Modes of Transmission of Severe Acute Respiratory Syndrome-Coronavirus-2 (SARS-CoV-2) and Factors Influencing on the Airborne Transmission: A Review. Int J Environ Res Public Health. 2021;18(2).
157	da Costa KM, Saxena AK. Coronavirus disease 2019 pandemic and identifying insufflators with desufflation mode and surgical smoke evacuators for safe CO(2) removal. Asian J Endosc Surg. 2021;14(2):165-9.
158	Curtis L. PM(2.5), NO(2), wildfires, and other environmental exposures are linked to higher Covid 19 incidence, severity, and death rates. Environ Sci Pollut Res Int. 2021;28(39):54429-47.
159	Comunian S, Dongo D, Milani C, Palestini P. Air Pollution and Covid-19: The Role of Particulate Matter in the Spread and Increase of Covid-19's Morbidity and Mortality. Int J Environ Res Public Health. 2020;17(12).
160	Cheruiyot I, Sehmi P, Ngure B, Misiani M, Karau P, Olabu B, et al. Laparoscopic surgery during the COVID-19 pandemic: detection of SARS-COV-2 in abdominal tissues, fluids, and surgical smoke. Langenbecks Arch Surg. 2021;406(4):1007-14.
161	Chennakesavulu K, Reddy GR. The effect of latitude and PM(2.5) on spreading of SARS-CoV-2 in tropical and temperate zone countries. Environ Pollut. 2020;266(Pt 3):115176.
162	Chen B, Jia P, Han J. Role of indoor aerosols for COVID-19 viral transmission: a review. Environ Chem Lett. 2021;19(3):1953-70.
163	Brandt EB, Mersha TB. Environmental Determinants of Coronavirus Disease 2019 (COVID-19). Curr Allergy Asthma Rep. 2021;21(3):15.
164	Borisova T, Komisarenko S. Air pollution particulate matter as a potential carrier of SARS-CoV-2 to the nervous system and/or neurological symptom enhancer: arguments in favor. Environ Sci Pollut Res Int. 2021;28(30):40371-7.
165	Barakat T, Muylkens B, Su BL. Is Particulate Matter of Air Pollution a Vector of Covid-19 Pandemic? Matter. 2020;3(4):977-80.
166	Anand U, Cabreros C, Mal J, Ballesteros F, Jr., Sillanpää M, Tripathi V, et al. Novel coronavirus disease 2019 (COVID-19) pandemic: From transmission to control with an interdisciplinary vision. Environ Res. 2021;197:111126.
167	Amoatey P, Omidvarborna H, Baawain MS, Al-Mamun A. Impact of building ventilation systems and habitual indoor incense burning on SARS-CoV-2 virus transmissions in Middle Eastern countries. Sci Total Environ. 2020;733:139356.
168	Abrams EM, Sinha I, Fernandes RM, Hawcutt DB. Pediatric asthma and COVID-19: The known, the unknown, and the controversial. Pediatr Pulmonol. 2020;55(12):3573-8.
169	Menendez JA. Metformin and SARS-CoV-2: mechanistic lessons on air pollution to weather the cytokine/thrombotic storm in COVID-19. Aging. 2020;12(10):8760-5.
170	Espejo W, Celis JE, Chiang G, Bahamonde P. Environment and COVID-19: Pollutants, impacts, dissemination, management and recommendations for facing future epidemic threats. Science of the Total Environment. 2020;747 (no pagination).

Supplementary Table S3j: Manuscripts Excluded because they are Unrelated	
1	Deschasaux-Tanguy M, Srour B, Bourhis L, Arnault N, Druesne-Pecollo N, Esseddik Y, et al. Nutritional risk factors for SARS-CoV-2 infection: a prospective study within the NutriNet-Sante cohort. BMC Medicine. 2021;19(1) (no pagination).
2	Sundaram ME, Calzavara A, Mishra S, Kustra R, Chan AK, Hamilton MA, et al. Individual and social determinants of sars-cov-2 testing and positivity in ontario, canada: A population-wide study. Cmaj. 2021;193(20):E723-E34.
3	Li M, Yang Y, He T, Wei R, Qi T, Han T, et al. Detection of SARS-CoV-2 in the ocular surface in different phases of COVID-19 patients in Shanghai, China. Annals of Translational Medicine. 2021;9(2) (no pagination).
4	Bazzazpour S, Rahmatinia M, Mohebbi SR, Hadei M, Shahsavani A, Hopke PK, et al. The detection of SARS-CoV-2 RNA in indoor air of dental clinics during the COVID-19 pandemic. Environmental science and pollution research international. 2021;03.
5	Beni AN, Dehghani A, Kianersi F, Ghanbari H, Habibi Z, Memarzadeh E, et al. Retinal findings of COVID-19 patients using Ocular coherence tomography angiography two to three months after infection: Ocular appearance recovered COVID-19 patient. Photodiagnosis and photodynamic therapy. 2022:102726.
6	Zoran MA, Savastru RS, Savastru DM, Tautan MN, Baschir LA, Tenciu DV. Assessing the impact of air pollution and climate seasonality on COVID-19 multiwaves in Madrid, Spain. Environmental Research. 2022;203 (no pagination).
7	Zor KR, Yildirim Bicer G, Tutas Gunaydin N, Kucuk E, Yilmaz U. Can the coronavirus disease 2019 (COVID-19) cause choroiditis and optic neuropathy? European Journal of Inflammation. 2021;19(no pagination).
8	Zhao M, Liu Y, Gyilbag A. Assessment of Meteorological Variables and Air Pollution Affecting COVID-19 Cases in Urban Agglomerations: Evidence from China. International Journal of Environmental Research and Public Health. 2022;19(1) (no pagination).
9	Zhang S, Chen S, Xiao G, Zhao M, Li J, Dong W, et al. The associations between air pollutant exposure and neutralizing antibody titers of an inactivated SARS-CoV-2 vaccine. Environmental science and pollution research international. 2022;29(9):13720-8.
10	Zhang R, Zhao H. Small-Angle Particle Counting Coupled Photometry for Real-Time Detection of Respirable Particle Size Segmentation Mass Concentration. Sensors. 2021;21(17).
11	Zangmeister CD, Radney JG, Vicenzi EP, Weaver JL. Filtration Efficiencies of Nanoscale Aerosol by Cloth Mask Materials Used to Slow the Spread of SARS-CoV-2. ACS nano. 2020;14(7):9188-200.
12	Yapici-Eser H, Koroglu YE, Oztop-Cakmak O, Keskin O, Gursoy A, Gursoy-Ozdemir Y. Neuropsychiatric Symptoms of COVID-19 Explained by SARS-CoV-2 Proteins' Mimicry of Human Protein Interactions. Frontiers in Human Neuroscience. 2021;15 (no pagination).
13	Yang Y, Liu J, Zhou X. A CRISPR-based and post-amplification coupled SARS-CoV-2 detection with a portable evanescent wave biosensor. Biosensors and Bioelectronics. 2021;190 (no pagination).
14	yang H, Sun G, Tang F, Peng M, Gao Y, Peng J, et al. Clinical features and outcomes of pregnant women suspected of coronavirus disease 2019. Journal of Infection. 2020;81(1):e40-e4.
15	Xu W, Liu J, Song D, Li C, Zhu A, Long F. Rapid, label-free, and sensitive point-of-care testing of anti-SARS-CoV-2 IgM/IgG using all-fiber Fresnel reflection microfluidic biosensor. Mikrochimica acta. 2021;188(8):261.
16	Xu R, Rahmandad H, Gupta M, DiGennaro C, Ghaffarzadegan N, Amini H, et al. Weather, air pollution, and SARS-CoV-2 transmission: a global analysis. The Lancet Planetary Health. 2021;5(10):e671-e80.
17	Workman AD, Jafari A, Xiao R, Bleier BS. Airborne aerosol olfactory deposition contributes to anosmia in COVID-19. PLoS ONE. 2021;16(2 February) (no pagination).
18	Workman AD, Jafari A, Welling DB, Varvares MA, Gray ST, Holbrook EH, et al. Airborne Aerosol Generation During Endonasal Procedures in the Era of COVID-19: Risks and Recommendations. Otolaryngology - Head and Neck Surgery (United States). 2020;163(3):465-70.
19	Wathore R, Gupta A, Bherwani H, Labhasetwar N. Understanding air and water borne transmission and survival of coronavirus: Insights and way forward for SARS-CoV-2. Science of the Total Environment. 2020;749 (no pagination).
20	Watad A, De Marco G, Mahajna H, Druyan A, Eltity M, Hijazi N, et al. Immune-mediated disease flares or new-onset disease in 27 subjects following mrna/dna sars-cov-2 vaccination. Vaccines. 2021;9(5) (no pagination).
21	Wang Y, Xu G, Huang YW. Modeling the load of SARS-CoV-2 virus in human expelled particles during coughing and speaking. PLoS ONE. 2020;15(10 October) (no pagination).
22	Wang L, Wang K, Zhong H, Zhao N, Xu W, Yang Y, et al. The Effect of Coronavirus 2019 Disease Control Measures on the Incidence of Respiratory Infectious Disease and Air Pollutant Concentrations in the Yangtze River Delta Region, China. International Journal of Environmental Research and Public Health. 2022;19(3) (no pagination).
23	Wang J, Lv M, Xia H, Du J, Zhao Y, Li H, et al. Minimalist Design for a Hand-Held SARS-Cov-2 Sensor: Peptide-Induced Covalent Assembly of Hydrogel Enabling Facile Fiber-Optic Detection of a Virus Marker Protein. ACS sensors. 2021;6(6):2465-71.
24	Wang C, Wu Z, Liu B, Zhang P, Lu J, Li J, et al. Track-etched membrane microplate and smartphone immunosensing for SARS-CoV-2 neutralizing antibody. Biosensors and Bioelectronics. 2021;192 (no pagination).
25	Vikhe DM, Dhope SV, Mhaske PN, Shah SV, Palekar UG. "Pravara tent"- An innovative protective device to control aerosol in dental clinics during the COVID-19 pandemic. Journal of Clinical and Diagnostic Research. 2020;14(11):ZH01-ZH3.

26	Veronica Lopez M, Vinzon SE, Cafferata EGA, Nunez FJ, Soto A, Sanchez-Lamas M, et al. A single dose of a hybrid hadv5-based anti-covid-19 vaccine induces a long-lasting immune response and broad coverage against voc. Vaccines. 2021;9(10) (no pagination).
27	Uncini A, Foresti C, Frigeni B, Storti B, Servalli MC, Gazzina S, et al. Electrophysiological features of acute inflammatory demyelinating polyneuropathy associated with SARS-CoV-2 infection. Neurophysiologie Clinique. 2021;51(2):183-91.
28	Traina G, Barbalace A, Betti F, Bolzacchini E, Bonini M, Contini D, et al. What impact of air pollution in pediatric respiratory allergic diseases. Pediatric Allergy and Immunology. 2020;31(S26):26-8.
29	Toczylowski K, Wietlicka-Piszcz M, Grabowska M, Sulik A. Cumulative effects of particulate matter pollution and meteorological variables on the risk of influenza-like illness. Viruses. 2021;13(4) (no pagination).
30	Tadano YS, Potgieter-Vermaak S, Kachba YR, Chiroli DMG, Casacio L, Santos-Silva JC, et al. Dynamic model to predict the association between air quality, COVID-19 cases, and level of lockdown. Environmental Pollution. 2021;Part B. 268 (no pagination).
31	Sumbana J, Sacarlal J, Rubino S. Air pollution and other risk factors might buffer COVID-19 severity in Mozambique. Journal of Infection in Developing Countries. 2020;14(9):994-1000.
32	Subat YW, Hainy ME, Torgerud KD, Sajgalik P, Guntupalli SK, Johnson BD, et al. Aerosol generation and mitigation during methacholine bronchoprovocation testing: Infection control implications in the era of covid-19. Respiratory Care. 2021;66(12):1858-65.
33	Subat YW, Guntupalli SK, Sajgalik P, Hainy ME, Torgerud KD, Helgeson SA, et al. Aerosol generation during peak flow testing: Clinical implications for COVID-19. Respiratory Care. 2021;66(8):1291-8.
34	Steward JE, Kitley WR, Schmidt CM, Sundaram CP. Urologic Surgery and COVID-19: How the Pandemic Is Changing the Way We Operate. Journal of Endourology. 2020;34(5):541-9.
35	Stern RA, Koutrakis P, Martins MAG, Lemos B, Dowd SE, Sunderland EM, et al. Characterization of hospital airborne SARS-CoV-2. Respiratory Research. 2021;22(1) (no pagination).
36	Shukla S, Khan R, Saxena A, Sekar S, Ali EF, Shaheen SM. Appraisal of COVID-19 lockdown and unlocking effects on the air quality of North India. Environmental Research. 2022;Part B. 204 (no pagination).
37	Shirazi J, Donzanti MJ, Nelson KM, Zurakowski R, Fromen CA, Gleghorn JP. Significant Unresolved Questions and Opportunities for Bioengineering in Understanding and Treating COVID-19 Disease Progression. Cellular and Molecular Bioengineering. 2020;13(4):259-84.
38	Sharma D, Campiti VJ, Ye MJ, Saltagi M, Carroll AE, Ting JY, et al. Aerosol generation during cadaveric simulation of otologic surgery and live cochlear implantation. Laryngoscope Investigative Otolaryngology. 2021;6(1):129-36.
39	Saw LH, Leo BF, Nor NSM, Yip CW, Ibrahim N, Hamid HHA, et al. Modeling aerosol transmission of SARS-CoV-2 from human-exhaled particles in a hospital ward. Environmental science and pollution research international. 2021;28(38):53478-92.
40	Savastano A, Crincoli E, Savastano MC, Younis S, Gambini G, De Vico U, et al. Peripapillary retinal vascular involvement in early post-covid-19 patients. Journal of Clinical Medicine. 2020;9(9):1-16.
41	Sarapultseva M, Hu D, Sarapultsev A. SARS-CoV-2 Seropositivity among Dental Staff and the Role of Aspirating Systems. JDR clinical and translational research. 2021;6(2):132-8.
42	Sami S, Horter L, Valencia D, Thomas I, Pomeroy M, Walker B, et al. Investigation of SARS-CoV-2 Transmission Associated With a Large Indoor Convention - New York City, November-December 2021. Mmwr. 2022;Morbidity and mortality weekly report. 71(7):243-8.
43	Samet JM, Burke TA, Lakdawala SS, Lowe JJ, Marr LC, Prather KA, et al. SARS-CoV-2 indoor air transmission is a threat that can be addressed with science. Proceedings of the National Academy of Sciences of the United States of America. 2021;118(45) (no pagination).
44	Safdar N, Crnich CJ, Maki DG. The pathogenesis of ventilator-associated pneumonia: Its relevance to developing effective strategies for prevention. Respiratory Care. 2005;50(6):725-39.
45	Ruran HB, Adamkiewicz G, Cunningham A, Petty CR, Greco KF, Gunnlaugsson S, et al. Air quality, Environment and Respiratory Outcomes in Bronchopulmonary Dysplasia, the AERO-BPD cohort study: Design and adaptation during the SARS-CoV-2 pandemic. BMJ Open Respiratory Research. 2021;8(1) (no pagination).
46	Rugani B, Conticini E, Frediani B, Caro D. Decrease in life expectancy due to COVID-19 disease not offset by reduced environmental impacts associated with lockdowns in Italy. Environmental Pollution. 2022;Part A. 292 (no pagination).
47	Rubas NC, Maunakea A. Medical School Hotline: Immunoepigenetic-Microbiome Axis: Implications for Health Disparities Research in Native Hawaiians and Pacific Islanders. Hawai'i journal of health & social welfare. 2021;80(8):195-8.
48	Rodriguez M, Palop ML, Sesena S, Rodriguez A. Are the Portable Air Cleaners (PAC) really effective to terminate airborne SARS-CoV-2? Science of the Total Environment. 2021;785 (no pagination).
49	Robotto A, Civra A, Quaglino P, Polato D, Brizio E, Lembo D. SARS-CoV-2 airborne transmission: A validated sampling and analytical method. Environmental Research. 2021;200 (no pagination).
50	Rizzo E, Maggiotto G. Correlation between atmospheric particulate matter and antibiotic resistance: A hypothesis. Medical Hypotheses. 2020;141 (no pagination).
51	Rivera-Rios JC, Joo T, Takeuchi M, Orlando TM, Bevington T, Mathis JW, et al. In-flight particulate matter concentrations in commercial flights are likely lower than other indoor environments. Indoor air. 2021;31(5):1484-94.
52	Reyes J, Stiehl B, Delgado J, Kinzel M, Ahmed K. Human Research Study of Particulate Propagation Distance from Human Respiratory Function. The Journal of infectious diseases. 2022;12.
53	Rexhepi I, Mangifesta R, Santilli M, Guri S, Di Carlo P, D'Addazio G, et al. Effects of natural ventilation and saliva standard ejectors during the covid-19 pandemic: A quantitative analysis of aerosol produced during

	dental procedures. International Journal of Environmental Research and Public Health. 2021;18(14) (no pagination).
54	Resmi CT, Nishanth T, Satheesh Kumar MK, Manoj MG, Balachandramohan M, Valsaraj KT. Air quality improvement during triple-lockdown in the coastal city of Kannur, Kerala to combat Covid-19 transmission. PeerJ. 2020;8 (no pagination).
55	Reis NM, Needs SH, Jegouic SM, Gill KK, Sirivisoot S, Howard S, et al. Gravity-Driven Microfluidic Siphons: Fluidic Characterization and Application to Quantitative Immunoassays. ACS sensors. 2021;6(12):4338-48.
56	Rathore DS, Nagda C, Shaktawat BS, Kain T, Chouhan CS, Purohit R, et al. COVID-19 lockdown: a boon in boosting the air quality of major Indian Metropolitan Cities. Aerobiologia. 2021;37(1):79-103.
57	Rathnasinghe R, Karlicek RF, Schotsaert M, Koffas M, Arduini BL, Jangra S, et al. Scalable, effective, and rapid decontamination of SARS-CoV-2 contaminated N95 respirators using germicidal ultraviolet C (UVC) irradiation device. Scientific reports. 2021;11(1):19970.
58	Rajajee V, Williamson CA. Use of a Novel Negative-Pressure Tent During Bedside Tracheostomy in COVID-19 Patients. Neurocritical Care. 2020;33(2):597-603.
59	Raciti L, Calabro RS. Can volcanic trace elements facilitate Covid-19 diffusion? A hypothesis stemming from the Mount Etna area, Sicily. Medical Hypotheses. 2020;144 (no pagination).
60	Racine-Brzostek SE, Yang HS, Jack GA, Chen Z, Chadburn A, Ketas TJ, et al. Postconvalescent sars-cov-2 igg and neutralizing antibodies are elevated in individuals with poor metabolic health. Journal of Clinical Endocrinology and Metabolism. 2021;106(5):E2025-E34.
61	Raboud J, Shigayeva A, McGeer A, Bontovics E, Chapman M, Gravel D, et al. Risk factors for SARS transmission from patients requiring intubation: A multicentre investigation in Toronto, Canada. PLoS ONE. 2010;5(5) (no pagination).
62	Przekwas A, Chen Z. Washing hands and the face may reduce COVID-19 infection. Medical Hypotheses. 2020;144 (no pagination).
63	Pons MN, Louis P, Vignati D. Effect of lockdown on wastewater characteristics: A comparison of two large urban areas. Water Science and Technology. 2020;82(12):2813-22.
64	Pivato A, Amoruso I, Formenton G, Di Maria F, Bonato T, Vanin S, et al. Evaluating the presence of SARS-CoV-2 RNA in the particulate matters during the peak of COVID-19 in Padua, northern Italy. Science of the Total Environment. 2021;784 (no pagination).
65	Phu HT, Park Y, Andrews AJ, Marabella I, Abraham A, Mimmack R, et al. Design and evaluation of a portable negative pressure hood with HEPA filtration to protect health care workers treating patients with transmissible respiratory infections. American Journal of Infection Control. 2020;48(10):1237-43.
66	Peng S, Cao F, Xia Y, Gao XD, Dai L, Yan J, et al. Particulate Alum via Pickering Emulsion for an Enhanced COVID-19 Vaccine Adjuvant. Advanced materials (Deerfield Beach, Fla). 2020;32(40):e2004210.
67	Peddinti BST, Morales-Gagnon N, Pourdeyhimi B, Scholle F, Spontak RJ, Ghiladi RA. Photodynamic Coatings on Polymer Microfibers for Pathogen Inactivation: Effects of Application Method and Composition. ACS applied materials & interfaces. 2021;13(1):155-63.
68	Pandey AS, Ringer AJ, Rai AT, Kan P, Jabbour P, Siddiqui AH, et al. Minimizing SARS-CoV-2 exposure when performing surgical interventions during the COVID-19 pandemic. Journal of NeuroInterventional Surgery. 2020;12(7):643-7.
69	Palmeri V, Le Turdu-Chicot C, Garin B, Auguste A, Roger PM. Seroprevalence of SARS-CoV-2 IgG antibodies among workers in the University Hospital of Guadeloupe. Clinica Terapeutica. 2021;172(5):461-6.
70	Ozbas M, Demirayak B, Vural A, Karabela Y, Yigit FU. Investigation of Retinal Alterations in Patients Recovered from COVID-19: A Comparative Study. Ocular Immunology and Inflammation. 2022.
71	Owen L, Shivkumar M, Laird K. The Stability of Model Human Coronaviruses on Textiles in the Environment and during Health Care Laundering. mSphere. 2021;6(2):1-15.
72	Oren B, Aksoy Aydemir G, Aydemir E, Atesoglu HI, Goker YS, Kiziltoprak H, et al. Quantitative assessment of retinal changes in COVID-19 patients. Clinical & experimental optometry. 2021;104(6):717-22.
73	Oaklander AL. Clinical significance of angiotensin-converting enzyme 2 receptors for severe acute respiratory syndrome coronavirus 2 (COVID-19) on peripheral small-fiber sensory neurons is unknown today. Pain. 2020;161(11):2431-3.
74	Nunez-Delgado A, Bontempi E, Coccia M, Kumar M, Farkas K, Domingo JL. SARS-CoV-2 and other pathogenic microorganisms in the environment. Environmental Research. 2021;201 (no pagination).
75	Nozza E, Valentini S, Melzi G, Vecchi R, Corsini E. Advances on the immunotoxicity of outdoor particulate matter: A focus on physical and chemical properties and respiratory defence mechanisms. Science of the Total Environment. 2021;780 (no pagination).
76	Nor NSM, Yip CW, Ibrahim N, Jaafar MH, Rashid ZZ, Mustafa N, et al. Particulate matter (PM<inf>2.5</inf>) as a potential SARS-CoV-2 carrier. Scientific reports. 2021;11(1):2508.
77	Nguyen TPM, Bui TH, Nguyen MK, Nguyen TH, Vu VT, Pham HL. Impact of Covid-19 partial lockdown on PM<inf>2.5</inf>, SO<inf>2</inf>, NO<inf>2</inf>, O<inf>3</inf>, and trace elements in PM<inf>2.5</inf> in Hanoi, Vietnam. Environmental science and pollution research international. 2021;08.
78	Naidoo P, Ghazi T, Chuturgoon AA, Naidoo RN, Ramsuran V, Mpaka-Mbatha MN, et al. SARS-CoV-2 and helminth co-infections, and environmental pollution exposure: An epidemiological and immunological perspective. Environment International. 2021;156 (no pagination).
79	Mulder C, Conti E, Saccone S, Federico C. Beyond virology: environmental constraints of the first wave of COVID-19 cases in Italy. Environmental science and pollution research international. 2021;28(24):31996-2004.
80	Mimura T, Noma H, Matsumoto K, Kawashima M, Kitsu K, Itoh E, et al. Concentration of droplets from patients during normal breathing and speech and their importance in protection from coronavirus sars-cov-2 (Covid-19) infection. Open Ophthalmology Journal. 2021;15(1):109-7.

81	Milone I, Vento R, Ippolito L, Paroni S, Vento MG. Therapeutic support protocol for patient with dysosmia with or without dysgeusia related to the SARS-CoV2 virus infection. Acta Biomedica. 2020;91(4):1-2.
82	Mescoli A, Maffei G, Pillo G, Bortone G, Marchesi S, Morandi E, et al. The Secretive Liaison of Particulate Matter and SARS-CoV-2. A Hypothesis and Theory Investigation. Frontiers in Genetics. 2020;11 (no pagination).
83	Mendoza DL, Benney TM, Bares R, Crosman ET. Intra-city variability of fine particulate matter during COVID-19 lockdown: A case study from Park City, Utah. Environmental Research. 2021;201 (no pagination).
84	Marcos-Garcia P, Carmona-Moreno C, Lopez-Puga J, Ruiz-Ruano Garcia AM. COVID-19 pandemic in Africa: Is it time for water, sanitation and hygiene to climb up the ladder of global priorities? Science of the Total Environment. 2021;791 (no pagination).
85	Manivannan J, Sundaresan L. Systems level insights into the impact of airborne exposure on SARS-CoV-2 pathogenesis and COVID-19 outcome - A multi-omics big data study. Gene Reports. 2021;25 (no pagination).
86	Macias-Verde D, Lara PC, Burgos-Burgos J. Same pollution sources for climate change might be hyperactivating the NLRP3 inflammasome and exacerbating neuroinflammation and SARS mortality. Medical Hypotheses. 2021;146 (no pagination).
87	Lopez JH, Romo AS, Molina DC, Hernandez GA, Cureno ABG, Acosta MA, et al. Detection of Sars-Cov-2 in the air of two hospitals in Hermosillo, Sonora, Mexico, utilizing a low-cost environmental monitoring system. International Journal of Infectious Diseases. 2021;102:478-82.
88	Liu D, Thompson JR, Carducci A, Bi X. Potential secondary transmission of SARS-CoV-2 via wastewater. Science of the Total Environment. 2020;749 (no pagination).
89	Liu BM, Yang QQ, Zhao LY, Xie W, Si XY. Epidemiological characteristics of COVID-19 patients in convalescence period. Epidemiology and Infection. 2020.
90	Linneberg A, Kampmann FB, Israelsen SB, Andersen LR, Jorgensen HL, Sandholt H, et al. The association of low vitamin k status with mortality in a cohort of 138 hospitalized patients with covid-19. Nutrients. 2021;13(6) (no pagination).
91	Lindsley WG, Derk RC, Coyle JP, Martin SB, Mead KR, Blachere FM, et al. Efficacy of Portable Air Cleaners and Masking for Reducing Indoor Exposure to Simulated Exhaled SARS-CoV-2 Aerosols - United States, 2021. Mmwr. 2021;Morbidity and mortality weekly report. 70(27):972-6.
92	Lin Z, Shu H, Jiang D, He Y, Xia H, Liu Y, et al. Ward renovation and PPE use procedures to protect medical staff from COVID-19 infection. Journal of Infection in Developing Countries. 2020;14(6):554-8.
93	Li L, Gu J, Gong E, Li X, Shao H, Jiang H, et al. Biosafety level 3 laboratory for autopsies of patients with severe acute respiratory syndrome: Principles, practices, and prospects. Clinical Infectious Diseases. 2005;41(6):815-21.
94	Lelieveld J, Helleis F, Borrmann S, Cheng Y, Drewnick F, Haug G, et al. Model calculations of aerosol transmission and infection risk of covid-19 in indoor environments. International Journal of Environmental Research and Public Health. 2020;17(21):1-18.
95	Leal-Martinez F, Abarca-Bernal L, Garcia-Perez A, Gonzalez-Tolosa D, Cruz-Cazares G, Montell-Garcia M, et al. Effect of a Nutritional Support System to Increase Survival and Reduce Mortality in Patients with COVID-19 in Stage III and Comorbidities: A Blinded Randomized Controlled Clinical Trial. International Journal of Environmental Research and Public Health. 2022;19(3) (no pagination).
96	Lani-Louzada R, do Val Ferreira Ramos C, Cordeiro RM, Sadun AA. Retinal changes in COVID-19 hospitalized cases. PLoS ONE. 2020;15(12 December) (no pagination).
97	Landry SA, Barr JJ, MacDonald MI, Subedi D, Mansfield D, Hamilton GS, et al. Viable virus aerosol propagation by positive airway pressure circuit leak and mitigation with a ventilated patient hood. European Respiratory Journal. 2021;57(6) (no pagination).
98	Kostoff RN, Briggs MB, Kanduc D, Shores DR, Kovatsi L, Drakoulis N, et al. Contributing factors common to COVID-19 and gastrointestinal cancer. Oncology Reports. 2022;47(1) (no pagination).
99	Khan YA. The COVID-19 pandemic and its impact on environment: the case of the major cities in Pakistan. Environmental science and pollution research international. 2021;28(39):54728-43.
100	Jones HAS, Salib RJ, Harries PG. Reducing Aerosolized Particles and Droplet Spread in Endoscopic Sinus Surgery during COVID-19. Laryngoscope. 2021;131(5):956-60.
101	Jones HA, Salib RJ, Harries PG. Reducing Aerosolised Particles and Droplet Spread in Endoscopic Sinus Surgery during COVID-19. The Laryngoscope. 2020;15.
102	Jones GW, Monopoli MP, Campagnolo L, Pietroiusti A, Tran L, Fadeel B. No small matter: A perspective on nanotechnology-enabled solutions to fight COVID-19. Nanomedicine. 2020;15(24):2411-27.
103	Jephcote C, Hansell AL, Adams K, Gulliver J. Changes in air quality during COVID-19 'lockdown' in the United Kingdom. Environmental Pollution. 2021;272 (no pagination).
104	Jeican II, Gheban D, Barbu-Tudoran L, Inisca P, Albu C, Ilies M, et al. Respiratory nasal mucosa in chronic rhinosinusitis with nasal polyps versus covid-19: Histopathology, electron microscopy analysis and assessing of tissue interleukin-33. Journal of Clinical Medicine. 2021;10(18) (no pagination).
105	Jagtap PK, Kolla V. A comprehensive report on critical aspects of the virus that caged us. European Journal of Molecular and Clinical Medicine. 2020;7(3):2587-601.
106	Irandoust F, Dini S. A new perspective of aroma face mask on COVID-19 pandemic. Journal of Medical Engineering and Technology. 2022.
107	Ionita C, Marcelli D, Nita C, Anton C, Berca S, Vacar S, et al. Comparison of antibody response to two different mRNA Covid-19 vaccines in patients on hemodialysis. Journal of Nephrology. 2022;35(1):143-51.
108	Ilardi A, Chieffi S, Ilardi CR. Predictive Role of Population Density and Use of Public Transport for Major Outcomes of SARS-CoV-2 Infection in the Italian Population: An Ecological Study. Journal of Research in Health Sciences. 2021;21(2) (no pagination).

109	Ibarra-Espinosa S, Dias de Freitas E, Ropkins K, Dominici F, Rehbein A. Negative-Binomial and quasi-poisson regressions between COVID-19, mobility and environment in Sao Paulo, Brazil. Environmental Research. 2022;Part D. 204 (no pagination).
110	Husain-Syed F, Birk HW, Wilhelm J, Ronco C, Ranieri VM, Karle B, et al. Extracorporeal Carbon Dioxide Removal Using a Renal Replacement Therapy Platform to Enhance Lung-Protective Ventilation in Hypercapnic Patients With Coronavirus Disease 2019-Associated Acute Respiratory Distress Syndrome. Frontiers in Medicine. 2020;7 (no pagination).
111	Humphreys H, Fitzpatrick F. Airborne transmission of covid-19: Implications for irish hospitals. Irish Medical Journal. 2020;113(7):1-3.
112	Huang JC, Chang YF, Chen KH, Su LC, Lee CW, Chen CC, et al. Detection of severe acute respiratory syndrome (SARS) coronavirus nucleocapsid protein in human serum using a localized surface plasmon coupled fluorescence fiber-optic biosensor. Biosensors and Bioelectronics. 2009;25(2):320-5.
113	Howard BE. High-Risk Aerosol-Generating Procedures in COVID-19: Respiratory Protective Equipment Considerations. Otolaryngology - Head and Neck Surgery (United States). 2020;163(1):98-103.
114	Hill WC, Hull MS, MacCuspie RI. Testing of Commercial Masks and Respirators and Cotton Mask Insert Materials using SARS-CoV-2 Virion-Sized Particulates: Comparison of Ideal Aerosol Filtration Efficiency versus Fitted Filtration Efficiency. Nano letters. 2020;20(10):7642-7.
115	Hao W, Wu J, Zhao X, Liang D, Yu X, Cao H, et al. Quantitative Evaluation of Aerosol Generation from Non-contact Tonometry and its Correlation with Tear Film Characteristics. Advances in Therapy. 2021;38(6):3066-76.
116	Hansell AL, Villeneuve PJ. Invited perspective: Ambient air pollution and sars-cov-2: Research challenges and public health implications. Environmental Health Perspectives. 2021;129(11) (no pagination).
117	Hamming I, Timens W, Bulthuis MLC, Lely AT, Navis GJ, van Goor H. Tissue distribution of ACE2 protein, the functional receptor for SARS coronavirus. A first step in understanding SARS pathogenesis. Journal of Pathology. 2004;203(2):631-7.
118	Haghayegh F, Salahandish R, Zare A, Khalghollah M, Sanati-Nezhad A. Immuno-biosensor on a chip: a self-powered microfluidic-based electrochemical biosensing platform for point-of-care quantification of proteins. Lab on a Chip. 2022;22(1):108-20.
119	Hadi MU, Khurshid M. SARS-CoV-2 Detection Using Optical Fiber Based Sensor Method. Sensors. 2022;22(3).
120	Gutsell J, Yang Y, Jeffrey M, Conway-Morris A, Mahroof R, Martin J. Reducing breathing system transmission of Covid-19: The Addenbrooke's experience. Intensive Care Medicine Experimental Conference: 33rd European Society of Intensive Care Medicine Annual Congress, ESICM. 2020;8(SUPPL 2).
121	Gupta S, Kalra J, Goyal H, Kumar V. Analysis on worldwide coronavirus (COVID-19) cases. Eastern Journal of Medicine. 2020;25(4):591-9.
122	Gupta S, Dubey H, Rai A, Singh P, Jhunjhunwala N, Singh S. Desirable and Undesirable Effects of Air Purifier in Clinical Settings during Covid-19 Pandemic. European Journal of Molecular and Clinical Medicine. 2020;7(6):233-6.
123	Grinshpun SA, Yermakov M, Kano M. Evaluation of AccuFIT 9000: A Novel Apparatus for Quantitative Fit Testing of Particulate Respirators. Annals of work exposures and health. 2021;65(4):458-62.
124	Gregorio PHP, Mariani AW, Brito JMLT, Santos BJM, Pego-Fernandes PM. Indoor Air Quality and Environmental Sampling as Support Tools to Detect SARS-CoV-2 in the Healthcare Setting. Journal of occupational and environmental medicine. 2021;63(11):956-62.
125	Gill AS, Oakley G, Error M, Kelly K, Orlandi R, Alt JA. Optimizing clinical productivity in the otolaryngology clinic during the COVID-19 pandemic. International Forum of Allergy and Rhinology. 2021;11(7):1121-3.
126	Gianquintieri L, Brovelli MA, Pagliosa A, Bonora R, Sechi GM, Caiani EG. Geospatial correlation analysis between air pollution indicators and estimated speed of covid-19 diffusion in the lombardy region (Italy). International Journal of Environmental Research and Public Health. 2021;18(22) (no pagination).
127	Ghaffari HR, Farshidi H, Alipour V, Dindarloo K, Azad MH, Jamalidoust M, et al. Detection of SARS-CoV-2 in the indoor air of intensive care unit (ICU) for severe COVID-19 patients and its surroundings: considering the role of environmental conditions. Environmental science and pollution research international. 2021;05.
128	George B, Megally M, Mrejen-Shakin K. Spontaneous Pneumothorax and Spontaneous Pneumomediastinum in Non-Intubated Patients in the Setting of Severe Acute Respiratory Syndrome Coronavirus 2. Chest. 2020;158(4 Supplement):A1653-A4.
129	Fronza R, Lusic M, Schmidt M, Lucic B. Spatial-temporal variations in atmospheric factors contribute to SARS-CoV-2 outbreak. Viruses. 2020;12(6) (no pagination).
130	Freire-Paspuel B, Vega-Marino P, Velez A, Castillo P, Gomez-Santos EE, Cruz M, et al. Cotton-Tipped Plastic Swabs for SARS-CoV-2 RT-qPCR Diagnosis to Prevent Supply Shortages. Frontiers in Cellular and Infection Microbiology. 2020;10 (no pagination).
131	Fongaro G, Stoco PH, Souza DSM, Grisard EC, Magri ME, Rogovski P, et al. The presence of SARS-CoV-2 RNA in human sewage in Santa Catarina, Brazil, November 2019. Science of the Total Environment. 2021;778 (no pagination).
132	Folcarelli L, Del Giudice GM, Corea F, Angelillo IF. Intention to Receive the COVID-19 Vaccine Booster Dose in a University Community in Italy. Vaccines. 2022;10(2) (no pagination).
133	Fernandez-Arribas J, Moreno T, Bartroli R, Eljarrat E. COVID-19 face masks: A new source of human and environmental exposure to organophosphate esters. Environment International. 2021;154 (no pagination).
134	Fenrich M, Mrdenovic S, Balog M, Tomic S, Zjalic M, Roncevic A, et al. SARS-CoV-2 Dissemination Through Peripheral Nerves Explains Multiple Organ Injury. Frontiers in Cellular Neuroscience. 2020;14 (no pagination).
135	Esser C, Hochrath K, Schikowski T, Haarmann-Stemmann T. COVID-19 research: toxicological input urgently needed! Archives of Toxicology. 2020;94(7):2547-8.

136	Edwards L, Rutter G, Iverson L, Wilson L, Chadha TS, Wilkinson P, et al. Personal exposure monitoring of PM<inf>2.5</inf> among US diplomats in Kathmandu during the COVID-19 lockdown, March to June 2020. Science of the Total Environment. 2021;772 (no pagination).
137	Duill FF, Schulz F, Jain A, Krieger L, van Wachem B, Beyrau F. The impact of large mobile air purifiers on aerosol concentration in classrooms and the reduction of airborne transmission of sars-cov-2. International Journal of Environmental Research and Public Health. 2021;18(21) (no pagination).
138	Dubey A, Kotnala G, Mandal TK, Sonkar SC, Singh VK, Guru SA, et al. Evidence of the presence of SARS-CoV-2 virus in atmospheric air and surfaces of a dedicated COVID hospital. Journal of Medical Virology. 2021;93(9):5339-49.
139	Dicerbo A. Air pollution and SARS-CoV-2 in the Po Valley: Possible environmental persistence? Minerva Medica. 2020;111(4):306-7.
140	Day AS, Ulep TH, Safavinia B, Hertenstein T, Budiman E, Dieckhaus L, et al. Emulsion-based isothermal nucleic acid amplification for rapid SARS-CoV-2 detection via angle-dependent light scatter analysis. Biosensors and Bioelectronics. 2021;179 (no pagination).
141	Curtius J, Granzin M, Schrod J. Testing mobile air purifiers in a school classroom: Reducing the airborne transmission risk for SARS-CoV-2. Aerosol Science and Technology. 2021;55(5):586-99.
142	Cruz R, Lima-Silva AE, Bertuzzi R, Hoinaski L. Exercising under particulate matter exposure: Providing theoretical support for lung deposition and its relationship with COVID-19. Environmental Research. 2021;202 (no pagination).
143	Crotty T, Sehgal R, Grundy J, Cahill R, Brennan D, Conneely J, et al. Cytoreductive surgery (Crs) and hyperthermic intraperitoneal chemotherapy (hipec) for peritoneal malignancy during the covid-19 pandemic. Irish Medical Journal. 2021;114(5) (no pagination).
144	Crosby DL, Sharma A. Evidence-Based Guidelines for Management of Head and Neck Mucosal Malignancies during the COVID-19 Pandemic. Otolaryngology - Head and Neck Surgery (United States). 2020;163(1):16-24.
145	Cortes MF, Espinoza EPS, Noguera SLV, Silva AA, de Medeiros MESA, Villas Boas LS, et al. Decontamination and re-use of surgical masks and respirators during the COVID-19 pandemic. International Journal of Infectious Diseases. 2021;104:320-8.
146	Conticini E, Frediani B, Caro D. Can atmospheric pollution be considered a co-factor in extremely high level of SARS-CoV-2 lethality in Northern Italy? Environmental Pollution. 2020;261 (no pagination).
147	Conte L, Toraldo DM. Targeting the gut-lung microbiota axis by means of a high-fibre diet and probiotics may have anti-inflammatory effects in COVID-19 infection. Therapeutic Advances in Respiratory Disease. 2020;14(no pagination).
148	Comisi JC, Ravenel TD, Kelly A, Teich ST, Renne W. Aerosol and spatter mitigation in dentistry: Analysis of the effectiveness of 13 setups. Journal of Esthetic and Restorative Dentistry. 2021;33(3):466-79.
149	Cohen SL, Liu G, Abrao M, Smart N, Heniford T. Perspectives on Surgery in the Time of COVID-19: Safety First. Journal of Minimally Invasive Gynecology. 2020;27(4):792-3.
150	Cognetti JS, Steiner DJ, Abedin M, Bryan MR, Shanahan C, Tokranova N, et al. Disposable photonics for cost-effective clinical bioassays: application to COVID-19 antibody testing. Lab on a Chip. 2021;21(15):2913-21.
151	Coelho WEGDS, Perrechil F, Pedreira MLG, Lopes JL, Santos MVLD, Gabrieloni MC, et al. Safety and structural integrity of N95/PFF2 respirators decontamination. American Journal of Infection Control. 2021;49(10):1221-6.
152	Christophi CA, Sotos-Prieto M, Lan FY, Delgado-Velandia M, Efthymiou V, Gaviola GC, et al. Ambient temperature and subsequent COVID-19 mortality in the OECD countries and individual United States. Scientific reports. 2021;11(1):8710.
153	Chirizzi D, Conte M, Feltracco M, Dinoi A, Gregoris E, Barbaro E, et al. SARS-CoV-2 concentrations and virus-laden aerosol size distributions in outdoor air in north and south of Italy. Environment International. 2021;146 (no pagination).
154	Ching J, Kajino M. Rethinking air quality and climate change after covid-19. International Journal of Environmental Research and Public Health. 2020;17(14):1-11.
155	Cheung YH, Ma K, Van Leeuwen HC, Wasson MC, Wang X, Idrees KB, et al. Immobilized Regenerable Active Chlorine within a Zirconium-Based MOF Textile Composite to Eliminate Biological and Chemical Threats. Journal of the American Chemical Society. 2021;143(40):16777-85.
156	Cheng VCC, Fung KSC, Siu GKH, Wong SC, Cheng LSK, Wong MS, et al. Nosocomial Outbreak of Coronavirus Disease 2019 by Possible Airborne Transmission Leading to a Superspreading Event. Clinical infectious diseases : an official publication of the Infectious Diseases Society of America. 2021;73(6):e1356-e64.
157	Chari DA, Workman AD, Chen JX, Jung DH, Abdul-Aziz D, Kozin ED, et al. Aerosol Dispersion During Mastoidectomy and Custom Mitigation Strategies for Otologic Surgery in the COVID-19 Era. Otolaryngology - Head and Neck Surgery (United States). 2021;164(1):67-73.
158	Chaovavanich A, Wongsawat J, Dowell SF, Inthong Y, Sangsajja C, Sanguanwongse N, et al. Early containment of severe acute respiratory syndrome (SARS); experience from Bamrasnaradura Institute, Thailand. Journal of the Medical Association of Thailand. 2004;87(10):1182-7.
159	Chakraborty P, Pasupuleti M, Jai Shankar MR, Bharat GK, Krishnasamy S, Dasgupta SC, et al. First surveillance of SARS-CoV-2 and organic tracers in community wastewater during post lockdown in Chennai, South India: Methods, occurrence and concurrence. Science of the Total Environment. 2021;778 (no pagination).
160	Chadeau-Hyam M, Bodinier B, Elliott J, Whitaker MD, Tzoulaki I, Vermeulen R, et al. Risk factors for positive and negative COVID-19 tests: A cautious and in-depth analysis of UK biobank data. International Journal of Epidemiology. 2020;49(5):1454-67.
161	Cennamo N, Pasquardini L, Arcadio F, Lunelli L, Vanzetti L, Carafa V, et al. SARS-CoV-2 spike protein detection through a plasmonic D-shaped plastic optical fiber aptasensor. Talanta. 2021;233:122532.

162	Cennamo N, D'Agostino G, Perri C, Arcadio F, Chiaretti G, Parisio EM, et al. Proof of Concept for a Quick and Highly Sensitive On-Site Detection of SARS-CoV-2 by Plasmonic Optical Fibers and Molecularly Imprinted Polymers. Sensors. 2021;21(5).
163	Caseiro A, von Schneidemesser E. APExpose_DE, an air quality exposure dataset for Germany 2010-2019. Scientific data. 2021;8(1):287.
164	Carteni A, Di Francesco L, Martino M. The role of transport accessibility within the spread of the Coronavirus pandemic in Italy. Safety Science. 2021;133 (no pagination).
165	Carteni A, Di Francesco L, Martino M. How mobility habits influenced the spread of the COVID-19 pandemic: Results from the Italian case study. Science of the Total Environment. 2020;741 (no pagination).
166	Calderon-Garciduenas L, Gonzalez-Maciel A, Reynoso-Robles R, Rodriguez-Lopez JL, Silva-Pereyra HG, Labrada-Delgado GJ, et al. Environmental Fe, Ti, Al, Cu, Hg, Bi, and Si nanoparticles in the atrioventricular conduction axis and the associated ultrastructural damage in young urbanites: Cardiac arrhythmias caused by anthropogenic, industrial, e-waste, and indoor nanoparticles. Environmental Science and Technology. 2021;55(12):8203-14.
167	Burgos-Blasco B, Guemes-Villahoz N, Vidal-Villegas B, Martinez-de-la-Casa JM, Donate-Lopez J, Martin-Sanchez FJ, et al. Optic nerve and macular optical coherence tomography in recovered COVID-19 patients. European Journal of Ophthalmology. 2022;32(1):628-36.
168	Buonanno M, Welch D, Shuryak I, Brenner DJ. Far-UVC light (222nm) efficiently and safely inactivates airborne human coronaviruses. Scientific reports. 2020;10(1):10285.
169	Buja A, Manfredi M, De Luca G, Zampieri C, Zanovello S, Perkovic D, et al. Using failure mode, effect and criticality analysis to improve safety in the covid mass vaccination campaign. Vaccines. 2021;9(8) (no pagination).
170	Brune Z, Kuschner CE, Mootz J, Davidson KW, Pena RCF, Ghanem MH, et al. Effectiveness of sars-cov-2 decontamination and containment in a covid-19 icu. International Journal of Environmental Research and Public Health. 2021;18(5):1-9.
171	Brocke S, Taft-Benz S, Robinette C, Knight N, Heise M, Jaspers I. Effects of Particulate Matter on SARS-CoV-2 Induced Antiviral Responses in Human Nasal Epithelial Cells. American Journal of Respiratory and Critical Care Medicine Conference: American Thoracic Society International Conference, ATS. 2021;203(9).
172	Briguglio M, Bona A, Porta M, Dell'Osso B, Pregliasco FE, Banfi G. Disentangling the Hypothesis of Host Dysosmia and SARS-CoV-2: The Bait Symptom That Hides Neglected Neurophysiological Routes. Frontiers in Physiology. 2020;11 (no pagination).
173	Brant-Zawadzki GM, Ockerse P, Brunson JR, Smith JL, McRae BR, Fonnesbeck A, et al. An Aerosol Containment and Filtration Tent for Intubation During the COVID-19 Pandemic. Surgical Innovation. 2021;28(2):226-30.
174	Braga F, Scarpa GM, Brando VE, Manfe G, Zaggia L. COVID-19 lockdown measures reveal human impact on water transparency in the Venice Lagoon. Science of the Total Environment. 2020;736 (no pagination).
175	Borak J. Airborne Transmission of COVID-19. Occupational medicine (Oxford, England). 2020;70(5):297-9.
176	Bontempi E, Coccia M. International trade as critical parameter of COVID-19 spread that outclasses demographic, economic, environmental, and pollution factors. Environmental Research. 2021;201 (no pagination).
177	Bontempi E. A global assessment of COVID-19 diffusion based on a single indicator: Some considerations about air pollution and COVID-19 spread. Environmental Research. 2022;Part B. 204 (no pagination).
178	Bitirgen G, Korkmaz C, Zamani A, Ozkagnici A, Zengin N, Ponirakis G, et al. Corneal confocal microscopy identifies corneal nerve fibre loss and increased dendritic cells in patients with long COVID. British Journal of Ophthalmology. 2021.
179	Bian S, Shang M, Sawan M. Rapid biosensing SARS-CoV-2 antibodies in vaccinated healthy donors. Biosensors and Bioelectronics. 2022;204 (no pagination).
180	Bhattarai B, Sahulka SQ, Podder A, Hong S, Li H, Gilcrease E, et al. Prevalence of SARS-CoV-2 genes in water reclamation facilities: From influent to anaerobic digester. Science of the Total Environment. 2021;796 (no pagination).
181	Belosi F, Conte M, Gianelle V, Santachiara G, Contini D. On the concentration of SARS-CoV-2 in outdoor air and the interaction with pre-existing atmospheric particles. Environmental Research. 2021;193 (no pagination).
182	Bello-Lopez JM, Silva-Bermudez P, Prado G, Martinez A, Ibanez-Cervantes G, Cureno-Diaz MA, et al. Biocide effect against SARS-CoV-2 and ESKAPE pathogens of a noncytotoxic silver-copper nanofilm. Biomedical Materials (Bristol). 2022;17(1) (no pagination).
183	Barros A, Queiruga-Pineiro J, Lozano-Sanroma J, Alcalde I, Gallar J, Fernandez-Vega Cueto L, et al. Small fiber neuropathy in the cornea of Covid-19 patients associated with the generation of ocular surface disease. Ocular Surface. 2022;23:40-8.
184	Barbieri P, Zupin L, Licen S, Torboli V, Semeraro S, Cozzutto S, et al. Molecular detection of SARS-CoV-2 from indoor air samples in environmental monitoring needs adequate temporal coverage and infectivity assessment. Environmental Research. 2021;198 (no pagination).
185	Baldelli G, Aliano MP, Amagliani G, Magnani M, Brandi G, Pennino C, et al. Airborne Microorganism Inactivation by a UV-C LED and Ionizer-Based Continuous Sanitation Air (CSA) System in Train Environments. International Journal of Environmental Research and Public Health. 2022;19(3) (no pagination).
186	Babkina AS, Ostrova IV, Yadgarov MY, Kuzovlev AN, Grechko AV, Volkov AV, et al. The Role of Von Willebrand Factor in the Pathogenesis of Pulmonary Vascular Thrombosis in COVID-19. Viruses. 2022;14(2) (no pagination).
187	Azuma K, Kagi N, Kim H, Hayashi M. Impact of climate and ambient air pollution on the epidemic growth during COVID-19 outbreak in Japan. Environmental Research. 2020;190 (no pagination).

188	Aylward R, Bieber B, Guedes M, Pisoni R, Tannor EK, Dreyer G, et al. The Global Impact of the COVID-19 Pandemic on In-Center Hemodialysis Services: An ISN-Dialysis Outcomes Practice Patterns Study Survey. Kidney International Reports. 2022.
189	Augenbraun BL, Lasner ZD, Mitra D, Prabhu S, Raval S, Sawaoka H, et al. Assessment and mitigation of aerosol airborne SARS-CoV-2 transmission in laboratory and office environments. Journal of occupational and environmental hygiene. 2020;17(10):447-56.
190	Anser MK, Godil DI, Khan MA, Nassani AA, Zaman K, Abro MMQ. The impact of coal combustion, nitrous oxide emissions, and traffic emissions on COVID-19 cases: a Markov-switching approach. Environmental science and pollution research international. 2021;28(45):64882-91.
191	Anghel L, Popovici CG, Statescu C, Sascau R, Verdes M, Ciocan V, et al. Impact of hvac-systems on the dispersion of infectious aerosols in a cardiac intensive care unit. International Journal of Environmental Research and Public Health. 2020;17(18):1-17.
192	Allen JG, Ibrahim AM. Indoor Air Changes and Potential Implications for SARS-CoV-2 Transmission. JAMA - Journal of the American Medical Association. 2021;325(20):2112-3.
193	Ali K, Raja M. Coronavirus disease 2019 (COVID-19): challenges and management of aerosol-generating procedures in dentistry. Evidence-based dentistry. 2020;21(2):44-5.
194	Abrishami M, Daneshvar R, Emamverdian Z, Tohidinezhad F, Eslami S. Optic Nerve Head Parameters and Peripapillary Retinal Nerve Fiber Layer Thickness in Patients with Coronavirus Disease 2019. Ocular Immunology and Inflammation. 2021.
195	Abrams RMC, Simpson DM, Navis A, Jette N, Zhou L, Shin SC. Small fiber neuropathy associated with SARS-CoV-2 infection. Muscle and Nerve. 2021.
196	Abdalrhman AS, Wang C, Manalac A, Weersink M, Yassine AA, Betz V, et al. Modeling the efficiency of UV at 254nm for disinfecting the different layers within N95 respirators. Journal of biophotonics. 2021;14(10):e202100135.
197	Zhao L, Zhang FS, Wang K, Zhu J. Chemical properties of heavy metals in typical hospital waste incinerator ashes in China. Waste Manag. 2009;29(3):1114-21.
198	Zhang YH, Guo GH, Shen GL, Han W, Zhao XY, Lin W, et al. [Analysis on treatment of extremely severe burn patients with severe inhalation injury in August 2nd Kunshan factory aluminum dust explosion accident]. Zhonghua Shao Shang Za Zhi. 2018;34(7):455-8.
199	Zerefos CS, Solomos S, Kapsomenakis J, Poupkou A, Dimitriadou L, Polychroni ID, et al. Lessons learned and questions raised during and post-COVID-19 anthropopause period in relation to the environment and climate. Environ Dev Sustain. 2021;23(7):10623-45.
200	Yuan JT, Jiang SIB. Urgent safety considerations for dermatologic surgeons in the COVID-19 pandemic. Dermatol Online J. 2020;26(8).
201	Yokoe T, Kita M, Odaka T, Fujisawa J, Hisamatsu Y, Okada H. Detection of human coronavirus RNA in surgical smoke generated by surgical devices. J Hosp Infect. 2021;117:89-95.
202	Yao Y, Pan J, Liu Z, Meng X, Wang W, Kan H, et al. Ambient nitrogen dioxide pollution and spreadability of COVID-19 in Chinese cities. Ecotoxicol Environ Saf. 2021;208:111421.
203	Yang FW, Xin HM, Zhu JH, Feng XY, Jiang XC, Gong ZY, et al. [Treatment of patients with different degree of acute respiratory distress syndrome caused by inhalation of white smoke]. Zhonghua Shao Shang Za Zhi. 2017;33(12):760-5.
204	Xie F, Zhang X, Xie L. Prognostic value of serum zinc levels in patients with acute HC/zinc chloride smoke inhalation. Medicine (Baltimore). 2017;96(39):e8156.
205	Xiao LI, Sakagami H, Miwa N. A New Method for Testing Filtration Efficiency of Mask Materials Under Sneeze-like Pressure. In Vivo. 2020;34(3 Suppl):1637-44.
206	Wannaz ED, Larrea Valdivia AE, Reyes Larico JA, Salcedo Peña J, Valenzuela Huillca C. PM(10) correlates with COVID-19 infections 15 days later in Arequipa, Peru. Environ Sci Pollut Res Int. 2021;28(29):39648-54.
207	Wang C, Wolters PJ, Calfee CS, Liu S, Balmes JR, Zhao Z, et al. Long-term ozone exposure is positively associated with telomere length in critically ill patients. Environ Int. 2020;141:105780.
208	Viteri G, Díaz de Mera Y, Rodríguez A, Rodríguez D, Tajuelo M, Escalona A, et al. Impact of SARS-CoV-2 lockdown and de-escalation on air-quality parameters. Chemosphere. 2021;265:129027.
209	Villanueva F, Notario A, Cabañas B, Martín P, Salgado S, Gabriel MF. Assessment of CO(2) and aerosol (PM(2.5), PM(10), UFP) concentrations during the reopening of schools in the COVID-19 pandemic: The case of a metropolitan area in Central-Southern Spain. Environ Res. 2021;197:111092.
210	Vasquez-Apestegui V, Parras-Garrido E, Tapia V, Paz-Aparicio VM, Rojas JP, Sánchez-Ccoyllo OR, et al. Association Between Air Pollution in Lima and the High Incidence of COVID-19: Findings from a Post Hoc Analysis. Res Sq. 2020.
211	Urrutia-Pereira M, Mello-da-Silva CA, Solé D. Household pollution and COVID-19: irrelevant association? Allergol Immunopathol (Madr). 2021;49(1):146-9.
212	Tobías A, Carnerero C, Reche C, Massagué J, Via M, Minguillón MC, et al. Changes in air quality during the lockdown in Barcelona (Spain) one month into the SARS-CoV-2 epidemic. Sci Total Environ. 2020;726:138540.
213	Teixidó O, Tobías A, Massagué J, Mohamed R, Ekaabi R, Hamed HI, et al. The influence of COVID-19 preventive measures on the air quality in Abu Dhabi (United Arab Emirates). Air Qual Atmos Health. 2021;14(7):1071-9.
214	Tavella RA, da Silva Júnior FMR. Watch out for trends: did ozone increased or decreased during the COVID-19 pandemic? Environ Sci Pollut Res Int. 2021;28(47):67880-5.
215	Su X, Sutarlie L, Loh XJ. Sensors and Analytical Technologies for Air Quality: Particulate Matters and Bioaerosols. Chem Asian J. 2020;15(24):4241-55.

216	Stenlo M, Silva IAN, Hyllén S, Bölükbas DA, Niroomand A, Grins E, et al. Monitoring lung injury with particle flow rate in LPS- and COVID-19-induced ARDS. Physiol Rep. 2021;9(13):e14802.
217	Skubacz K, Hildebrandt R, Zgórska A, Dyduch Z, Samolej K, Smolinski A. Transport of Aerosols in Underground Mine Workings in Terms of SARS-CoV-2 Virus Threat. Molecules. 2021;26(12).
218	Singh A, Salunke P, Chhabra R, Sethi S, Sahoo SK, Karthigeyan M, et al. The Risk of Spread of Infection During Craniotomy/Craniostomy on Patients with Active Coronavirus Disease 2019 (COVID-19) Infection: Myth or Fact? World Neurosurg. 2021;147:e272-e4.
219	Shimazaki Y, Okubo M, Yamamoto T. Three-dimensional Numerical Simulation of Gas-particulate Flow around Breathing Human and Particulate Inhalation. AIP Conf Proc. 2006;832(1):439-44.
220	Setti L, Passarini F, De Gennaro G, Barbieri P, Perrone MG, Borelli M, et al. SARS-Cov-2RNA found on particulate matter of Bergamo in Northern Italy: First evidence. Environ Res. 2020;188:109754.
221	Selvam S, Muthukumar P, Venkatramanan S, Roy PD, Manikanda Bharath K, Jesuraja K. SARS-CoV-2 pandemic lockdown: Effects on air quality in the industrialized Gujarat state of India. Sci Total Environ. 2020;737:140391.
222	Sciomer S, Moscucci F, Magrì D, Badagliacca R, Piccirillo G, Agostoni P. SARS-CoV-2 spread in Northern Italy: what about the pollution role? Environ Monit Assess. 2020;192(6):325.
223	Scheier T, Shah C, Huber M, Sax H, Hasse B, Günthard HF, et al. Do we cause false positives? An experimental series on droplet or airborne SARS-CoV-2 contamination of sampling tubes during swab collection in a test center. Antimicrob Resist Infect Control. 2021;10(1):51.
224	Roy S, Saha M, Dhar B, Pandit S, Nasrin R. Geospatial analysis of COVID-19 lockdown effects on air quality in the South and Southeast Asian region. Sci Total Environ. 2021;756:144009.
225	Roviello V, Roviello GN. Lower COVID-19 mortality in Italian forested areas suggests immunoprotection by Mediterranean plants. Environ Chem Lett. 2020:1-12.
226	Rovetta A, Bhagavathula AS, Castaldo L. Modeling the Epidemiological Trend and Behavior of COVID-19 in Italy. Cureus. 2020;12(8):e9884.
227	Rodríguez-Urrego D, Rodríguez-Urrego L. Air quality during the COVID-19: PM(2.5) analysis in the 50 most polluted capital cities in the world. Environ Pollut. 2020;266(Pt 1):115042.
228	Reilly JP, Zhao Z, Shashaty MGS, Koyama T, Christie JD, Lanken PN, et al. Low to Moderate Air Pollutant Exposure and Acute Respiratory Distress Syndrome after Severe Trauma. Am J Respir Crit Care Med. 2019;199(1):62-70.
229	Ravenel TD, Kessler R, Comisi JC, Kelly A, Renne WG, Teich ST. Evaluation of the spatter-reduction effectiveness and aerosol containment of eight dry-field isolation techniques. Quintessence Int. 2020;51(8):660-70.
230	Querol X, Massagué J, Alastuey A, Moreno T, Gangoiti G, Mantilla E, et al. Lessons from the COVID-19 air pollution decrease in Spain: Now what? Sci Total Environ. 2021;779:146380.
231	Pozzer A, Dominici F, Haines A, Witt C, Münzel T, Lelieveld J. Regional and global contributions of air pollution to risk of death from COVID-19. Cardiovasc Res. 2020;116(14):2247-53.
232	Ponce de Leon S, Lazcano A. Panspermia--true or false? Lancet. 2003;362(9381):406-7; author reply 7-8.
233	Pierpaoli M, Giosuè C, Czerwińska N, Rycewicz M, Wieloszyńska A, Bogdanowicz R, et al. Characterization and Filtration Efficiency of Sustainable PLA Fibers Obtained via a Hybrid 3D-Printed/Electrospinning Technique. Materials (Basel). 2021;14(22).
234	Pearce E, Campen MJ, Baca JT, Blewett JP, Femling J, Hanson DT, et al. Aerosol generation with various approaches to oxygenation in healthy volunteers in the emergency department. J Am Coll Emerg Physicians Open. 2021;2(2):e12390.
235	Patel H, Talbot N, Salmond J, Dirks K, Xie S, Davy P. Implications for air quality management of changes in air quality during lockdown in Auckland (New Zealand) in response to the 2020 SARS-CoV-2 epidemic. Sci Total Environ. 2020;746:141129.
236	Parida BR, Bar S, Roberts G, Mandal SP, Pandey AC, Kumar M, et al. Improvement in air quality and its impact on land surface temperature in major urban areas across India during the first lockdown of the pandemic. Environ Res. 2021;199:111280.
237	Orak NH, Ozdemir O. The impacts of COVID-19 lockdown on PM(10) and SO(2) concentrations and association with human mobility across Turkey. Environ Res. 2021;197:111018.
238	Nugroho A, Saunar R, Lalisang TJM, Wiradisuria E. Local adaptation of laparoscopic smoke evacuator in COVID-19 pandemic situation. Asian J Endosc Surg. 2021;14(3):620-3.
239	Miller PW, Reesman C, Grossman MK, Nelson SA, Liu V, Wang P. Marginal warming associated with a COVID-19 quarantine and the implications for disease transmission. Sci Total Environ. 2021;780:146579.
240	Mettias B, Mair M, Conboy P. COVID-19 Cross-Infection Rate After Surgical Procedures: Incidence and Outcome. Laryngoscope. 2021;131(11):E2749-e54.
241	Mendy A, Wu X, Keller JL, Fassler CS, Apewokin S, Mersha TB, et al. Long-term exposure to fine particulate matter and hospitalization in COVID-19 patients. Respir Med. 2021;178:106313.
242	Mendez-Espinosa JF, Rojas NY, Vargas J, Pachón JE, Belalcazar LC, Ramírez O. Air quality variations in Northern South America during the COVID-19 lockdown. Sci Total Environ. 2020;749:141621.
243	Martorell-Marugán J, Villatoro-García JA, García-Moreno A, López-Domínguez R, Requena F, Merelo JJ, et al. DatAC: A visual analytics platform to explore climate and air quality indicators associated with the COVID-19 pandemic in Spain. Sci Total Environ. 2021;750:141424.
244	Martins G, Gogola JL, Budni LH, Janegitz BC, Marcolino-Junior LH, Bergamini MF. 3D-printed electrode as a new platform for electrochemical immunosensors for virus detection. Anal Chim Acta. 2021;1147:30-7.
245	Maestre JP, Jarma D, Yu JF, Siegel JA, Horner SD, Kinney KA. Distribution of SARS-CoV-2 RNA signal in a home with COVID-19 positive occupants. Sci Total Environ. 2021;778:146201.

246	Lovrić M, Pavlović K, Vuković M, Grange SK, Haberl M, Kern R. Understanding the true effects of the COVID-19 lockdown on air pollution by means of machine learning. Environ Pollut. 2021;274:115900.
247	López A, Fuentes E, Yusà V, López-Labrador FX, Camaró M, Peris-Martinez C, et al. Indoor Air Quality including Respiratory Viruses. Toxics. 2021;9(11).
248	Linillos-Pradillo B, Rancan L, Ramiro ED, Vara E, Artíñano B, Arias J. Determination of SARS-CoV-2 RNA in different particulate matter size fractions of outdoor air samples in Madrid during the lockdown. Environ Res. 2021;195:110863.
249	Lin S, Wei D, Sun Y, Chen K, Yang L, Liu B, et al. Region-specific air pollutants and meteorological parameters influence COVID-19: A study from mainland China. Ecotoxicol Environ Saf. 2020;204:111035.
250	Lepore E, Aguilera Benito P, Piña Ramírez C, Viccione G. Indoors ventilation in times of confinement by SARS-CoV-2 epidemic: A comparative approach between Spain and Italy. Sustain Cities Soc. 2021;72:103051.
251	Leão MLP, Penteado JO, Ulguim SM, Gabriel RR, Dos Santos M, Brum AN, et al. Health impact assessment of air pollutants during the COVID-19 pandemic in a Brazilian metropolis. Environ Sci Pollut Res Int. 2021;28(31):41843-50.
252	Lawrence RJ, O'Donoghue GM, Kitterick P, Hartley DEH. Use of a novel drape 'tent' as an infection prevention control measure for mastoid surgery. J Laryngol Otol. 2020;134(12):1115-7.
253	Land WG. Role of Damage-Associated Molecular Patterns in Light of Modern Environmental Research: A Tautological Approach. Int J Environ Res. 2020;14(5):583-604.
254	Lai AC, Poon CK, Cheung AC. Effectiveness of facemasks to reduce exposure hazards for airborne infections among general populations. J R Soc Interface. 2012;9(70):938-48.
255	Kyomba GK, Konde JNN, Saila-Ngita D, Solo TK, Kiyombo GM. Assessing the management of healthcare waste for disease prevention and environment protection at selected hospitals in Kinshasa, Democratic Republic of Congo. Waste Manag Res. 2021;39(10):1237-44.
256	Kumari P, Toshniwal D. Impact of lockdown on air quality over major cities across the globe during COVID-19 pandemic. Urban Clim. 2020;34:100719.
257	Kumar P, Hama S, Omidvarborna H, Sharma A, Sahani J, Abhijith KV, et al. Temporary reduction in fine particulate matter due to 'anthropogenic emissions switch-off' during COVID-19 lockdown in Indian cities. Sustain Cities Soc. 2020;62:102382.
258	Kudryashova OB, Muravlev EV, Antonnikova AA, Titov SS. Propagation of viral bioaerosols indoors. PLoS One. 2021;16(1):e0244983.
259	Khan TR, Parker DS, Withers C. Mitigation of Airborne Contaminant Spread through Simple Interventions in an Occupied Single-Family Home. Int J Environ Res Public Health. 2021;18(11).
260	Kerimray A, Baimatova N, Ibragimova OP, Bukenov B, Kenessov B, Plotitsyn P, et al. Assessing air quality changes in large cities during COVID-19 lockdowns: The impacts of traffic-free urban conditions in Almaty, Kazakhstan. Sci Total Environ. 2020;730:139179.
261	Kayalar O, Arı A, Babuçcu G, Konyalılar N, Doğan O, Can F, et al. Existence of SARS-CoV-2 RNA on ambient particulate matter samples: A nationwide study in Turkey. Sci Total Environ. 2021;789:147976.
262	Kasloff SB, Leung A, Strong JE, Funk D, Cutts T. Stability of SARS-CoV-2 on critical personal protective equipment. Sci Rep. 2021;11(1):984.
263	Jakovljević I, Strukil ZS, Godec R, Davila S, Pehnec G. Influence of lockdown caused by the COVID-19 pandemic on air pollution and carcinogenic content of particulate matter observed in Croatia. Air Qual Atmos Health. 2021;14(4):467-72.
264	Islam MS, Larpruenrudee P, Saha SC, Pourmehran O, Paul AR, Gemci T, et al. How severe acute respiratory syndrome coronavirus-2 aerosol propagates through the age-specific upper airways. Phys Fluids (1994). 2021;33(8):081911.
265	In 't Veen J, Kappen JH, van Schayck OCP. [Air pollution: a determinant for COVID-19?]. Ned Tijdschr Geneeskd. 2020;164.
266	Huang KL, Chen CW, Chu SJ, Perng WC, Wu CP. Systemic inflammation caused by white smoke inhalation in a combat exercise. Chest. 2008;133(3):722-8.
267	Hokajärvi AM, Rytkönen A, Tiwari A, Kauppinen A, Oikarinen S, Lehto KM, et al. The detection and stability of the SARS-CoV-2 RNA biomarkers in wastewater influent in Helsinki, Finland. Sci Total Environ. 2021;770:145274.
268	He S, Han J. Electrostatic fine particles emitted from laser printers as potential vectors for airborne transmission of COVID-19. Environ Chem Lett. 2020:1-8.
269	Feng Z, Cao SJ, Wang J, Kumar P, Haghighat F. Indoor airborne disinfection with electrostatic disinfector (ESD): Numerical simulations of ESD performance and reduction of computing time. Build Environ. 2021;200:107956.
270	Fabiani L, Saroglia M, Galatà G, De Santis R, Fillo S, Luca V, et al. Magnetic beads combined with carbon black-based screen-printed electrodes for COVID-19: A reliable and miniaturized electrochemical immunosensor for SARS-CoV-2 detection in saliva. Biosens Bioelectron. 2021;171:112686.
271	Dunker S, Hornick T, Szczepankiewicz G, Maier M, Bastl M, Bumberger J, et al. No SARS-CoV-2 detected in air samples (pollen and particulate matter) in Leipzig during the first spread. Sci Total Environ. 2021;755(Pt 1):142881.
272	Duffy C, Kidd A, Francis S, Tsim S, McNaughton L, Ferguson K, et al. Chest drain aerosol generation in COVID-19 and emission reduction using a simple anti-viral filter. BMJ Open Respir Res. 2020;7(1).
273	Domínguez-Amarillo S, Fernández-Agüera J, Cesteros-García S, González-Lezcano RA. Bad Air Can Also Kill: Residential Indoor Air Quality and Pollutant Exposure Risk during the COVID-19 Crisis. Int J Environ Res Public Health. 2020;17(19).

274	Doggett N, Chow CW, Mubareka S. Characterization of Experimental and Clinical Bioaerosol Generation During Potential Aerosol-Generating Procedures. Chest. 2020;158(6):2467-73.
275	Dey P, Saha SK, Sarkar S. Study of the interactions of sneezing droplets with particulate matter in a polluted environment. Phys Fluids (1994). 2021;33(11):113310.
276	Derrick JL, Gomersall CD. Surgical helmets and SARS infection. Emerg Infect Dis. 2004;10(2):277-9.
277	de Rooij MMT, Hakze-Van der Honing RW, Hulst MM, Harders F, Engelsma M, van de Hoef W, et al. Occupational and environmental exposure to SARS-CoV-2 in and around infected mink farms. Occup Environ Med. 2021;78(12):893-9.
278	Dave GS, Rakholiya KD, Kaneria MJ, Galvadiya BP, Vyas SR, Kanbi VH, et al. High affinity interaction of Solanum tuberosum and Brassica juncea residue smoke water compounds with proteins involved in coronavirus infection. Phytother Res. 2020;34(12):3400-10.
279	Das D, Ramachandran G. Risk analysis of different transport vehicles in India during COVID-19 pandemic. Environ Res. 2021;199:111268.
280	Das A, Mitra S, Kumar S, Sengupta A. Two-drape closed pocket technique: minimizing aerosolization in mastoid exploration during COVID-19 pandemic. Eur Arch Otorhinolaryngol. 2020;277(12):3529-32.
281	Cui Y, Zhang ZF, Froines J, Zhao J, Wang H, Yu SZ, et al. Air pollution and case fatality of SARS in the People's Republic of China: an ecologic study. Environ Health. 2003;2(1):15.
282	Crane-Godreau MA, Clem KJ, Payne P, Fiering S. Vitamin D Deficiency and Air Pollution Exacerbate COVID-19 Through Suppression of Antiviral Peptide LL37. Front Public Health. 2020;8:232.
283	Collivignarelli MC, Abbà A, Bertanza G, Pedrazzani R, Ricciardi P, Carnevale Miino M. Lockdown for CoViD-2019 in Milan: What are the effects on air quality? Sci Total Environ. 2020;732:139280.
284	Colacci A, Bortone G, Maffei G, Marchesi S, Mescoli A, Parmagnani F, et al. Environmental pollution and COVID-19: the molecular terms and predominant disease outcomes of their sweetheart agreement. Epidemiol Prev. 2020;44(5-6 Suppl 2):169-82.
285	Coccia M. Effects of the spread of COVID-19 on public health of polluted cities: results of the first wave for explaining the dejà vu in the second wave of COVID-19 pandemic and epidemics of future vital agents. Environ Sci Pollut Res Int. 2021;28(15):19147-54.
286	Coccia M. How do low wind speeds and high levels of air pollution support the spread of COVID-19? Atmos Pollut Res. 2021;12(1):437-45.
287	Coccia M. Factors determining the diffusion of COVID-19 and suggested strategy to prevent future accelerated viral infectivity similar to COVID. Sci Total Environ. 2020;729:138474.
288	Ciglenečki I, Orlović-Leko P, Vidović K, Tasić V. The possible role of the surface active substances (SAS) in the airborne transmission of SARS-CoV-2. Environ Res. 2021;198:111215.
289	Cicuttin E, Cobianchi L, Chiarugi M, Catena F, Coccolini F, Pietrabissa A. Detect to protect: pneumoperitoneum gas samples for SARS-CoV-2 and biohazard testing. Surg Endosc. 2020;34(7):2863-5.
290	Chow TT, Kwan A, Lin Z, Bai W. Conversion of operating theatre from positive to negative pressure environment. J Hosp Infect. 2006;64(4):371-8.
291	Chiu YJ, Ma H, Liao WC, Shih YC, Chen MC, Shih CC, et al. Extracorporeal membrane oxygenation support may be a lifesaving modality in patients with burn and severe acute respiratory distress syndrome: Experience of Formosa Water Park dust explosion disaster in Taiwan. Burns. 2018;44(1):118-23.
292	Chia PY, Coleman KK, Tan YK, Ong SWX, Gum M, Lau SK, et al. Detection of air and surface contamination by SARS-CoV-2 in hospital rooms of infected patients. Nat Commun. 2020;11(1):2800.
293	Chen Y, Zhang S, Peng C, Shi G, Tian M, Huang RJ, et al. Impact of the COVID-19 pandemic and control measures on air quality and aerosol light absorption in Southwestern China. Sci Total Environ. 2020;749:141419.
294	Calfee CS, Matthay MA, Eisner MD, Benowitz N, Call M, Pittet JF, et al. Active and passive cigarette smoking and acute lung injury after severe blunt trauma. Am J Respir Crit Care Med. 2011;183(12):1660-5.
295	Cahyadi MN, Handayani HH, Warmadewanthi I, Rokhmana CA, Sulistiawan SS, Waloedjo CS, et al. Spatiotemporal Analysis for COVID-19 Delta Variant Using GIS-Based Air Parameter and Spatial Modeling. Int J Environ Res Public Health. 2022;19(3).
296	Bui TT, Shin MK, Jee SY, Long DX, Hong J, Kim MG. Ferroelectric PVDF nanofiber membrane for high-efficiency PM0.3 air filtration with low air flow resistance. Colloids Surf A Physicochem Eng Asp. 2022;640:128418.
297	Bryant J, Tobias JD. Enclosure with augmented airflow to decrease risk of exposure to aerosolized pathogens including coronavirus during endotracheal intubation. Can the reduction in aerosolized particles be quantified? Paediatr Anaesth. 2020;30(8):900-4.
298	Brocke SA, Billings GT, Taft-Benz S, Alexis NE, Heise MT, Jaspers I. Woodsmoke particle exposure prior to SARS-CoV-2 infection alters antiviral response gene expression in human nasal epithelial cells in a sex-dependent manner. Am J Physiol Lung Cell Mol Physiol. 2022.
299	Briz-Redón A, Belenguer-Sapiña C, Serrano-Aroca A. A city-level analysis of PM(2.5) pollution, climate and COVID-19 early spread in Spain. J Environ Health Sci Eng. 2022:1-9.
300	Briz-Redón A, Belenguer-Sapiña C, Serrano-Aroca A. Changes in air pollution during COVID-19 lockdown in Spain: A multi-city study. J Environ Sci (China). 2021;101:16-26.
301	Bostanci Ceran B, Karakoç A, Taciroğlu E. Airborne pathogen projection during ophthalmic examination. Graefes Arch Clin Exp Ophthalmol. 2020;258(10):2275-82.
302	Borro M, Di Girolamo P, Gentile G, De Luca O, Preissner R, Marcolongo A, et al. Evidence-Based Considerations Exploring Relations between SARS-CoV-2 Pandemic and Air Pollution: Involvement of PM2.5-Mediated Up-Regulation of the Viral Receptor ACE-2. Int J Environ Res Public Health. 2020;17(15).

303	Bherwani H, Gautam S, Gupta A. Qualitative and quantitative analyses of impact of COVID-19 on sustainable development goals (SDGs) in Indian subcontinent with a focus on air quality. Int J Environ Sci Technol (Tehran). 2021:1-10.
304	Beig G, Korhale N, Rathod A, Maji S, Sahu SK, Dole S, et al. On modelling growing menace of household emissions under COVID-19 in Indian metros. Environ Pollut. 2021;272:115993.
305	Baron YM. Could changes in the airborne pollutant particulate matter acting as a viral vector have exerted selective pressure to cause COVID-19 evolution? Med Hypotheses. 2021;146:110401.
306	Baron YM. Are there medium to short-term multifaceted effects of the airborne pollutant PM(2.5) determining the emergence of SARS-CoV-2 variants? Med Hypotheses. 2021;158:110718.
307	Banik RK, Ulrich A. Evidence of Short-Range Aerosol Transmission of SARS-CoV-2 and Call for Universal Airborne Precautions for Anesthesiologists During the COVID-19 Pandemic. Anesth Analg. 2020;131(2):e102-e4.
308	Anil I, Alagha O. The impact of COVID-19 lockdown on the air quality of Eastern Province, Saudi Arabia. Air Qual Atmos Health. 2021;14(1):117-28.
309	Anil I, Alagha O. Source Apportionment of Ambient Black Carbon During the COVID-19 Lockdown. Int J Environ Res Public Health. 2020;17(23).
310	Alvarez AE, Marson FA, Bertuzzo CS, Arns CW, Ribeiro JD. Epidemiological and genetic characteristics associated with the severity of acute viral bronchiolitis by respiratory syncytial virus. J Pediatr (Rio J). 2013;89(6):531-43.
311	Aloufi N, Traboulsi H, Ding J, Fonseca GJ, Nair P, Huang SK, et al. Angiotensin-converting enzyme 2 expression in COPD and IPF fibroblasts: the forgotten cell in COVID-19. Am J Physiol Lung Cell Mol Physiol. 2021;320(1):L152-l7.
312	Ali SM, Malik F, Anjum MS, Siddiqui GF, Anwar MN, Lam SS, et al. Exploring the linkage between PM(2.5) levels and COVID-19 spread and its implications for socio-economic circles. Environ Res. 2021;193:110421.
313	Adams MD. Air pollution in Ontario, Canada during the COVID-19 State of Emergency. Sci Total Environ. 2020;742:140516.
314	Zuniga-Montanez R, Coil DA, Eisen JA, Pechacek R, Guerrero RG, Kim M, et al. The challenge of SARS-CoV-2 environmental monitoring in schools using floors and portable HEPA filtration units: Fresh or relic RNA? medRxiv. 2021;15.
315	Rocha CA, Marques EV, Dos Santos RP, de Santiago IS, Cavalcante CLA, Cassiano DR, et al. A better understanding of air quality resulting from the effects of the 2020 pandemic in a city in the equatorial region (Fortaleza, Brazil). Environmental science and pollution research international. 2021;08.
316	Huang WC, Zhou S, He X, Chiem K, Mabrouk MT, Nissly RH, et al. SARS-CoV-2 RBD Neutralizing Antibody Induction is Enhanced by Particulate Vaccination. Advanced materials (Deerfield Beach, Fla). 2020;32(50):e2005637.
317	Carbone M, Lednicky J, Xiao SY, Venditti M, Bucci E. Coronavirus 2019 Infectious Disease Epidemic: Where We Are, What Can Be Done and Hope For. Journal of Thoracic Oncology. 2021;16(4):546-71.
